# Supplementary material for: Uncovering biomarkers for chronic toxoplasmosis detection highlights alternative pathways shaping parasite dormancy
Source: EMBO Mol Med. 2025 May 19;17(7):1686–715. doi: 10.1038/s44321-025-00252-0 (PMC12254245; doi:10.1038/s44321-025-00252-0)
Supplement: Supplementary file 6 — Movie EV1 [file 44321_2025_252_MOESM6_ESM.zip › Movie EV1/Legend.docx]

**Movie EV1 – Three-dimensional Deconvoluted Visualization of BSM Detection in a Brain Cyst by immunofluorescence assay.** 3D deconvoluted image of a brain cyst in a mouse chronically infected with the Toxoplasma gondii type II strain (76K). BSM (red) was detected by immunofluorescence assay. Nuclei were counterstained with the DNA-specific Hoechst dye (blue).
