# Supplementary material for: Uncovering biomarkers for chronic toxoplasmosis detection highlights alternative pathways shaping parasite dormancy
Source: EMBO Mol Med. 2025 May 19;17(7):1686–715. doi: 10.1038/s44321-025-00252-0 (PMC12254245; doi:10.1038/s44321-025-00252-0)

5/02/21

WB IP Flag RH 202020 · HF · Morc MAID avec IAA 24h

MW - E1 (ou E2)

Serum souris

CD1
ME49 (2)
inf 11 semaines
BCLA⊕

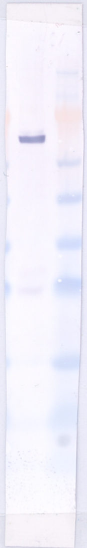

CD1
ME49 (3)
inf 11 semaines
BCLA ⊖

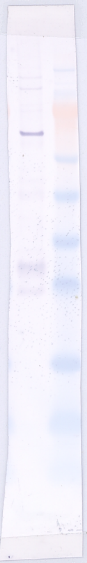

NMRI
76K (5)
56 W
BCLA ⊕

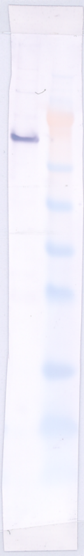

NMRI
76K (2)
56 W
BCLA ⊕

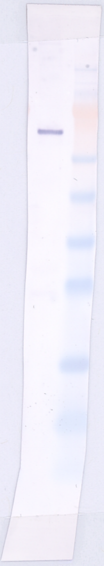

NMRI
76K (4)
56 W
BCLA ⊕

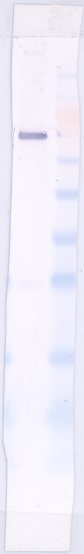

NMRI
76K (3)
56 W
BCLA ⊕

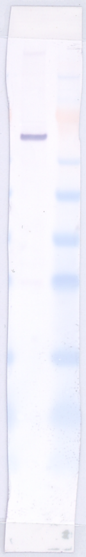

NMRI
76K Δ147 (5)
56 W
BCLA ⊖

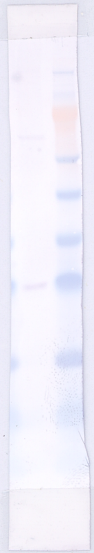

NMRI
76K Δ147 (6)
56 W
BCLA ⊖

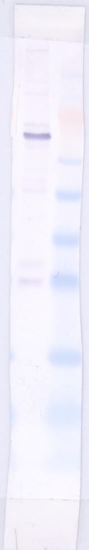

Δ min 142 n°80
7 W
BCLA ⊕

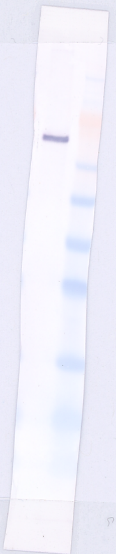

Projet
Δ min 142 n°48
NI

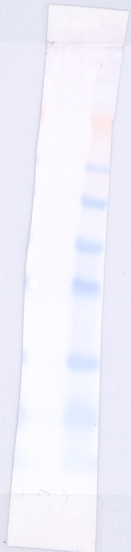

5/02/21 feuille n°2

WB IP Flag RH 202020 · HF · MORC MAID avec IAA 24h

MW · E1 (ou E2)

Serum souris

32W
BCLA⊖

NMRI
CTG ③
32

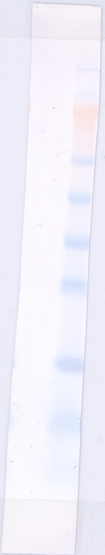

NMRI
CTG ④
32W
BCLA⊖

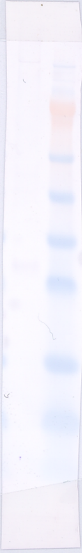

Supplement: Supplementary file 7 — Source data Fig. 1 [file 44321_2025_252_MOESM7_ESM.zip › Figure 1 Source Data/1e/1e.pdf]
