# Supplementary figures and images for: Uncovering biomarkers for chronic toxoplasmosis detection highlights alternative pathways shaping parasite dormancy (part 1 of 2)
Source: EMBO Mol Med. 2025 May 19;17(7):1686–715. doi: 10.1038/s44321-025-00252-0 (PMC12254245; doi:10.1038/s44321-025-00252-0)

**a**

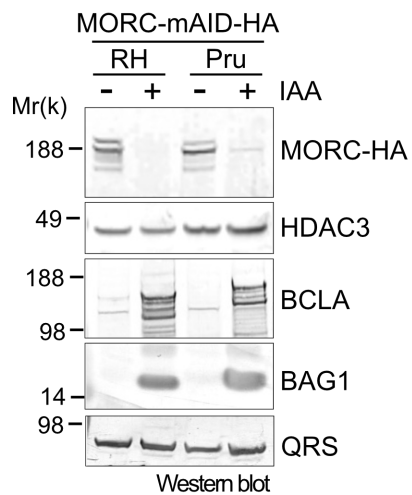

**CRC = MORC**

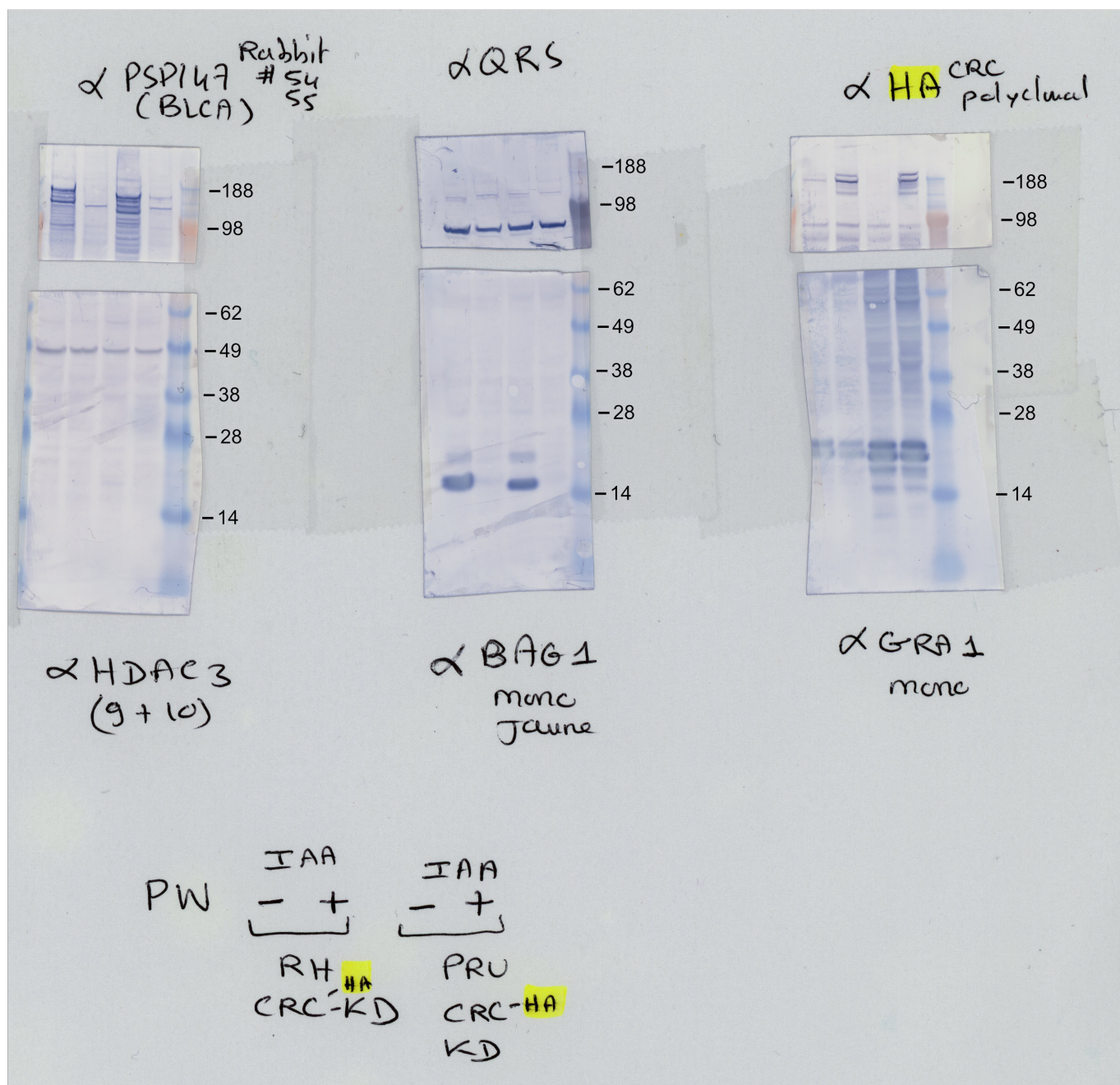

Supplement: Supplementary file 7 — Source data Fig. 1 [file 44321_2025_252_MOESM7_ESM.zip › Figure 1 Source Data/1a/1a.pdf]

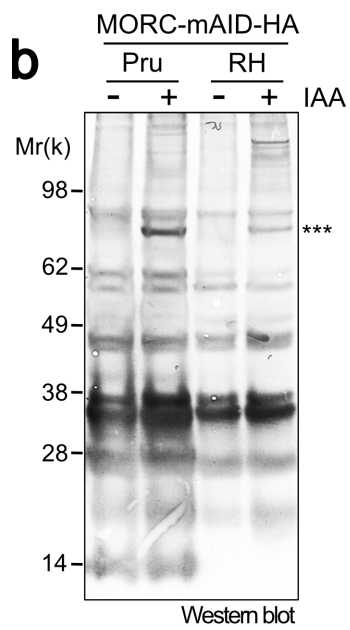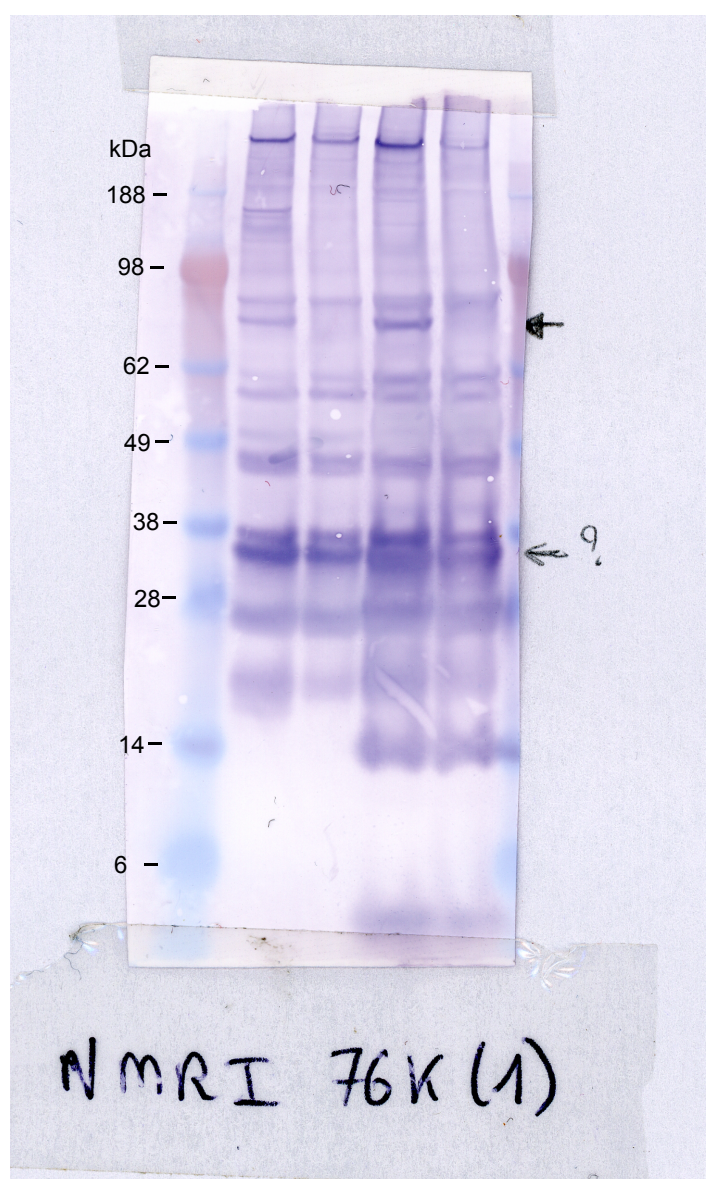

Supplement: Supplementary file 7 — Source data Fig. 1 [file 44321_2025_252_MOESM7_ESM.zip › Figure 1 Source Data/1b/1b.pdf]

**d**

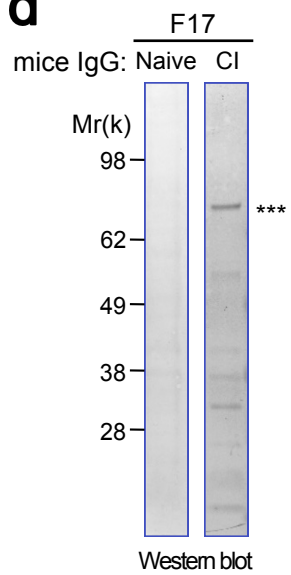

NI (min 142-48)

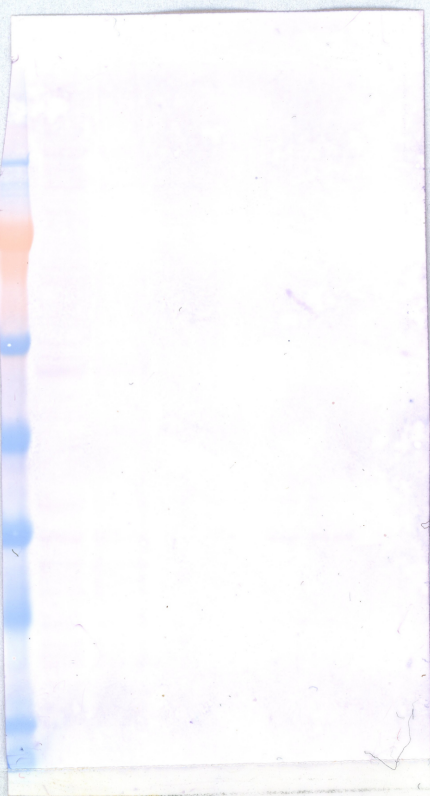

BCLA⊕ (min 142-80)

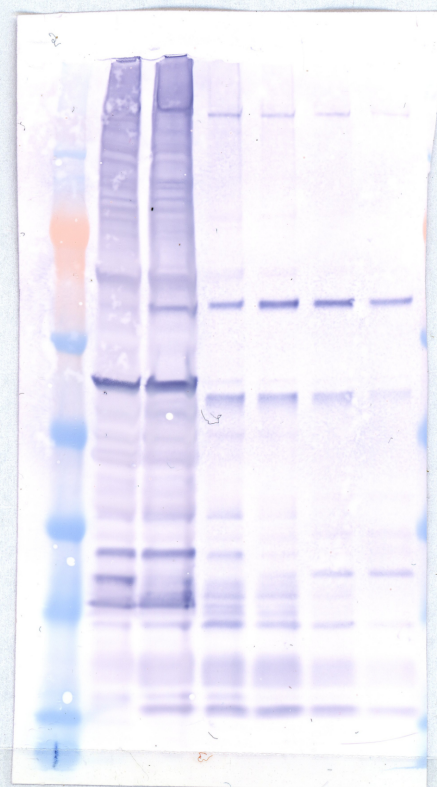

Supplement: Supplementary file 7 — Source data Fig. 1 [file 44321_2025_252_MOESM7_ESM.zip › Figure 1 Source Data/1d/1d.pdf]

C

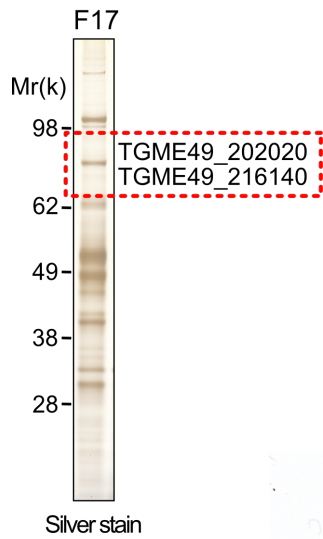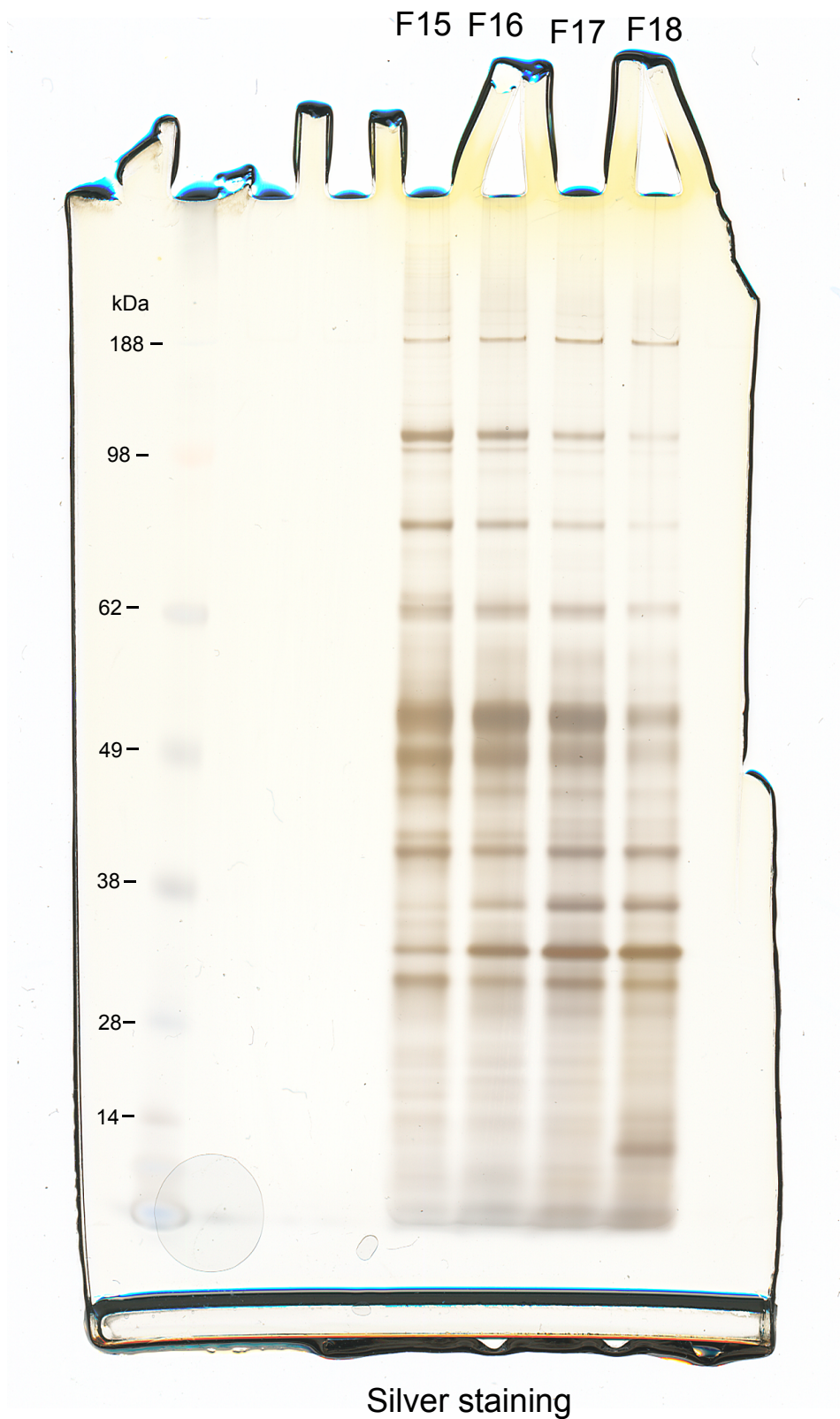

Supplement: Supplementary file 7 — Source data Fig. 1 [file 44321_2025_252_MOESM7_ESM.zip › Figure 1 Source Data/1c/1c.pdf]

f

Pruku80 BFD1-HAFlag

FR235222 (h)

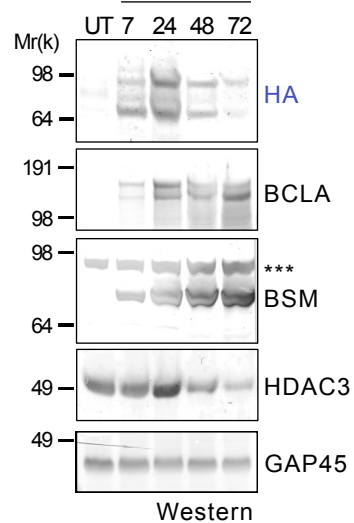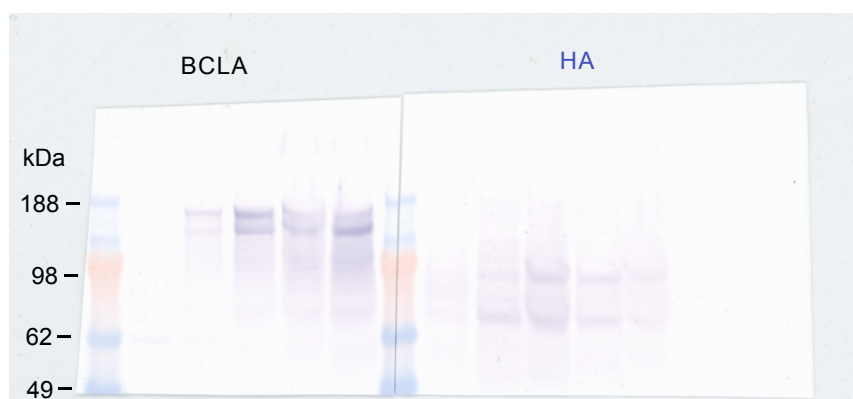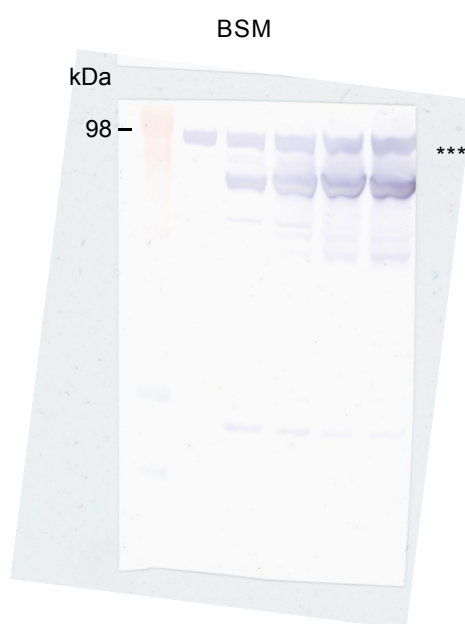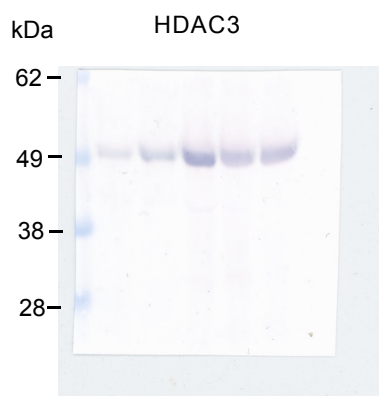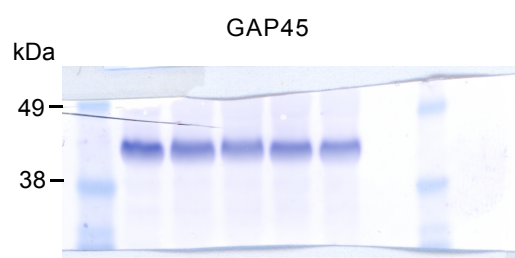

Supplement: Supplementary file 8 — Source data Fig. 2 [file 44321_2025_252_MOESM8_ESM.zip › Figure 2 Source Data/2f/2f.pdf]

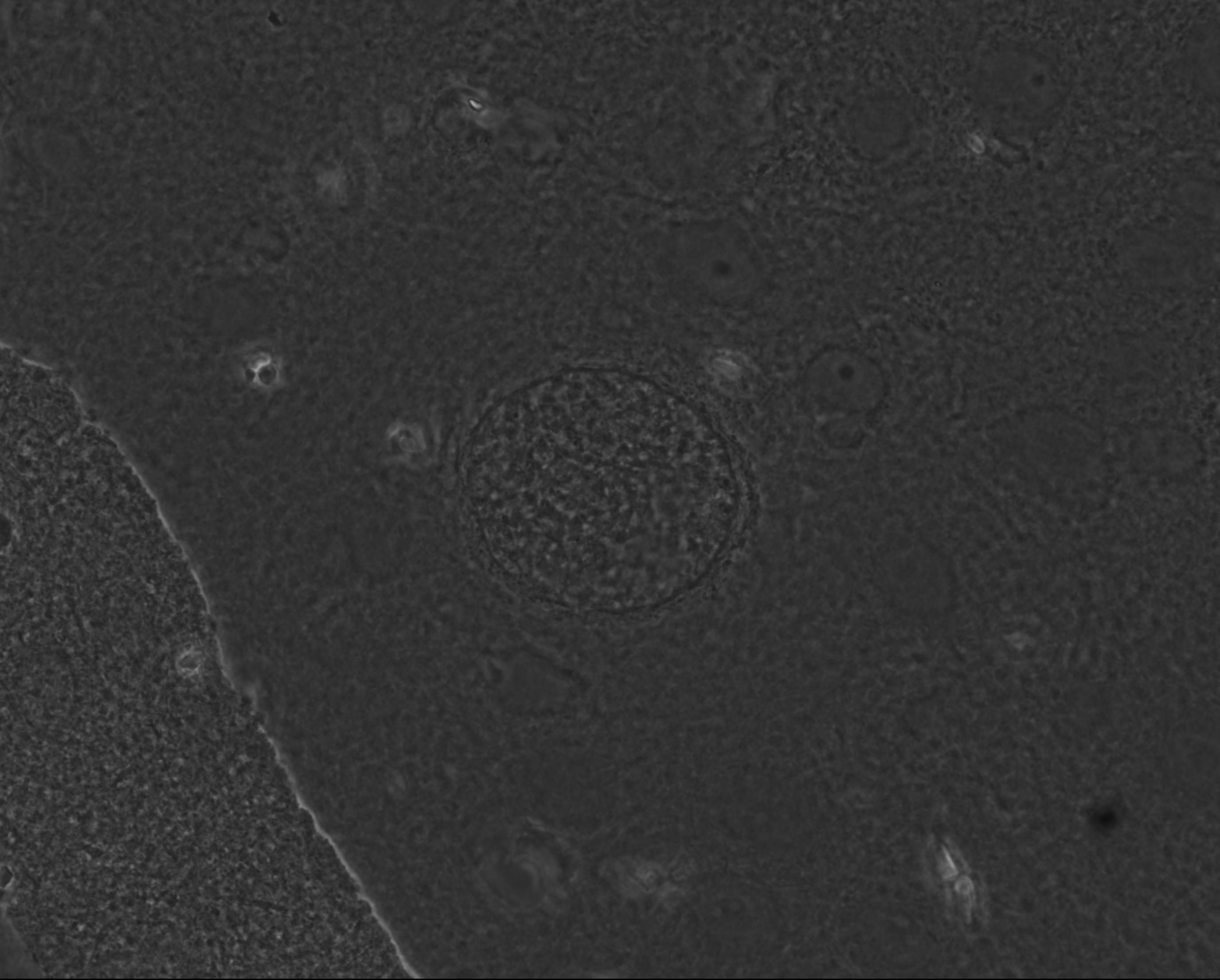

Supplement: Supplementary file 8 — Source data Fig. 2 [file 44321_2025_252_MOESM8_ESM.zip › Figure 2 Source Data/2c/BSM kyste 3 (Phase).png]

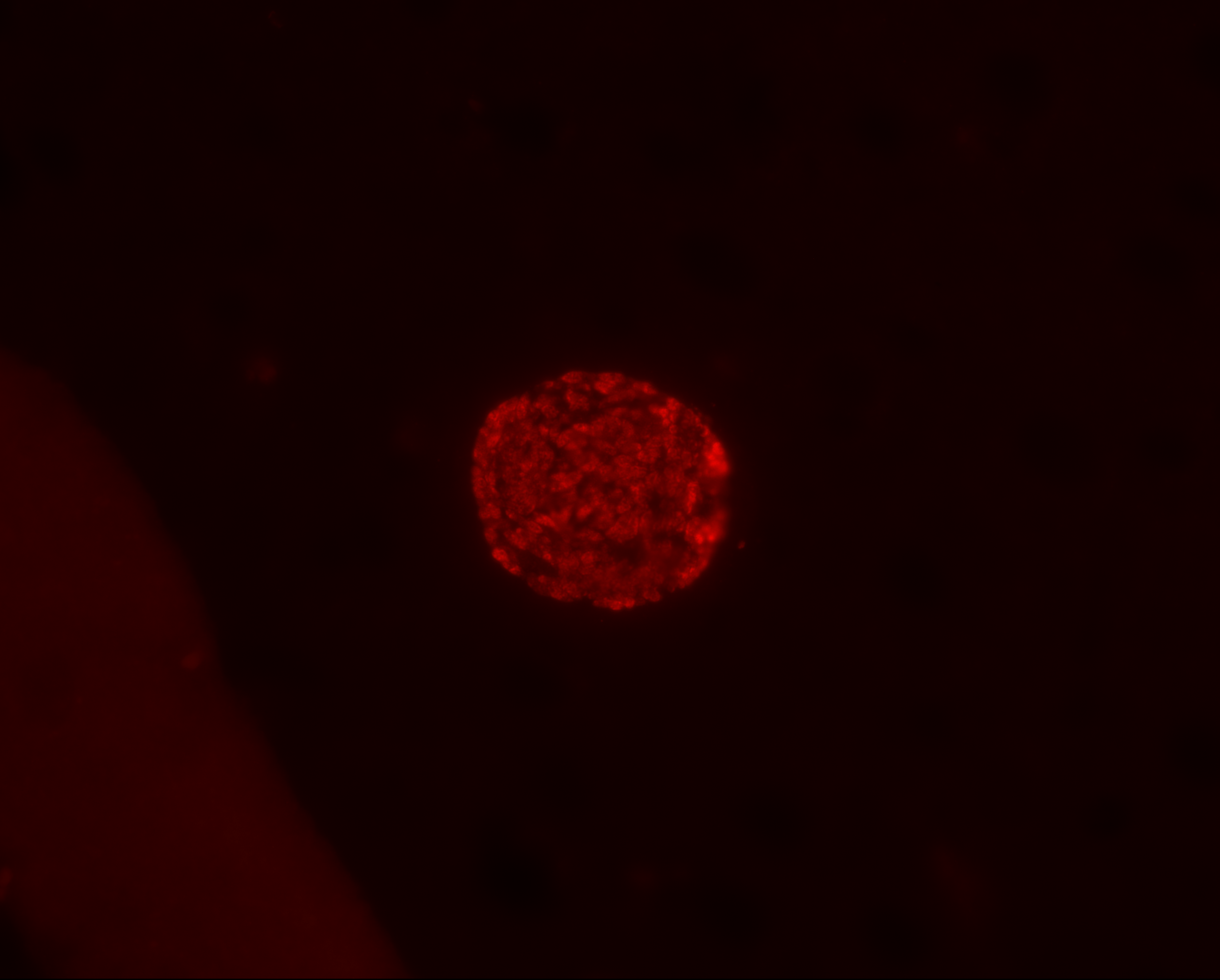

Supplement: Supplementary file 8 — Source data Fig. 2 [file 44321_2025_252_MOESM8_ESM.zip › Figure 2 Source Data/2c/BSM kyste 3 (BSM in red).png]

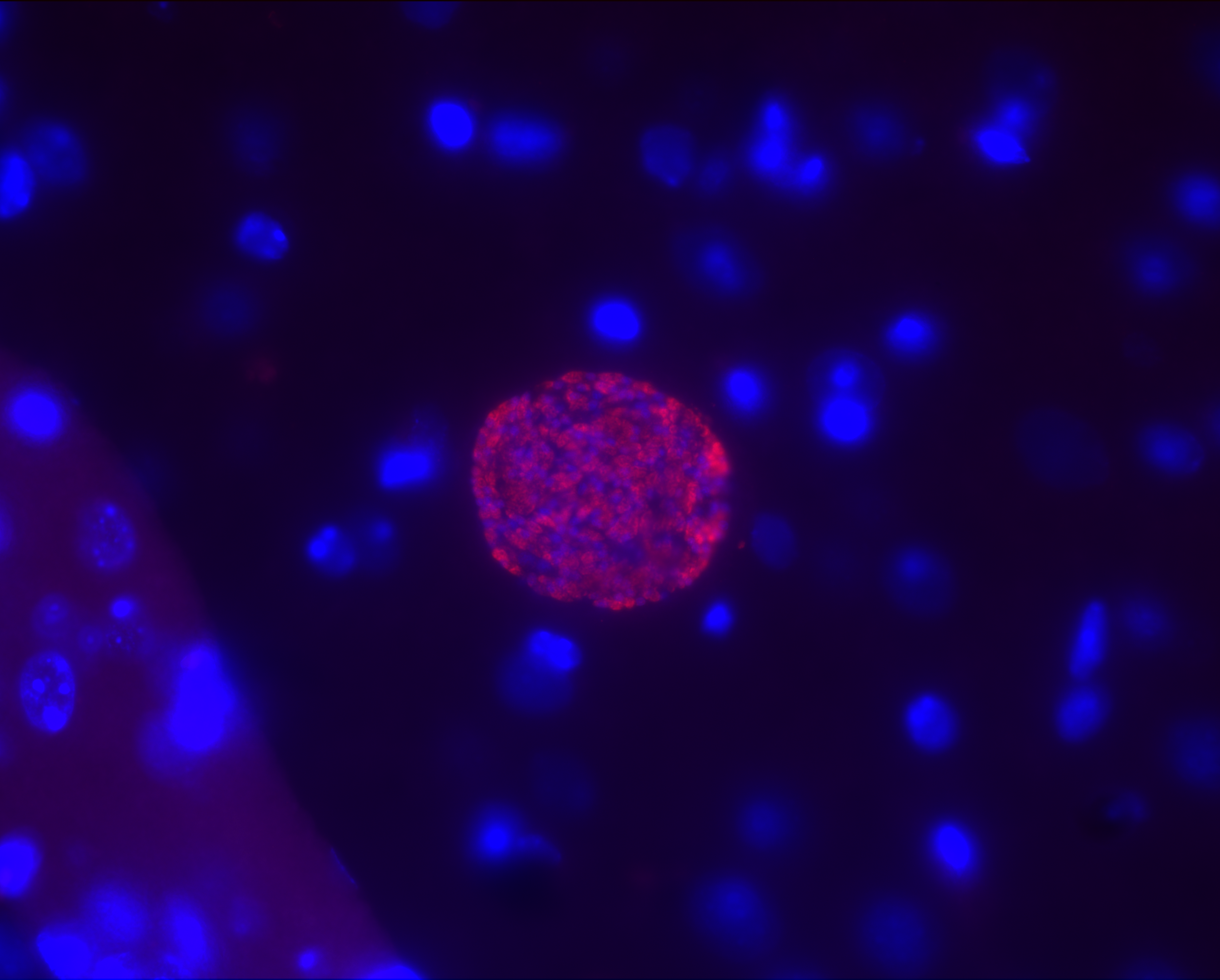

Supplement: Supplementary file 8 — Source data Fig. 2 [file 44321_2025_252_MOESM8_ESM.zip › Figure 2 Source Data/2c/BSM kyste 3 (DNA in blue BSM in red).png]

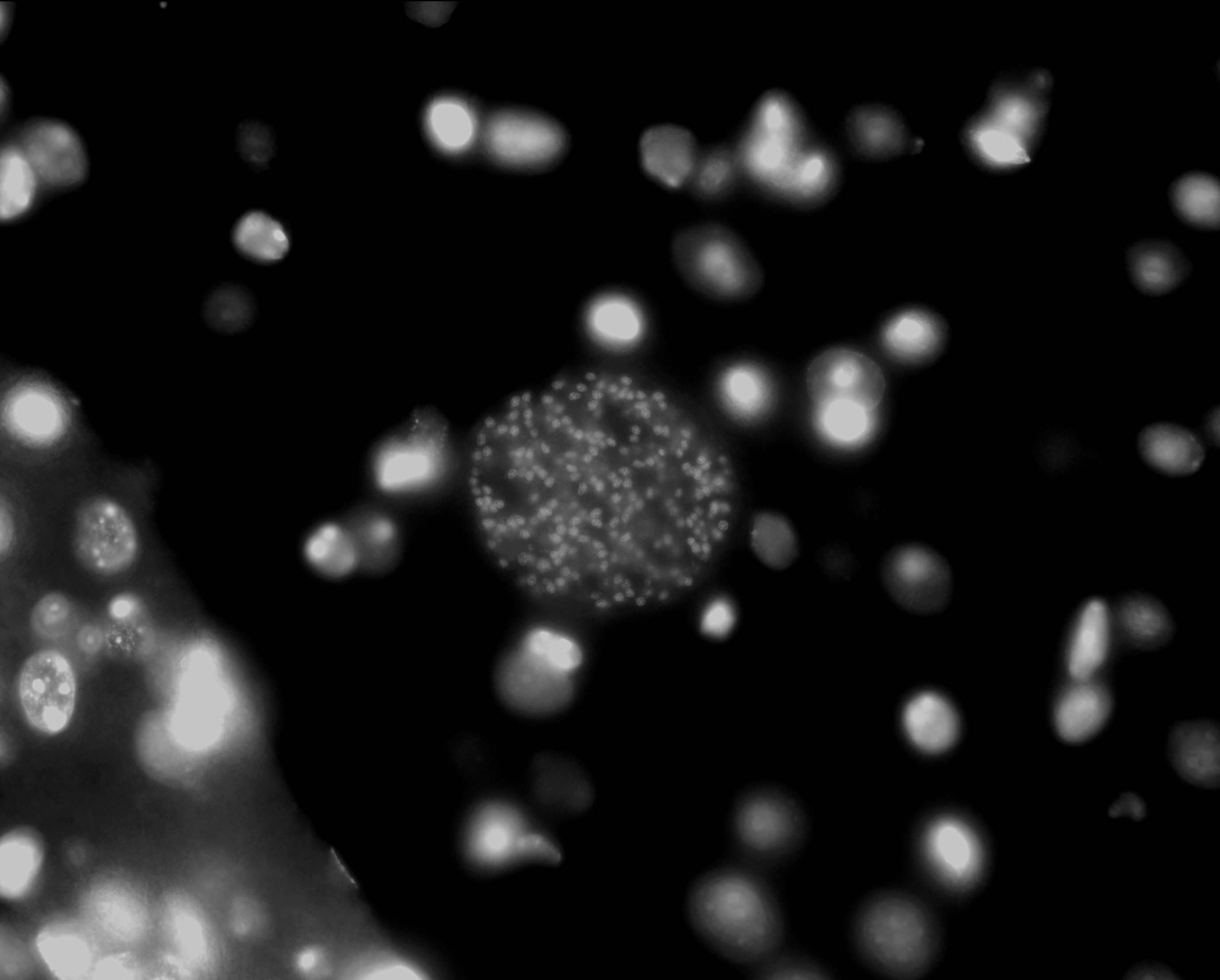

Supplement: Supplementary file 8 — Source data Fig. 2 [file 44321_2025_252_MOESM8_ESM.zip › Figure 2 Source Data/2c/BSM kyste 3 (DNA BW)png.png]

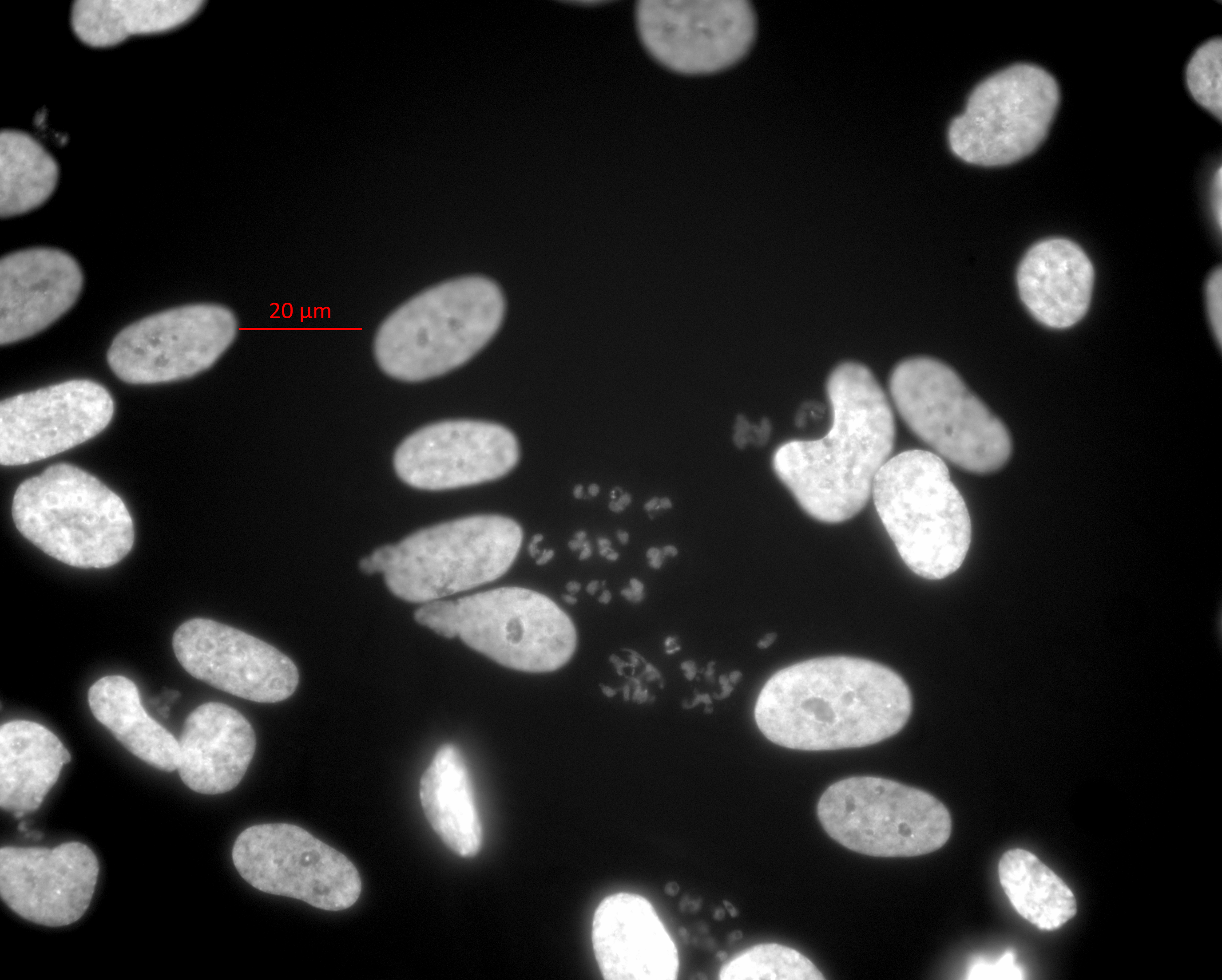

Supplement: Supplementary file 8 — Source data Fig. 2 [file 44321_2025_252_MOESM8_ESM.zip › Figure 2 Source Data/2a/image IAA 48h/Snap-3966_c2 (DNA).tif]

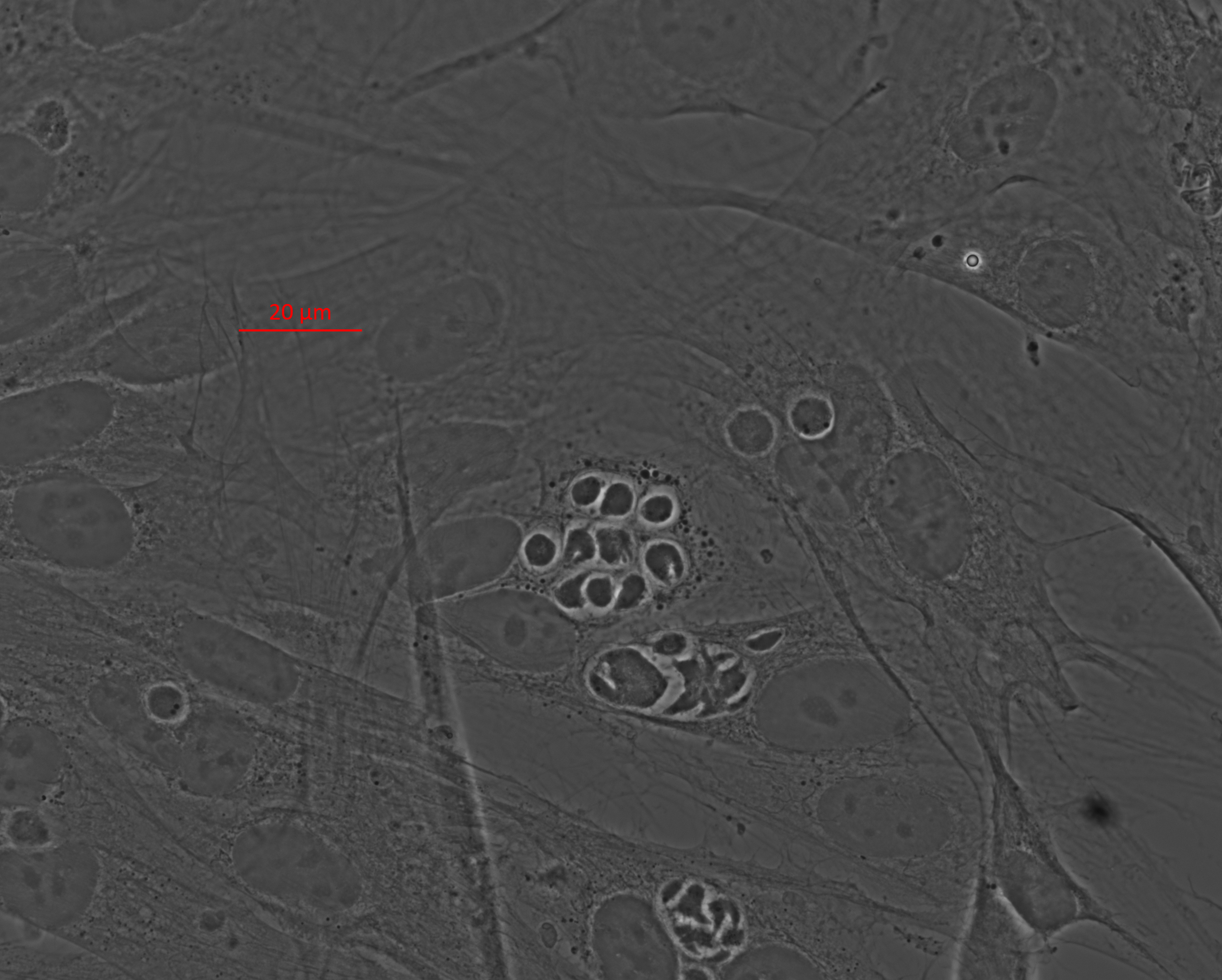

Supplement: Supplementary file 8 — Source data Fig. 2 [file 44321_2025_252_MOESM8_ESM.zip › Figure 2 Source Data/2a/image IAA 48h/Snap-3966_c1 (Phase).tif]

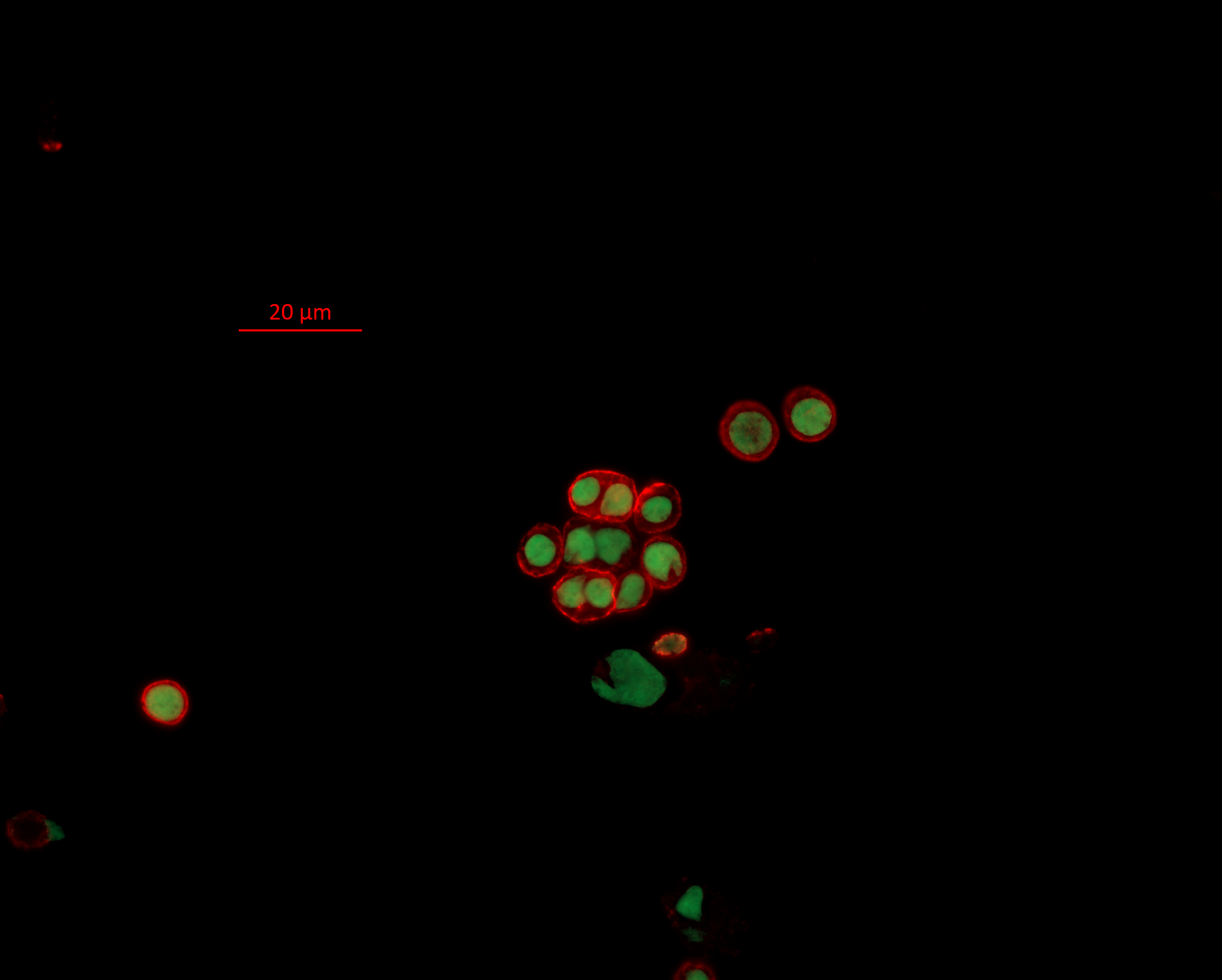

Supplement: Supplementary file 8 — Source data Fig. 2 [file 44321_2025_252_MOESM8_ESM.zip › Figure 2 Source Data/2a/image IAA 48h/Snap-3966_c3+4 (DBA BSM).tif]

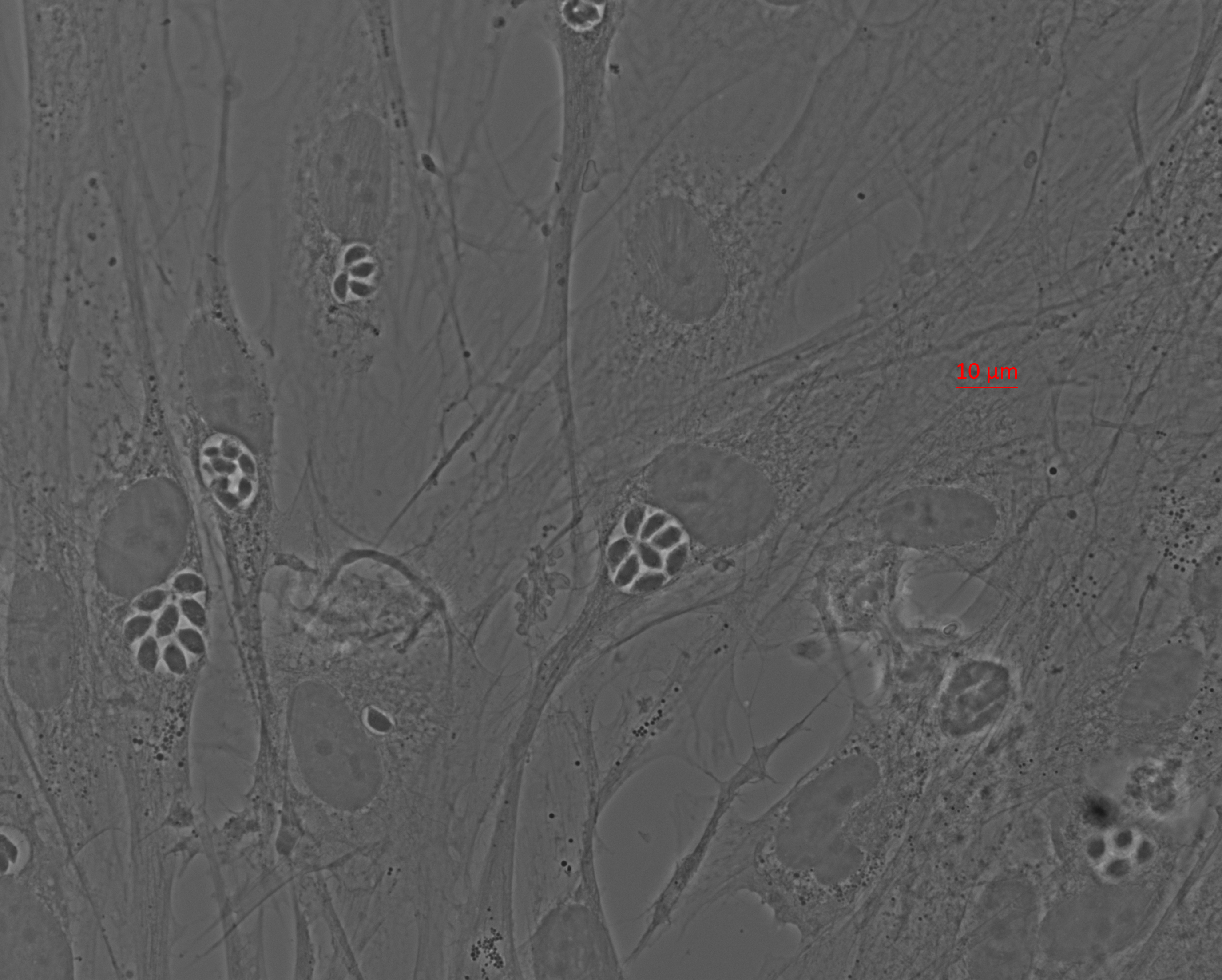

Supplement: Supplementary file 8 — Source data Fig. 2 [file 44321_2025_252_MOESM8_ESM.zip › Figure 2 Source Data/2a/image UT/Snap-3976_c1 (Phase).tif]

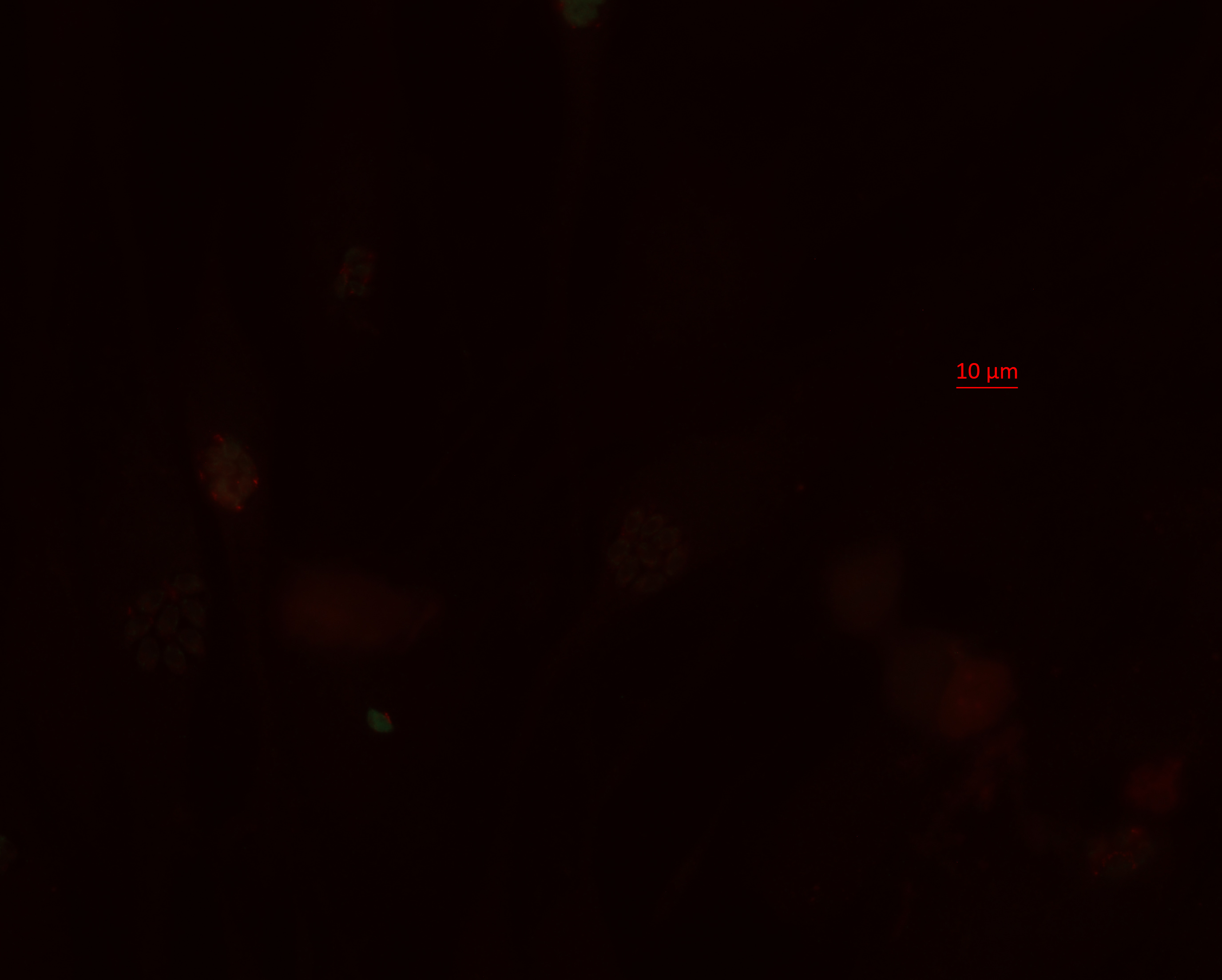

Supplement: Supplementary file 8 — Source data Fig. 2 [file 44321_2025_252_MOESM8_ESM.zip › Figure 2 Source Data/2a/image UT/Snap-3976_c3+4 (DBA+BSM).tif]

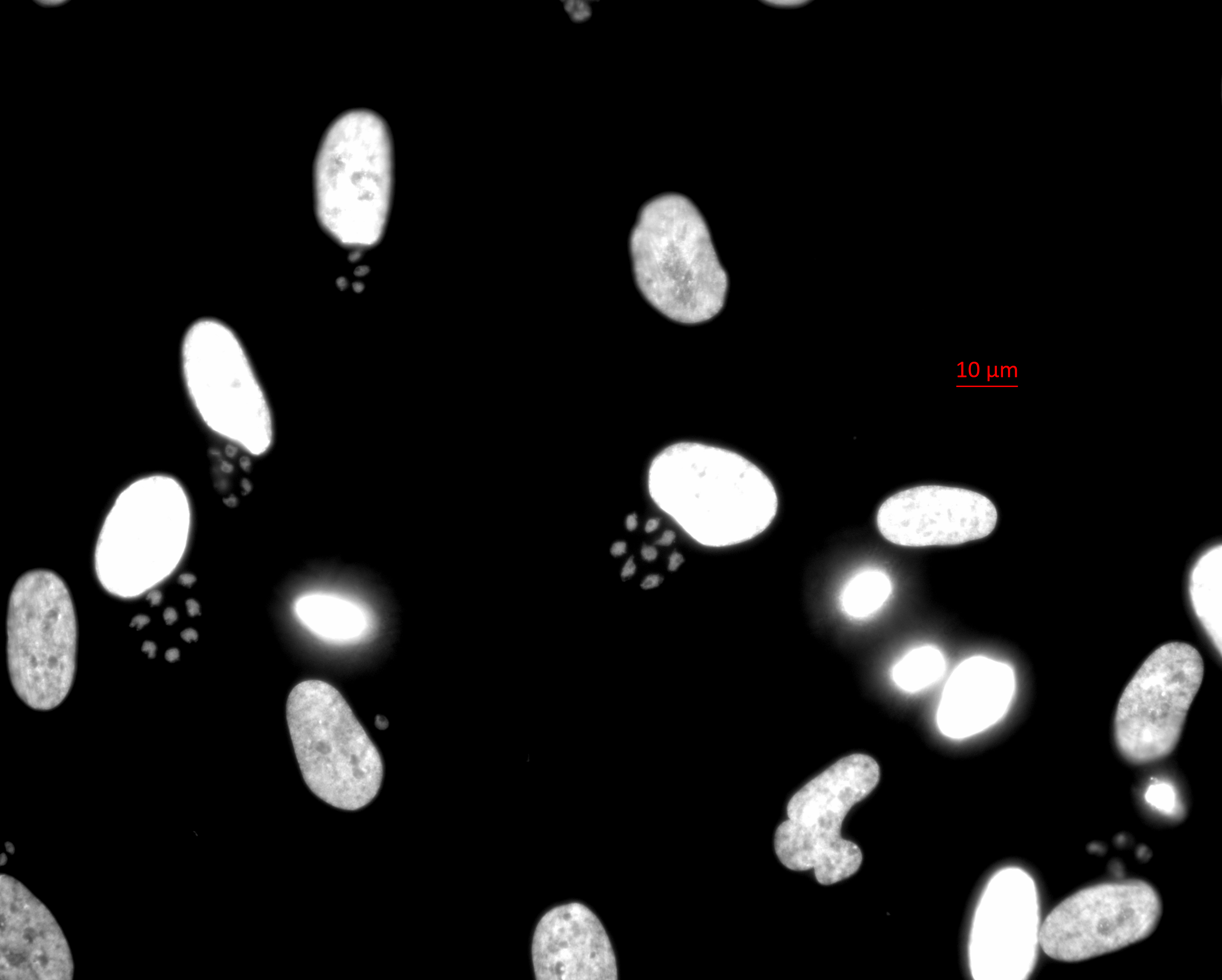

Supplement: Supplementary file 8 — Source data Fig. 2 [file 44321_2025_252_MOESM8_ESM.zip › Figure 2 Source Data/2a/image UT/Snap-3976_c2 (DNA).tif]

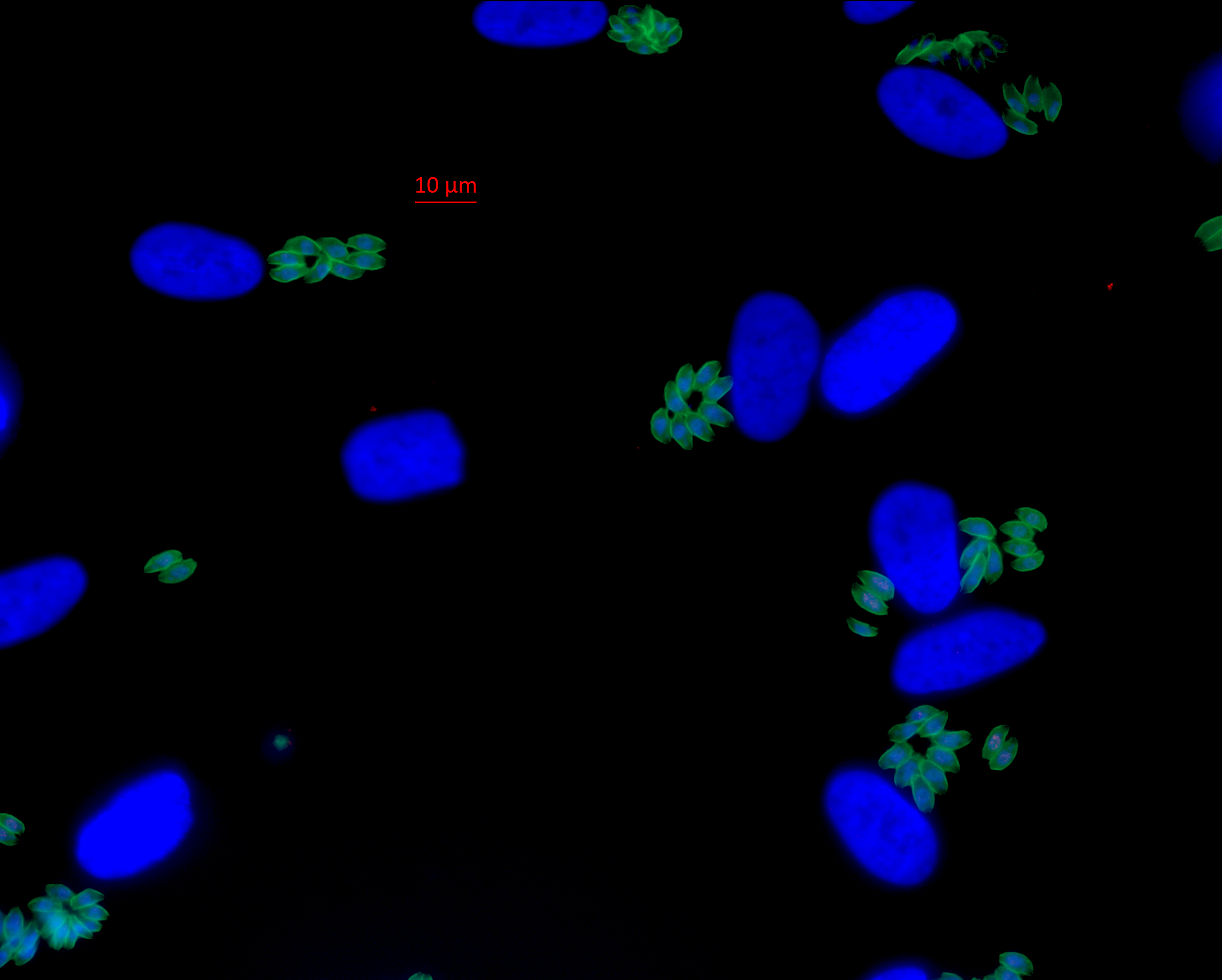

Supplement: Supplementary file 8 — Source data Fig. 2 [file 44321_2025_252_MOESM8_ESM.zip › Figure 2 Source Data/2e/RHku80 MORC KD BFD1-Flag /UT/Snap-3737_c2+3+4.tif]

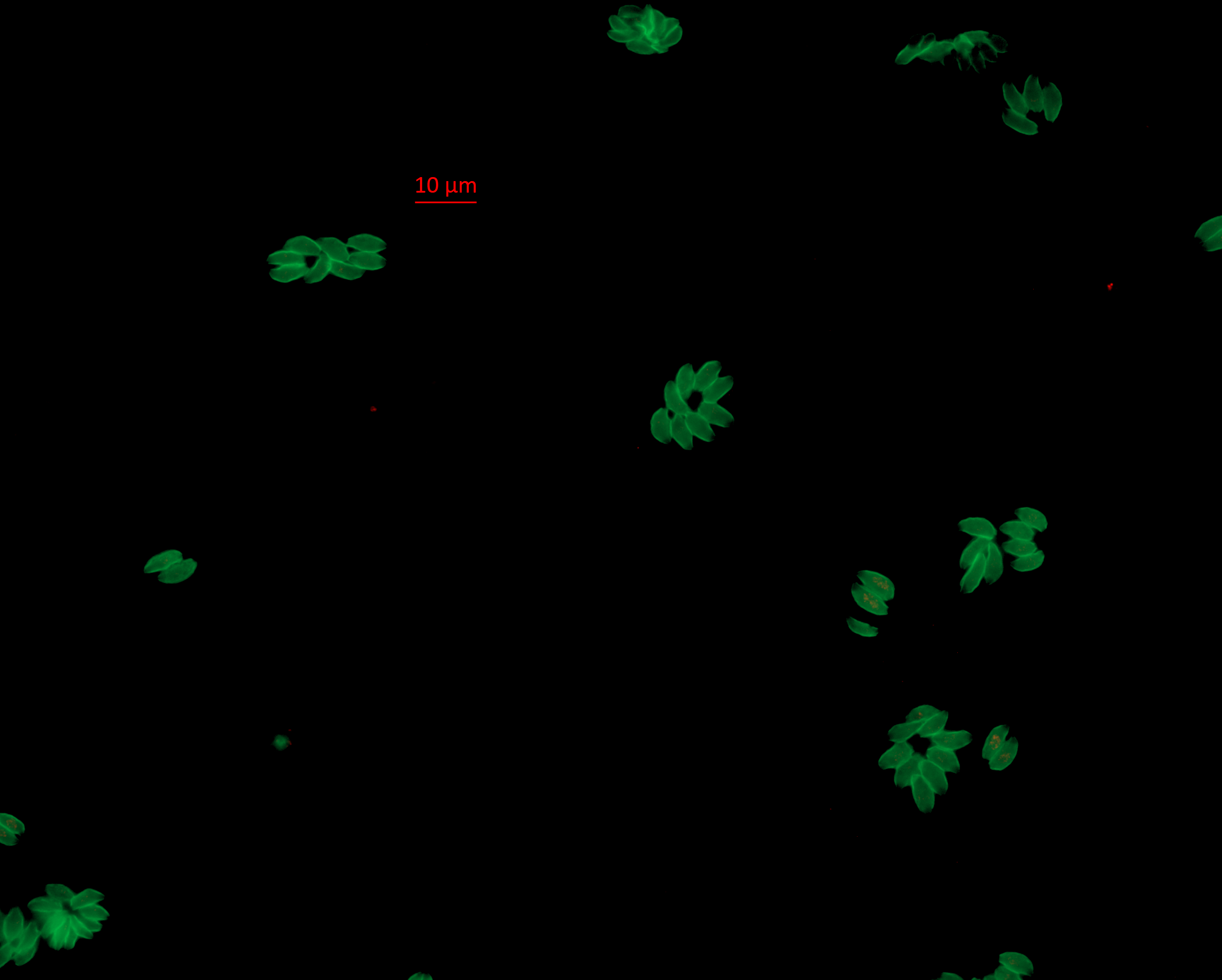

Supplement: Supplementary file 8 — Source data Fig. 2 [file 44321_2025_252_MOESM8_ESM.zip › Figure 2 Source Data/2e/RHku80 MORC KD BFD1-Flag /UT/Snap-3737_c3+4.tif]

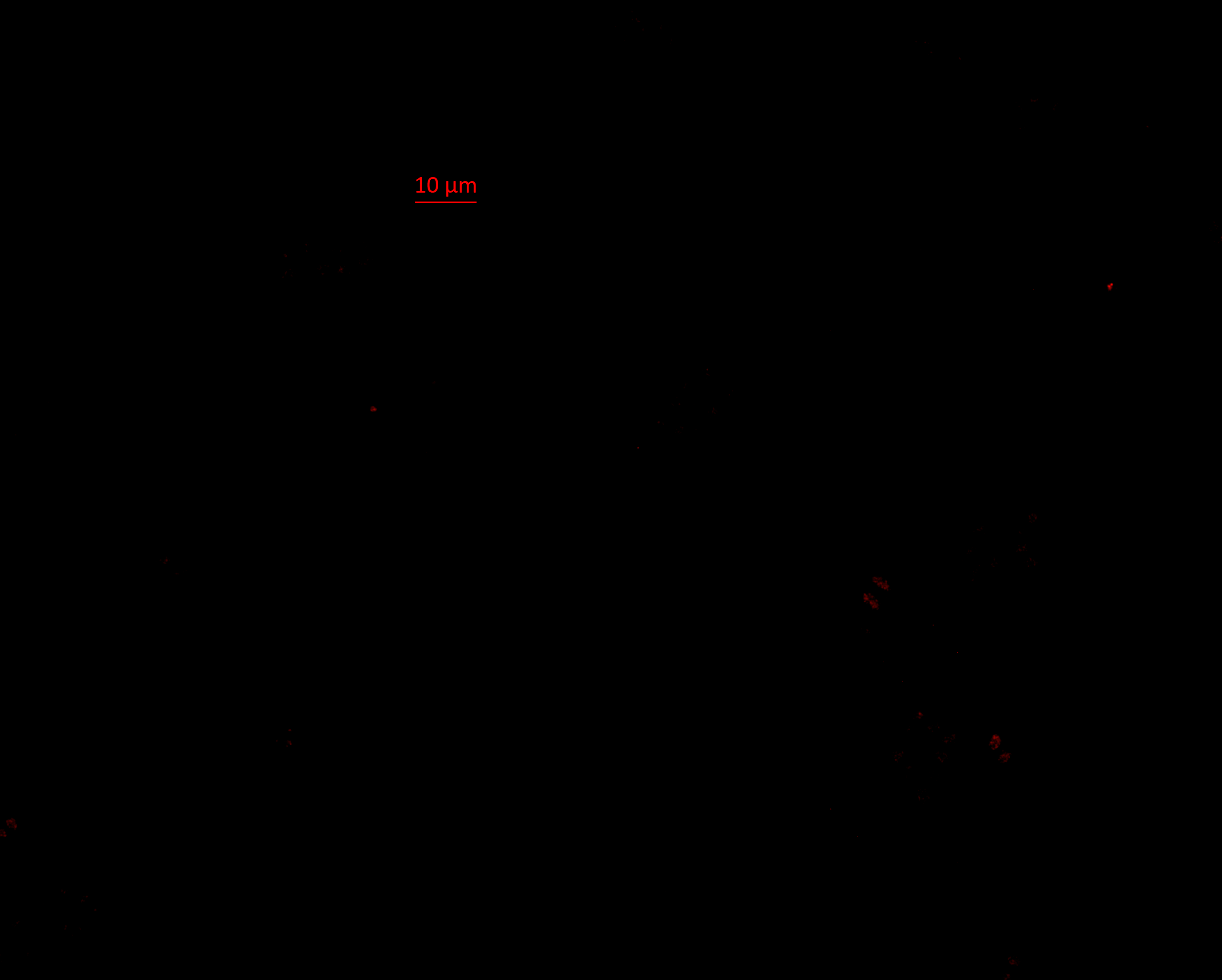

Supplement: Supplementary file 8 — Source data Fig. 2 [file 44321_2025_252_MOESM8_ESM.zip › Figure 2 Source Data/2e/RHku80 MORC KD BFD1-Flag /UT/Snap-3737_c4 (FLAG in red).tif]

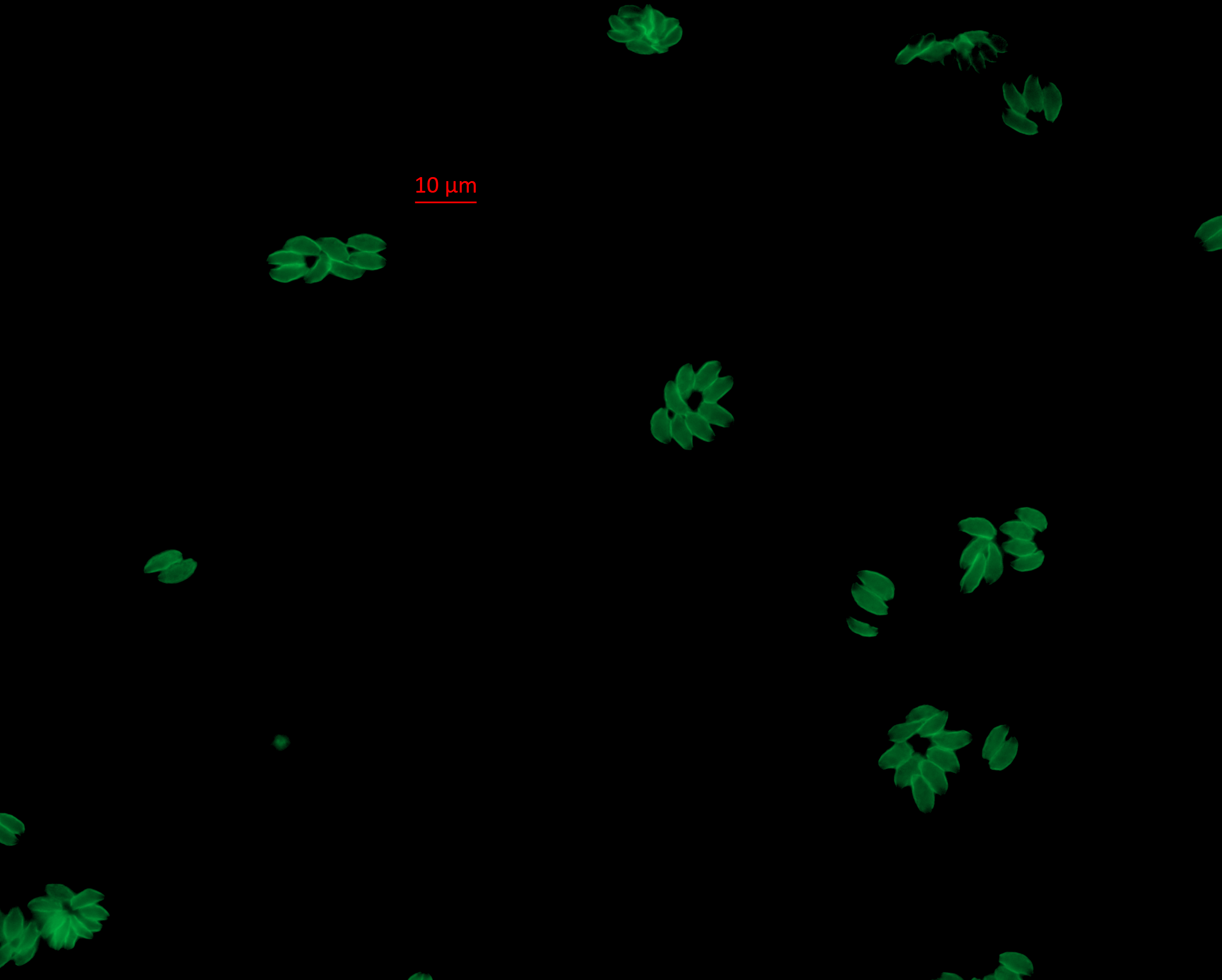

Supplement: Supplementary file 8 — Source data Fig. 2 [file 44321_2025_252_MOESM8_ESM.zip › Figure 2 Source Data/2e/RHku80 MORC KD BFD1-Flag /UT/Snap-3737_c3 (GAP45 in green).tif]

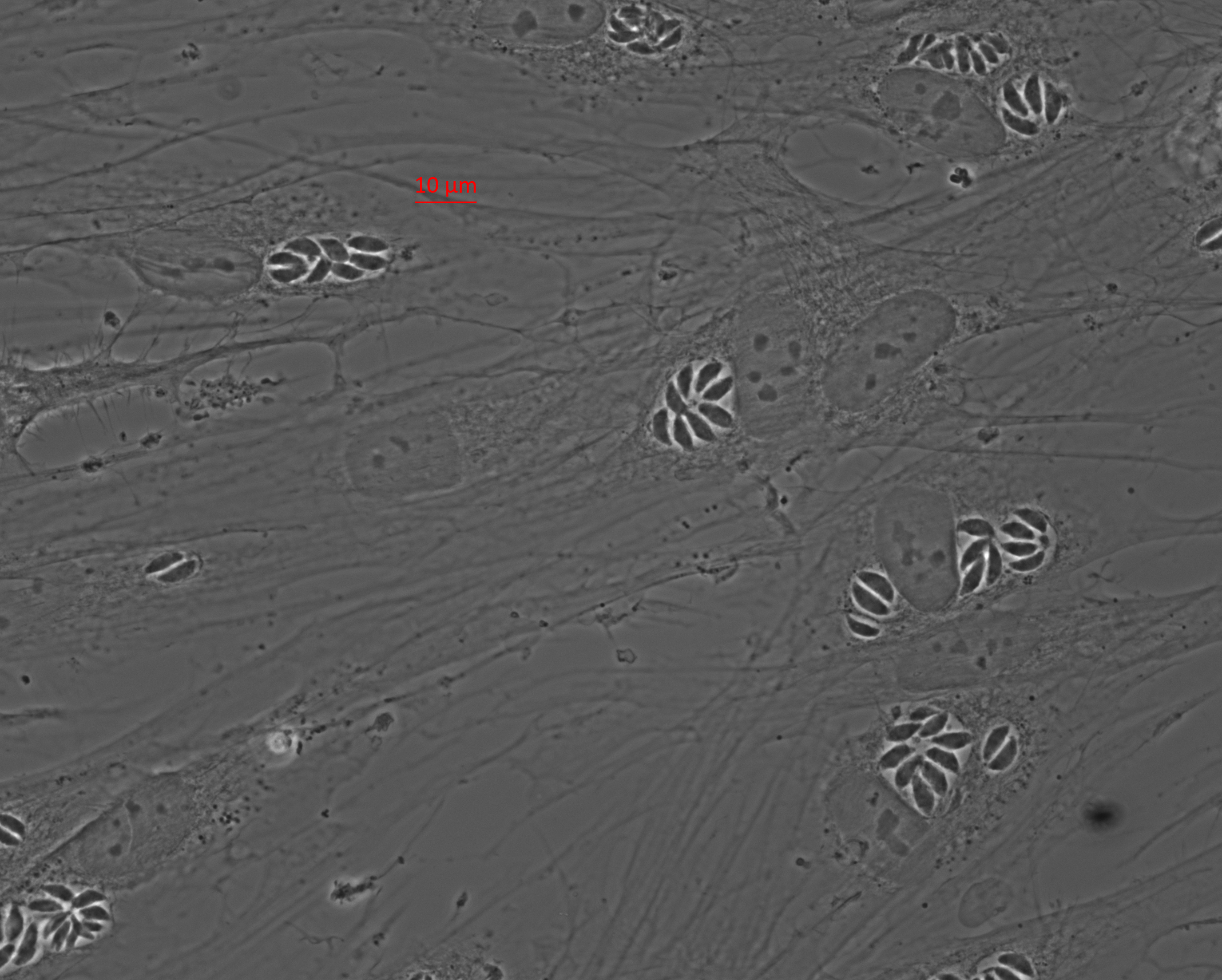

Supplement: Supplementary file 8 — Source data Fig. 2 [file 44321_2025_252_MOESM8_ESM.zip › Figure 2 Source Data/2e/RHku80 MORC KD BFD1-Flag /UT/Snap-3737_c1 (Phase).tif]

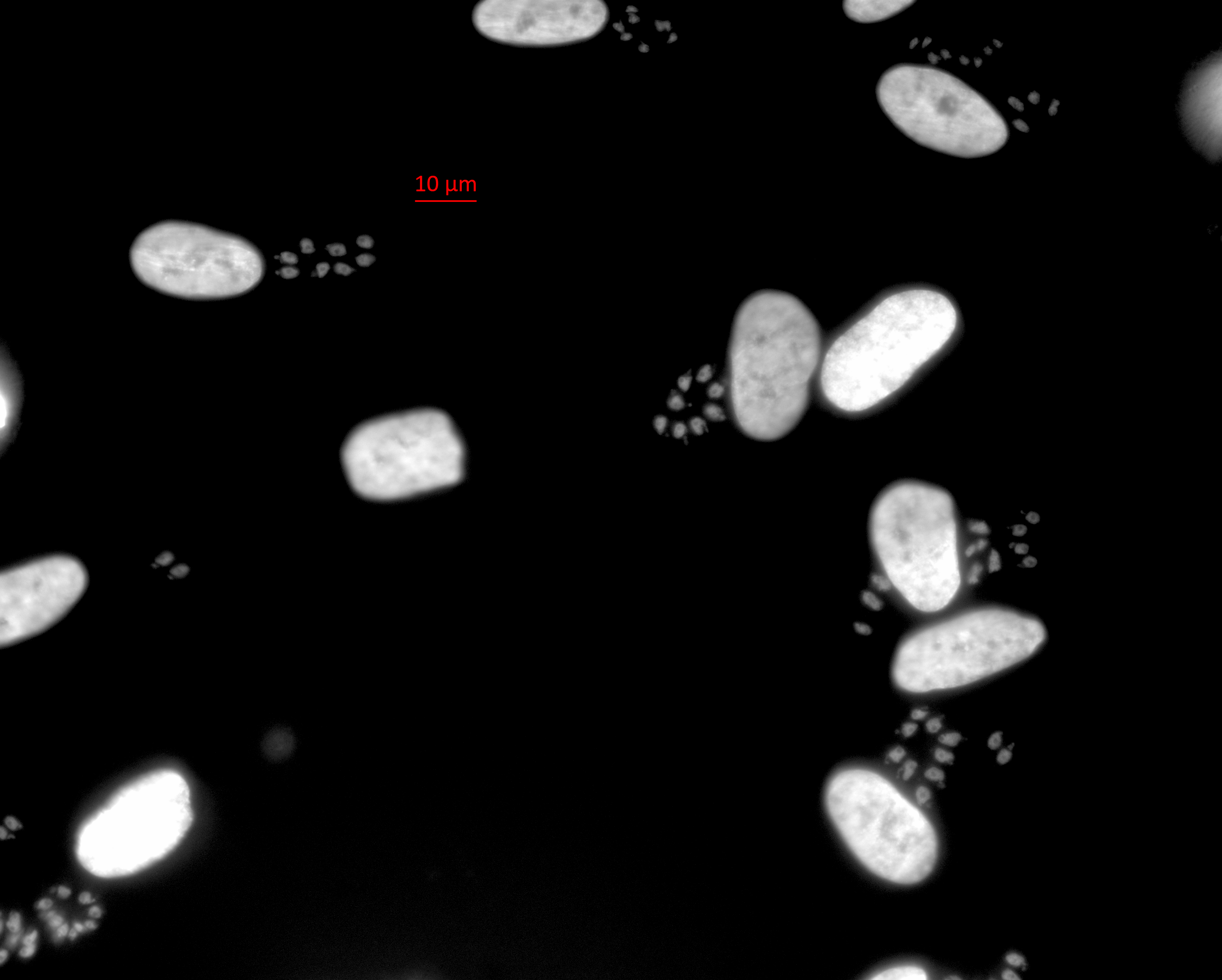

Supplement: Supplementary file 8 — Source data Fig. 2 [file 44321_2025_252_MOESM8_ESM.zip › Figure 2 Source Data/2e/RHku80 MORC KD BFD1-Flag /UT/Snap-3737_c2 (DNA).tif]

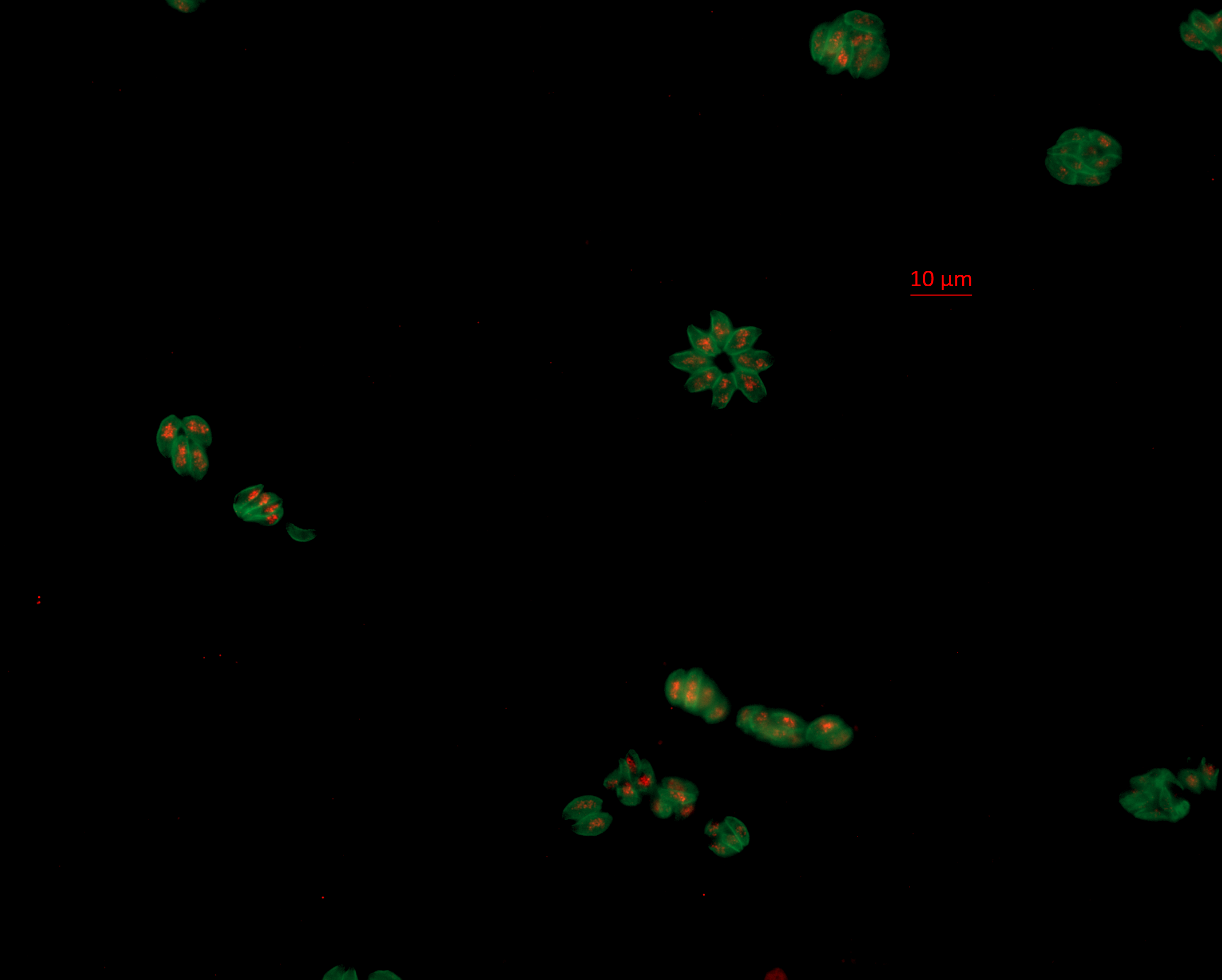

Supplement: Supplementary file 8 — Source data Fig. 2 [file 44321_2025_252_MOESM8_ESM.zip › Figure 2 Source Data/2e/RHku80 MORC KD BFD1-Flag /IAA (7h)/Snap-3730_c3+4.tif]

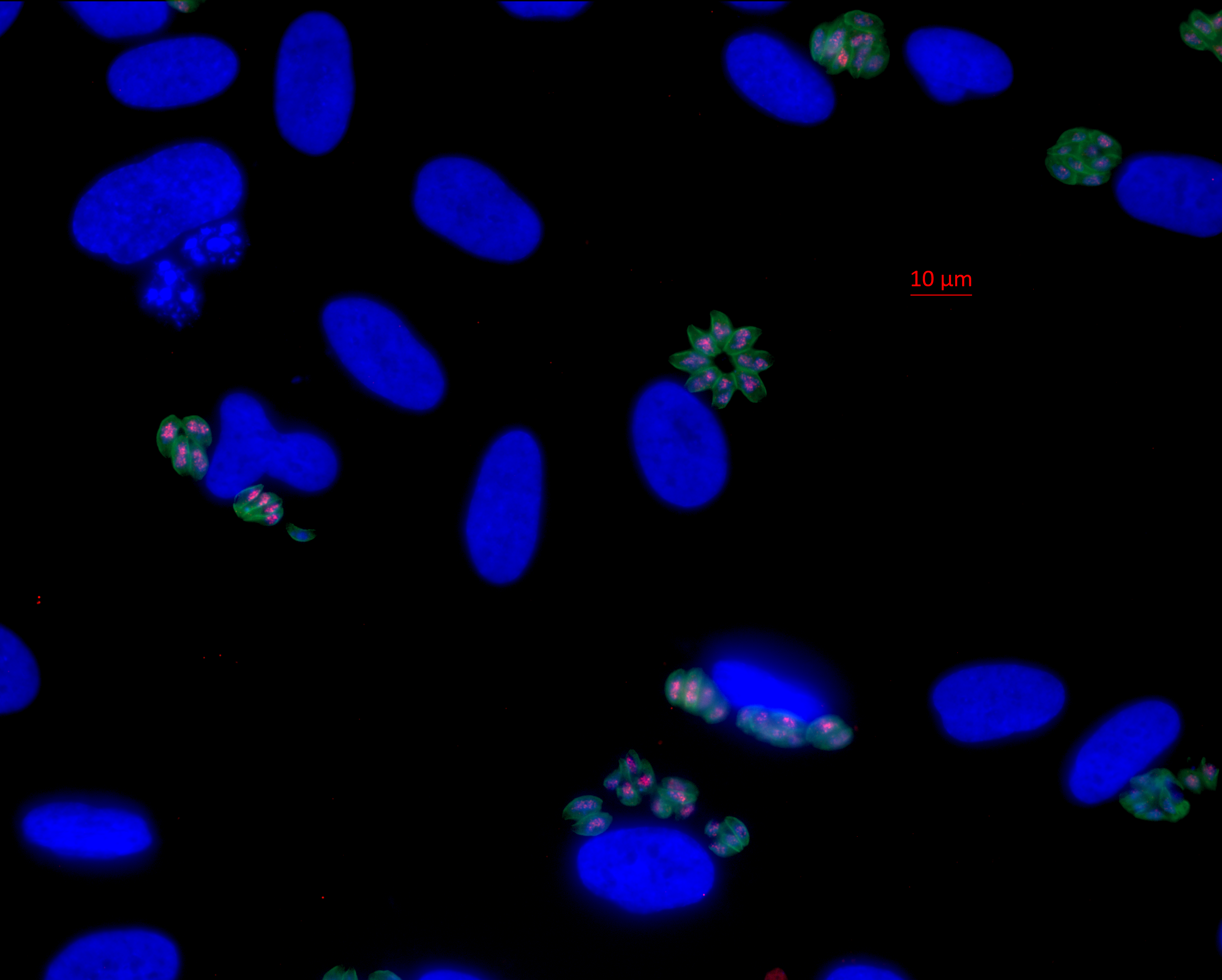

Supplement: Supplementary file 8 — Source data Fig. 2 [file 44321_2025_252_MOESM8_ESM.zip › Figure 2 Source Data/2e/RHku80 MORC KD BFD1-Flag /IAA (7h)/Snap-3730_c2+3+4.tif]

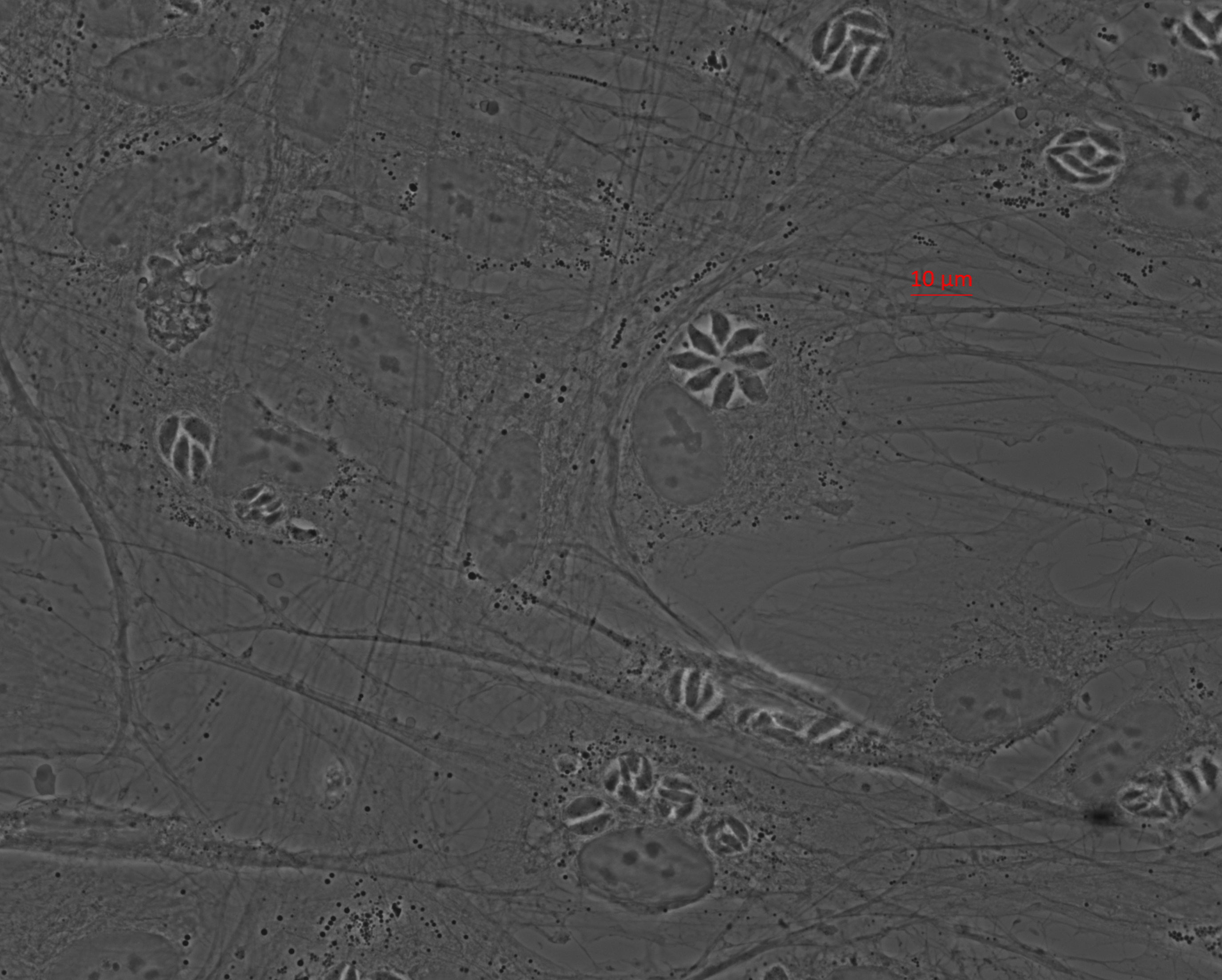

Supplement: Supplementary file 8 — Source data Fig. 2 [file 44321_2025_252_MOESM8_ESM.zip › Figure 2 Source Data/2e/RHku80 MORC KD BFD1-Flag /IAA (7h)/Snap-3730_c1 (Phase).tif]

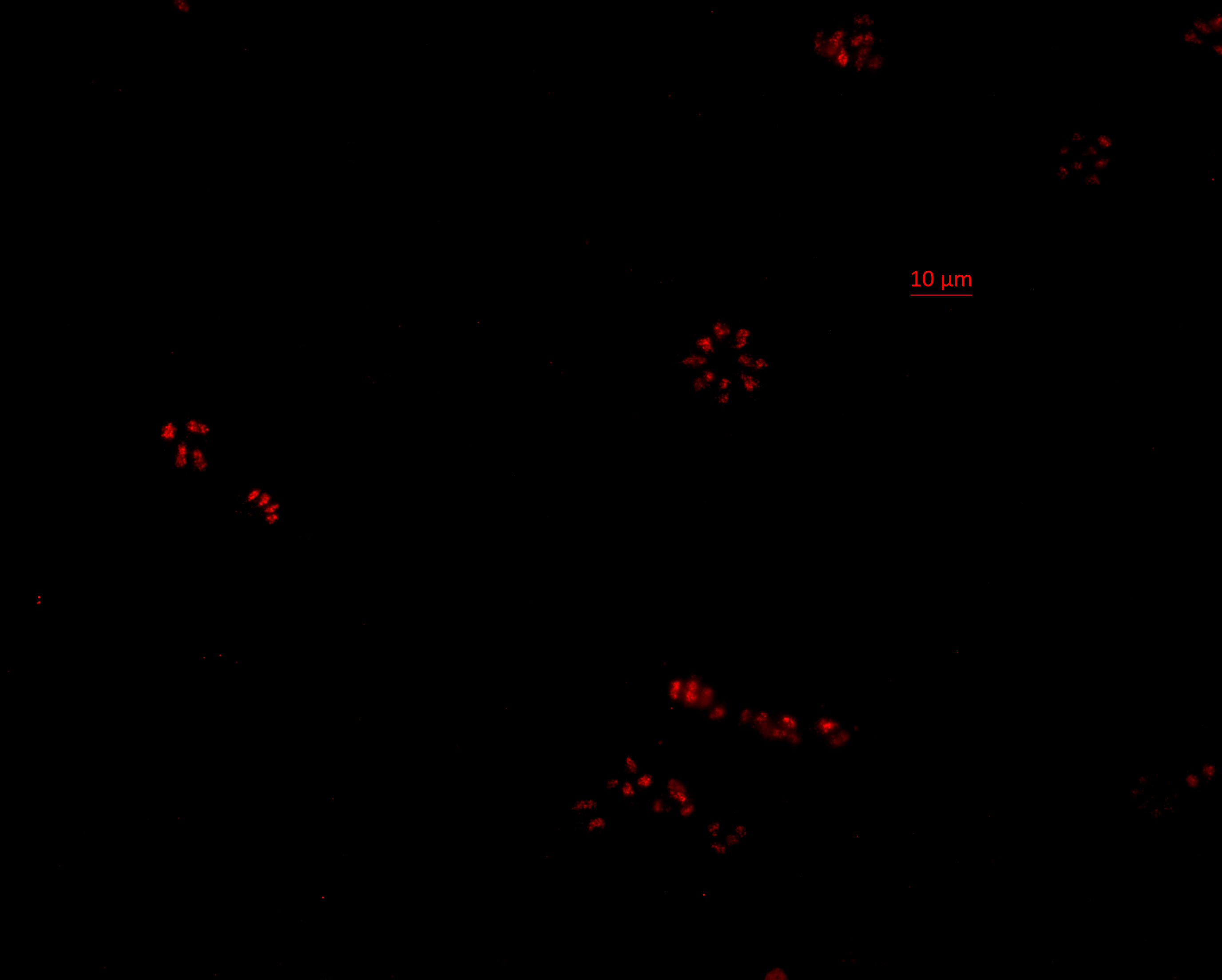

Supplement: Supplementary file 8 — Source data Fig. 2 [file 44321_2025_252_MOESM8_ESM.zip › Figure 2 Source Data/2e/RHku80 MORC KD BFD1-Flag /IAA (7h)/Snap-3730_c4 (FLAG in red).tif]

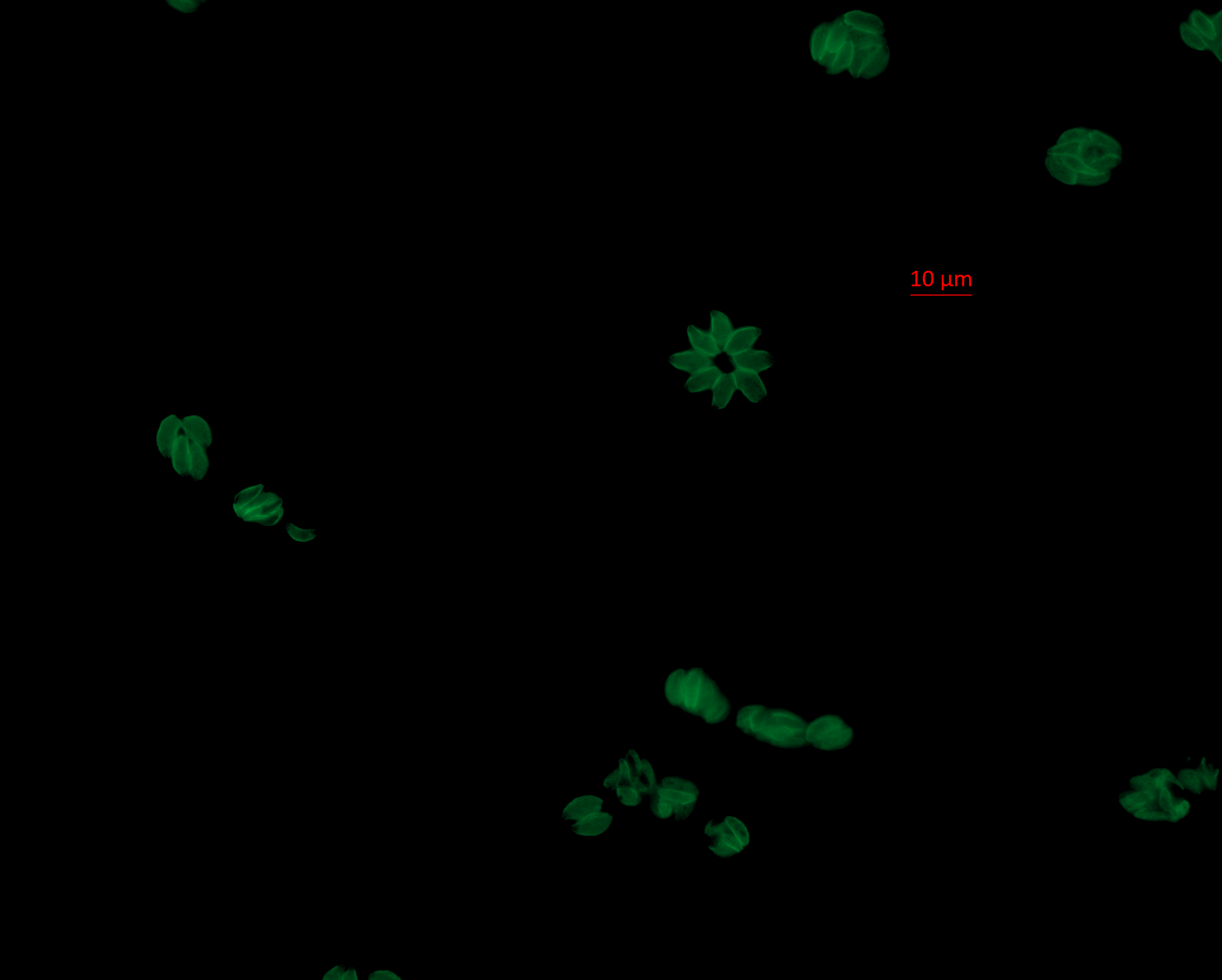

Supplement: Supplementary file 8 — Source data Fig. 2 [file 44321_2025_252_MOESM8_ESM.zip › Figure 2 Source Data/2e/RHku80 MORC KD BFD1-Flag /IAA (7h)/Snap-3730_c3 (GAP45 in green).tif]

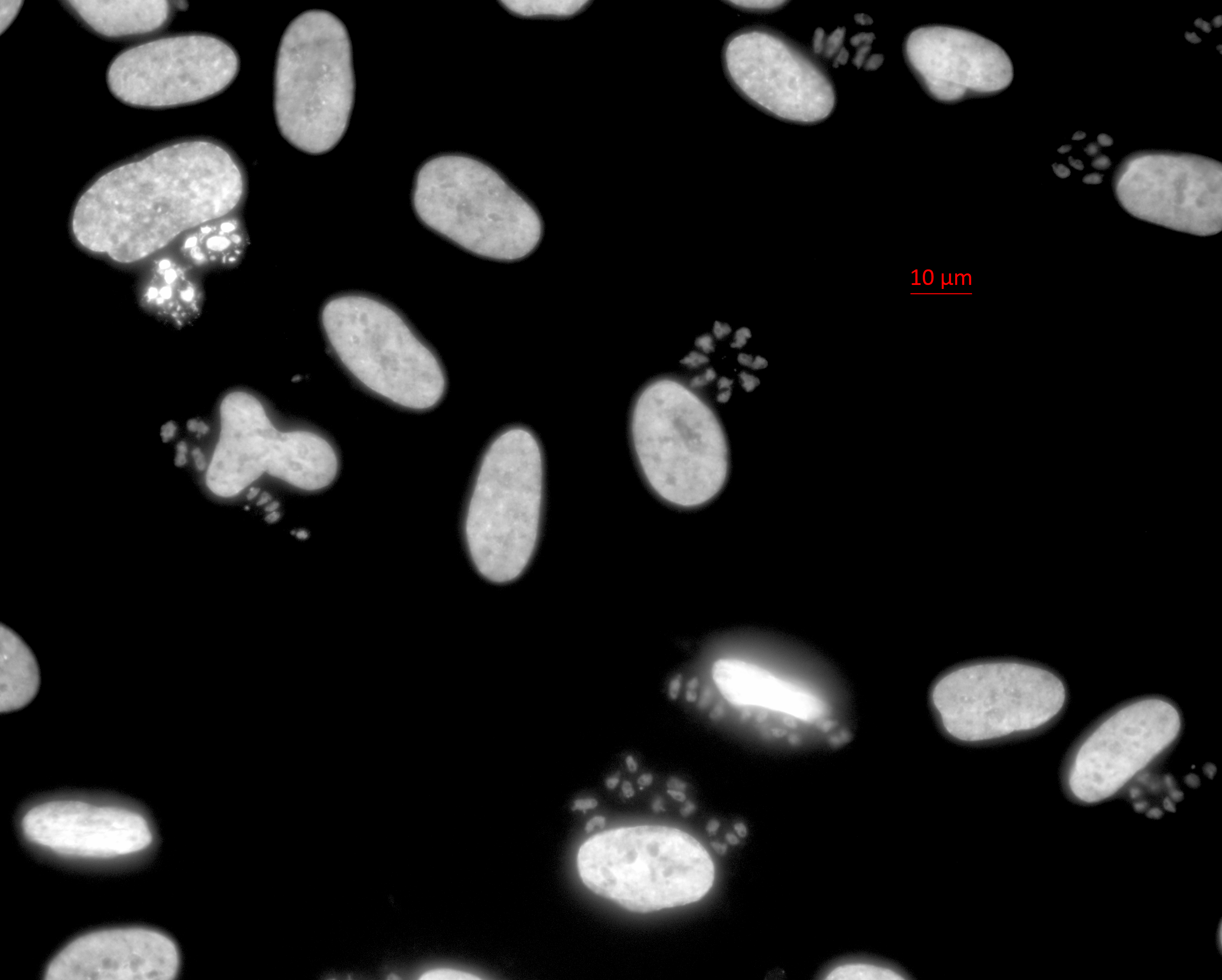

Supplement: Supplementary file 8 — Source data Fig. 2 [file 44321_2025_252_MOESM8_ESM.zip › Figure 2 Source Data/2e/RHku80 MORC KD BFD1-Flag /IAA (7h)/Snap-3730_c2 (DNA).tif]

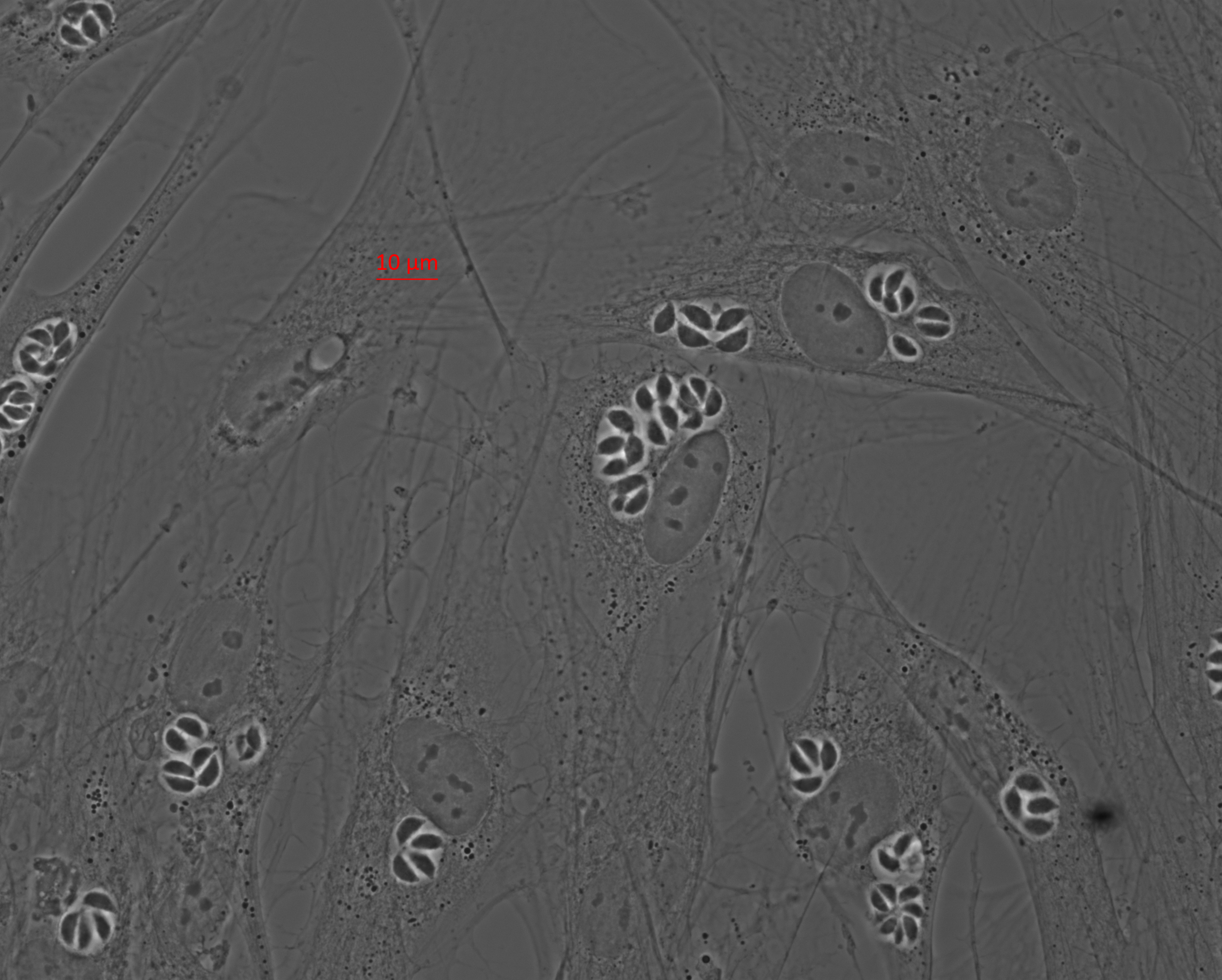

Supplement: Supplementary file 8 — Source data Fig. 2 [file 44321_2025_252_MOESM8_ESM.zip › Figure 2 Source Data/2e/Pruku80 BFD1-HAFLag/UT/Snap-3979_c1 (Phase).tif]

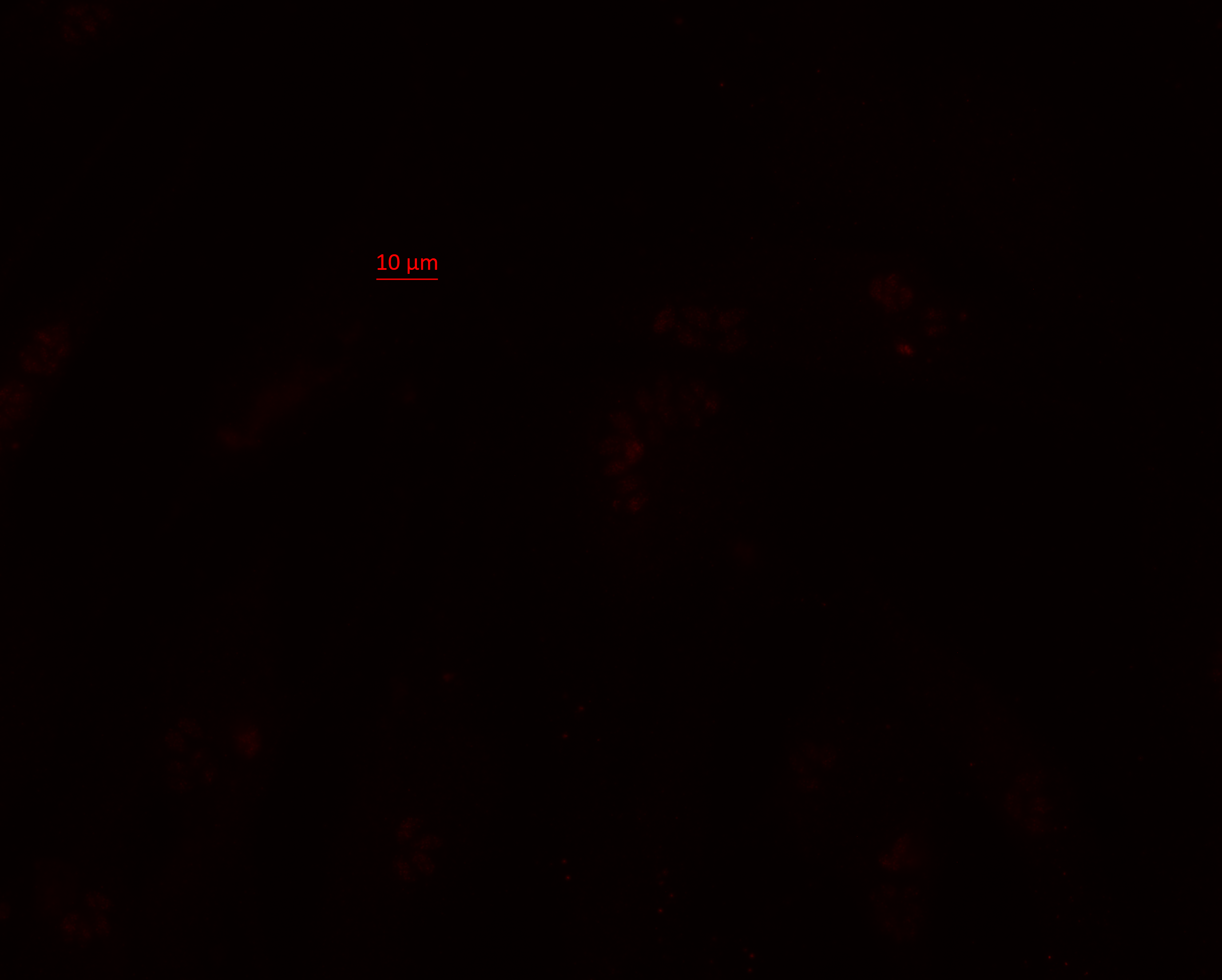

Supplement: Supplementary file 8 — Source data Fig. 2 [file 44321_2025_252_MOESM8_ESM.zip › Figure 2 Source Data/2e/Pruku80 BFD1-HAFLag/UT/Snap-3979_c4 (FLAG in red).tif]

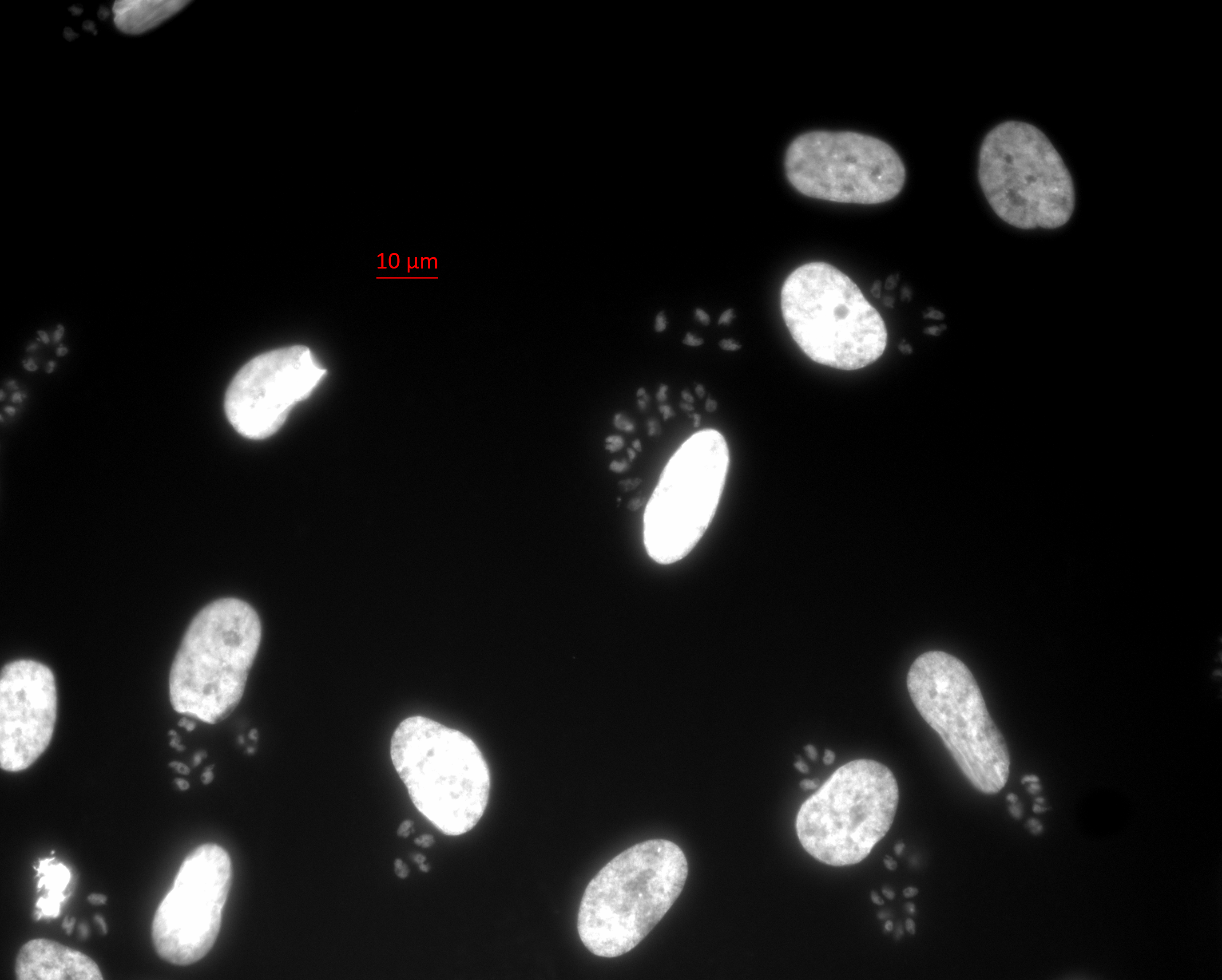

Supplement: Supplementary file 8 — Source data Fig. 2 [file 44321_2025_252_MOESM8_ESM.zip › Figure 2 Source Data/2e/Pruku80 BFD1-HAFLag/UT/Snap-3979_c2 (DNA).tif]

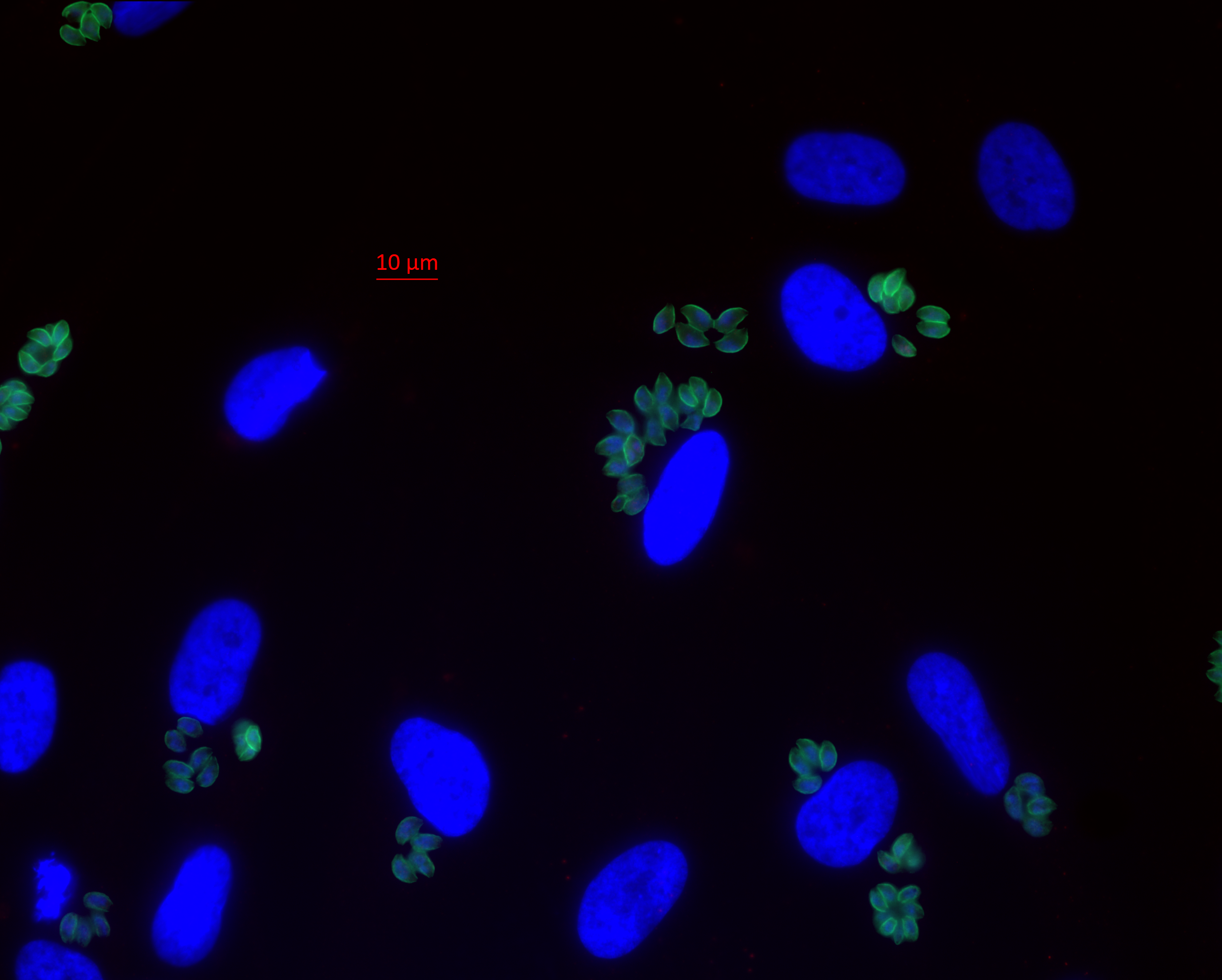

Supplement: Supplementary file 8 — Source data Fig. 2 [file 44321_2025_252_MOESM8_ESM.zip › Figure 2 Source Data/2e/Pruku80 BFD1-HAFLag/UT/Snap-3979_c2+3+4.tif]

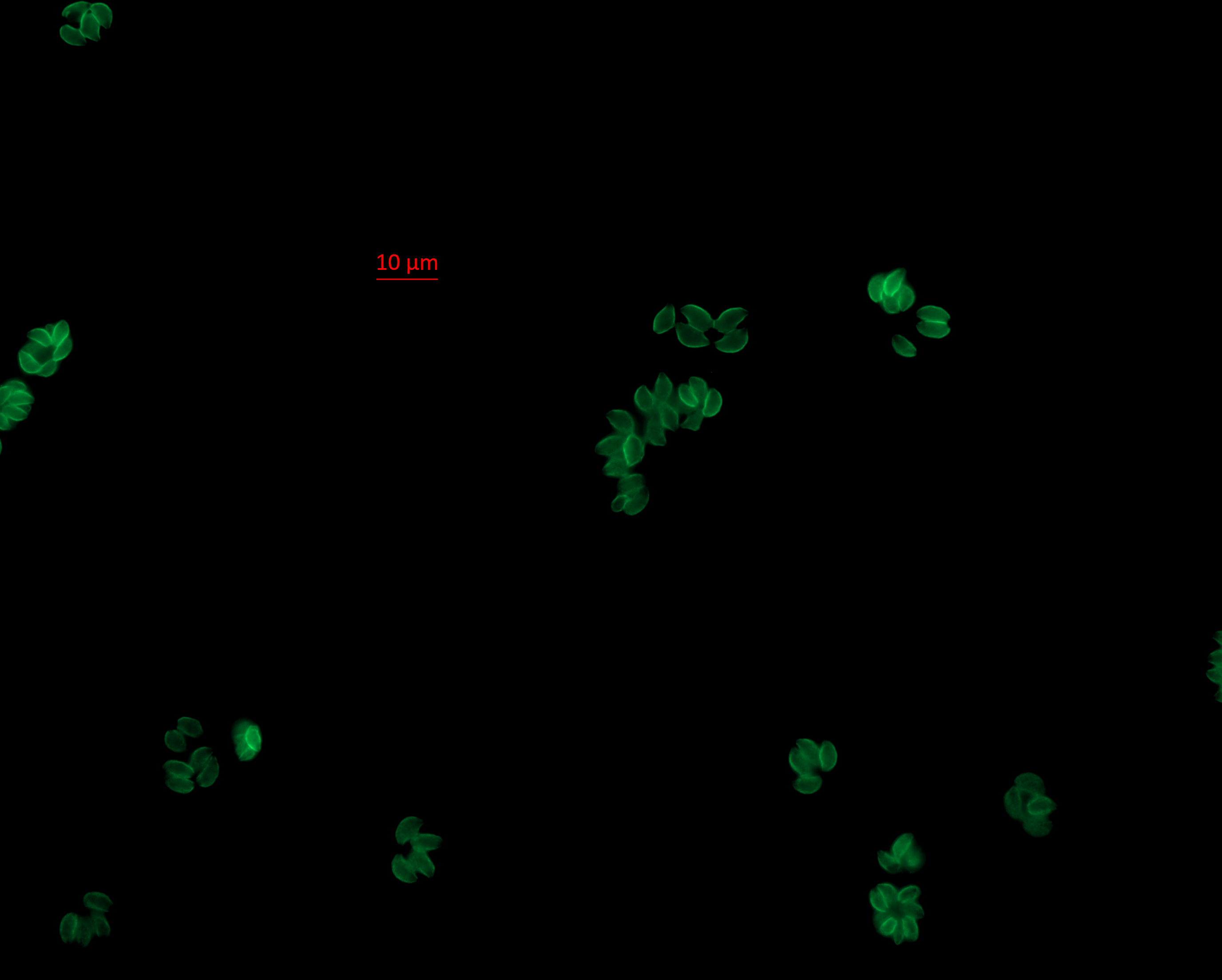

Supplement: Supplementary file 8 — Source data Fig. 2 [file 44321_2025_252_MOESM8_ESM.zip › Figure 2 Source Data/2e/Pruku80 BFD1-HAFLag/UT/Snap-3979_c3 (GAP45 in green).tif]

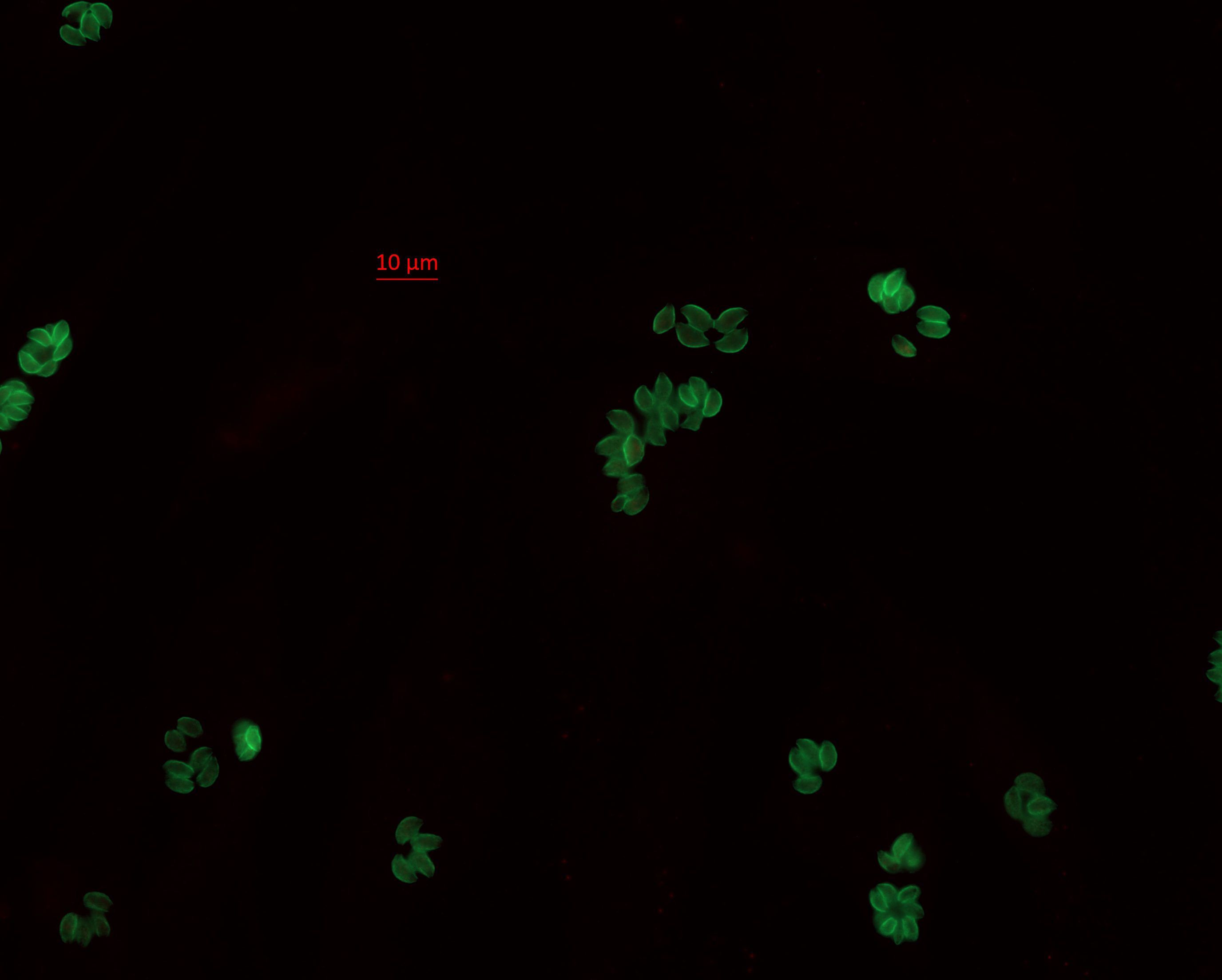

Supplement: Supplementary file 8 — Source data Fig. 2 [file 44321_2025_252_MOESM8_ESM.zip › Figure 2 Source Data/2e/Pruku80 BFD1-HAFLag/UT/Snap-3979_c3+4.tif]

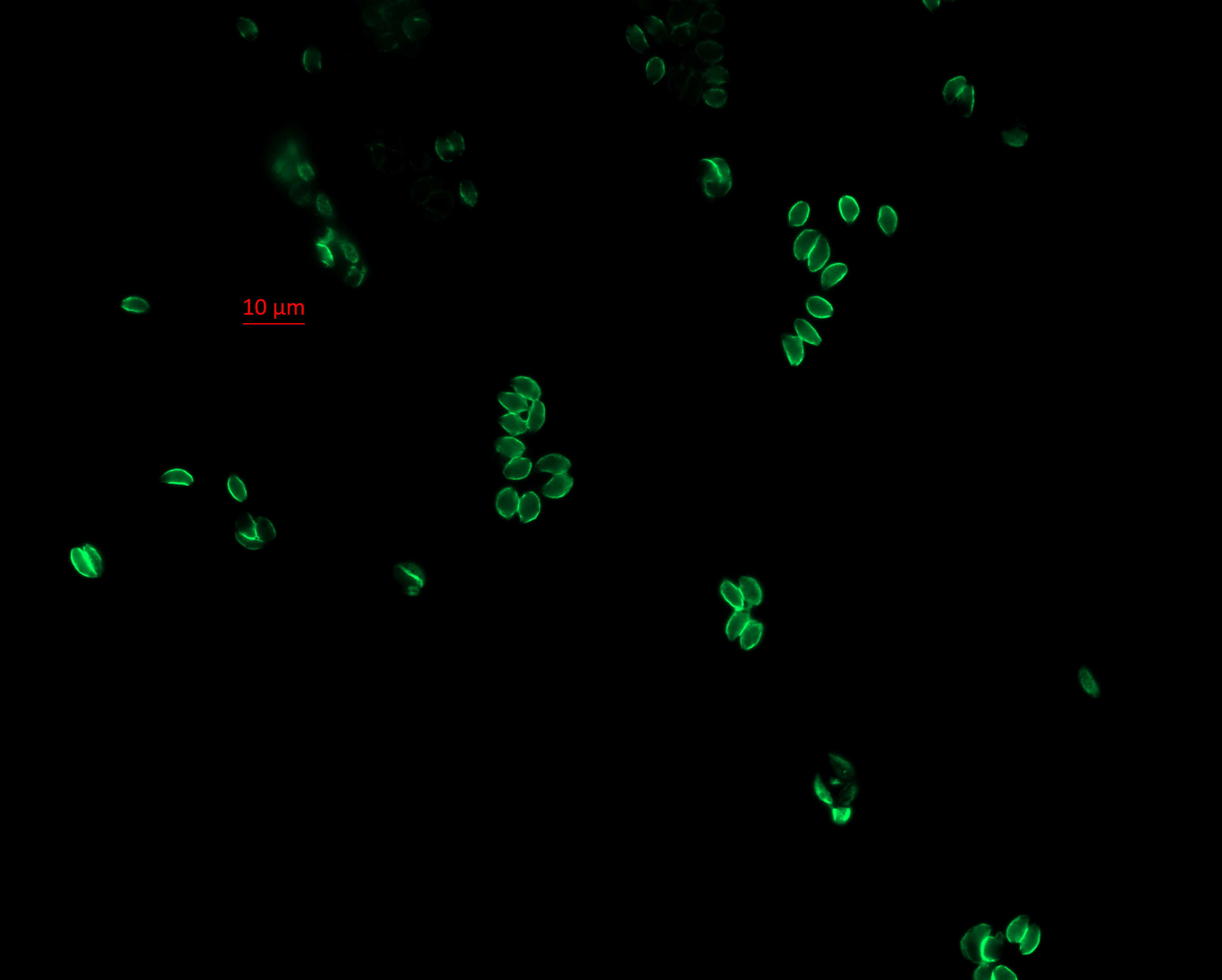

Supplement: Supplementary file 8 — Source data Fig. 2 [file 44321_2025_252_MOESM8_ESM.zip › Figure 2 Source Data/2e/Pruku80 BFD1-HAFLag/FR235222/Snap-3956_c3 (GAP45 in green).tif]

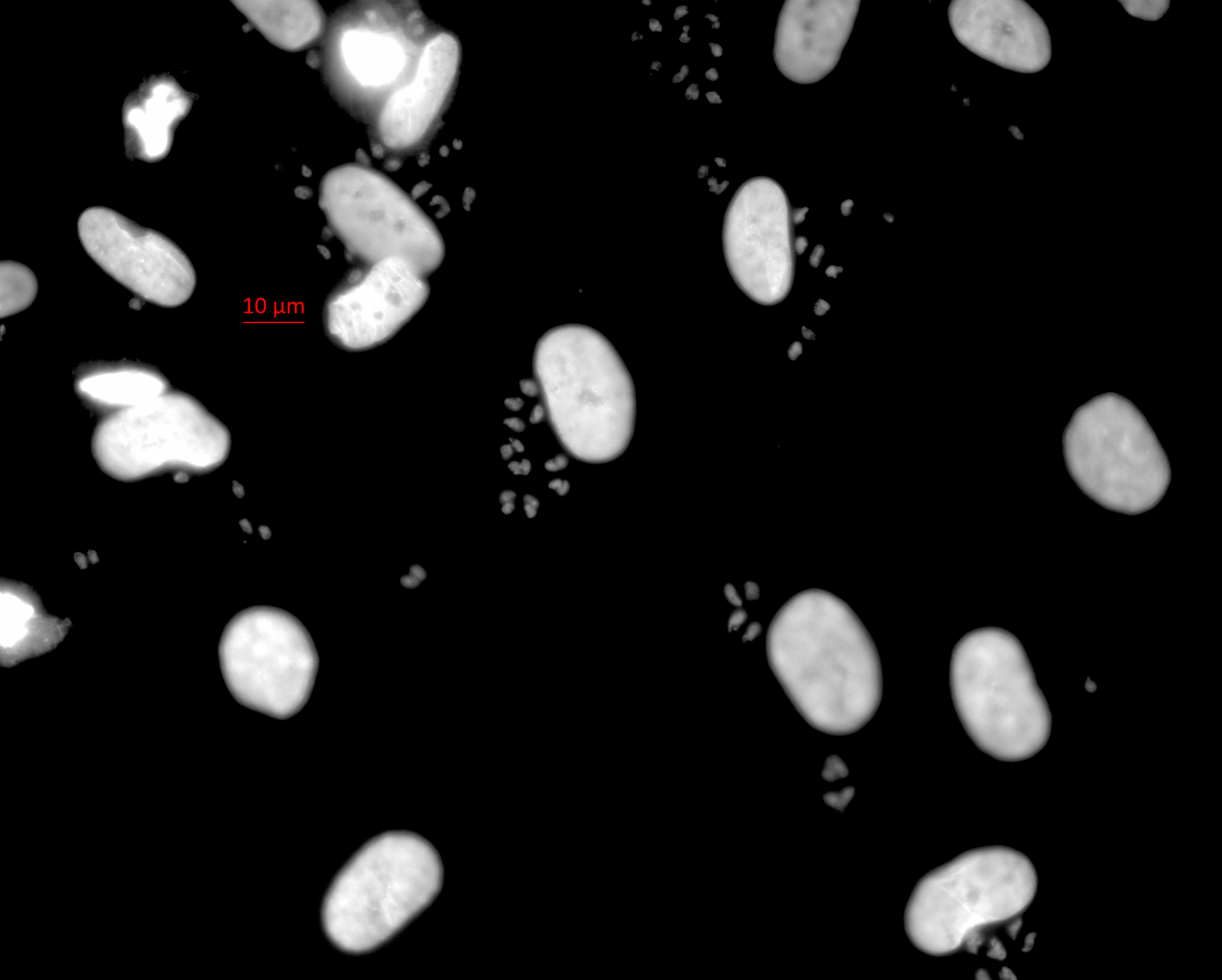

Supplement: Supplementary file 8 — Source data Fig. 2 [file 44321_2025_252_MOESM8_ESM.zip › Figure 2 Source Data/2e/Pruku80 BFD1-HAFLag/FR235222/Snap-3956_c2 (DNA).tif]

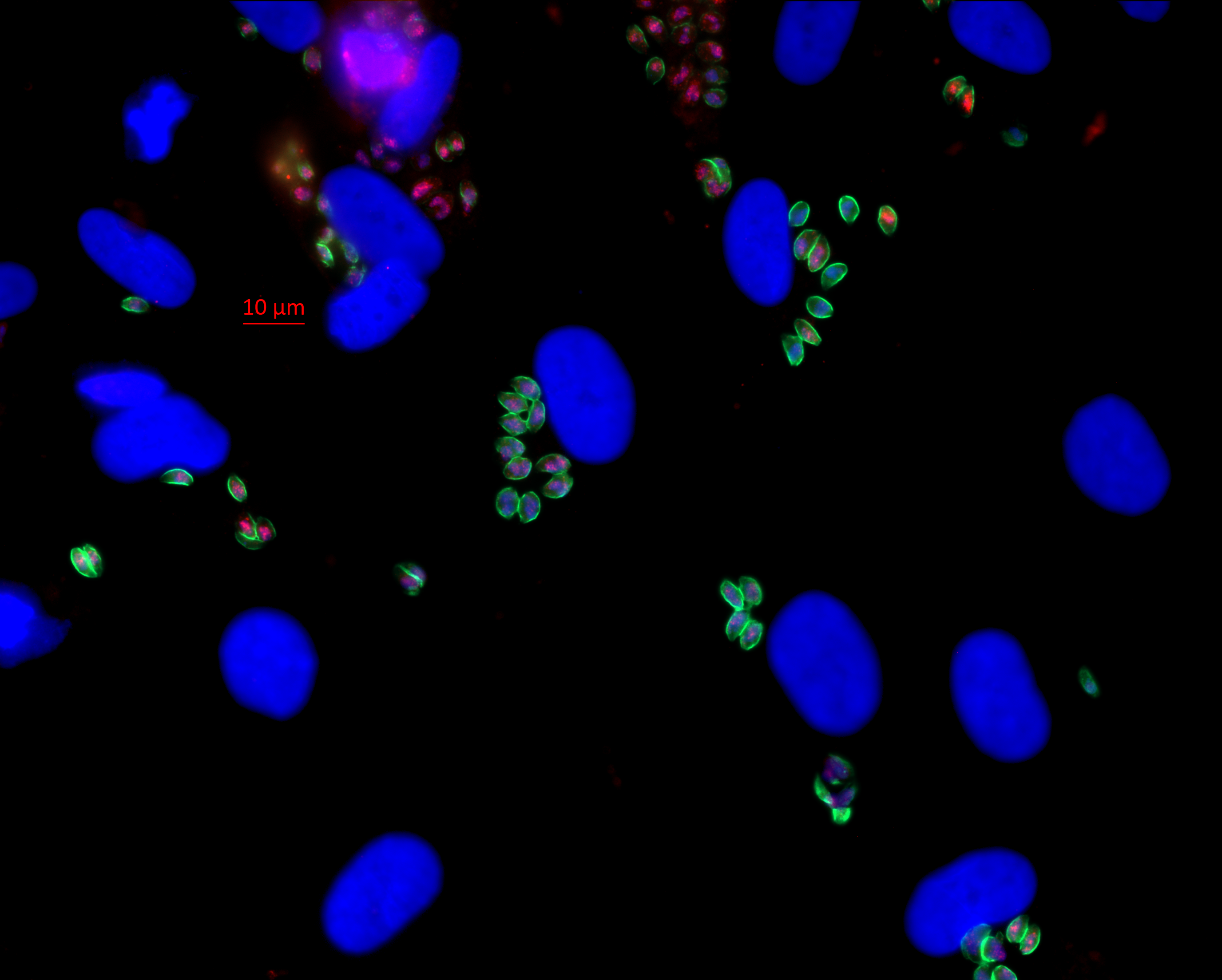

Supplement: Supplementary file 8 — Source data Fig. 2 [file 44321_2025_252_MOESM8_ESM.zip › Figure 2 Source Data/2e/Pruku80 BFD1-HAFLag/FR235222/Snap-3956_c2+3+4.tif]

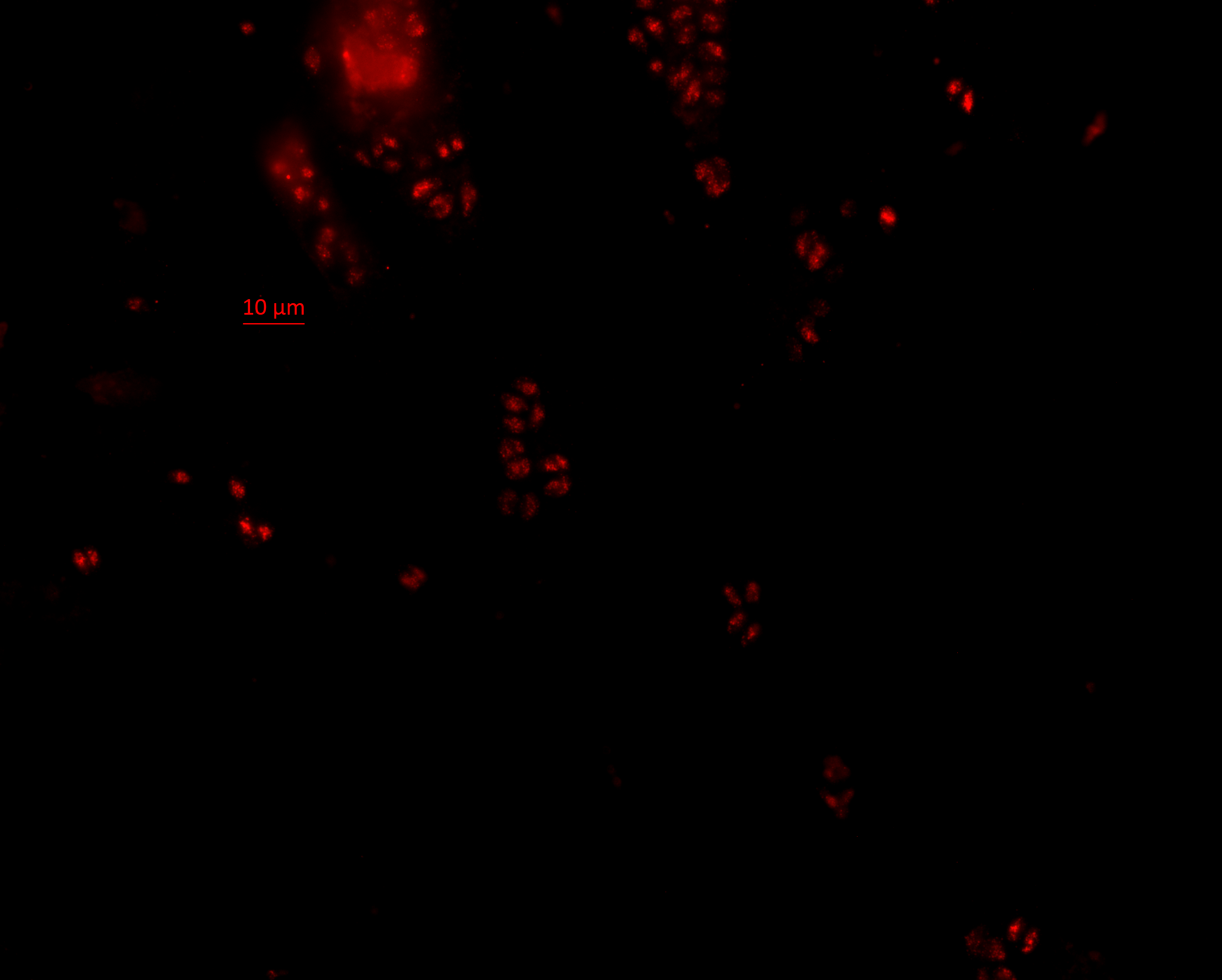

Supplement: Supplementary file 8 — Source data Fig. 2 [file 44321_2025_252_MOESM8_ESM.zip › Figure 2 Source Data/2e/Pruku80 BFD1-HAFLag/FR235222/Snap-3956_c4 (FLAG in red).tif]

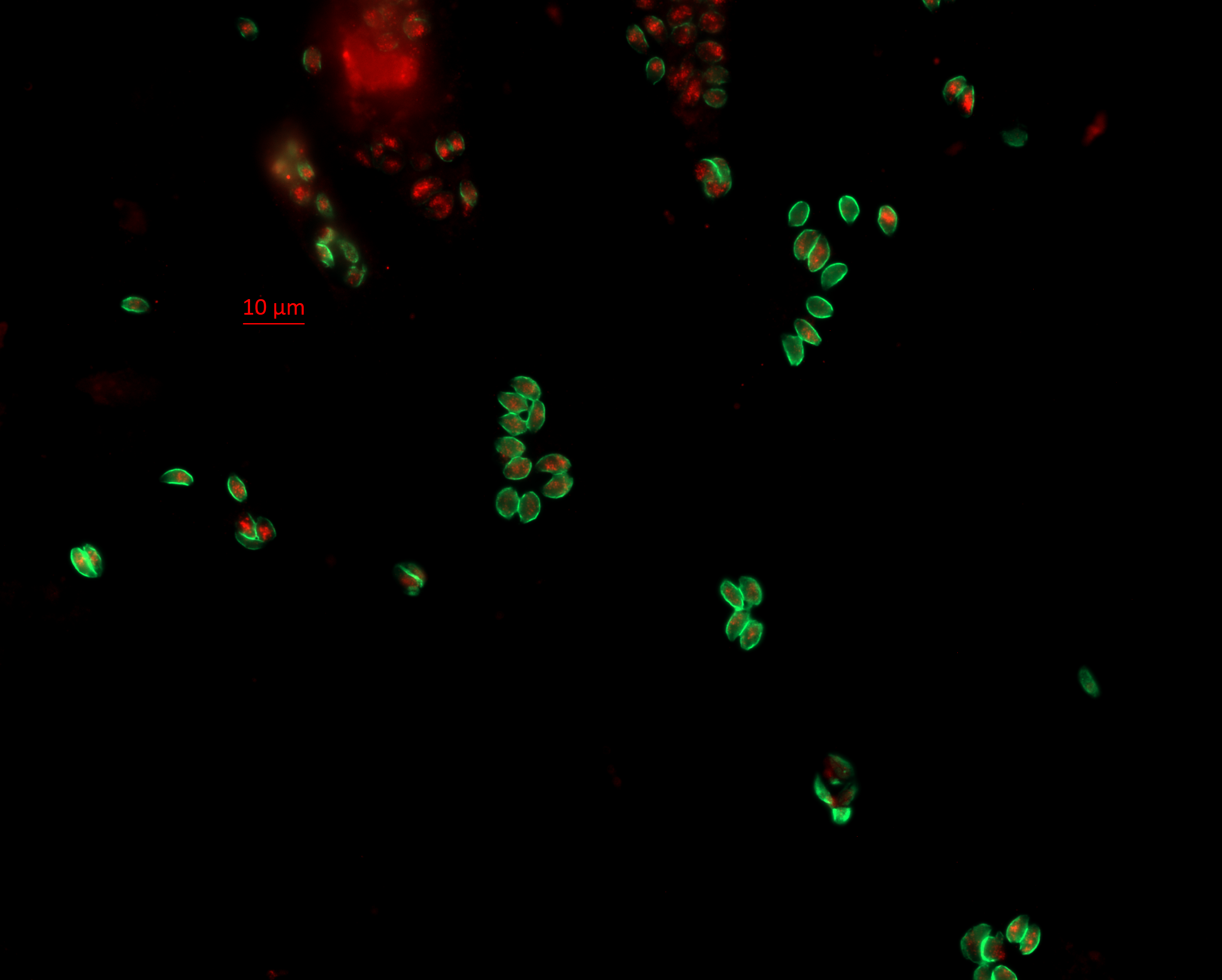

Supplement: Supplementary file 8 — Source data Fig. 2 [file 44321_2025_252_MOESM8_ESM.zip › Figure 2 Source Data/2e/Pruku80 BFD1-HAFLag/FR235222/Snap-3956_c3+4.tif]

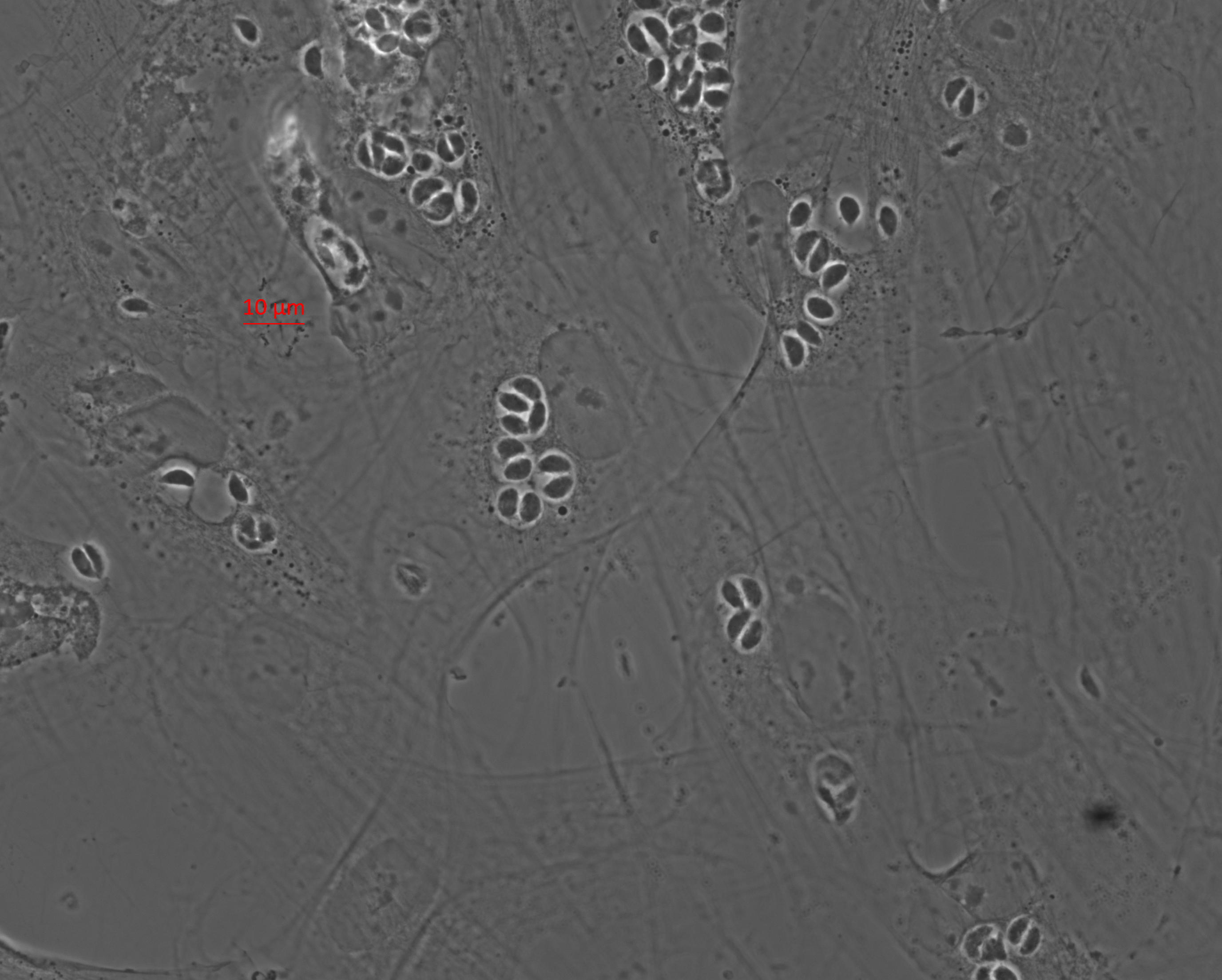

Supplement: Supplementary file 8 — Source data Fig. 2 [file 44321_2025_252_MOESM8_ESM.zip › Figure 2 Source Data/2e/Pruku80 BFD1-HAFLag/FR235222/Snap-3956_c1 (Phase).tif]

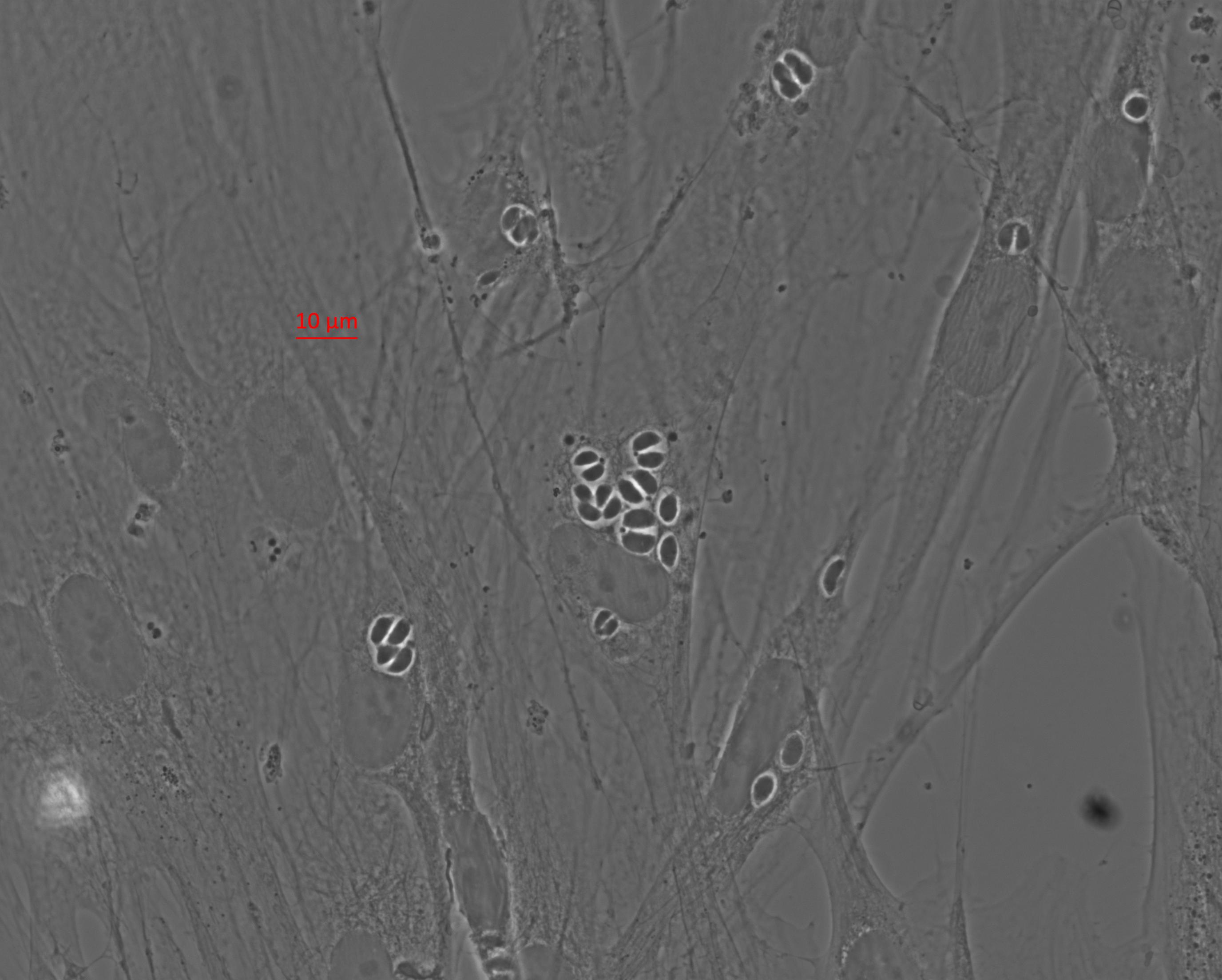

Supplement: Supplementary file 8 — Source data Fig. 2 [file 44321_2025_252_MOESM8_ESM.zip › Figure 2 Source Data/2b/BSM IFA (red) in 76K vs 76K BSM KO/76K BSM KO UT/Snap-4031_c1 (Phase).tif]

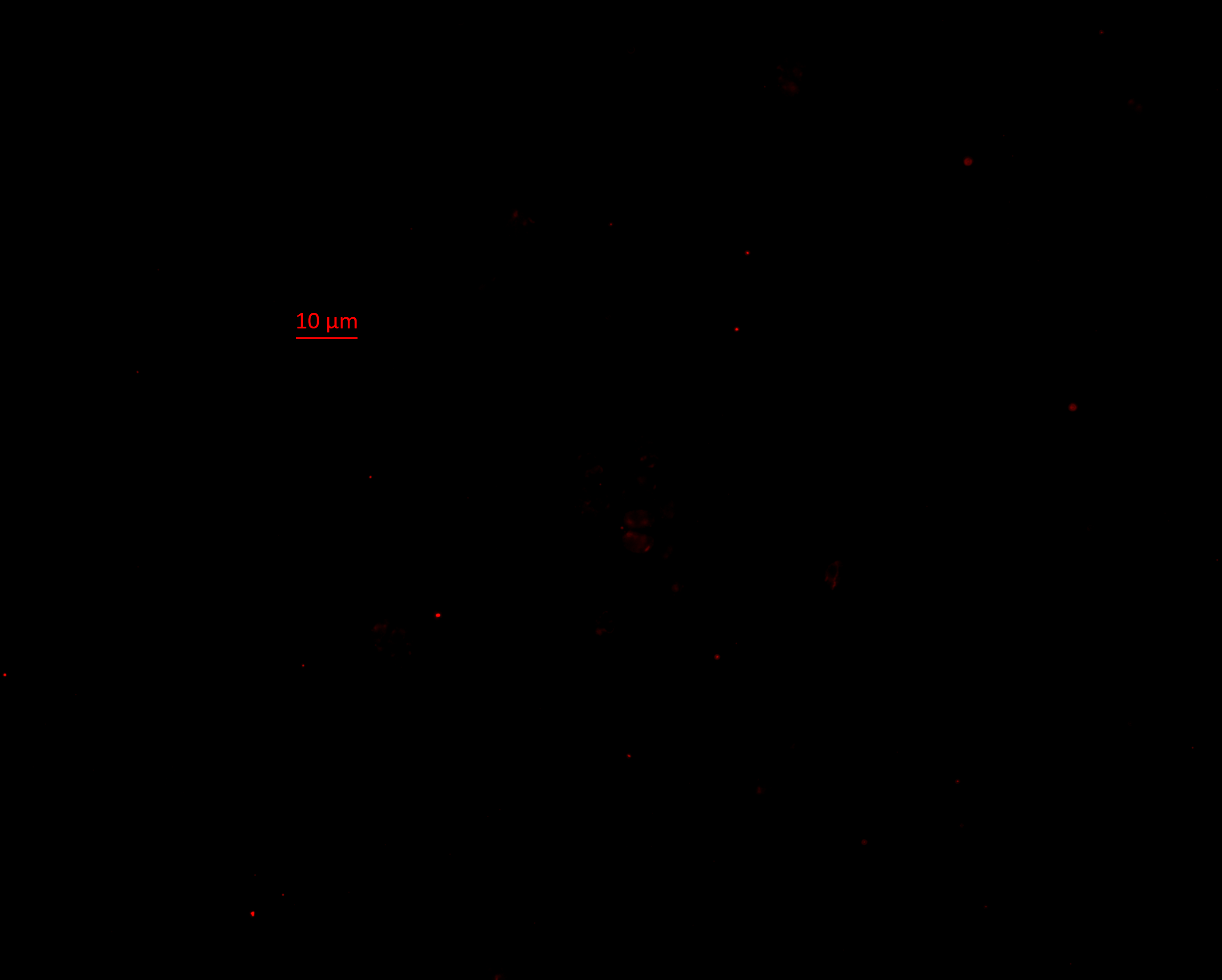

Supplement: Supplementary file 8 — Source data Fig. 2 [file 44321_2025_252_MOESM8_ESM.zip › Figure 2 Source Data/2b/BSM IFA (red) in 76K vs 76K BSM KO/76K BSM KO UT/Snap-4031_c3 (BSM).tif]

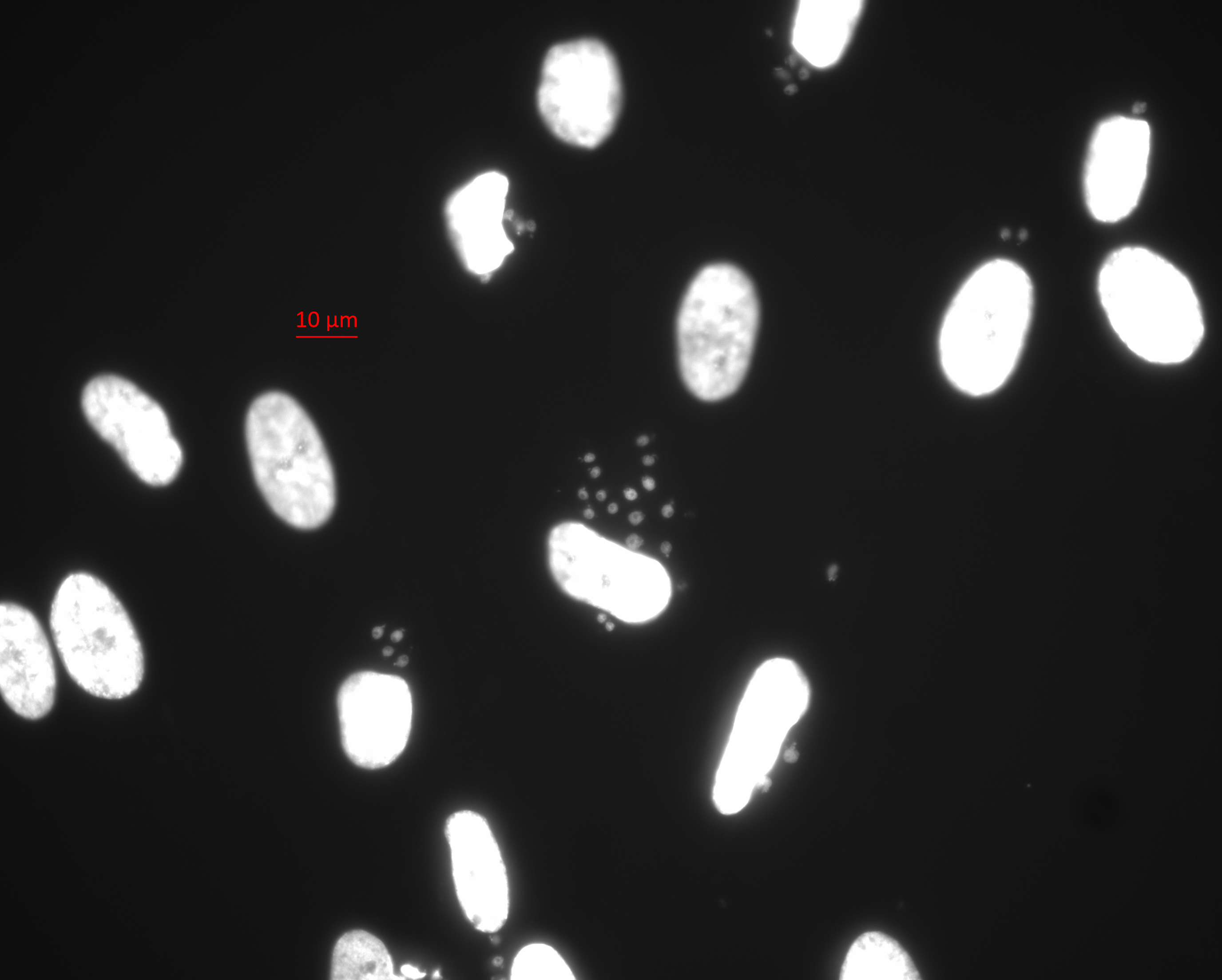

Supplement: Supplementary file 8 — Source data Fig. 2 [file 44321_2025_252_MOESM8_ESM.zip › Figure 2 Source Data/2b/BSM IFA (red) in 76K vs 76K BSM KO/76K BSM KO UT/Snap-4031_c2 (DNA).tif]

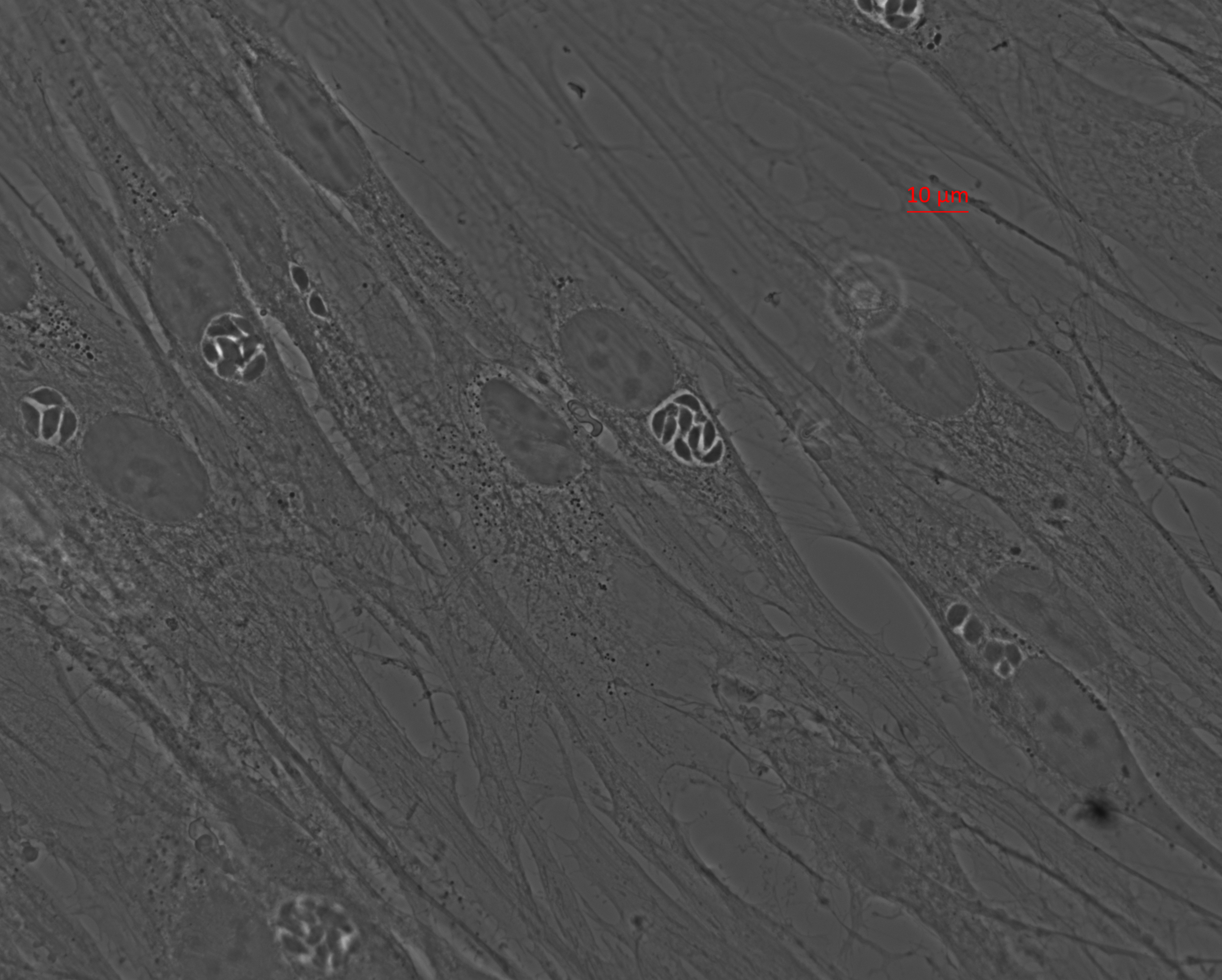

Supplement: Supplementary file 8 — Source data Fig. 2 [file 44321_2025_252_MOESM8_ESM.zip › Figure 2 Source Data/2b/BSM IFA (red) in 76K vs 76K BSM KO/76K UT/Snap-4028_c1 (Phase).tif]

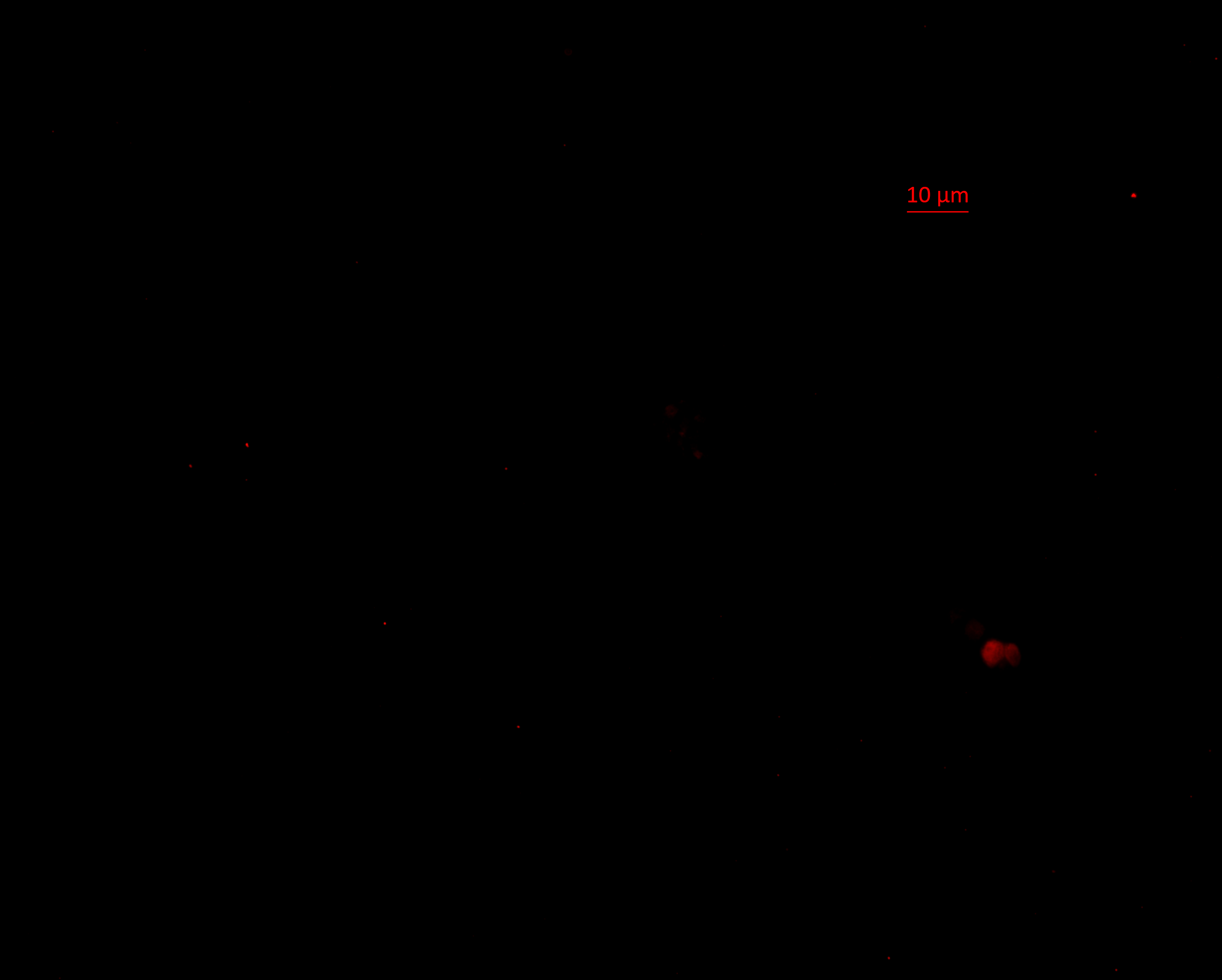

Supplement: Supplementary file 8 — Source data Fig. 2 [file 44321_2025_252_MOESM8_ESM.zip › Figure 2 Source Data/2b/BSM IFA (red) in 76K vs 76K BSM KO/76K UT/Snap-4028_c3 (BSM).tif]

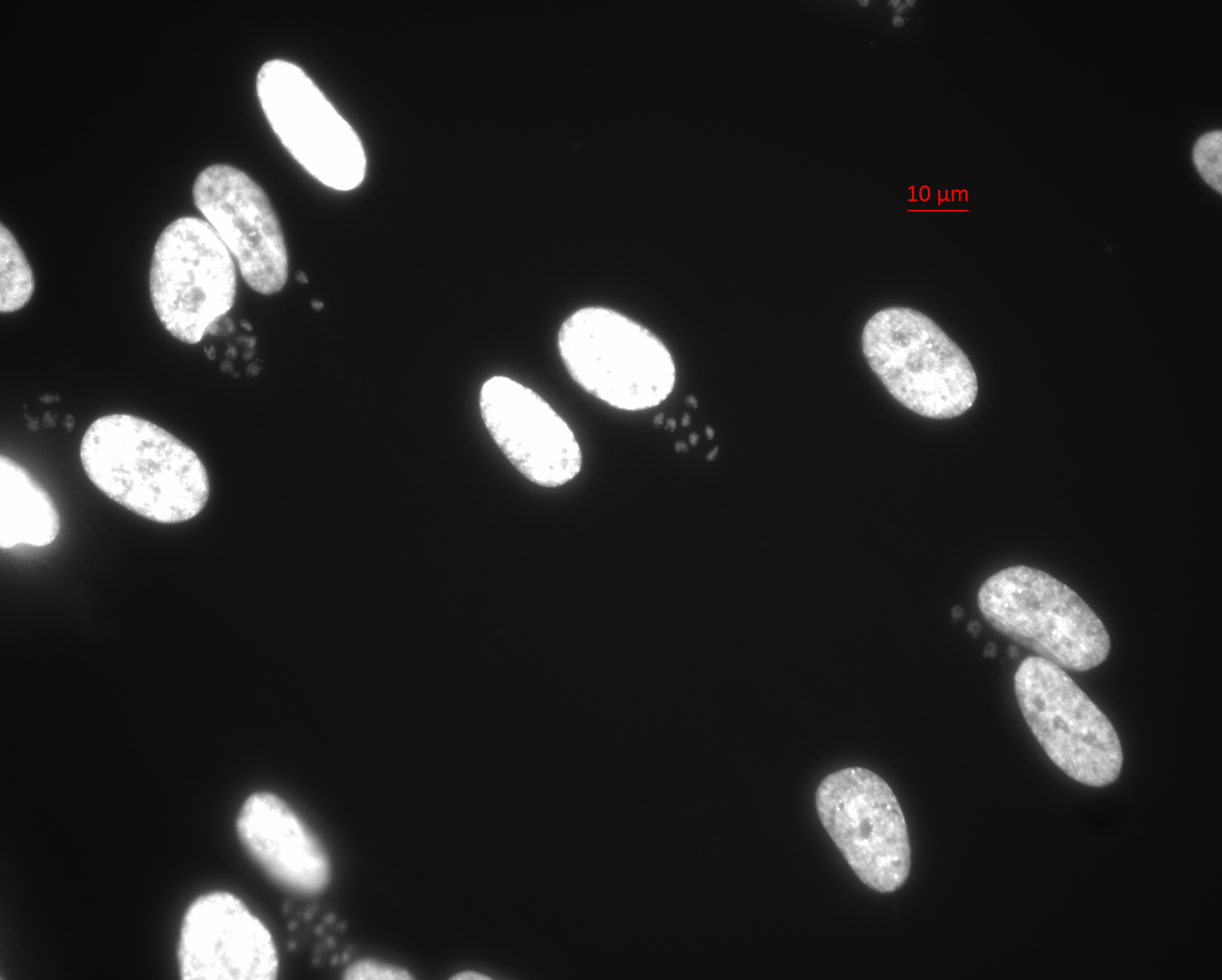

Supplement: Supplementary file 8 — Source data Fig. 2 [file 44321_2025_252_MOESM8_ESM.zip › Figure 2 Source Data/2b/BSM IFA (red) in 76K vs 76K BSM KO/76K UT/Snap-4028_c2 (DNA).tif]

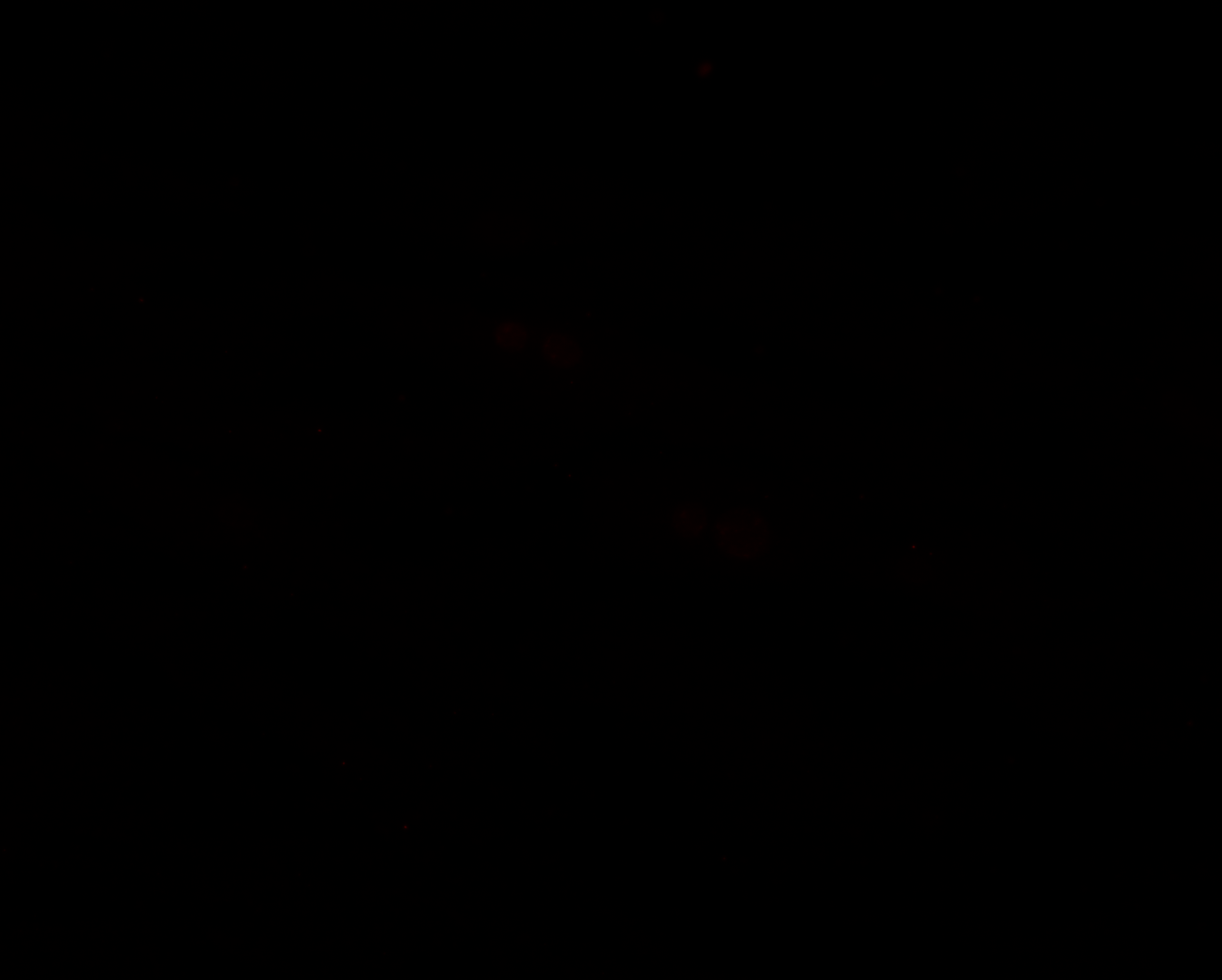

Supplement: Supplementary file 8 — Source data Fig. 2 [file 44321_2025_252_MOESM8_ESM.zip › Figure 2 Source Data/2b/BSM IFA (red) in 76K vs 76K BSM KO/76K BSM KO FR235222 24h/Snap-4044_c3 (BSM).tif]

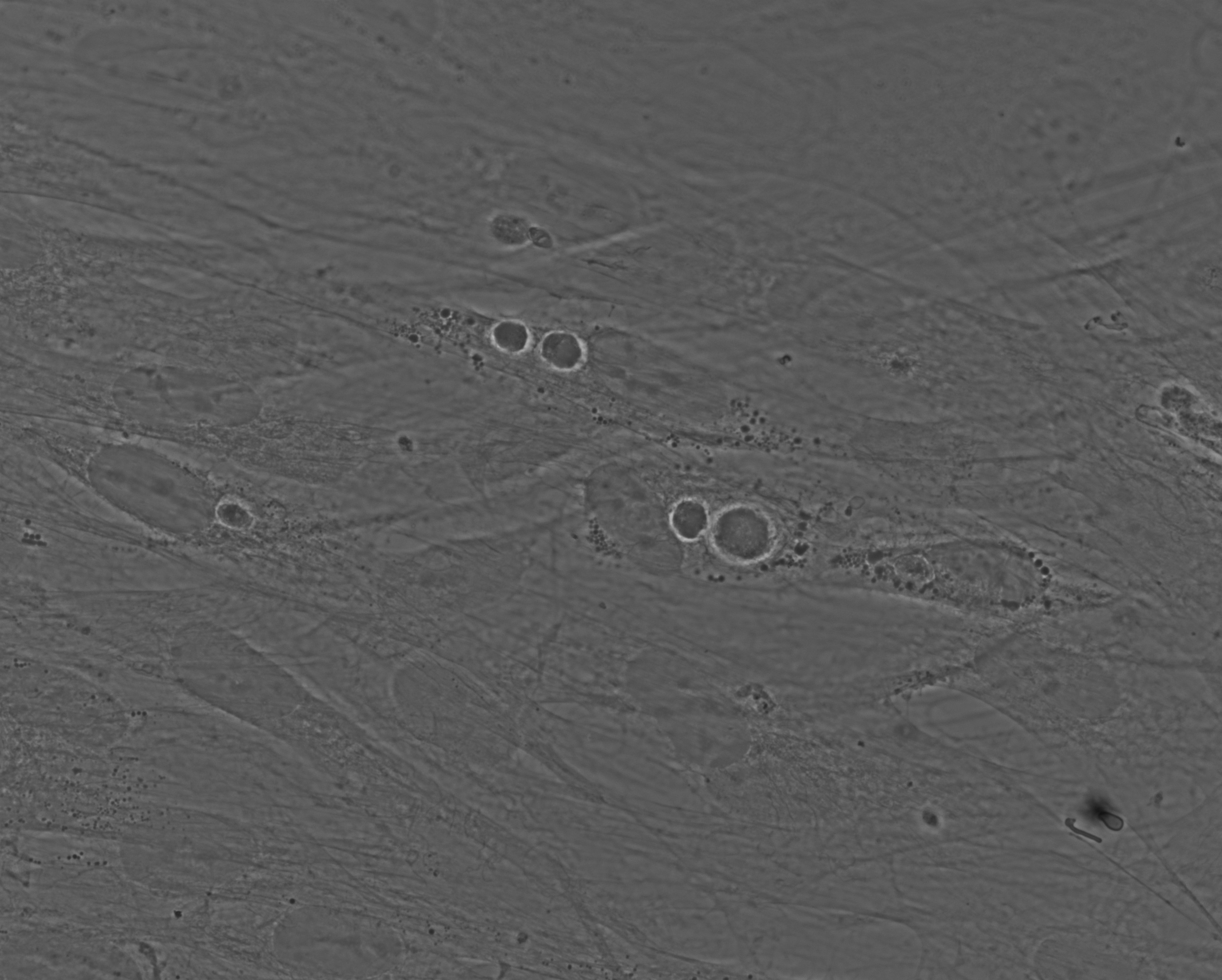

Supplement: Supplementary file 8 — Source data Fig. 2 [file 44321_2025_252_MOESM8_ESM.zip › Figure 2 Source Data/2b/BSM IFA (red) in 76K vs 76K BSM KO/76K BSM KO FR235222 24h/Snap-4044_c1 (Phase).tif]

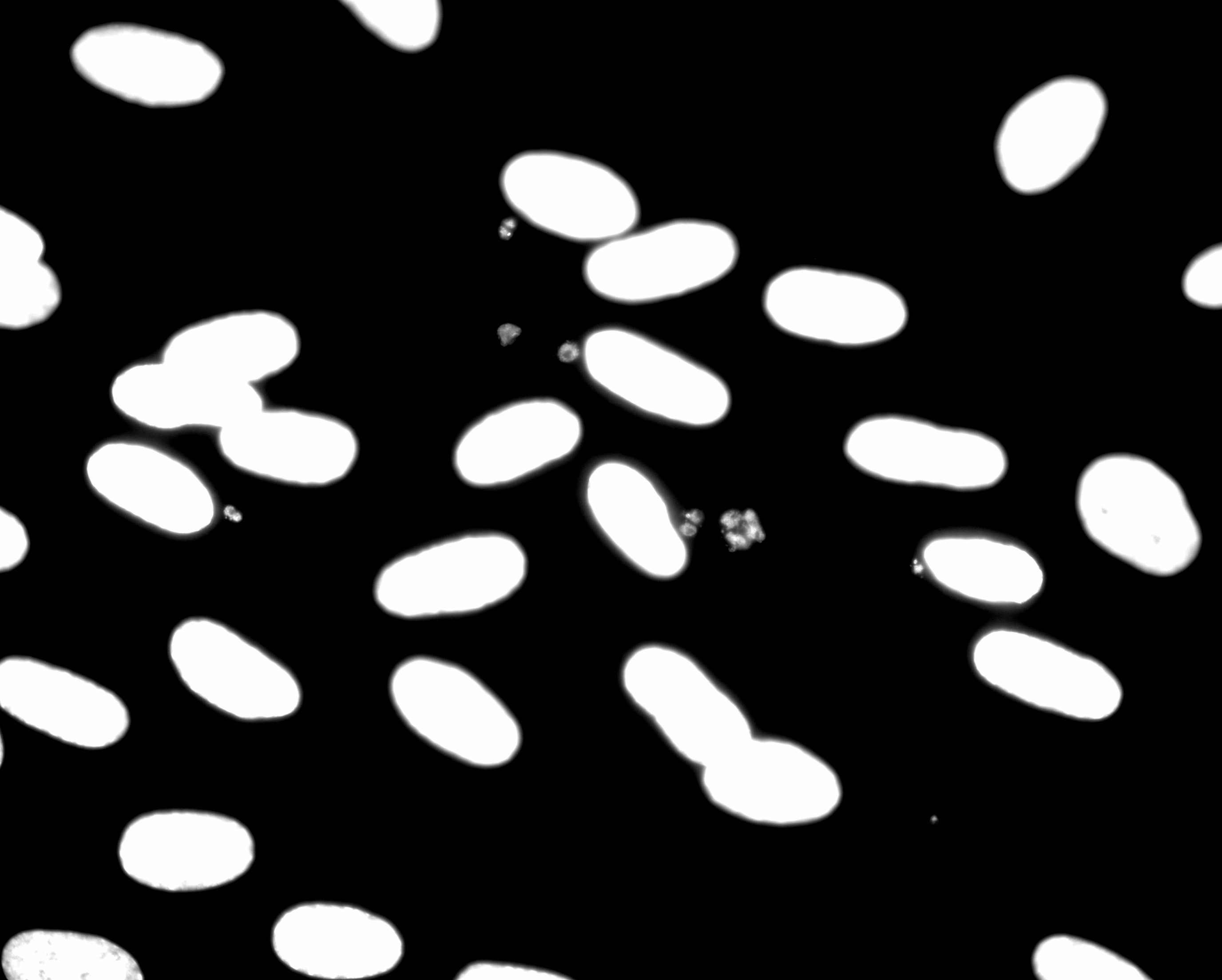

Supplement: Supplementary file 8 — Source data Fig. 2 [file 44321_2025_252_MOESM8_ESM.zip › Figure 2 Source Data/2b/BSM IFA (red) in 76K vs 76K BSM KO/76K BSM KO FR235222 24h/Snap-4044_c2 (DNA).tif]

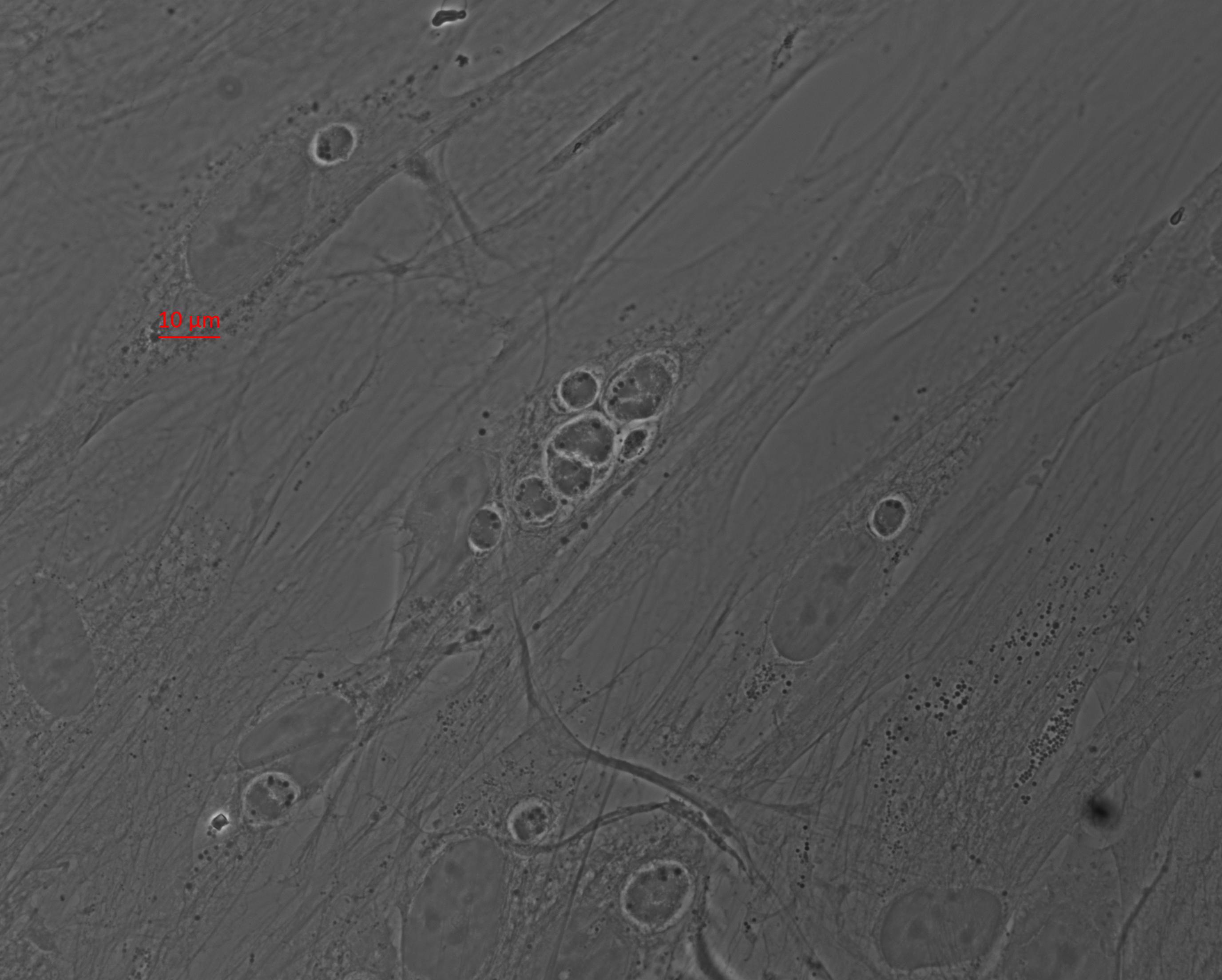

Supplement: Supplementary file 8 — Source data Fig. 2 [file 44321_2025_252_MOESM8_ESM.zip › Figure 2 Source Data/2b/BSM IFA (red) in 76K vs 76K BSM KO/76K FR235222 24h/Snap-4023_c1 (Phase).tif]

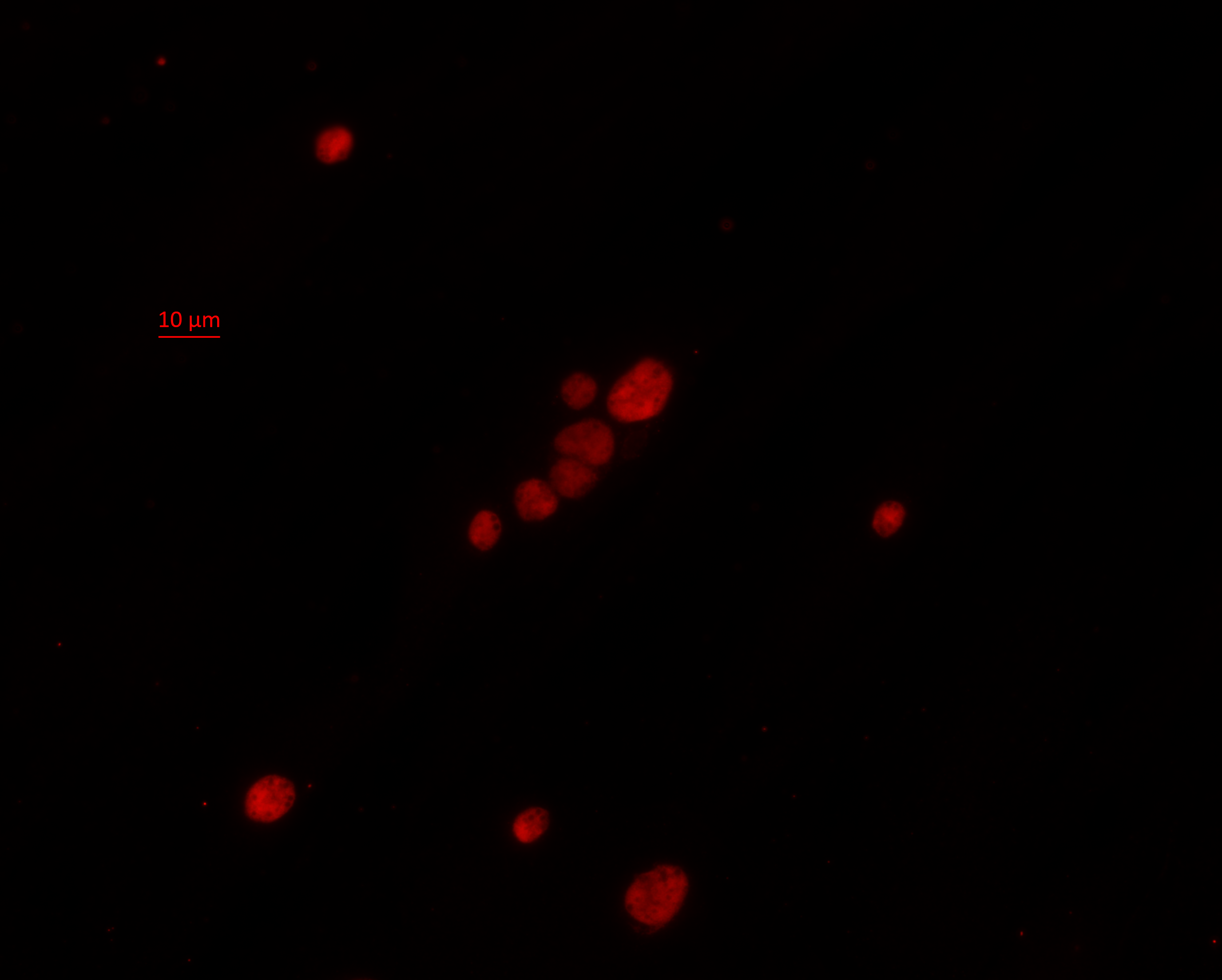

Supplement: Supplementary file 8 — Source data Fig. 2 [file 44321_2025_252_MOESM8_ESM.zip › Figure 2 Source Data/2b/BSM IFA (red) in 76K vs 76K BSM KO/76K FR235222 24h/Snap-4023_c3 (BSM).tif]

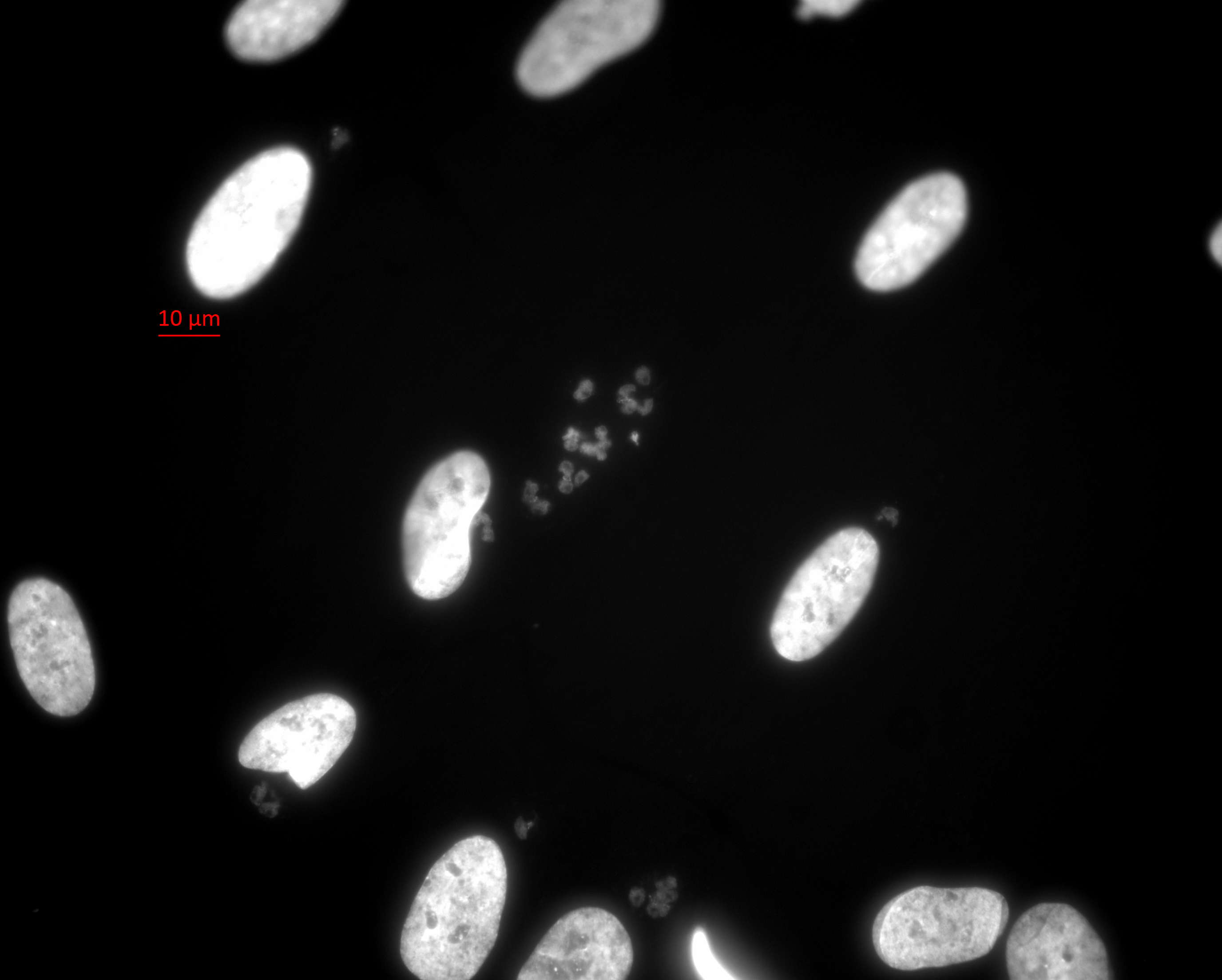

Supplement: Supplementary file 8 — Source data Fig. 2 [file 44321_2025_252_MOESM8_ESM.zip › Figure 2 Source Data/2b/BSM IFA (red) in 76K vs 76K BSM KO/76K FR235222 24h/Snap-4023_c2 (DNA).tif]

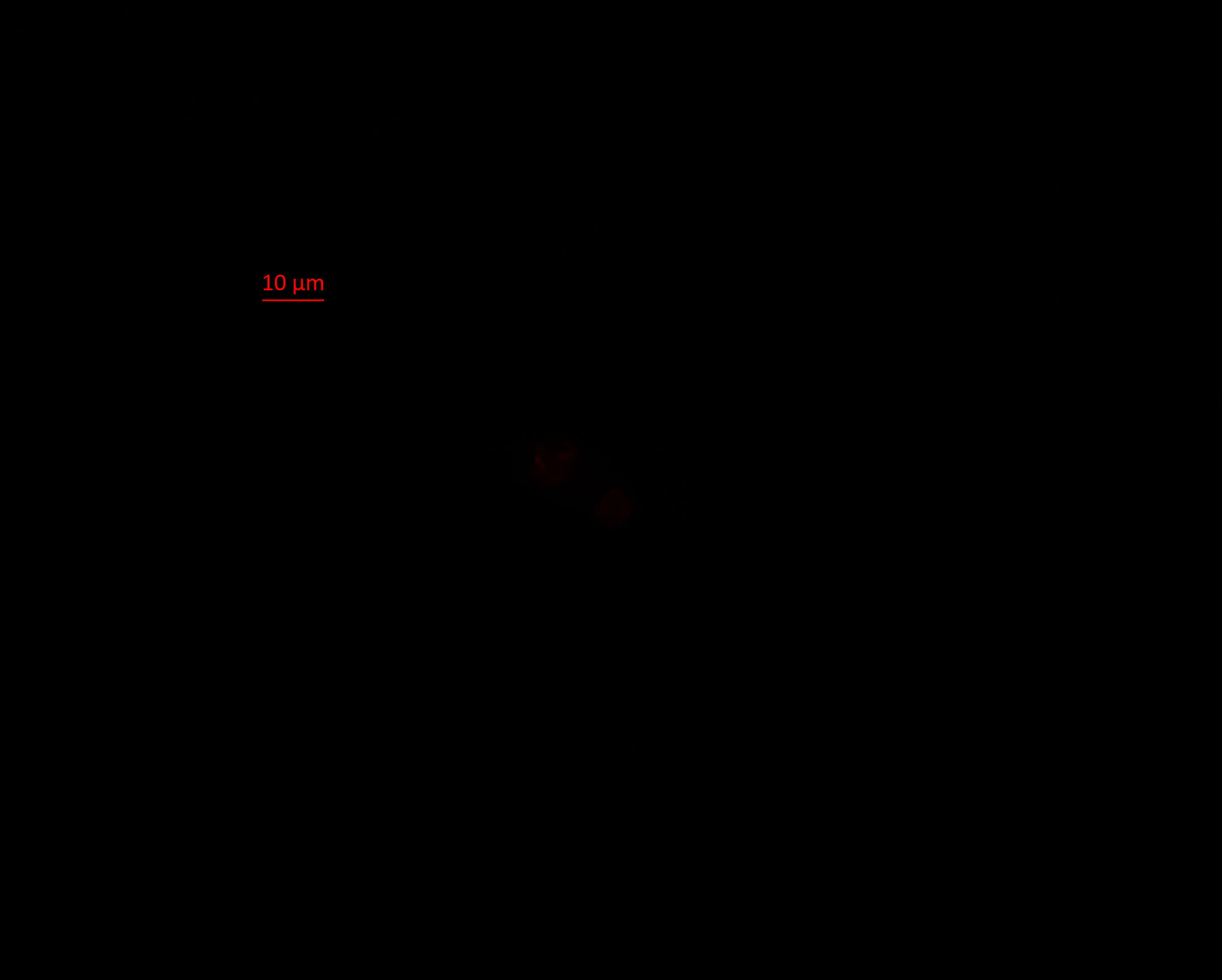

Supplement: Supplementary file 8 — Source data Fig. 2 [file 44321_2025_252_MOESM8_ESM.zip › Figure 2 Source Data/2b/BCLA IFA (red) in 76K vs 76K BSM KO/76K BSM KO UT/Snap-4062_c3 (BCLA).tif]

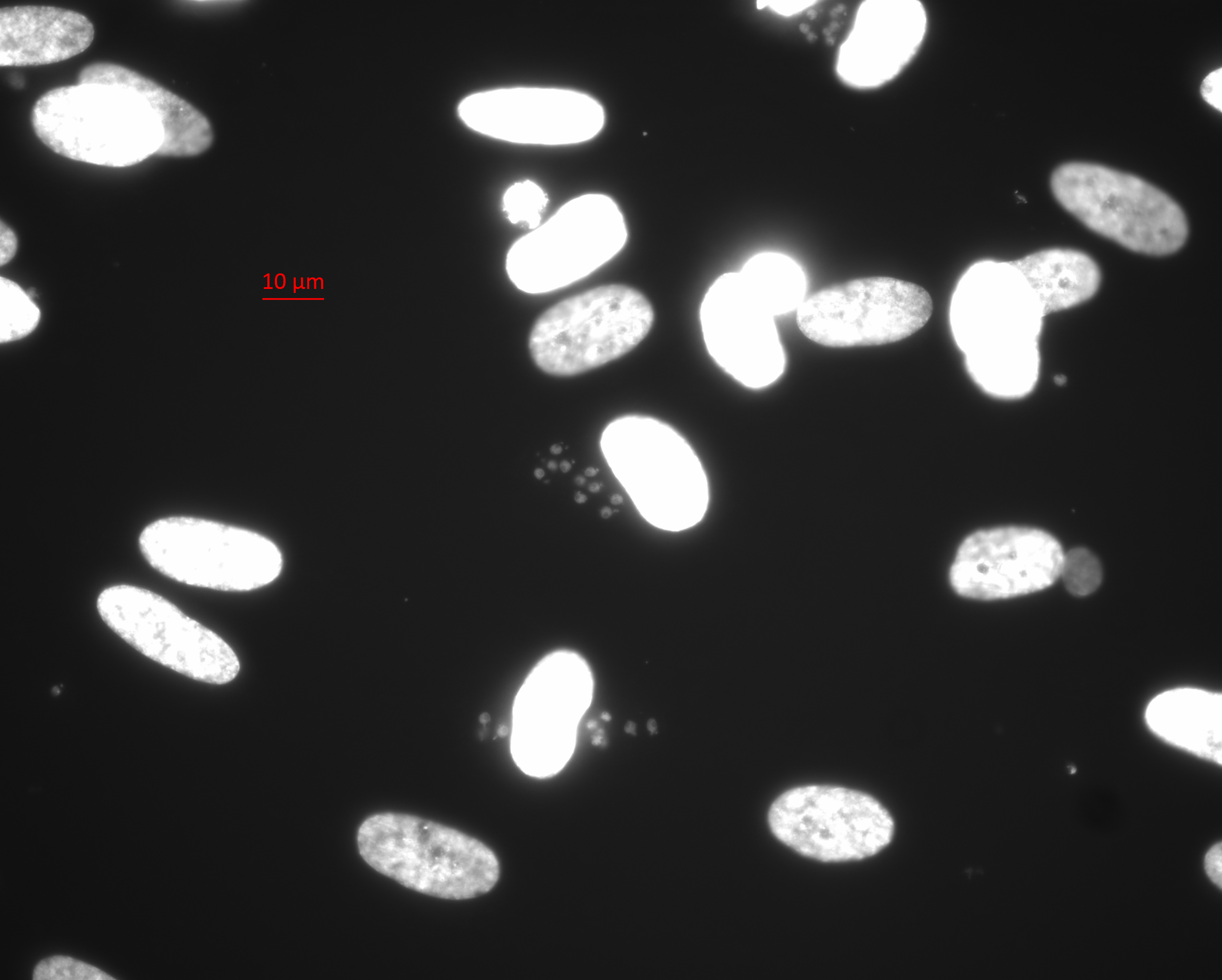

Supplement: Supplementary file 8 — Source data Fig. 2 [file 44321_2025_252_MOESM8_ESM.zip › Figure 2 Source Data/2b/BCLA IFA (red) in 76K vs 76K BSM KO/76K BSM KO UT/Snap-4062_c2 (DNA).tif]

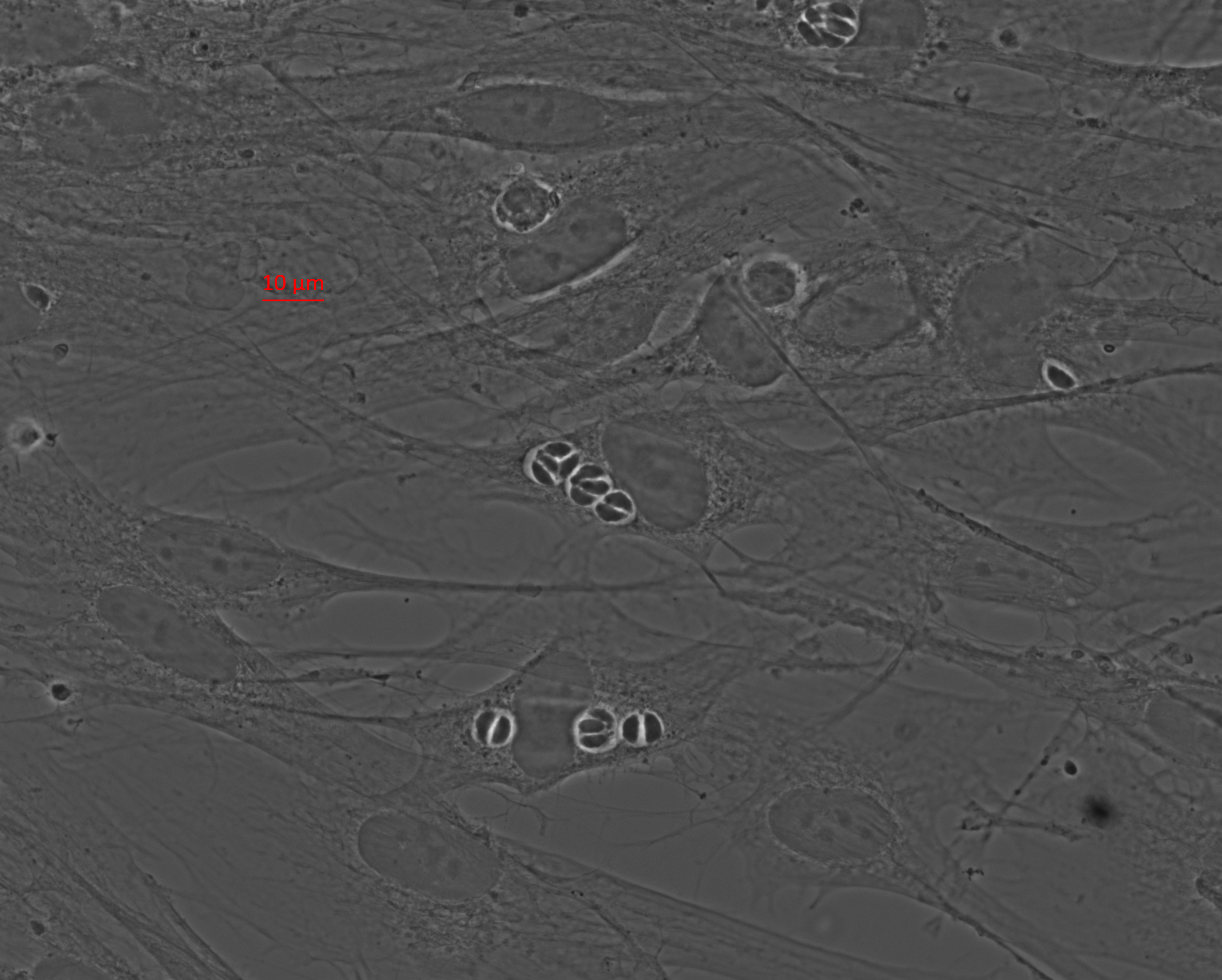

Supplement: Supplementary file 8 — Source data Fig. 2 [file 44321_2025_252_MOESM8_ESM.zip › Figure 2 Source Data/2b/BCLA IFA (red) in 76K vs 76K BSM KO/76K BSM KO UT/Snap-4062_c1 (Phase).tif]

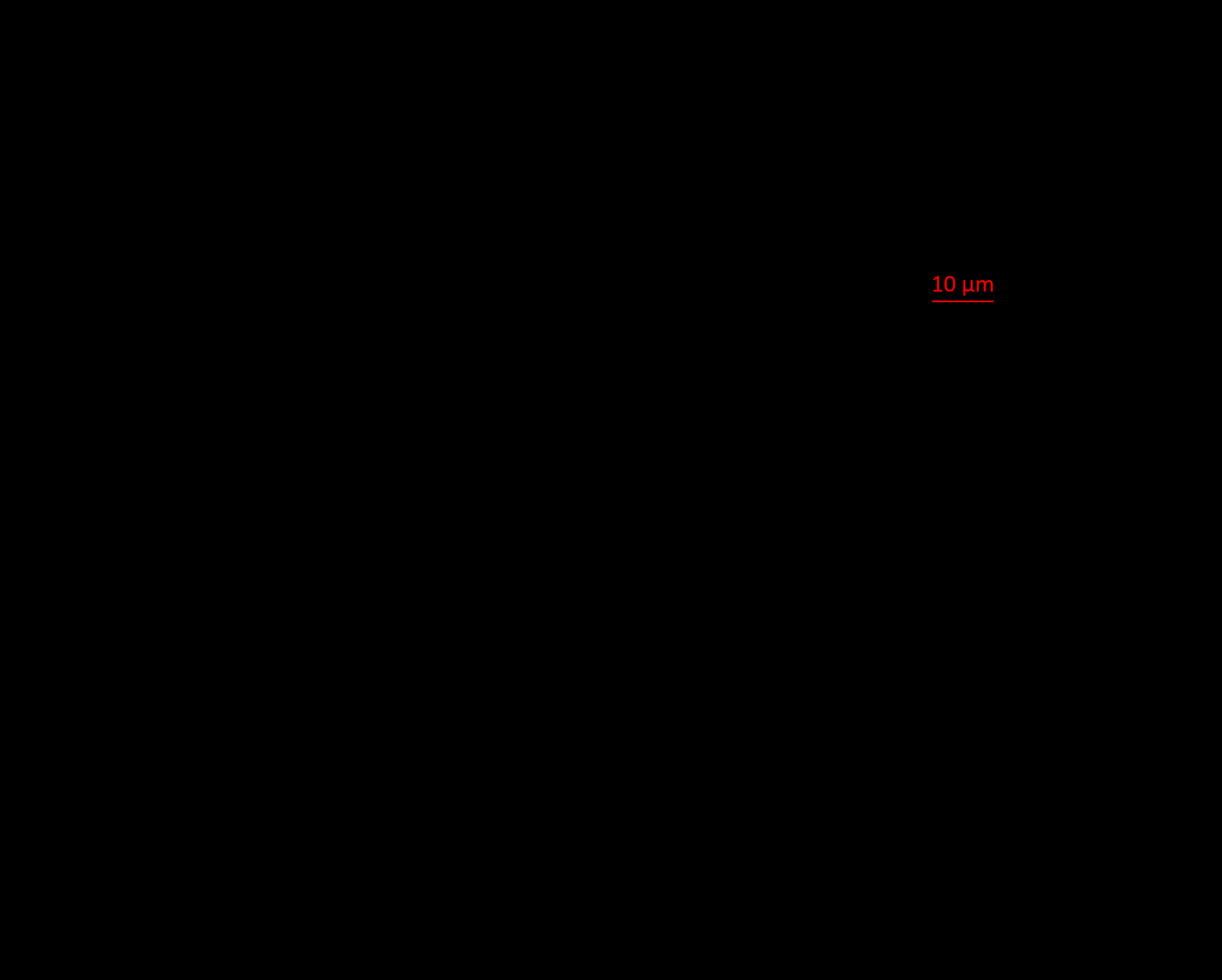

Supplement: Supplementary file 8 — Source data Fig. 2 [file 44321_2025_252_MOESM8_ESM.zip › Figure 2 Source Data/2b/BCLA IFA (red) in 76K vs 76K BSM KO/76K UT/Snap-4059_c3 (BCLA).tif]

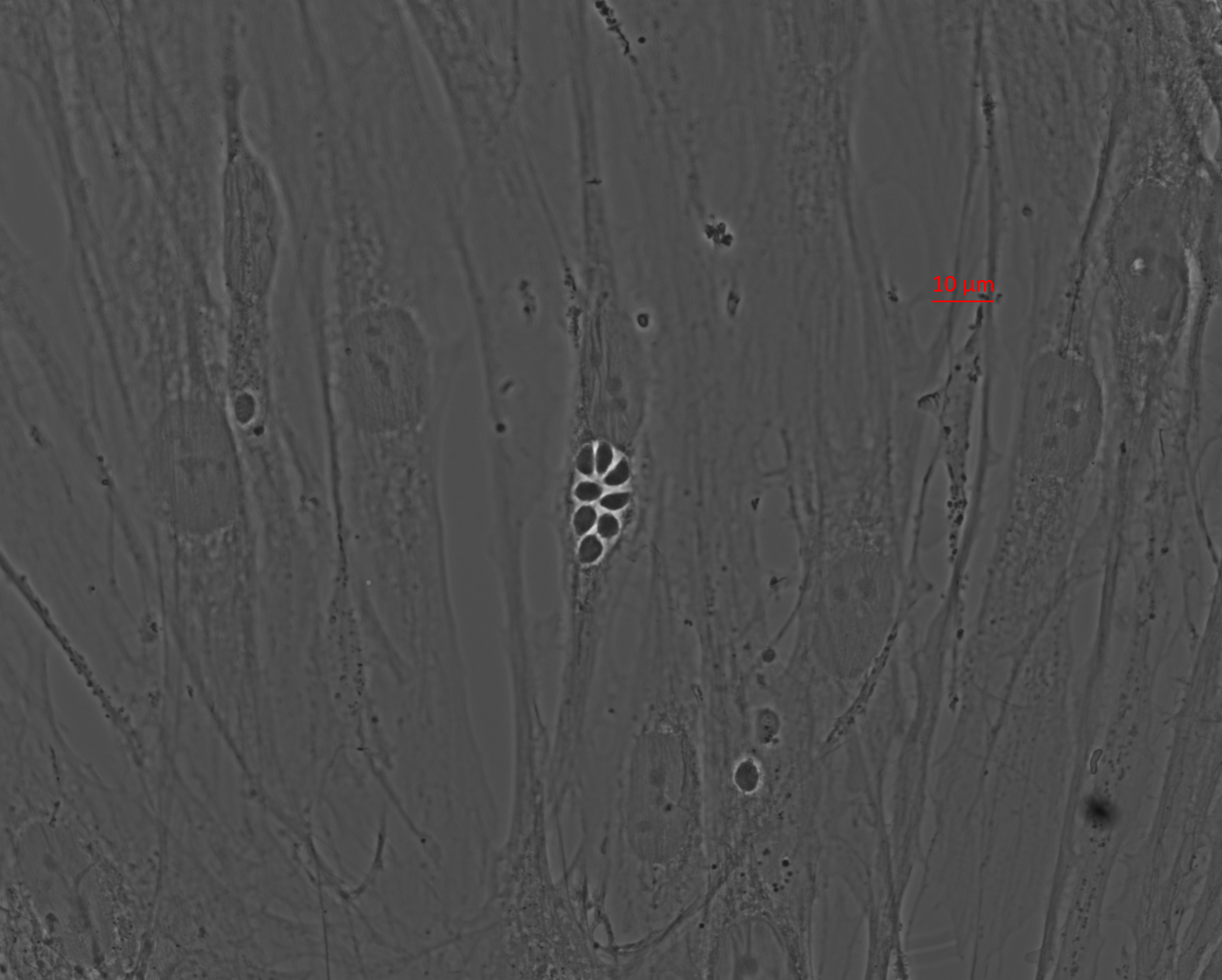

Supplement: Supplementary file 8 — Source data Fig. 2 [file 44321_2025_252_MOESM8_ESM.zip › Figure 2 Source Data/2b/BCLA IFA (red) in 76K vs 76K BSM KO/76K UT/Snap-4059_c1 (Phase).tif]

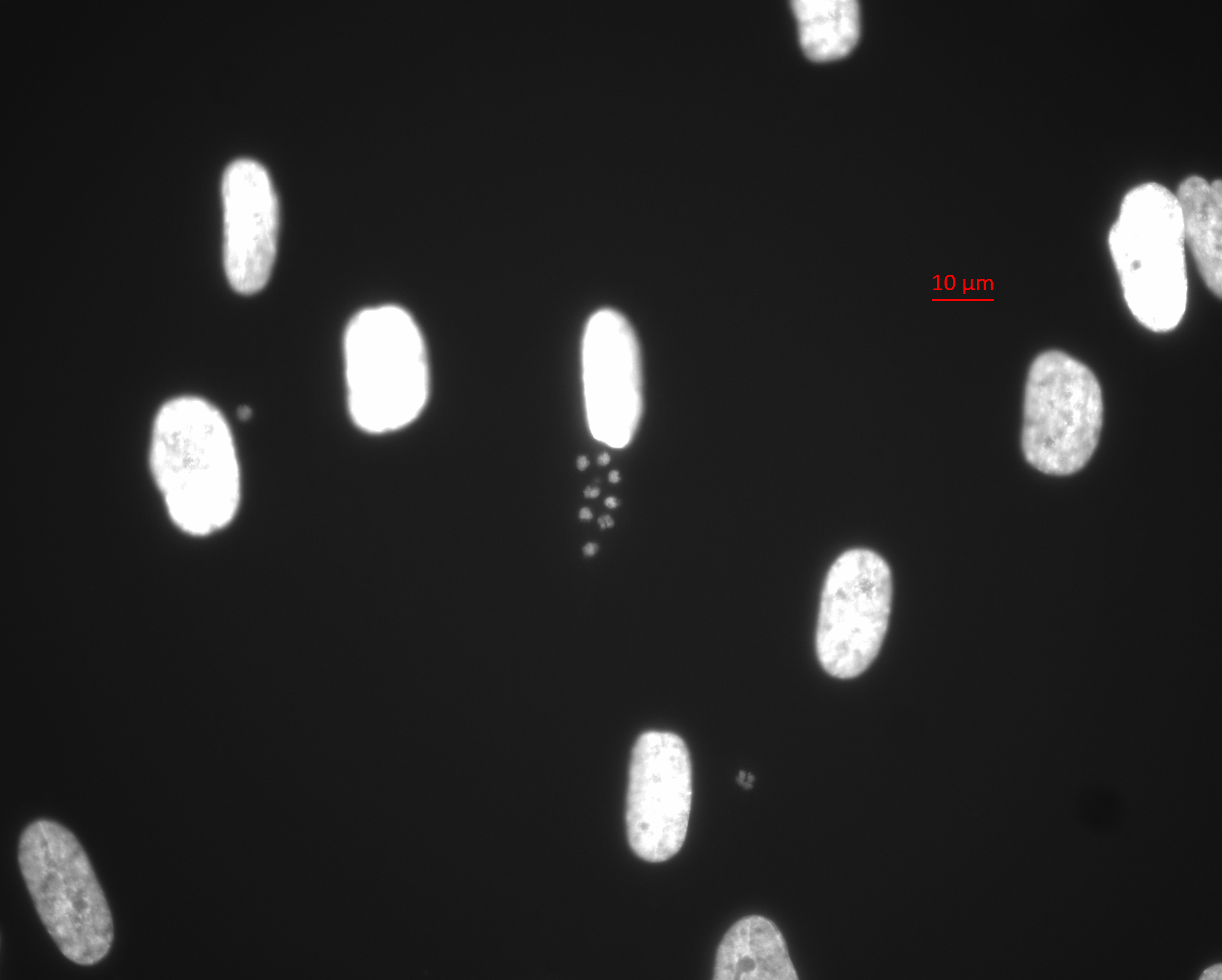

Supplement: Supplementary file 8 — Source data Fig. 2 [file 44321_2025_252_MOESM8_ESM.zip › Figure 2 Source Data/2b/BCLA IFA (red) in 76K vs 76K BSM KO/76K UT/Snap-4059_c2 (DNA).tif]

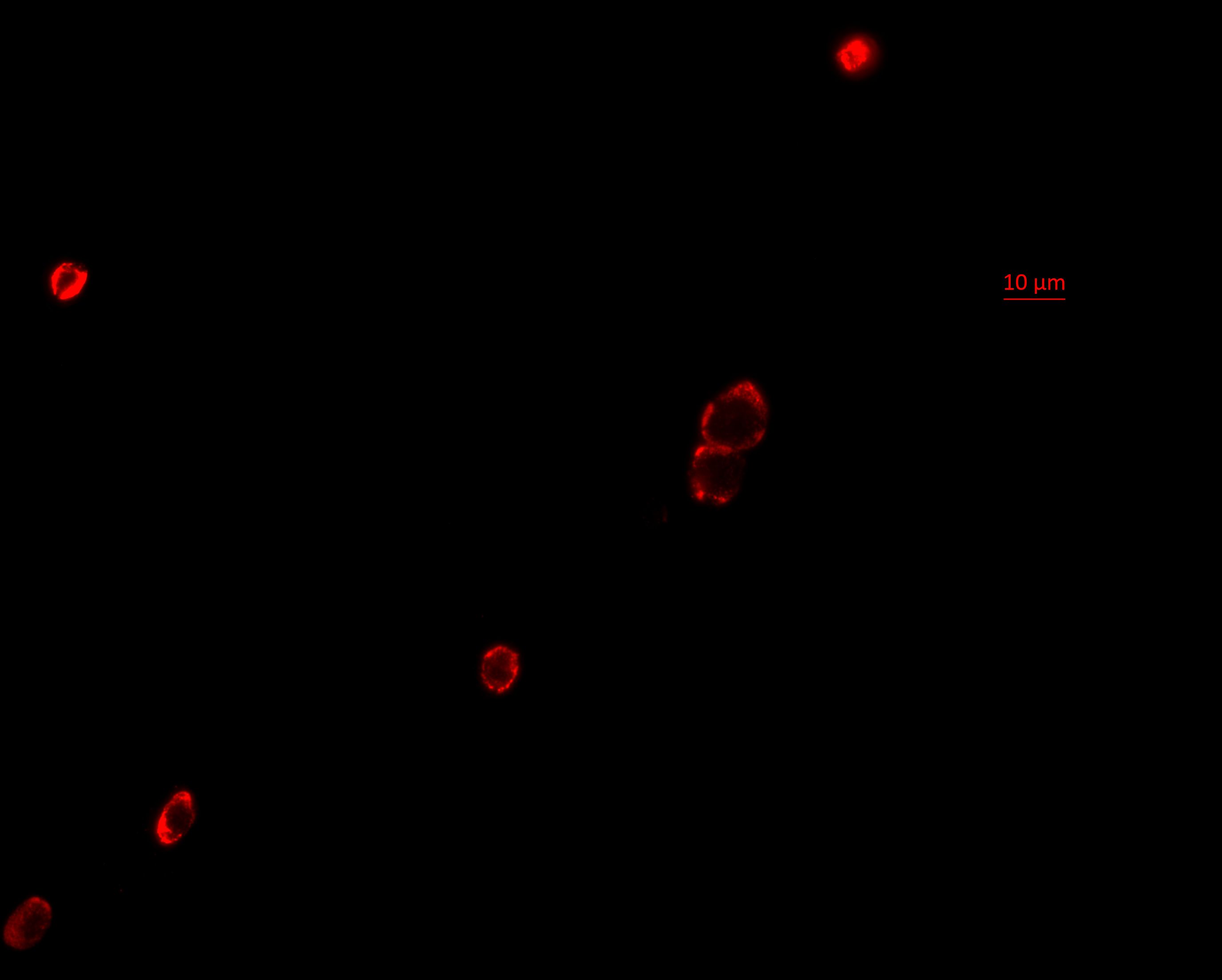

Supplement: Supplementary file 8 — Source data Fig. 2 [file 44321_2025_252_MOESM8_ESM.zip › Figure 2 Source Data/2b/BCLA IFA (red) in 76K vs 76K BSM KO/76K BSM KO FR235222 24h/Snap-4054_c3 (BCLA).tif]

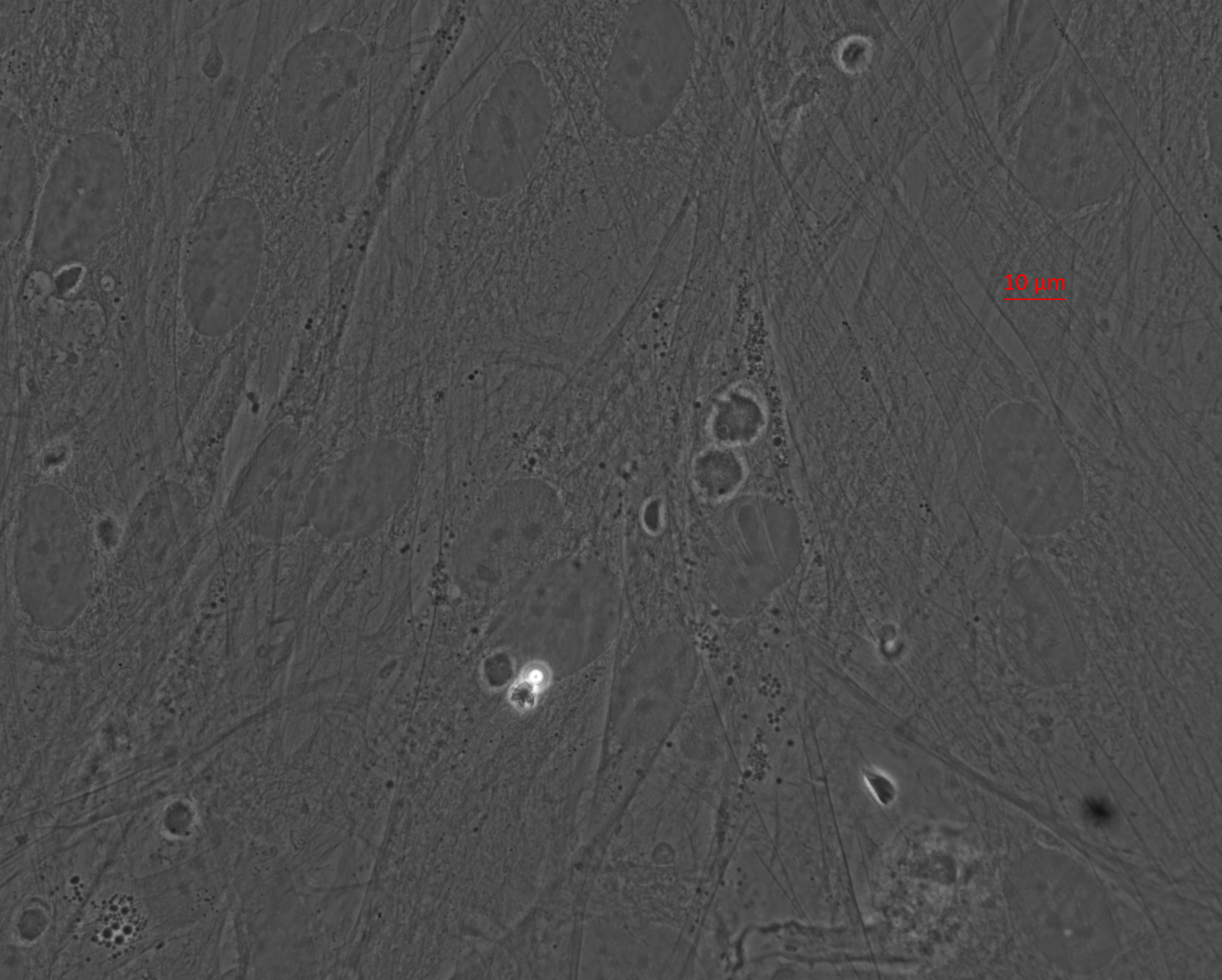

Supplement: Supplementary file 8 — Source data Fig. 2 [file 44321_2025_252_MOESM8_ESM.zip › Figure 2 Source Data/2b/BCLA IFA (red) in 76K vs 76K BSM KO/76K BSM KO FR235222 24h/Snap-4054_c1 (Phase).tif]

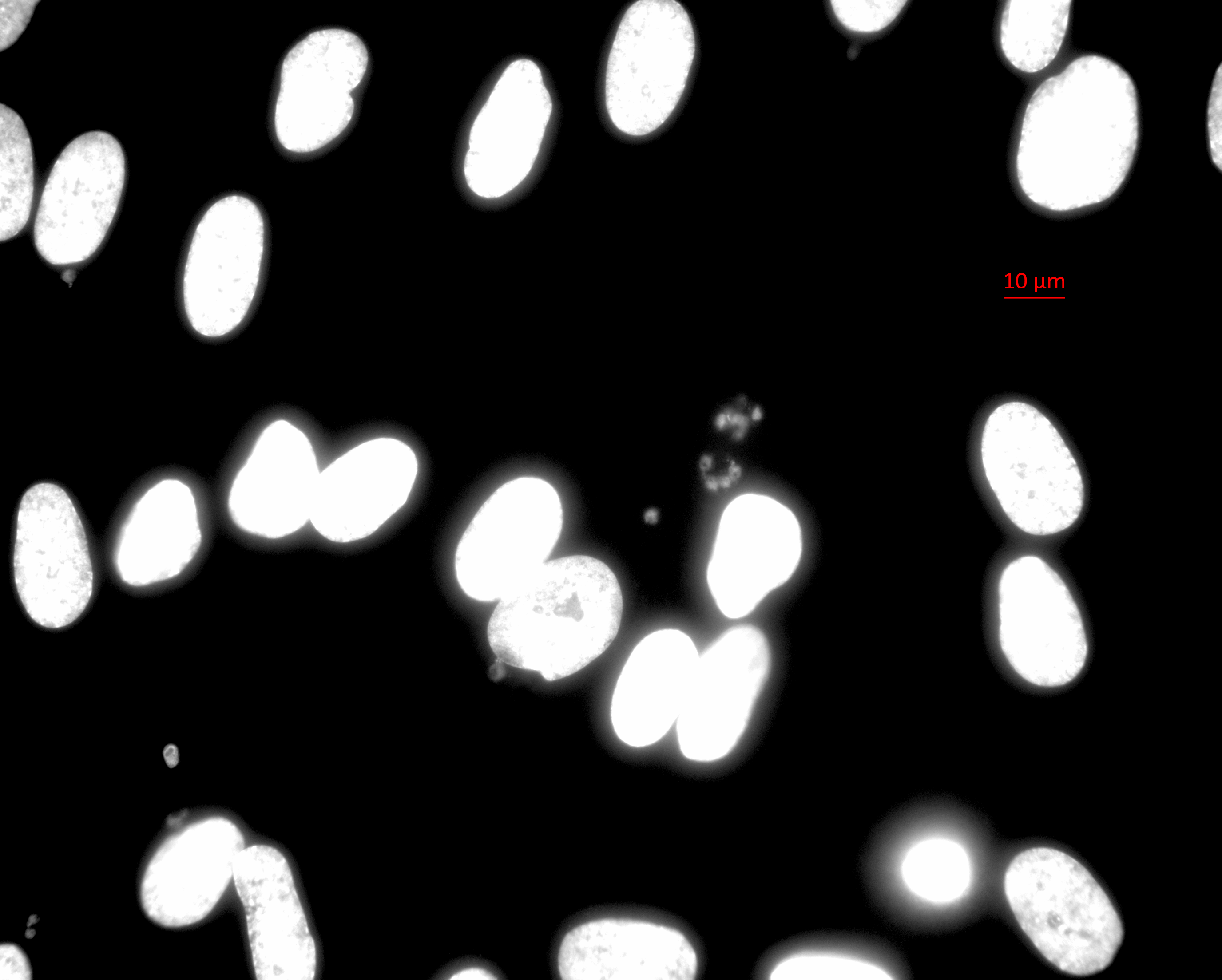

Supplement: Supplementary file 8 — Source data Fig. 2 [file 44321_2025_252_MOESM8_ESM.zip › Figure 2 Source Data/2b/BCLA IFA (red) in 76K vs 76K BSM KO/76K BSM KO FR235222 24h/Snap-4054_c2 (DNA).tif]

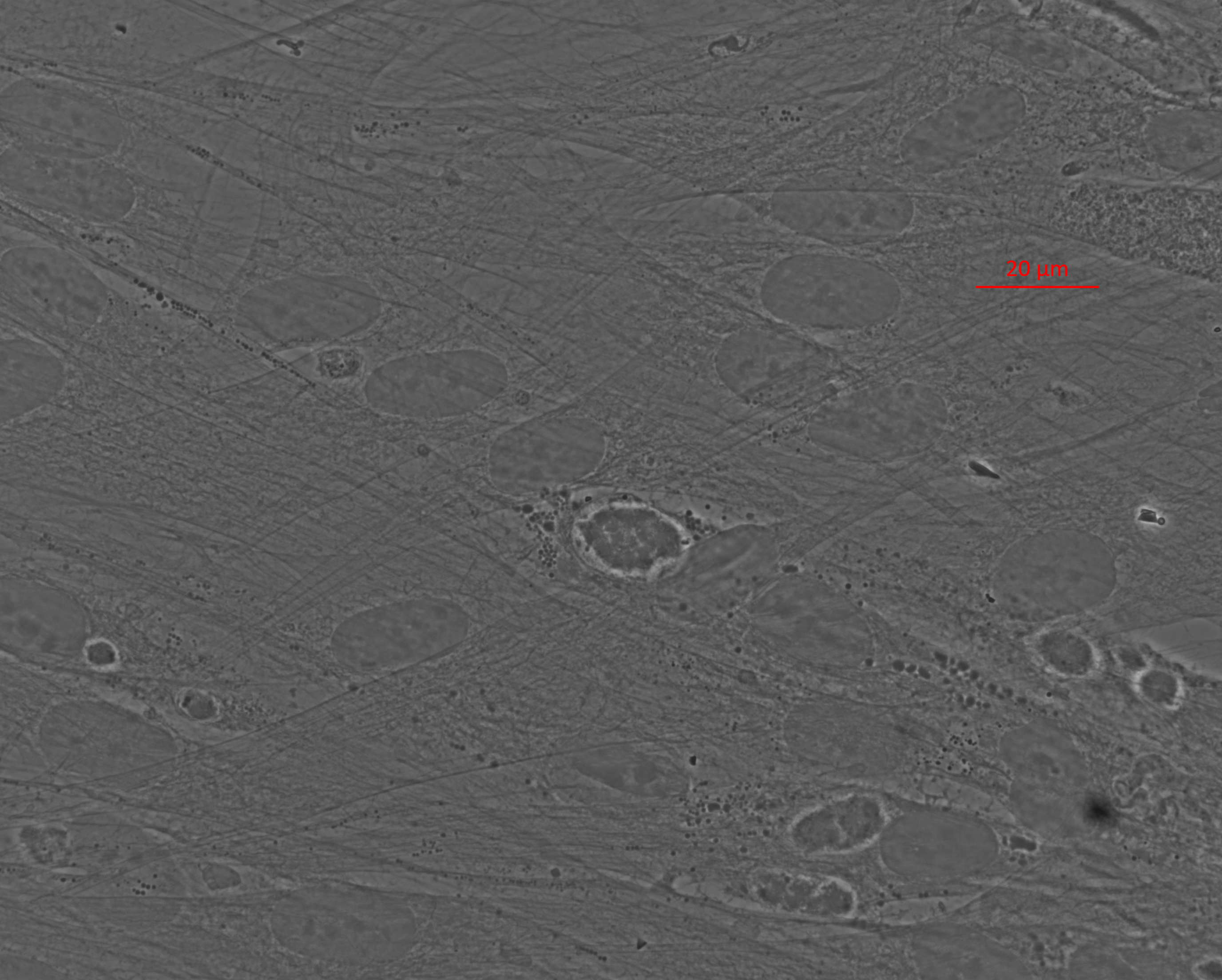

Supplement: Supplementary file 8 — Source data Fig. 2 [file 44321_2025_252_MOESM8_ESM.zip › Figure 2 Source Data/2b/BCLA IFA (red) in 76K vs 76K BSM KO/76K FR235222 24h/Snap-4053_c1 (Phase).tif]

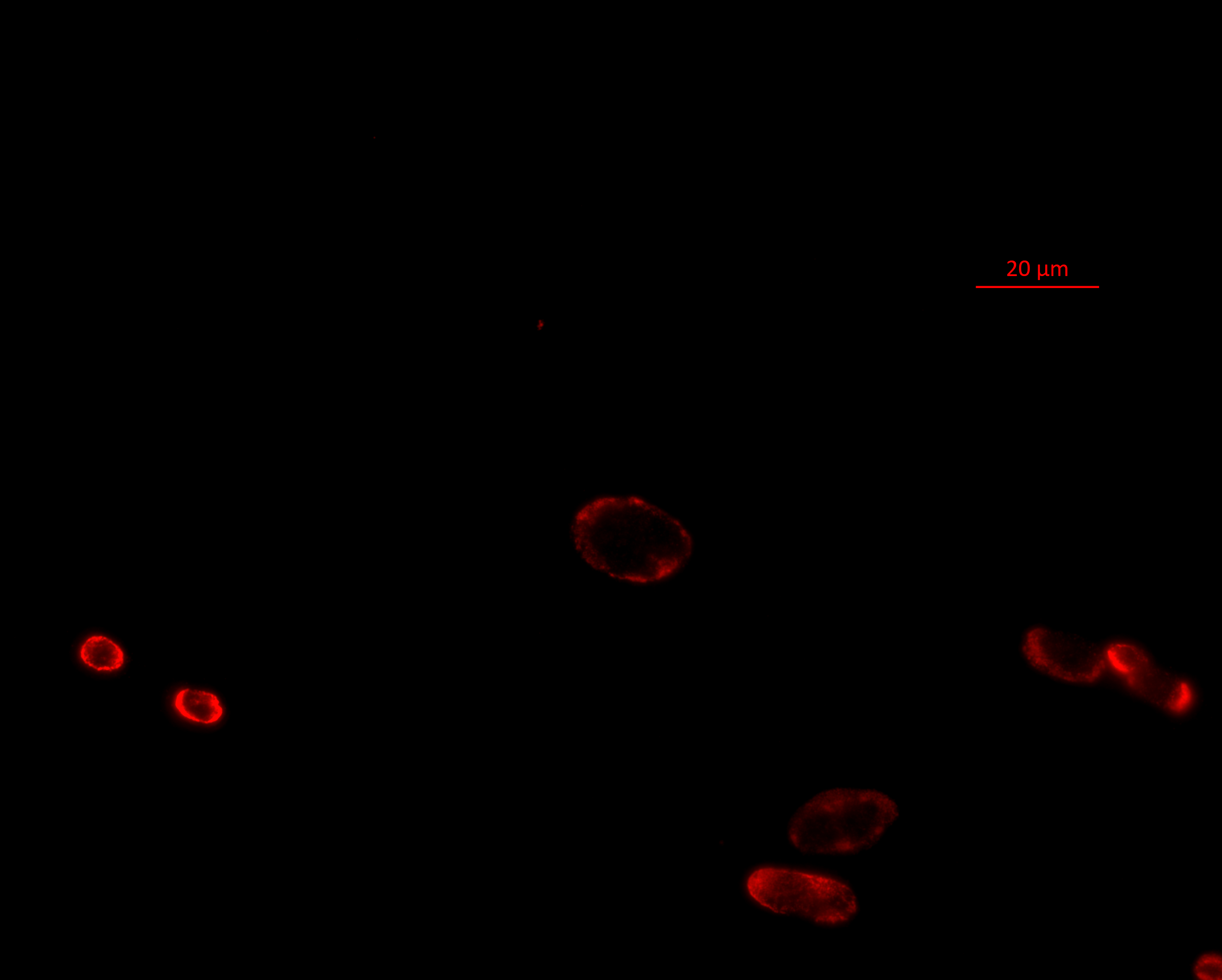

Supplement: Supplementary file 8 — Source data Fig. 2 [file 44321_2025_252_MOESM8_ESM.zip › Figure 2 Source Data/2b/BCLA IFA (red) in 76K vs 76K BSM KO/76K FR235222 24h/Snap-4053_c3 (BCLA).tif]

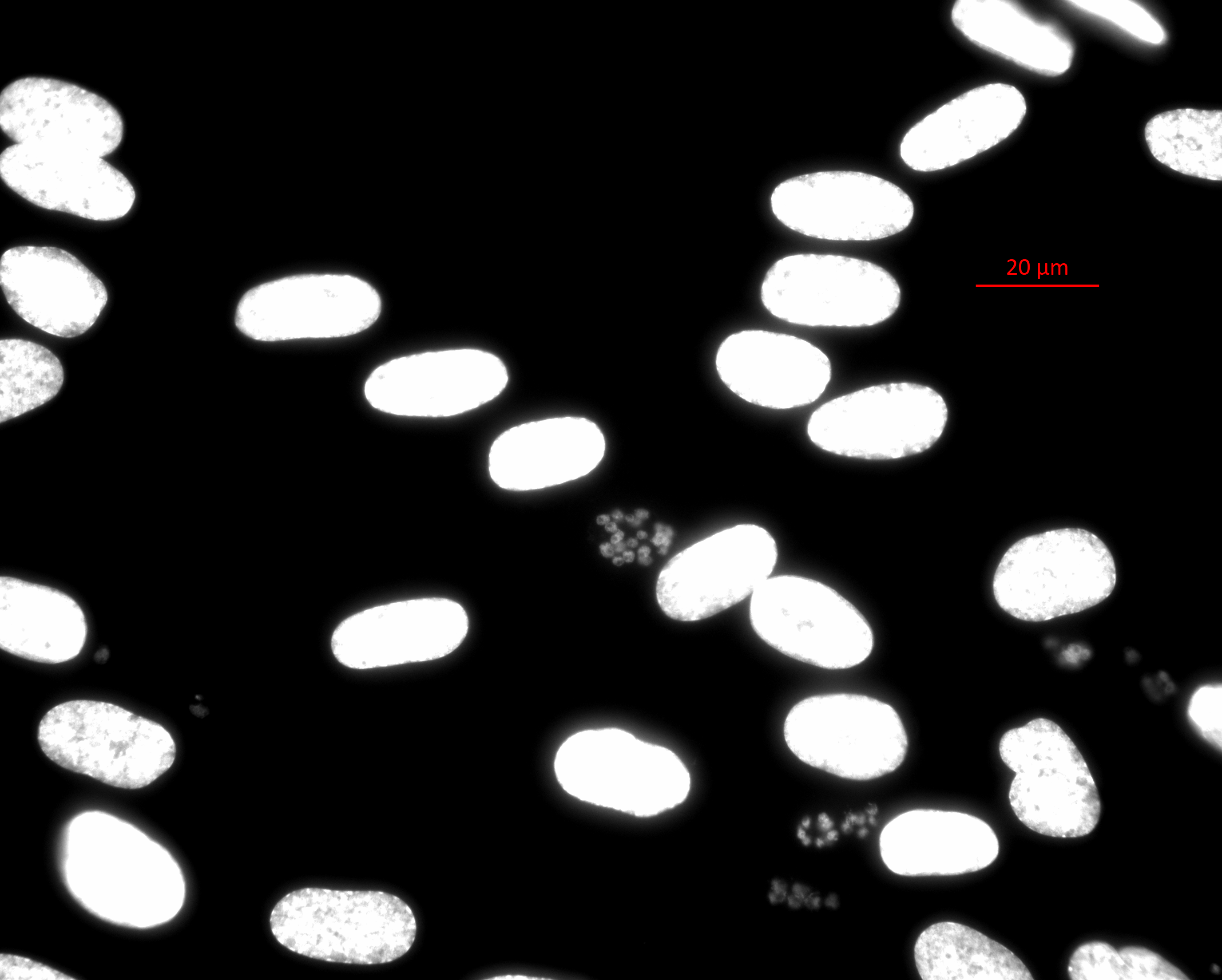

Supplement: Supplementary file 8 — Source data Fig. 2 [file 44321_2025_252_MOESM8_ESM.zip › Figure 2 Source Data/2b/BCLA IFA (red) in 76K vs 76K BSM KO/76K FR235222 24h/Snap-4053_c2 (DNA).tif]

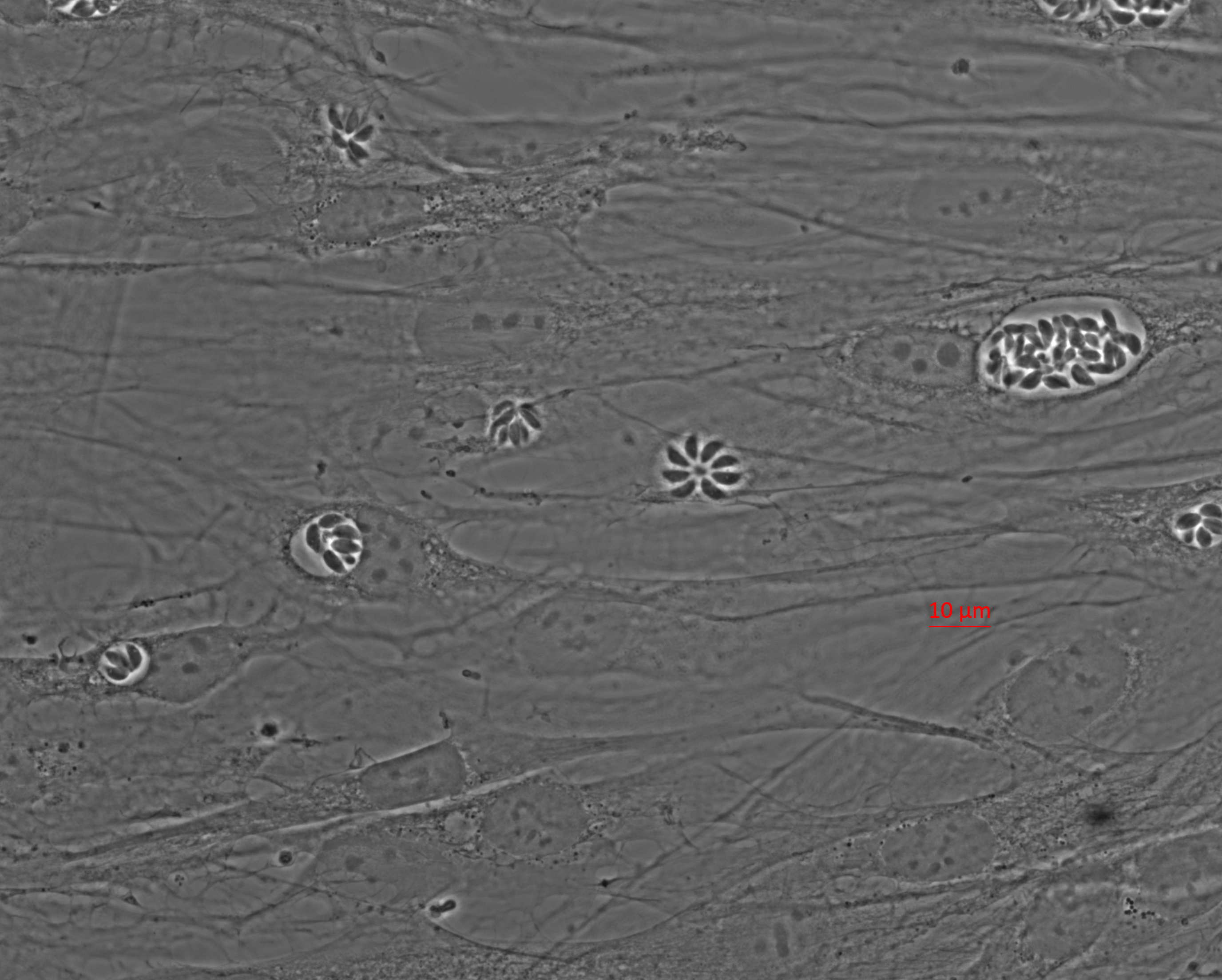

Supplement: Supplementary file 9 — Source data Fig. 3 [file 44321_2025_252_MOESM9_ESM.zip › Figure 3 Source Data/3b/UT/Snap-1795_c1 (Phase).tif]

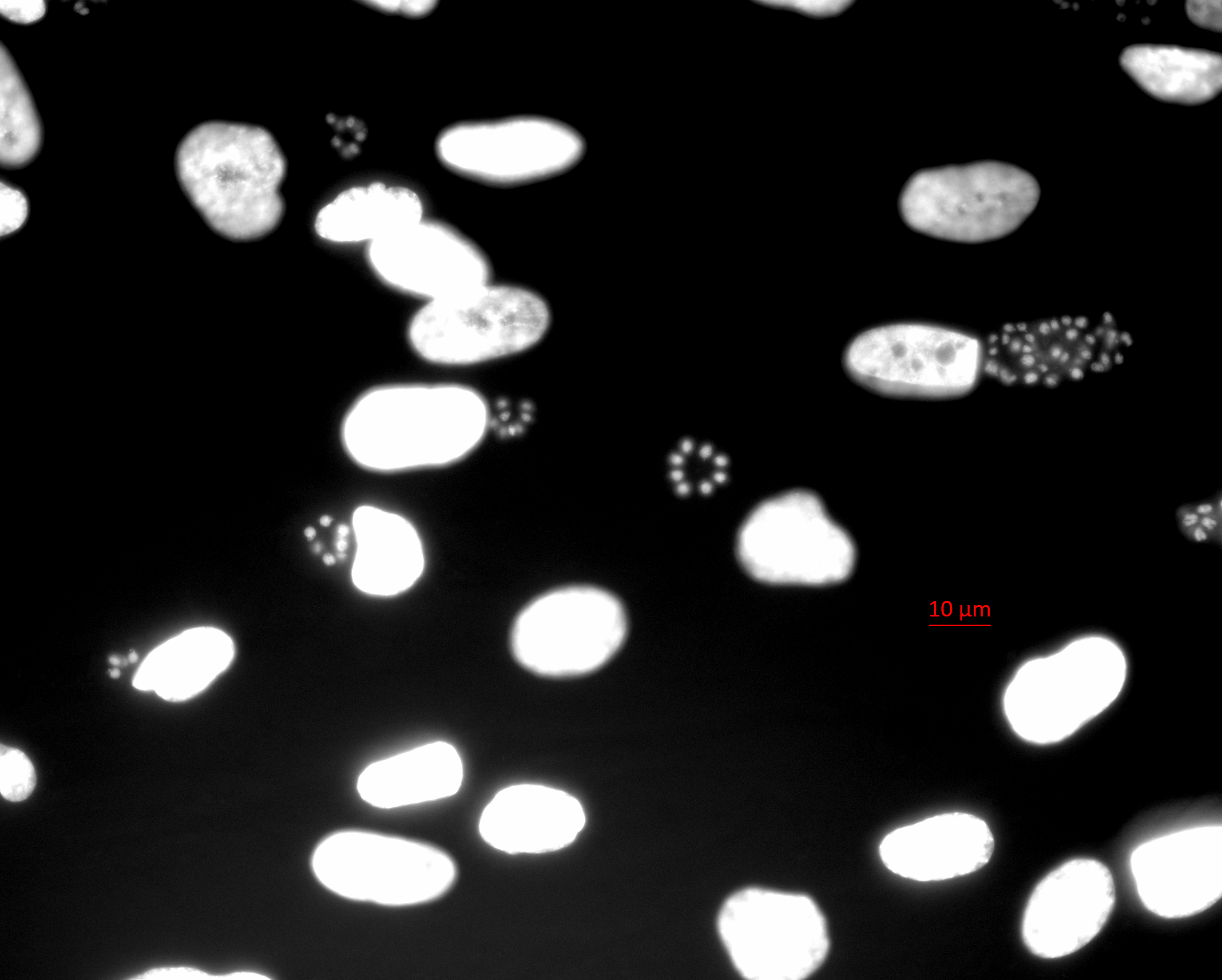

Supplement: Supplementary file 9 — Source data Fig. 3 [file 44321_2025_252_MOESM9_ESM.zip › Figure 3 Source Data/3b/UT/Snap-1795_c2 (DNA).tif]

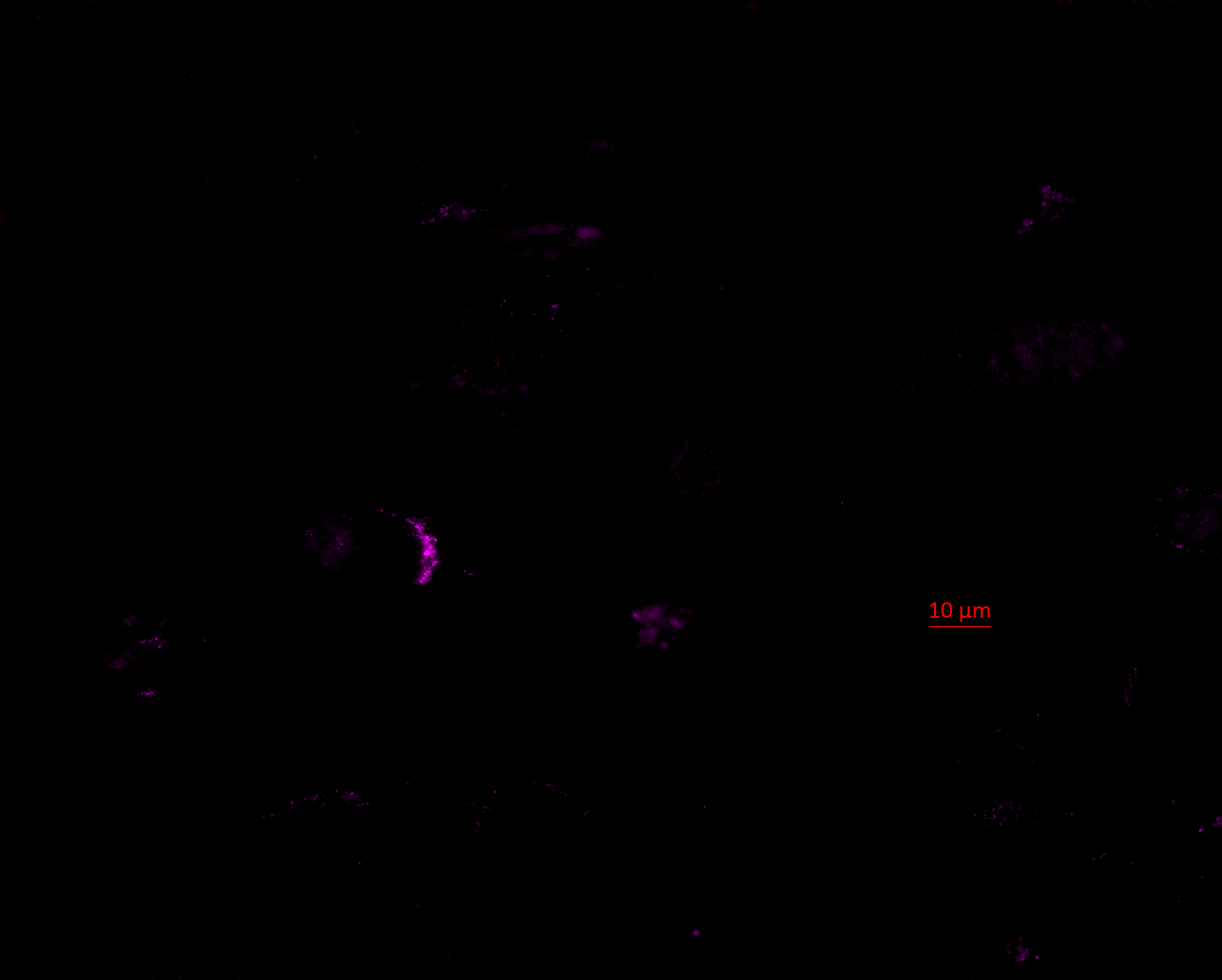

Supplement: Supplementary file 9 — Source data Fig. 3 [file 44321_2025_252_MOESM9_ESM.zip › Figure 3 Source Data/3b/UT/Snap-1795_c3 (BFD1-Ty in red).tif]

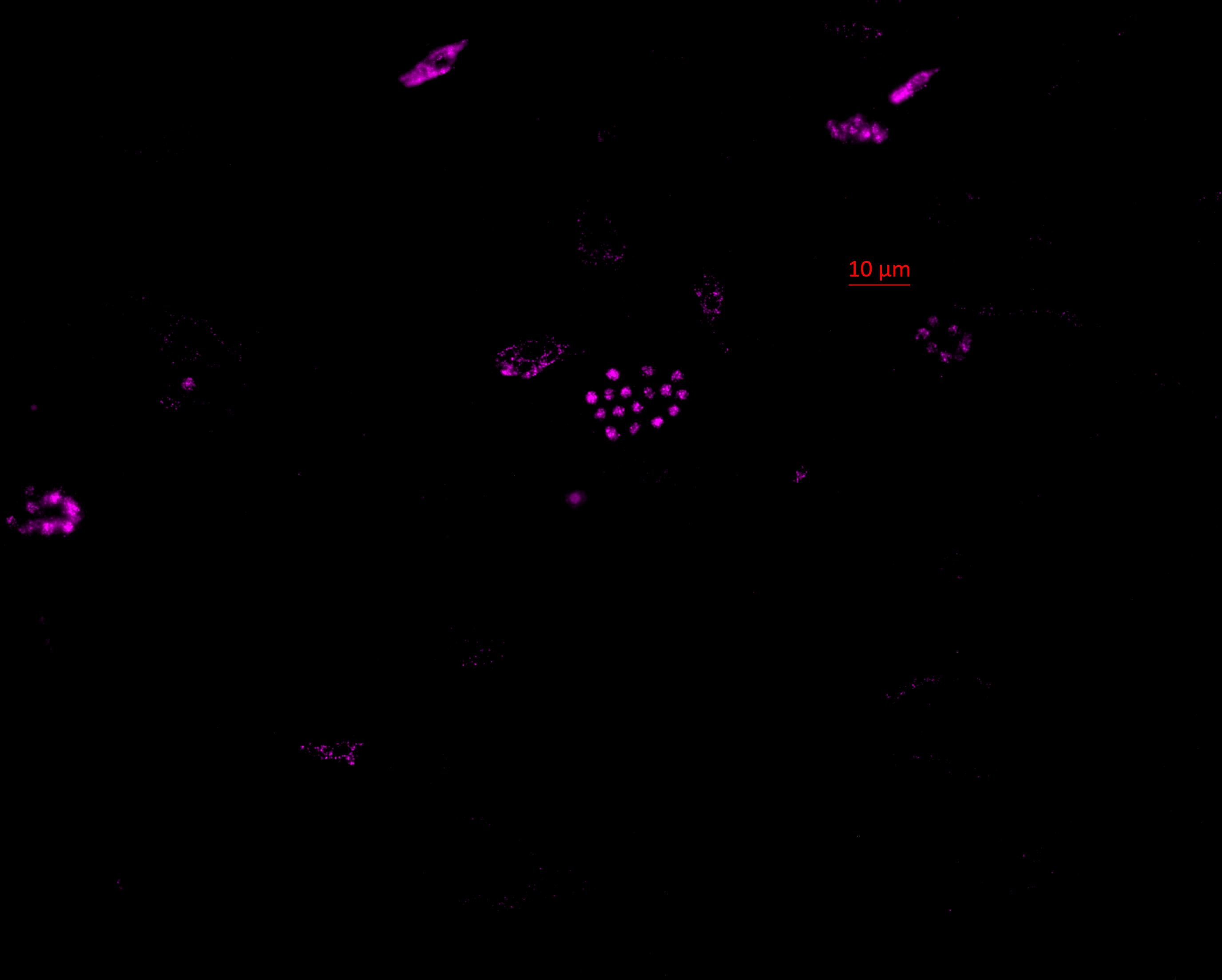

Supplement: Supplementary file 9 — Source data Fig. 3 [file 44321_2025_252_MOESM9_ESM.zip › Figure 3 Source Data/3b/Shield (72h)/Snap-1645_c3 (BFD1-Ty in red).tif]

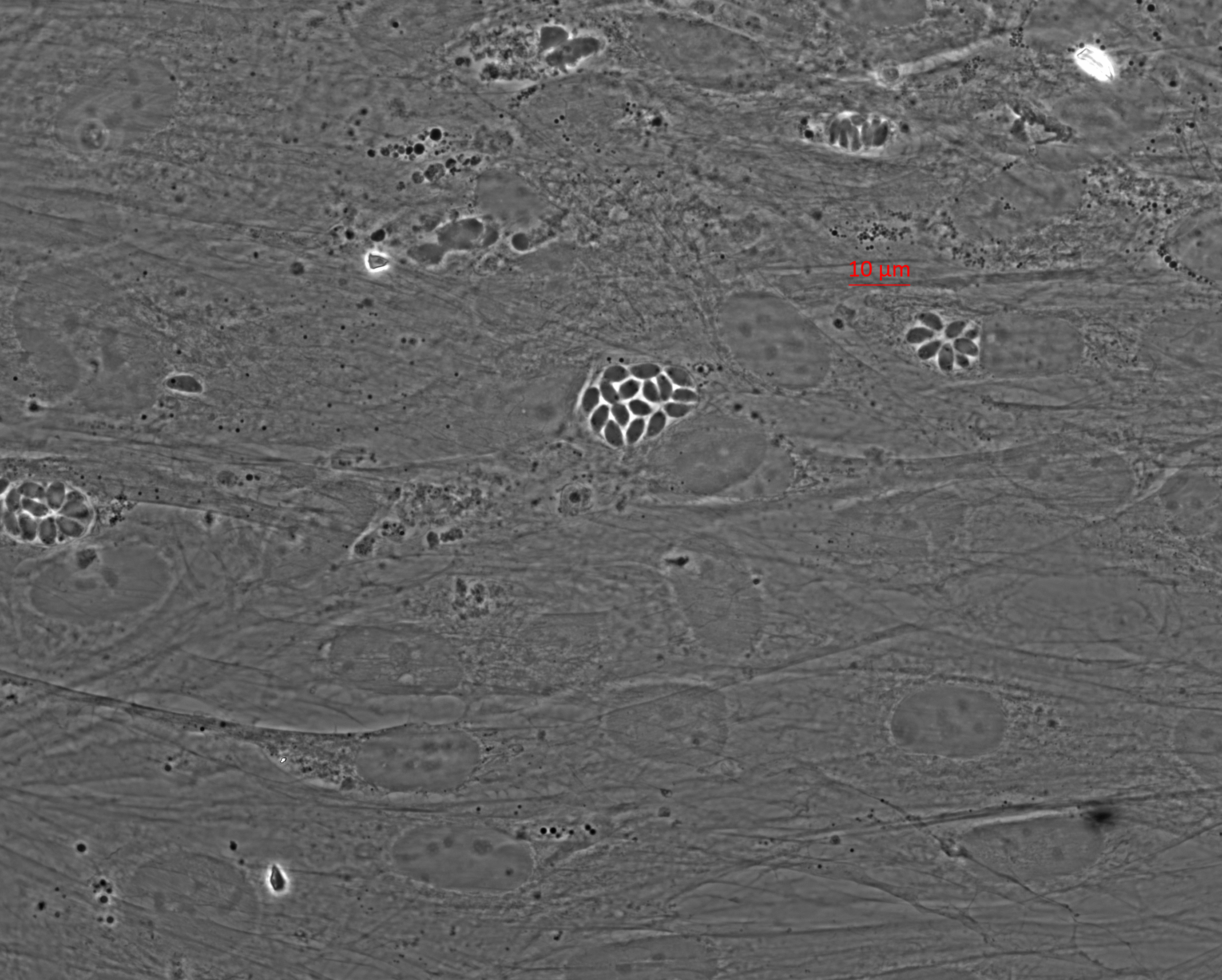

Supplement: Supplementary file 9 — Source data Fig. 3 [file 44321_2025_252_MOESM9_ESM.zip › Figure 3 Source Data/3b/Shield (72h)/Snap-1645_c1 (Phase).tif]

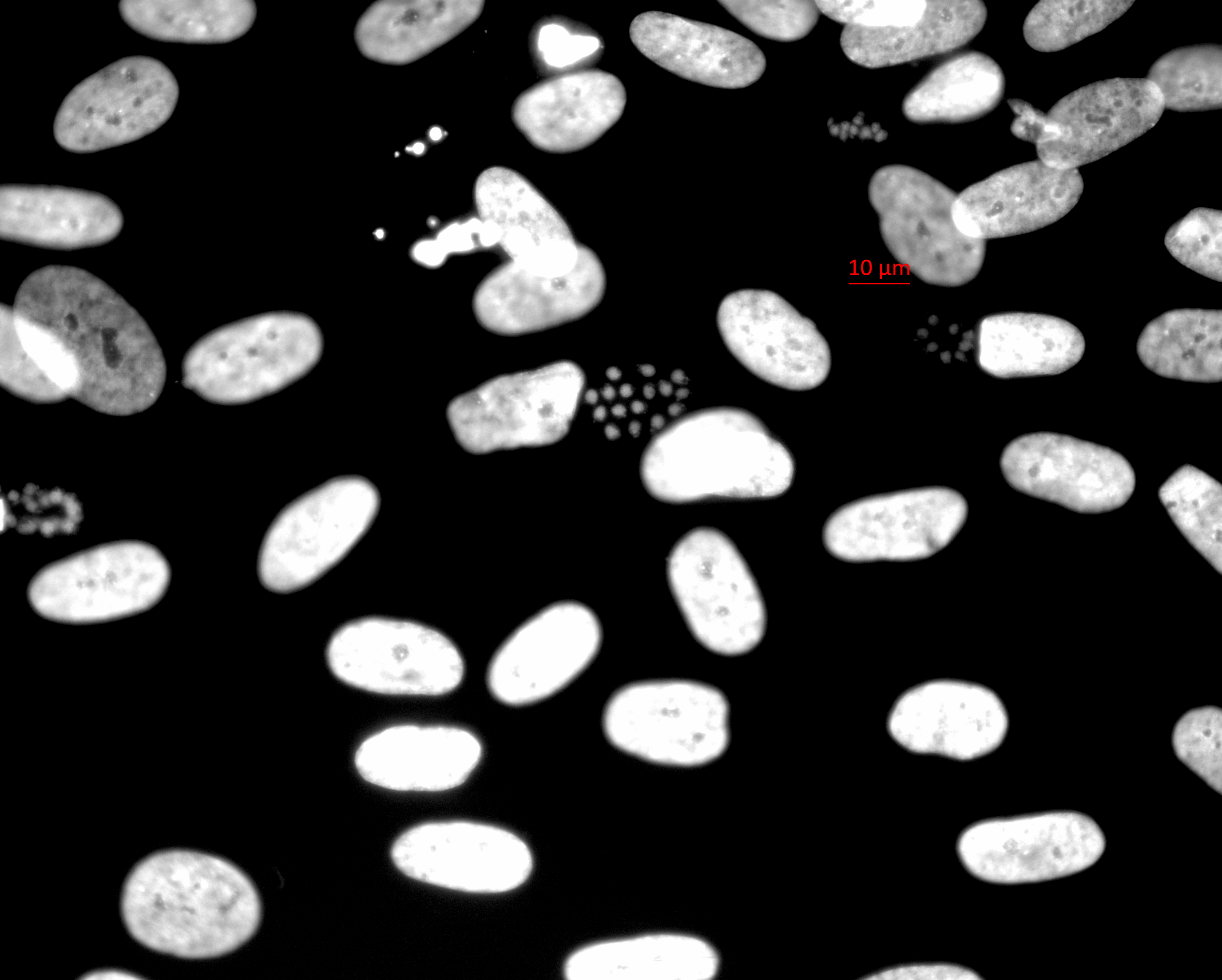

Supplement: Supplementary file 9 — Source data Fig. 3 [file 44321_2025_252_MOESM9_ESM.zip › Figure 3 Source Data/3b/Shield (72h)/Snap-1645_c2 (DNA).tif]

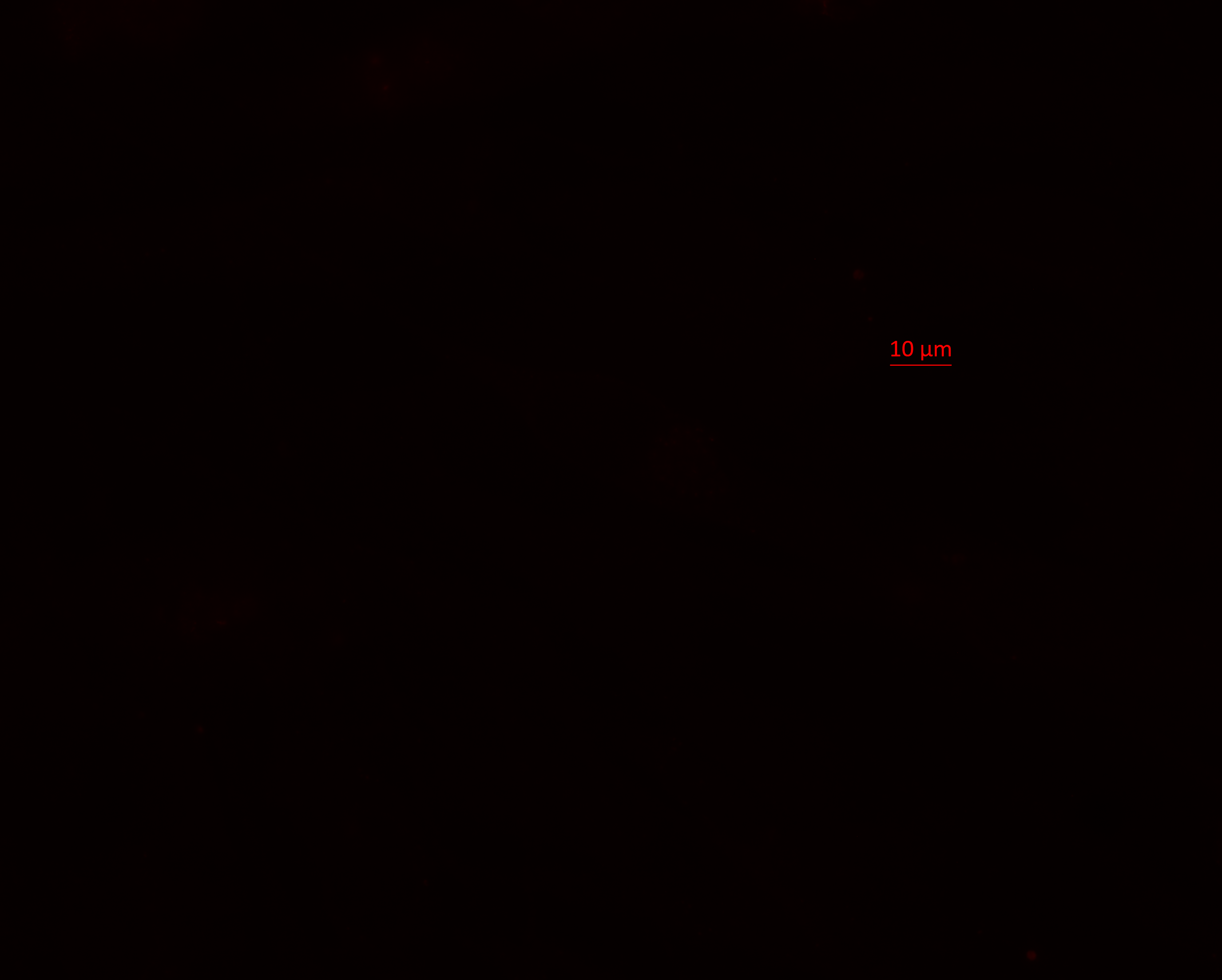

Supplement: Supplementary file 9 — Source data Fig. 3 [file 44321_2025_252_MOESM9_ESM.zip › Figure 3 Source Data/3c/BAG1 (green) - DBA (red)/UT/Snap-1735_c4 (DBA).tif]

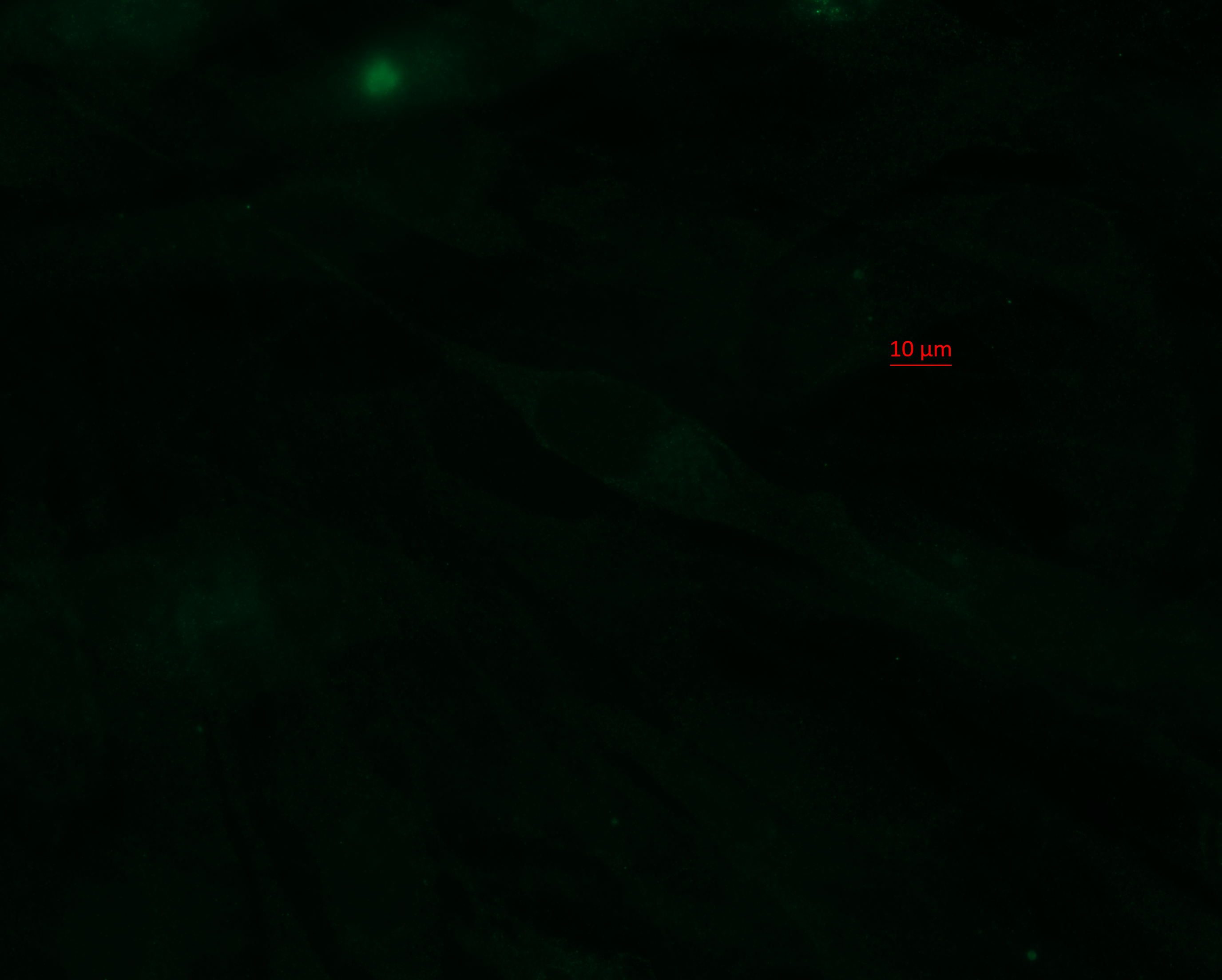

Supplement: Supplementary file 9 — Source data Fig. 3 [file 44321_2025_252_MOESM9_ESM.zip › Figure 3 Source Data/3c/BAG1 (green) - DBA (red)/UT/Snap-1735_c3 (BAG1).tif]

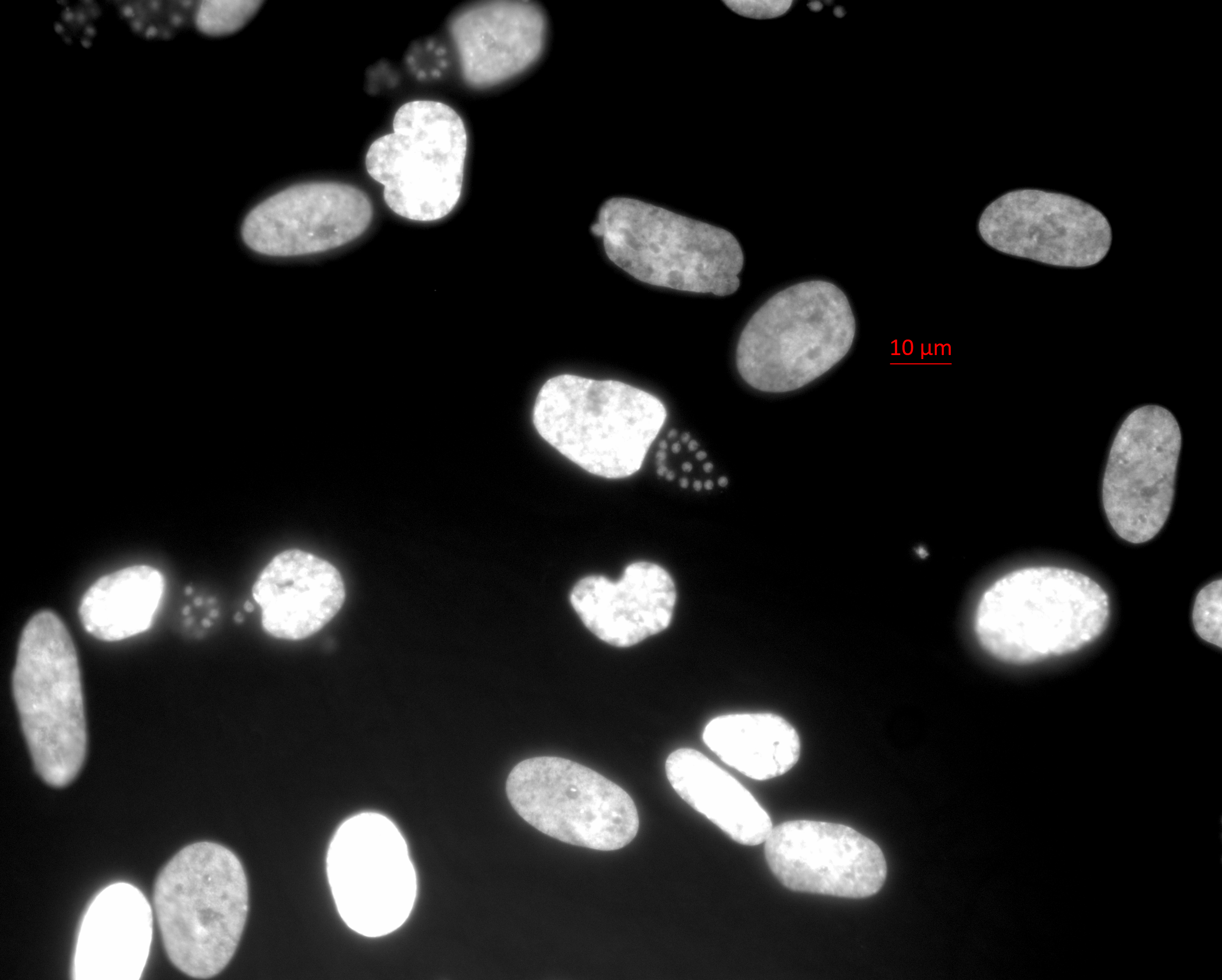

Supplement: Supplementary file 9 — Source data Fig. 3 [file 44321_2025_252_MOESM9_ESM.zip › Figure 3 Source Data/3c/BAG1 (green) - DBA (red)/UT/Snap-1735_c2 (DNA).tif]

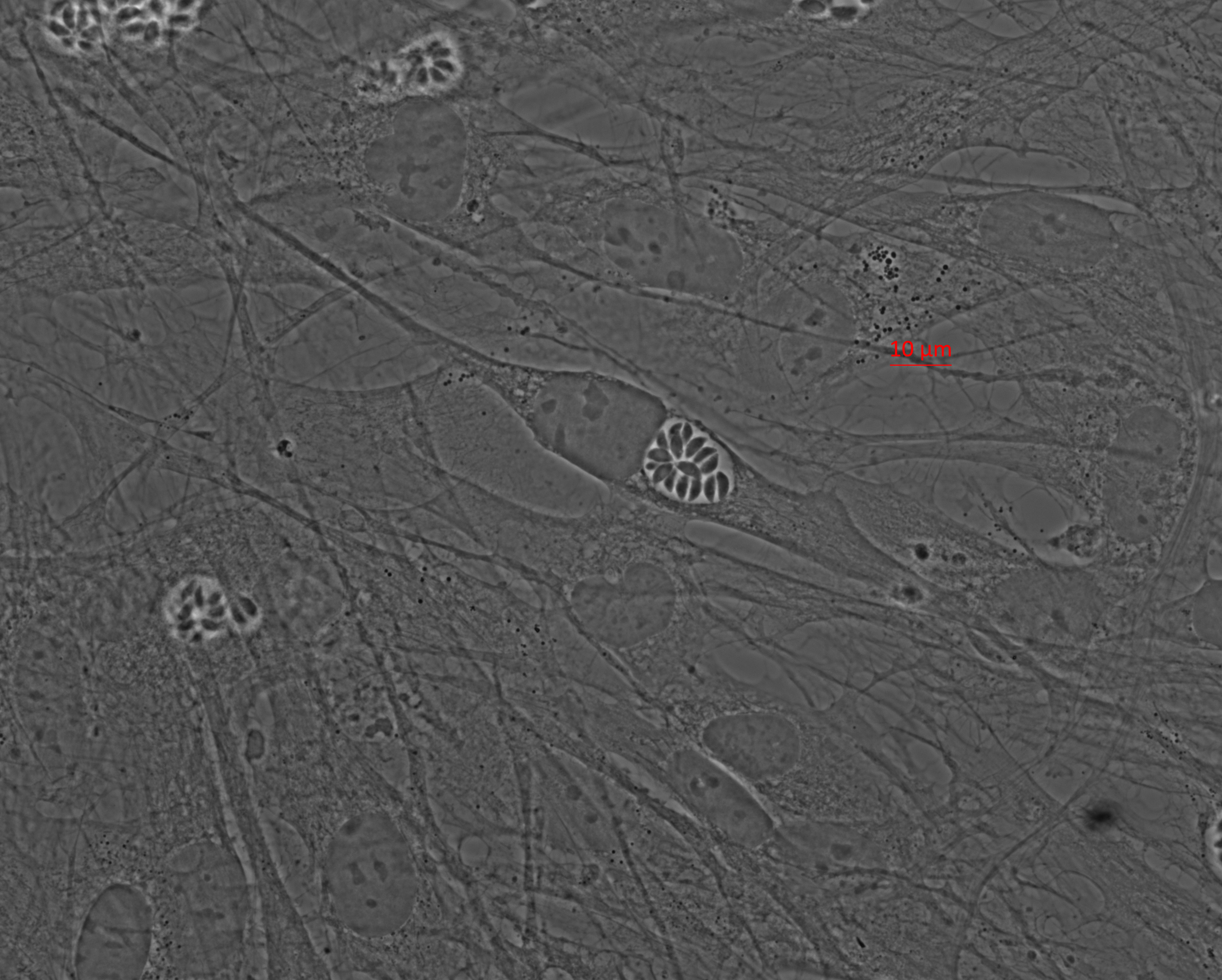

Supplement: Supplementary file 9 — Source data Fig. 3 [file 44321_2025_252_MOESM9_ESM.zip › Figure 3 Source Data/3c/BAG1 (green) - DBA (red)/UT/Snap-1735_c1 (Phase).tif]

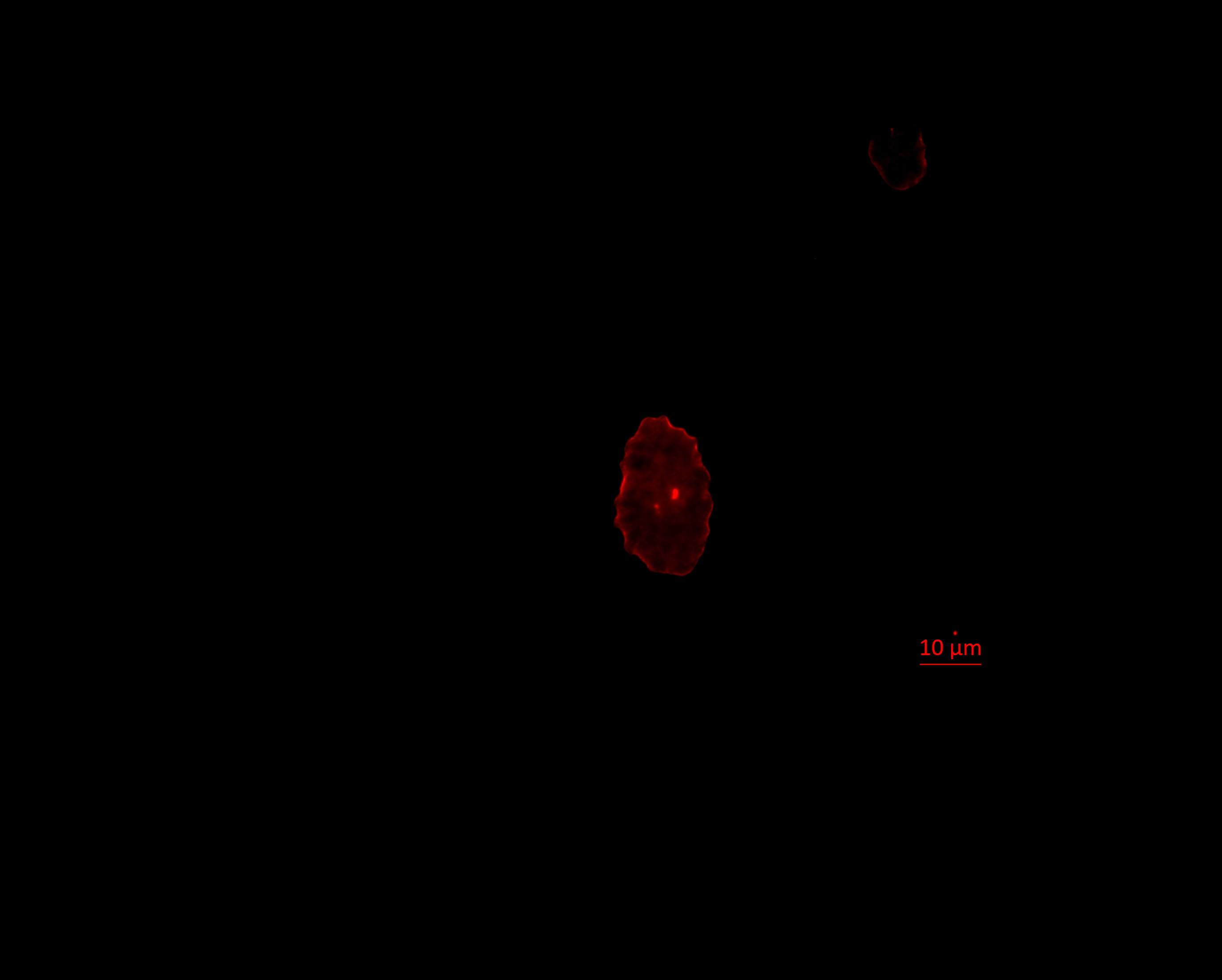

Supplement: Supplementary file 9 — Source data Fig. 3 [file 44321_2025_252_MOESM9_ESM.zip › Figure 3 Source Data/3c/BAG1 (green) - DBA (red)/Shield (72h)/Snap-1733_c4 (DBA).tif]

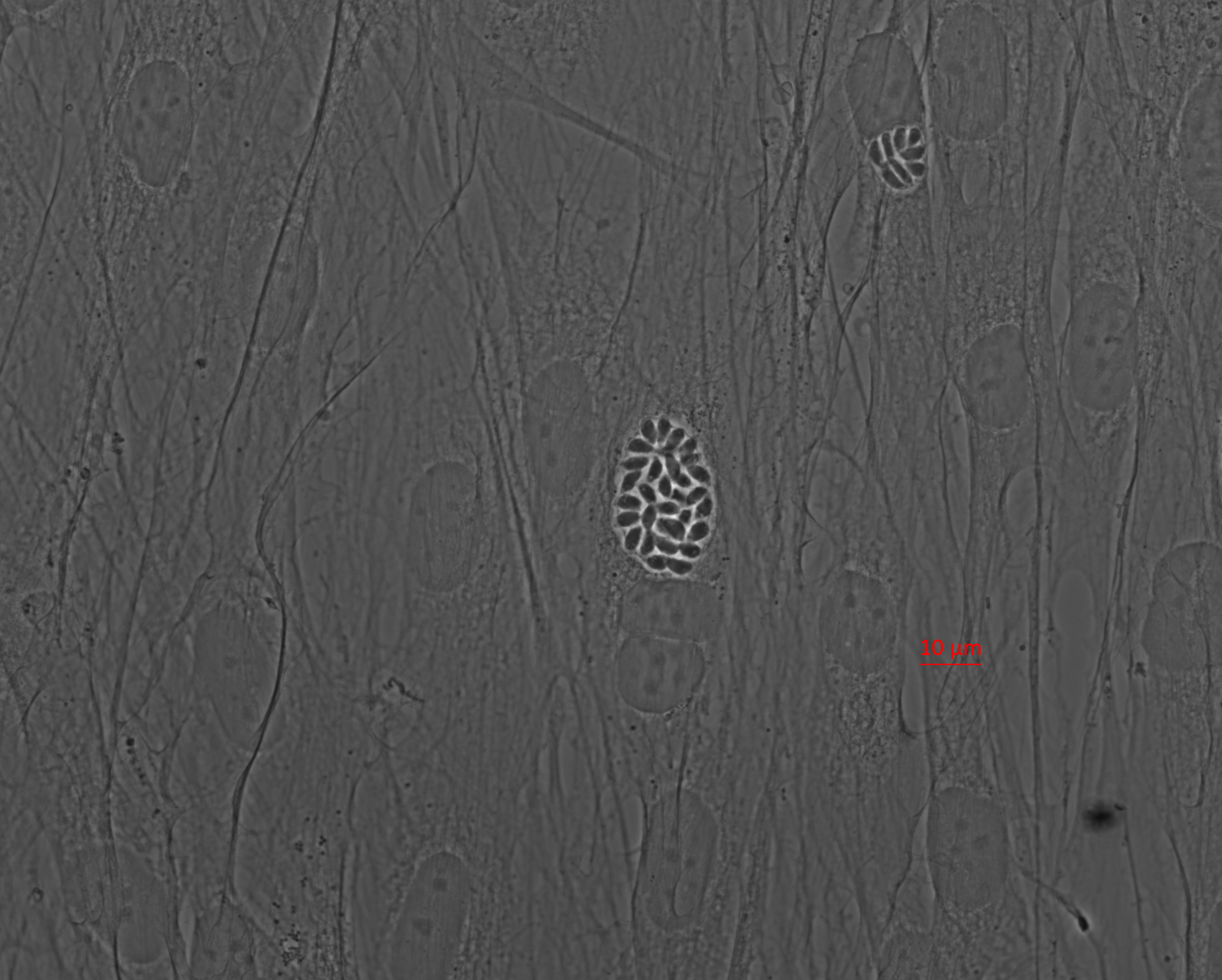

Supplement: Supplementary file 9 — Source data Fig. 3 [file 44321_2025_252_MOESM9_ESM.zip › Figure 3 Source Data/3c/BAG1 (green) - DBA (red)/Shield (72h)/Snap-1733_c1 (Phase).tif]

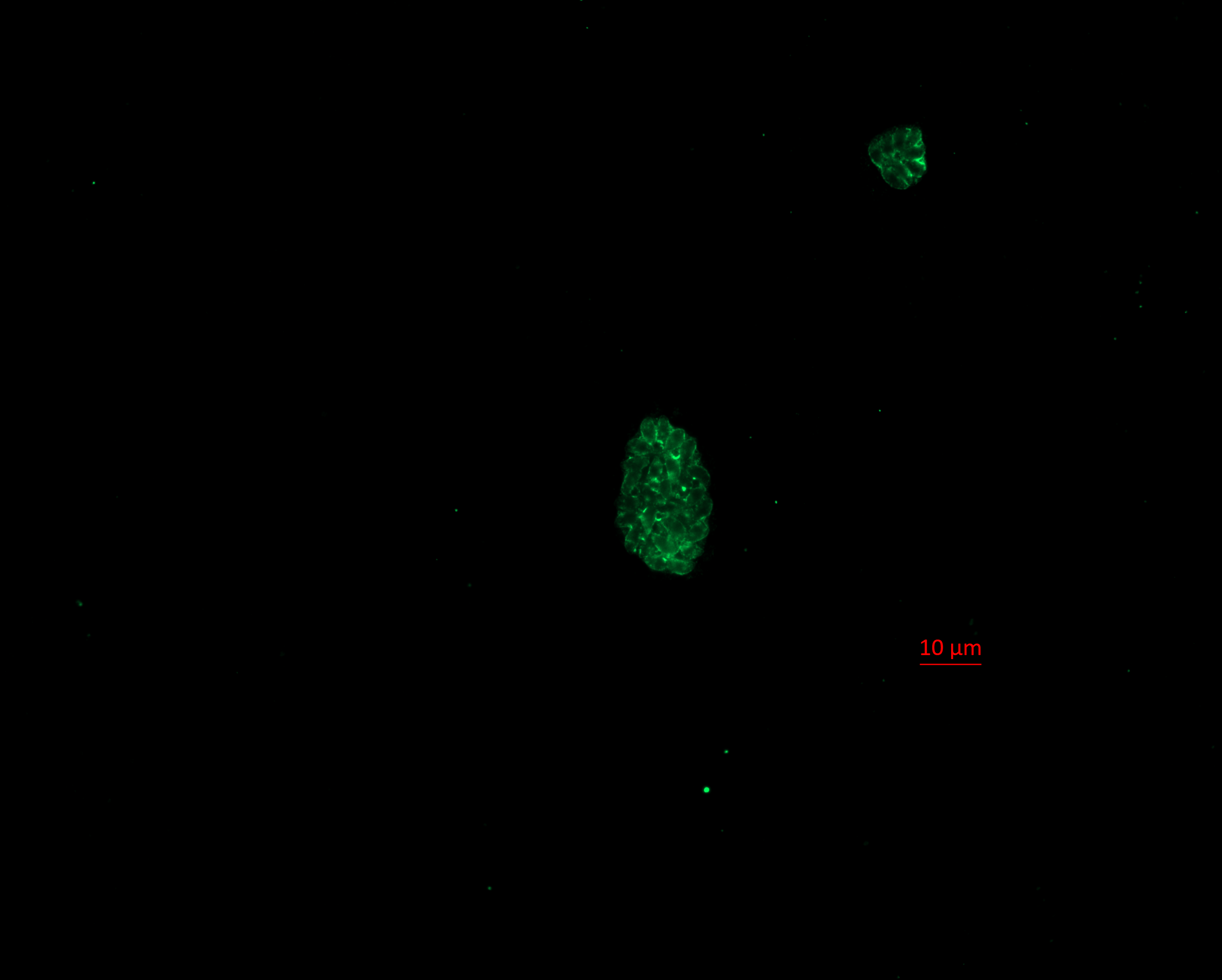

Supplement: Supplementary file 9 — Source data Fig. 3 [file 44321_2025_252_MOESM9_ESM.zip › Figure 3 Source Data/3c/BAG1 (green) - DBA (red)/Shield (72h)/Snap-1733_c3 (BAG1).tif]

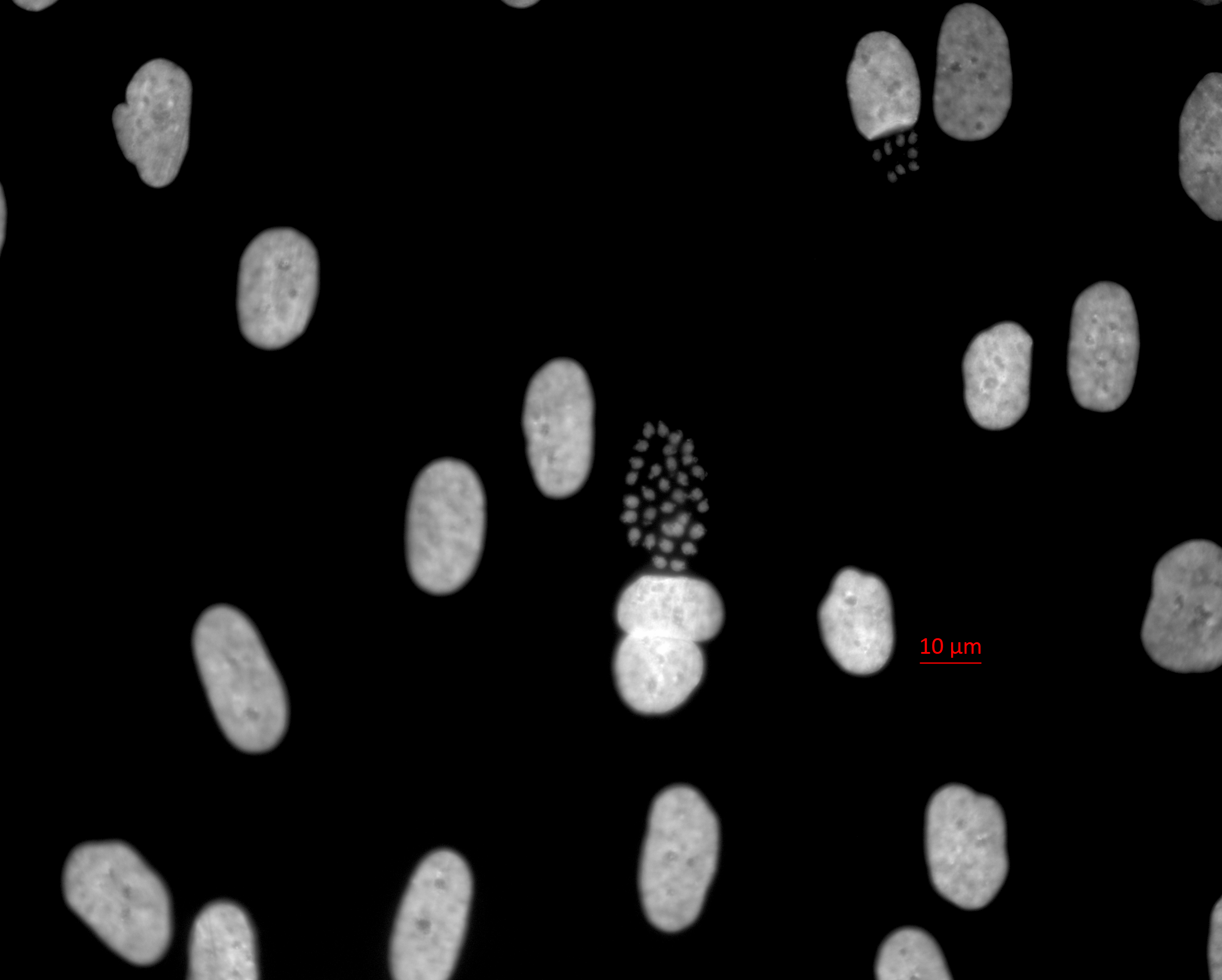

Supplement: Supplementary file 9 — Source data Fig. 3 [file 44321_2025_252_MOESM9_ESM.zip › Figure 3 Source Data/3c/BAG1 (green) - DBA (red)/Shield (72h)/Snap-1733_c2 (DNA).tif]

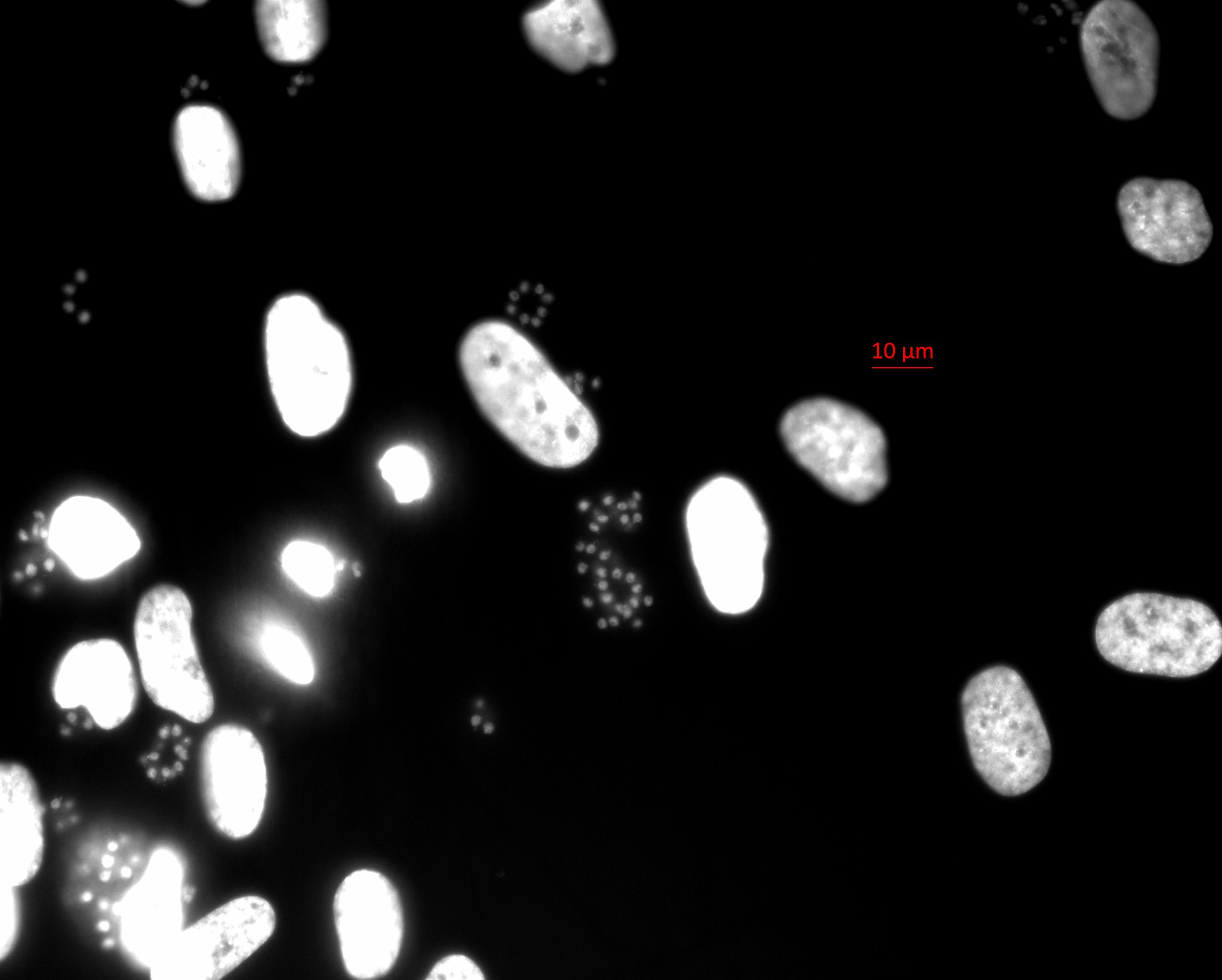

Supplement: Supplementary file 9 — Source data Fig. 3 [file 44321_2025_252_MOESM9_ESM.zip › Figure 3 Source Data/3c/BSM (green) - DBA (red)/UT/Snap-1763_c2 (DNA).tif]

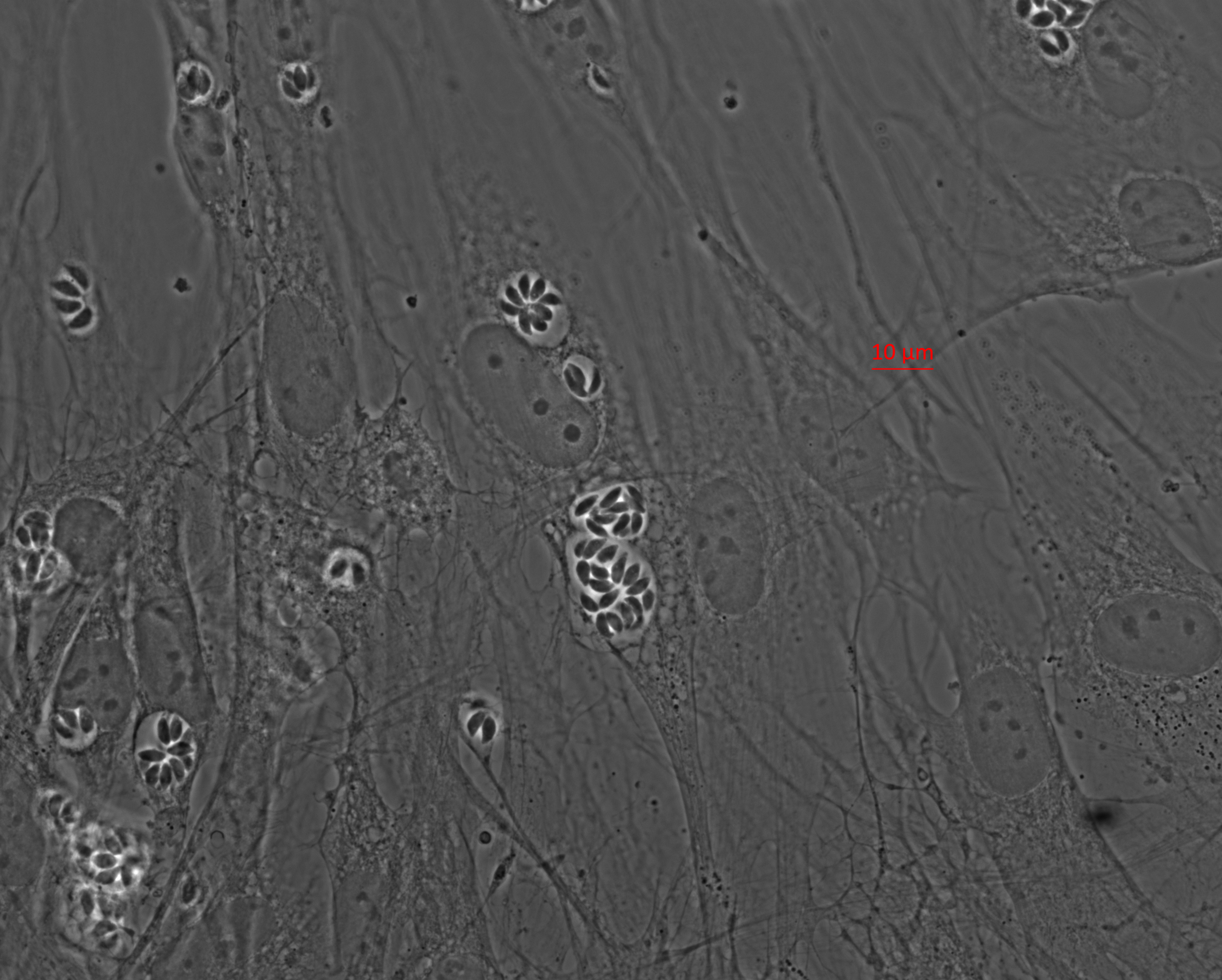

Supplement: Supplementary file 9 — Source data Fig. 3 [file 44321_2025_252_MOESM9_ESM.zip › Figure 3 Source Data/3c/BSM (green) - DBA (red)/UT/Snap-1763_c1 (Phase).tif]

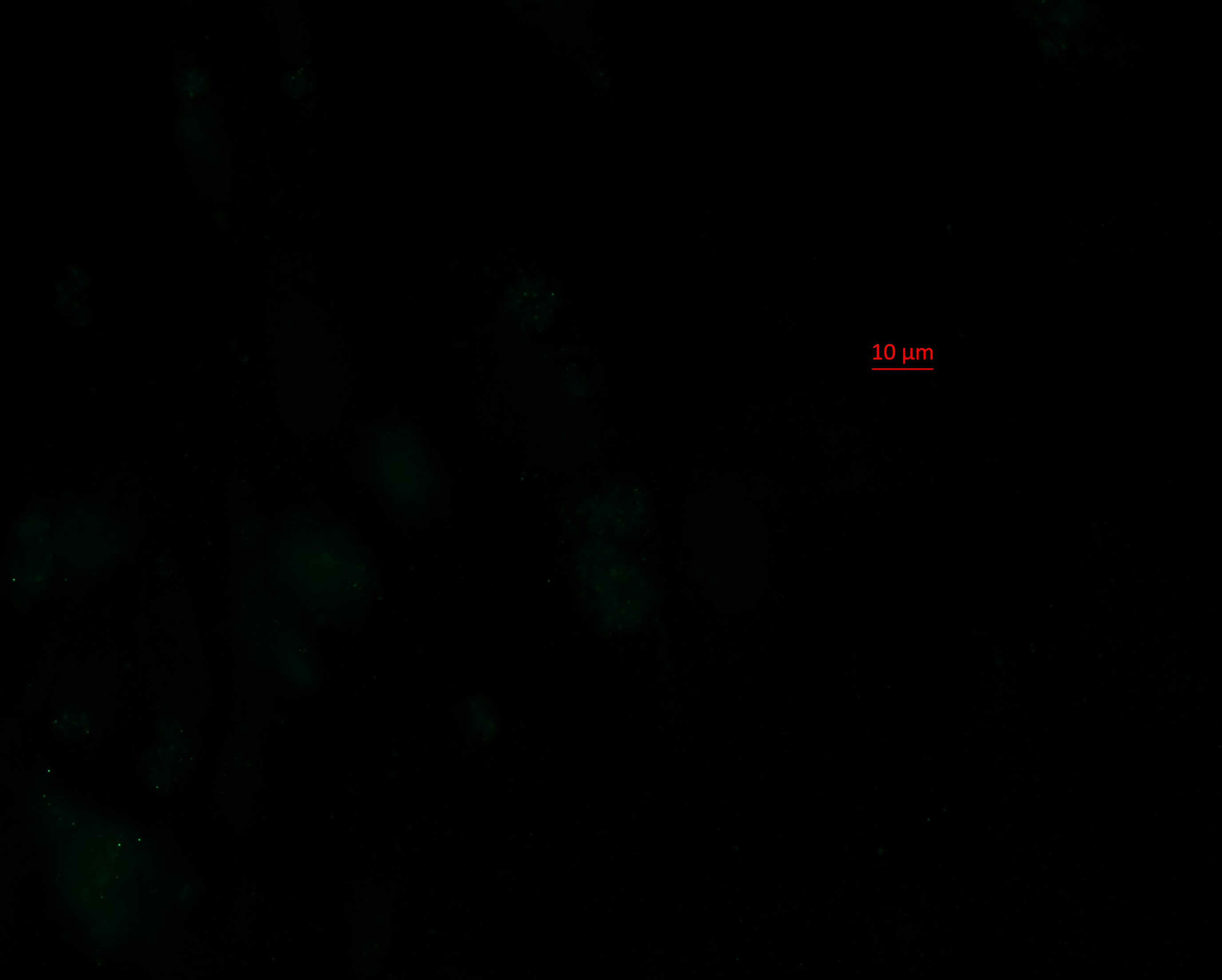

Supplement: Supplementary file 9 — Source data Fig. 3 [file 44321_2025_252_MOESM9_ESM.zip › Figure 3 Source Data/3c/BSM (green) - DBA (red)/UT/Snap-1763_c3 (BSM).tif]

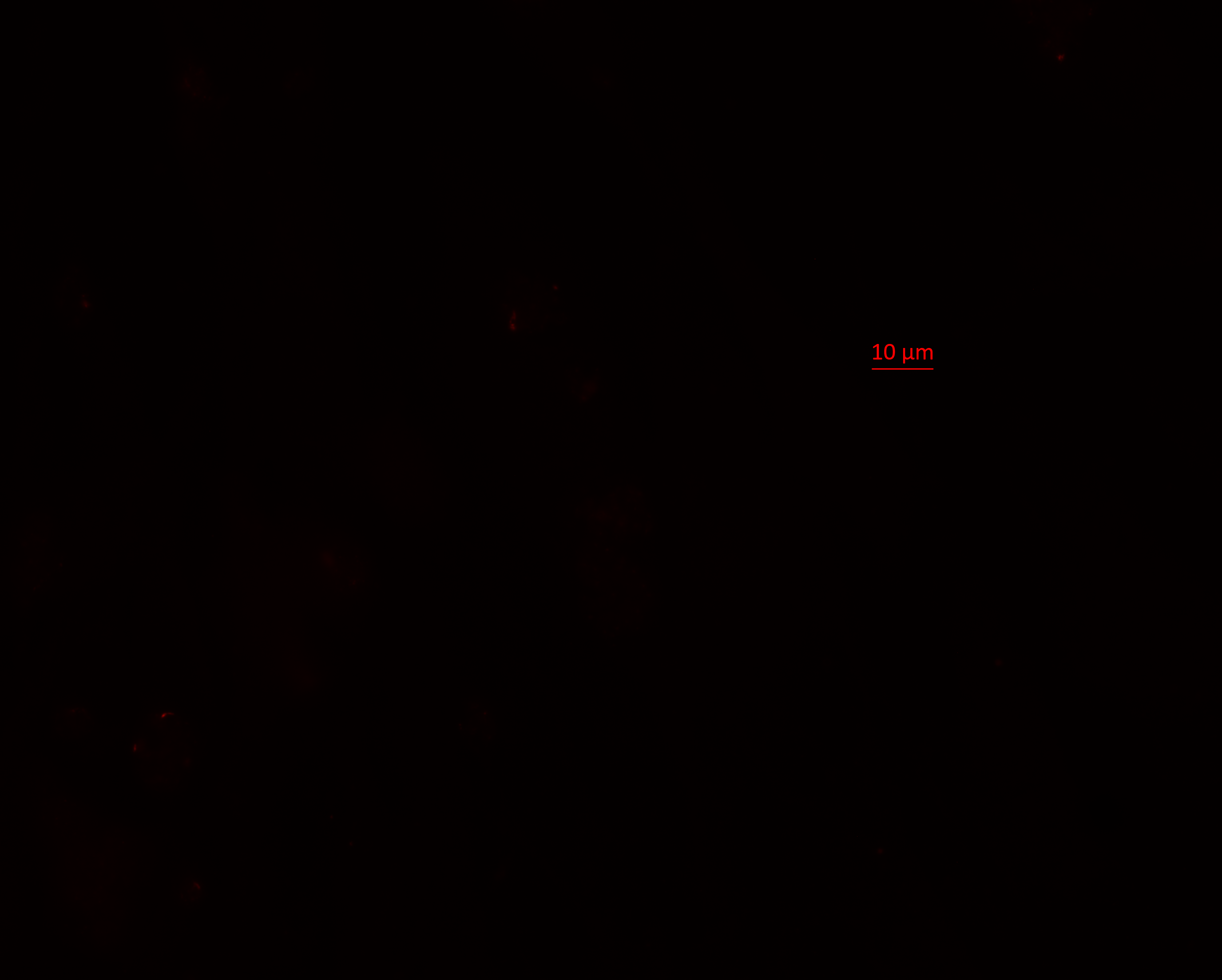

Supplement: Supplementary file 9 — Source data Fig. 3 [file 44321_2025_252_MOESM9_ESM.zip › Figure 3 Source Data/3c/BSM (green) - DBA (red)/UT/Snap-1763_c4 (DBA).tif]

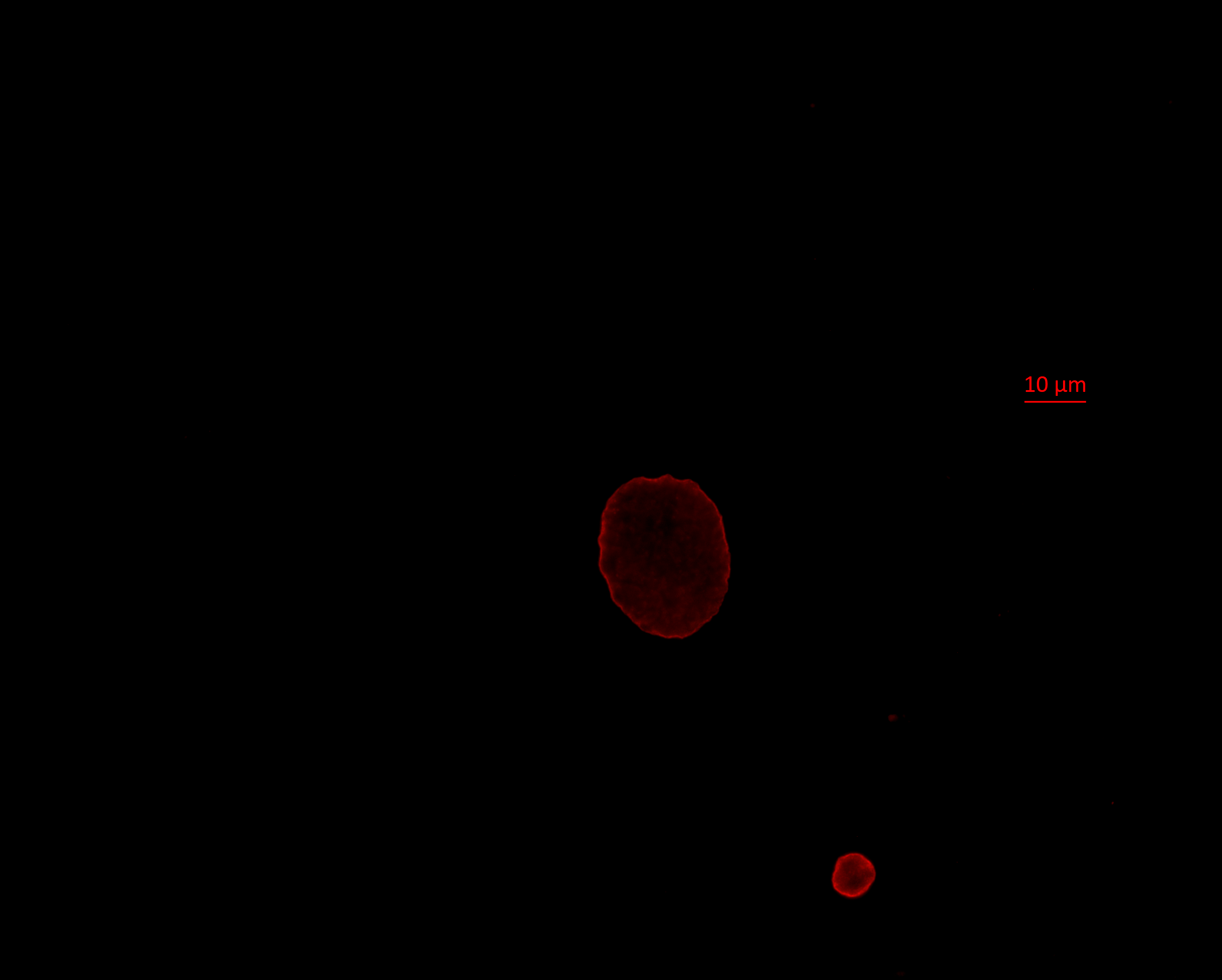

Supplement: Supplementary file 9 — Source data Fig. 3 [file 44321_2025_252_MOESM9_ESM.zip › Figure 3 Source Data/3c/BSM (green) - DBA (red)/Shield (72h)/Snap-1772_c4 (DBA).tif]

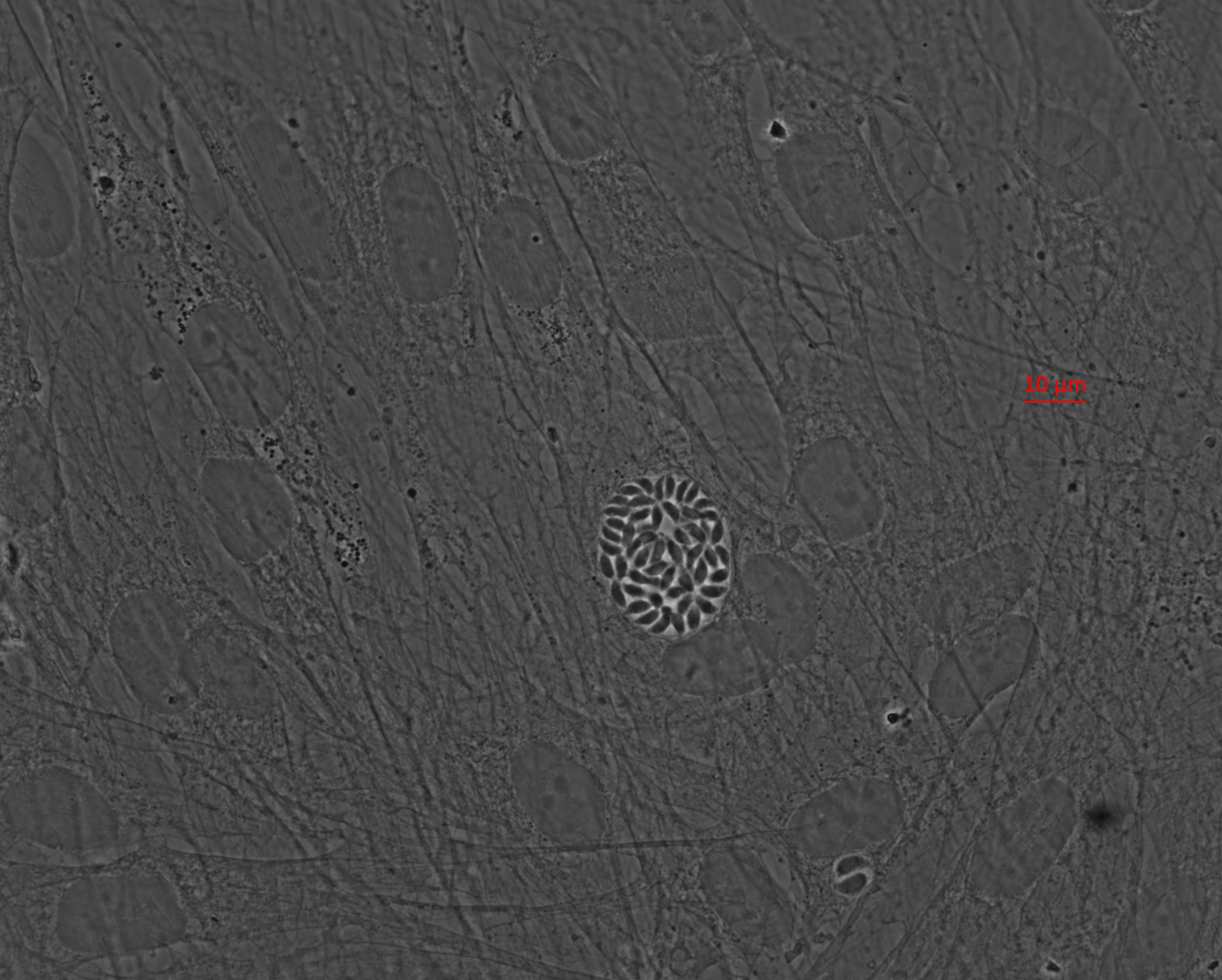

Supplement: Supplementary file 9 — Source data Fig. 3 [file 44321_2025_252_MOESM9_ESM.zip › Figure 3 Source Data/3c/BSM (green) - DBA (red)/Shield (72h)/Snap-1772_c1 (Phase).tif]

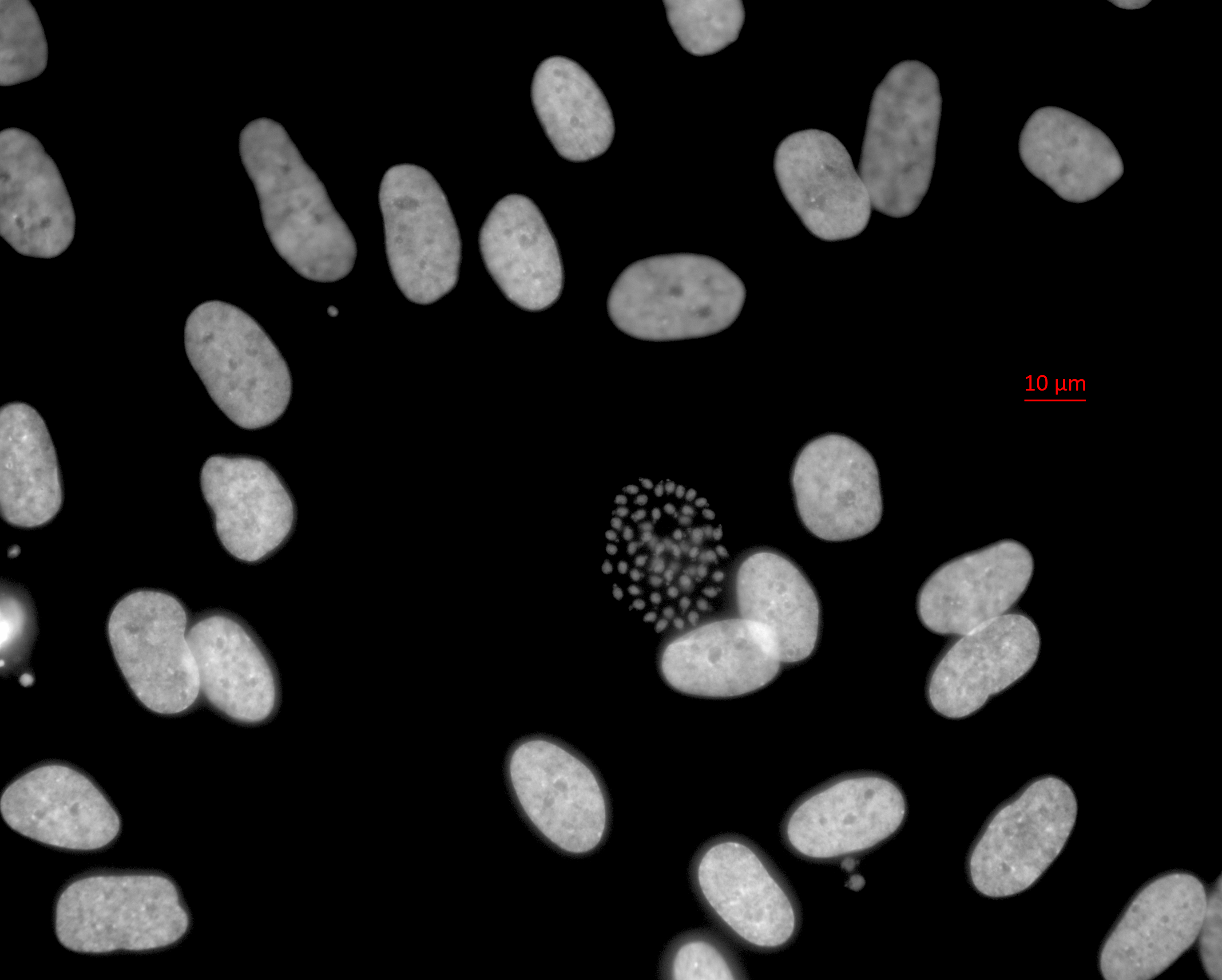

Supplement: Supplementary file 9 — Source data Fig. 3 [file 44321_2025_252_MOESM9_ESM.zip › Figure 3 Source Data/3c/BSM (green) - DBA (red)/Shield (72h)/Snap-1772_c2 (DNA).tif]

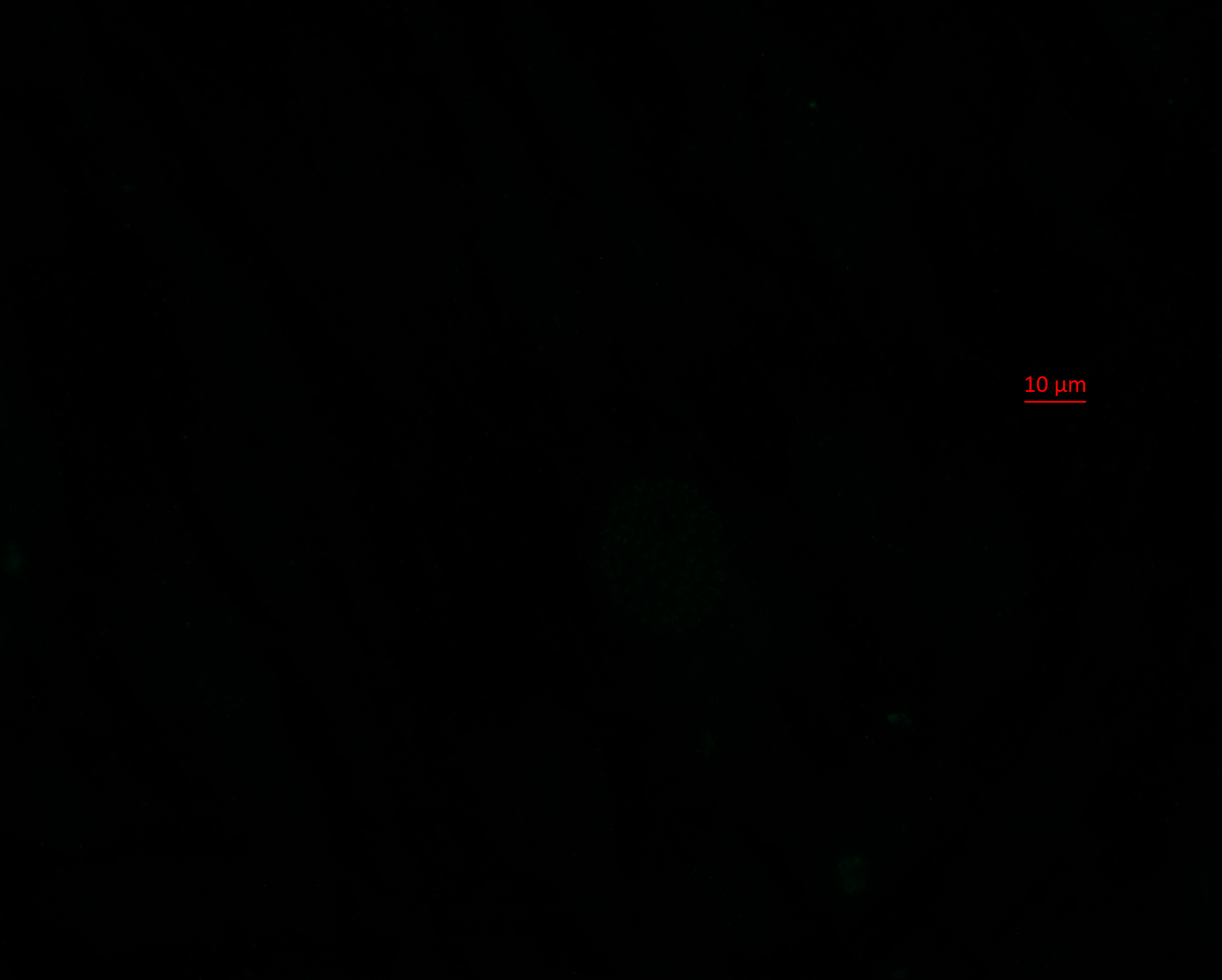

Supplement: Supplementary file 9 — Source data Fig. 3 [file 44321_2025_252_MOESM9_ESM.zip › Figure 3 Source Data/3c/BSM (green) - DBA (red)/Shield (72h)/Snap-1772_c3 (BSM).tif]

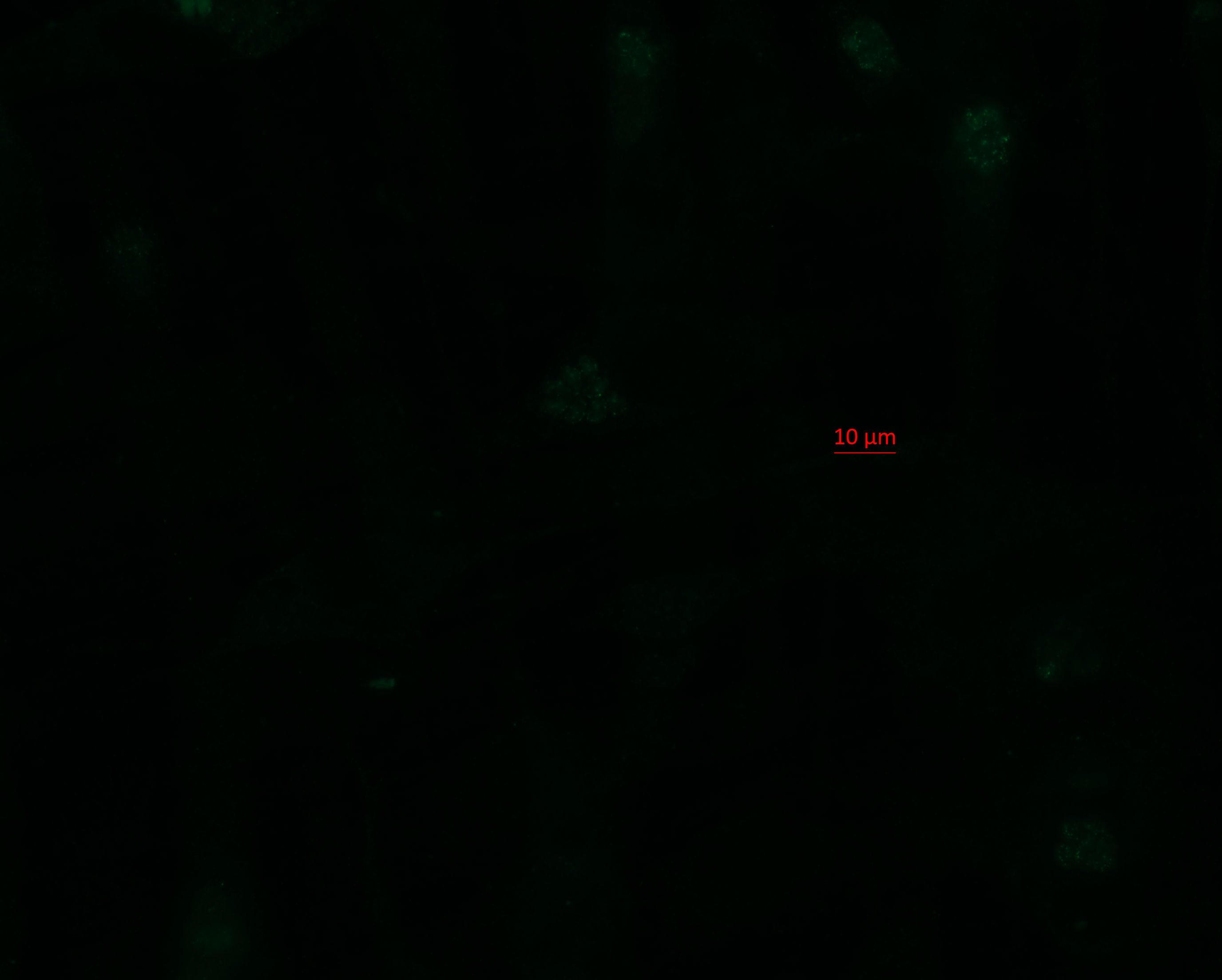

Supplement: Supplementary file 9 — Source data Fig. 3 [file 44321_2025_252_MOESM9_ESM.zip › Figure 3 Source Data/3c/SRS35A (green) - DBA (red)/UT/Snap-1776_c3 (SRS35A).tif]

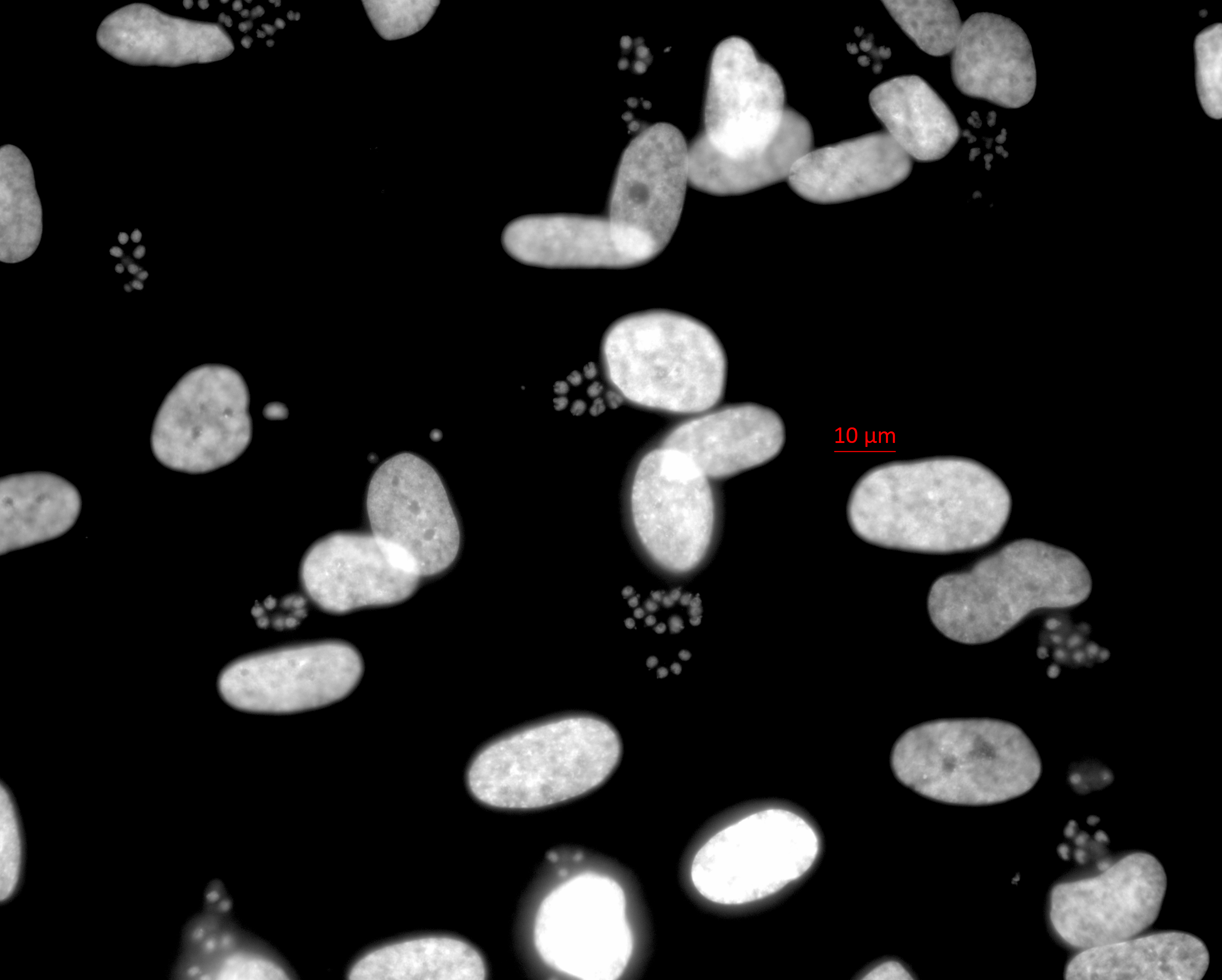

Supplement: Supplementary file 9 — Source data Fig. 3 [file 44321_2025_252_MOESM9_ESM.zip › Figure 3 Source Data/3c/SRS35A (green) - DBA (red)/UT/Snap-1776_c2 (DNA).tif]

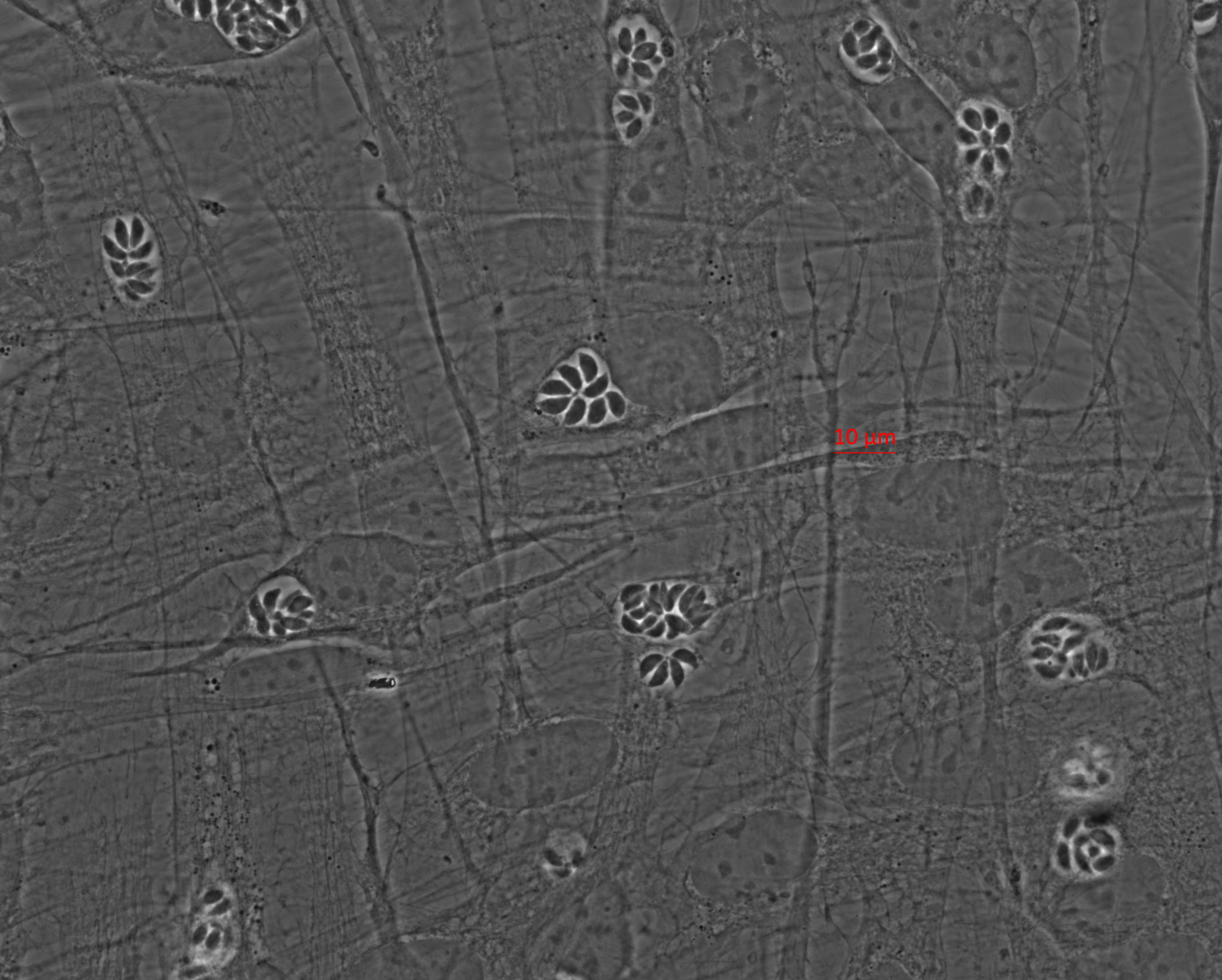

Supplement: Supplementary file 9 — Source data Fig. 3 [file 44321_2025_252_MOESM9_ESM.zip › Figure 3 Source Data/3c/SRS35A (green) - DBA (red)/UT/Snap-1776_c1 (Phase).tif]

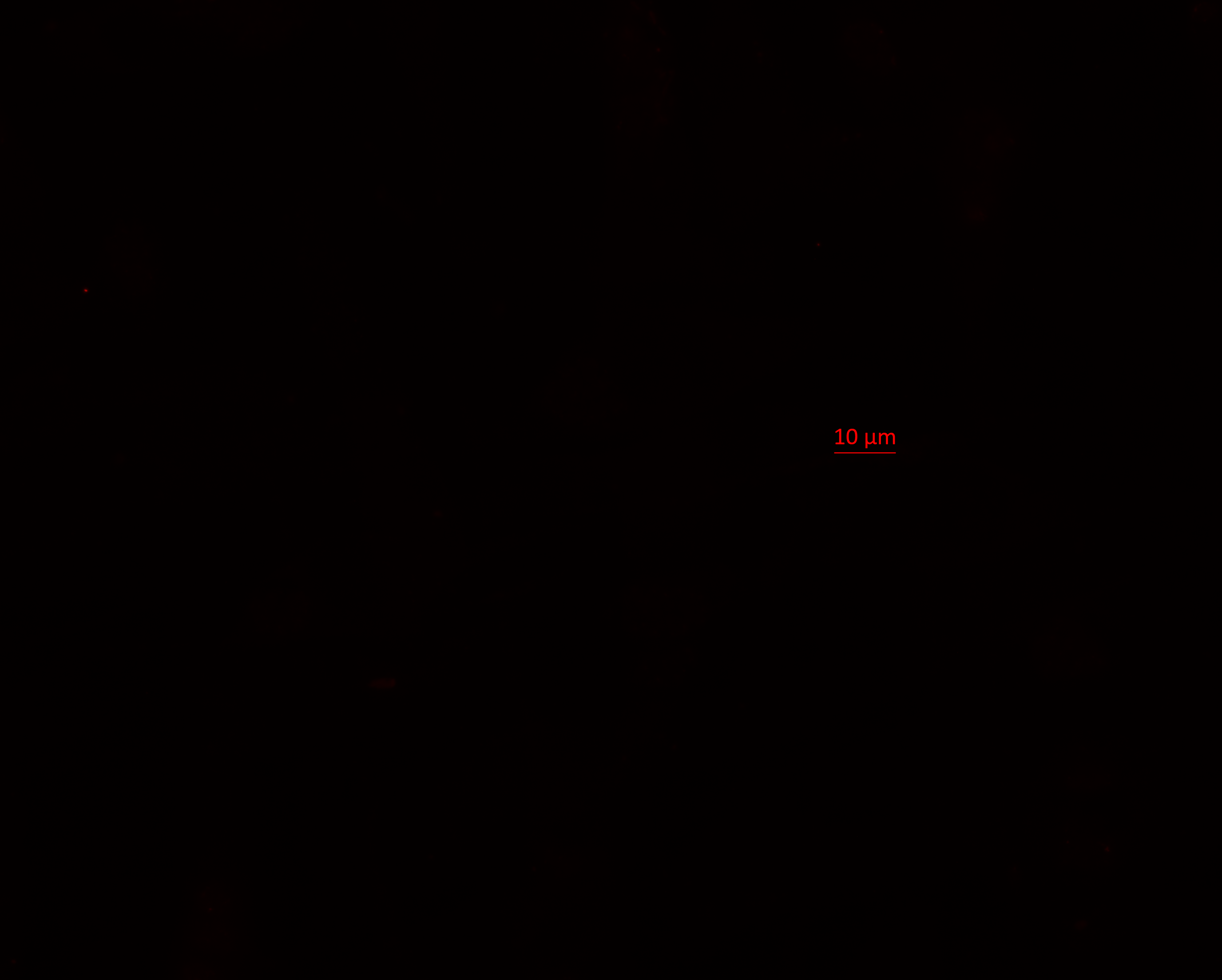

Supplement: Supplementary file 9 — Source data Fig. 3 [file 44321_2025_252_MOESM9_ESM.zip › Figure 3 Source Data/3c/SRS35A (green) - DBA (red)/UT/Snap-1776_c4 (DBA).tif]

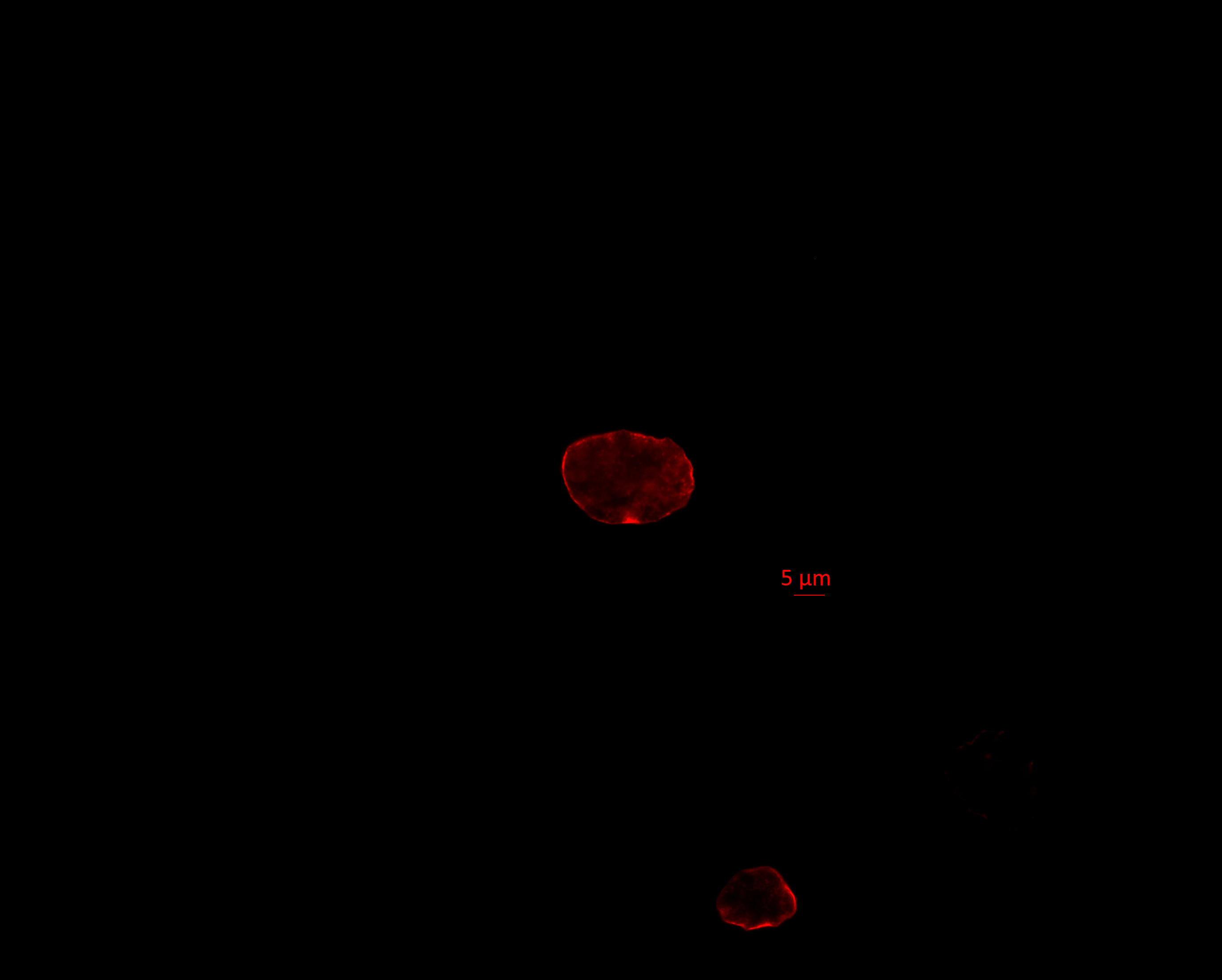

Supplement: Supplementary file 9 — Source data Fig. 3 [file 44321_2025_252_MOESM9_ESM.zip › Figure 3 Source Data/3c/SRS35A (green) - DBA (red)/Shield (72h)/Snap-1757_c4 (DBA).tif]

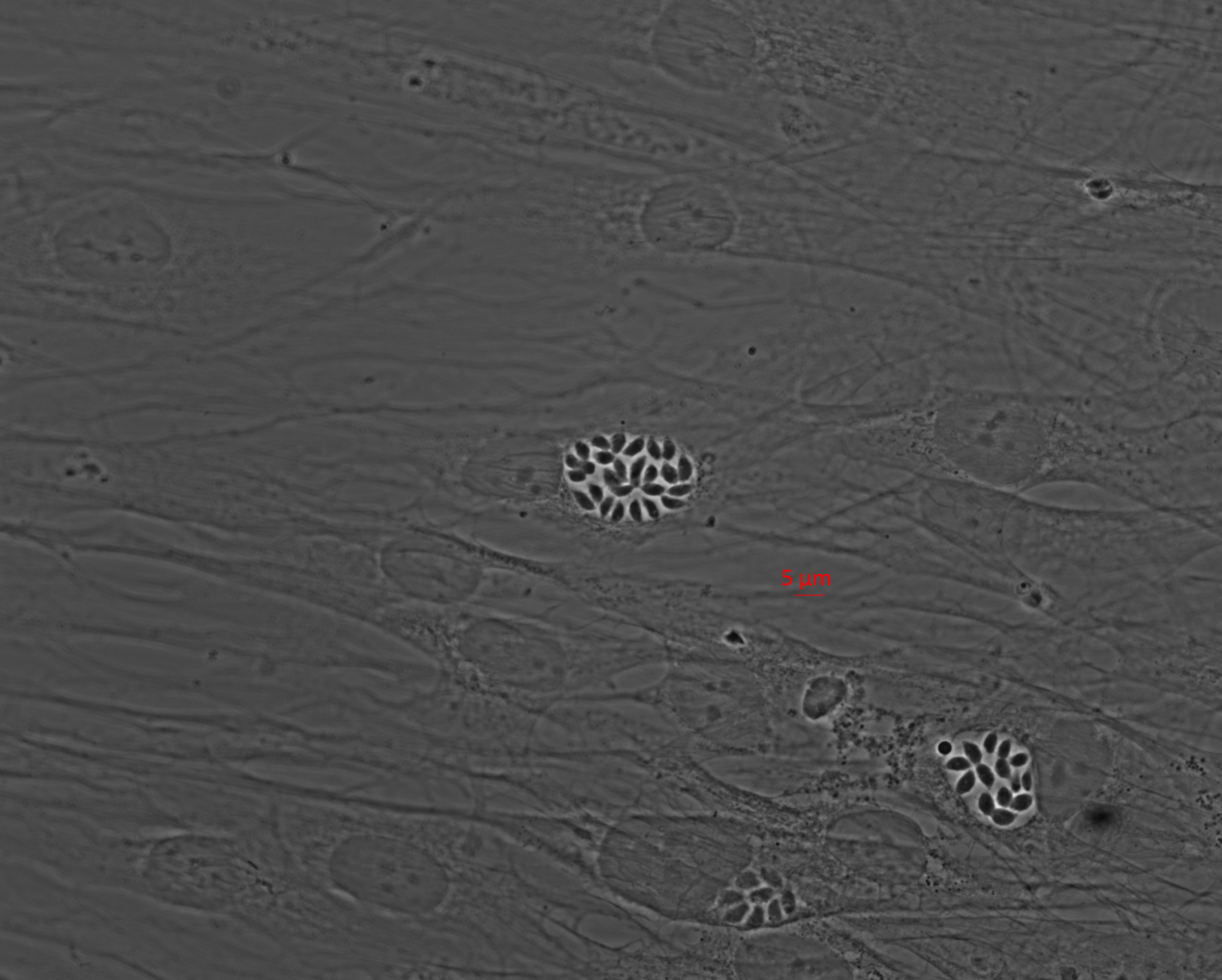

Supplement: Supplementary file 9 — Source data Fig. 3 [file 44321_2025_252_MOESM9_ESM.zip › Figure 3 Source Data/3c/SRS35A (green) - DBA (red)/Shield (72h)/Snap-1757_c1 (Phase).tif]

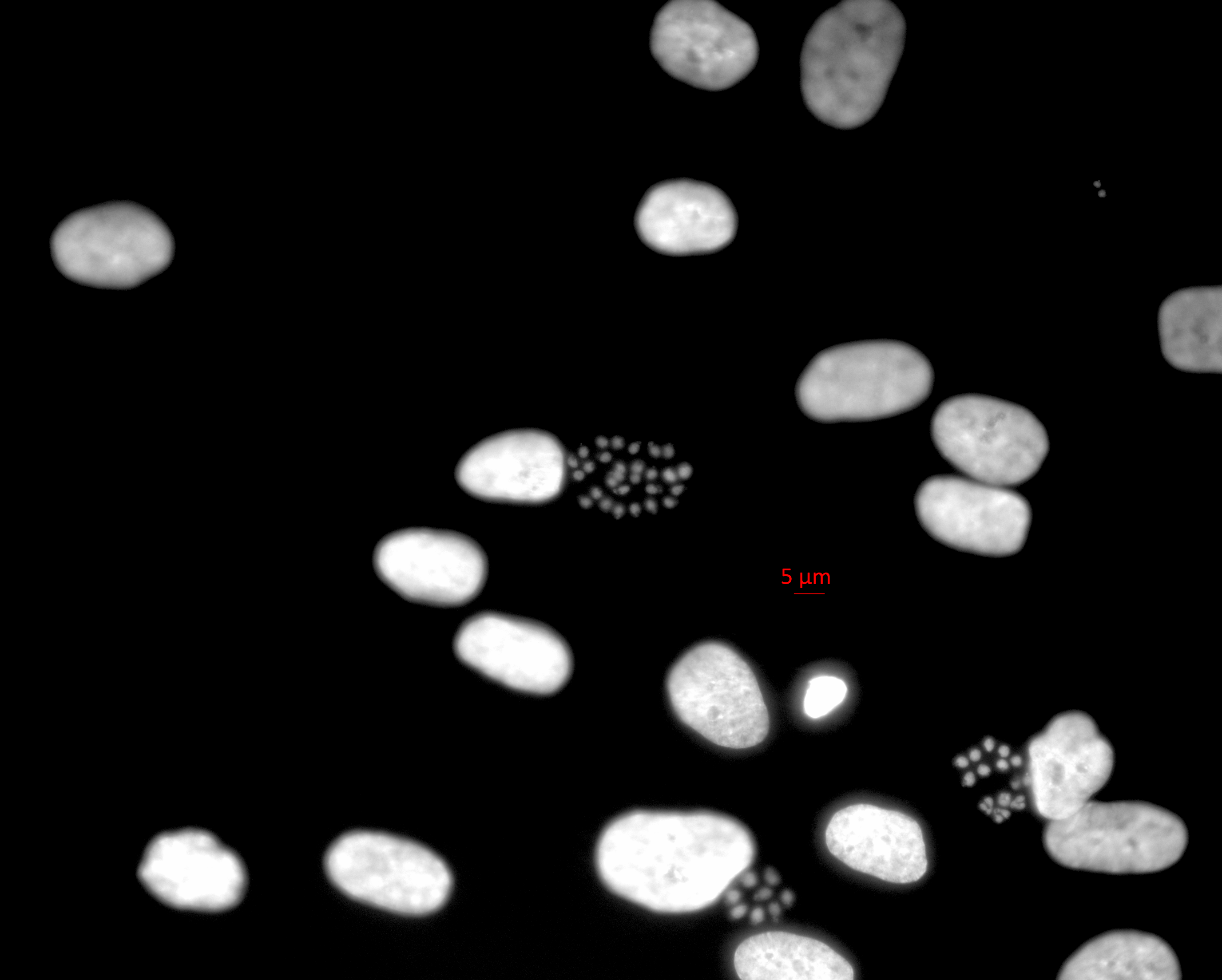

Supplement: Supplementary file 9 — Source data Fig. 3 [file 44321_2025_252_MOESM9_ESM.zip › Figure 3 Source Data/3c/SRS35A (green) - DBA (red)/Shield (72h)/Snap-1757_c2 (DNA).tif]

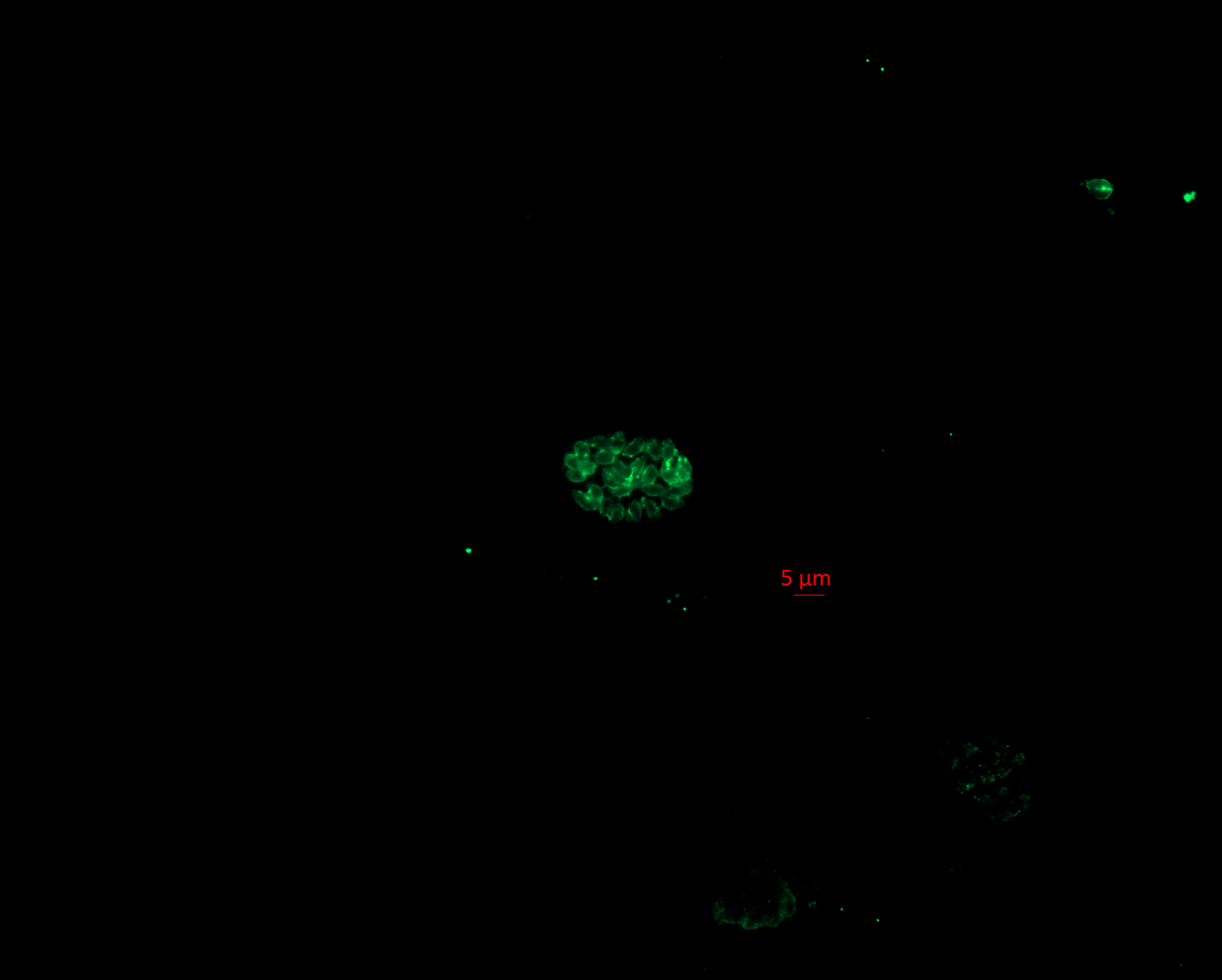

Supplement: Supplementary file 9 — Source data Fig. 3 [file 44321_2025_252_MOESM9_ESM.zip › Figure 3 Source Data/3c/SRS35A (green) - DBA (red)/Shield (72h)/Snap-1757_c3 (SRS35A).tif]

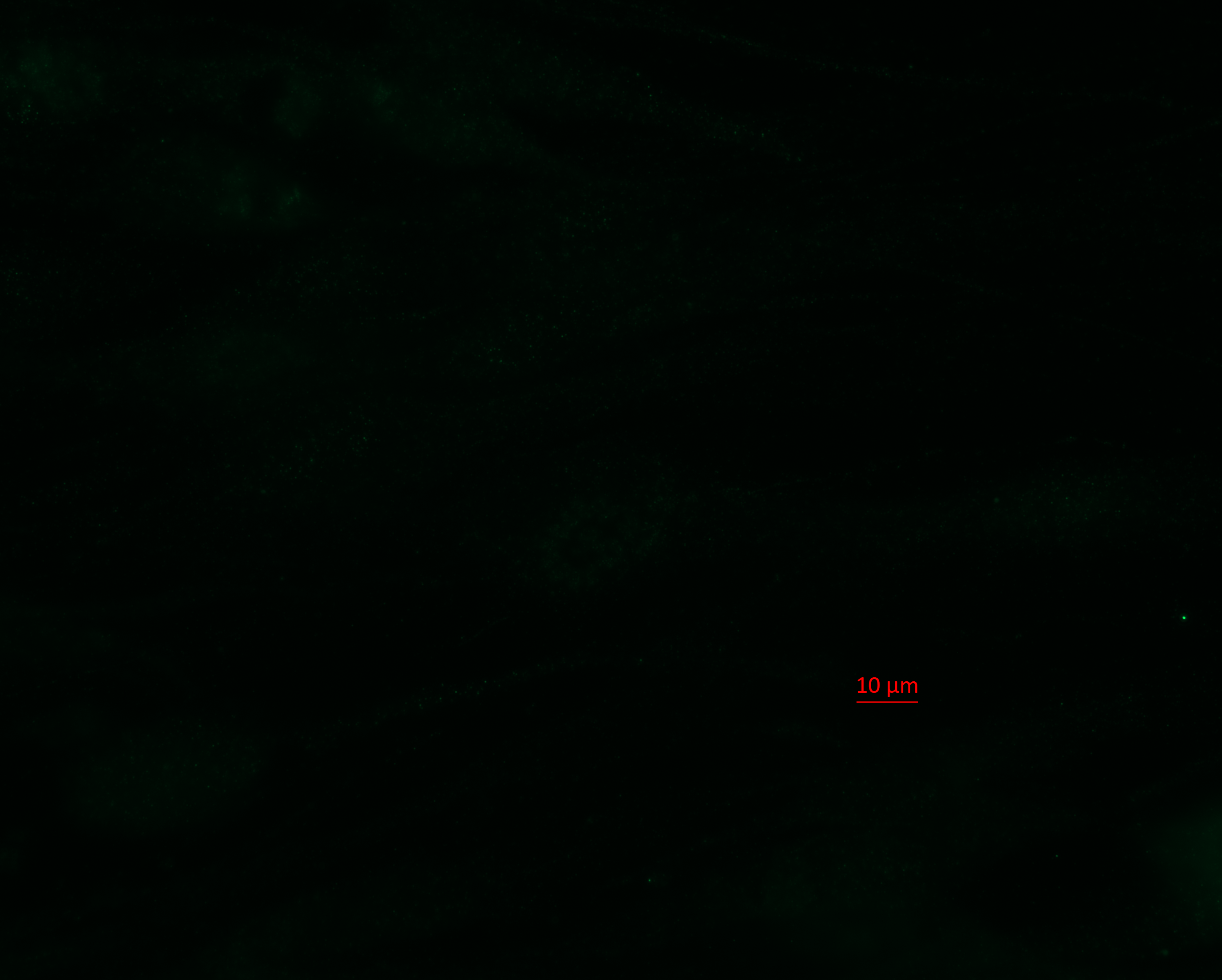

Supplement: Supplementary file 9 — Source data Fig. 3 [file 44321_2025_252_MOESM9_ESM.zip › Figure 3 Source Data/3c/BCLA (green) - DBA (red)/UT/Snap-1750_c3 (BCLA).tif]

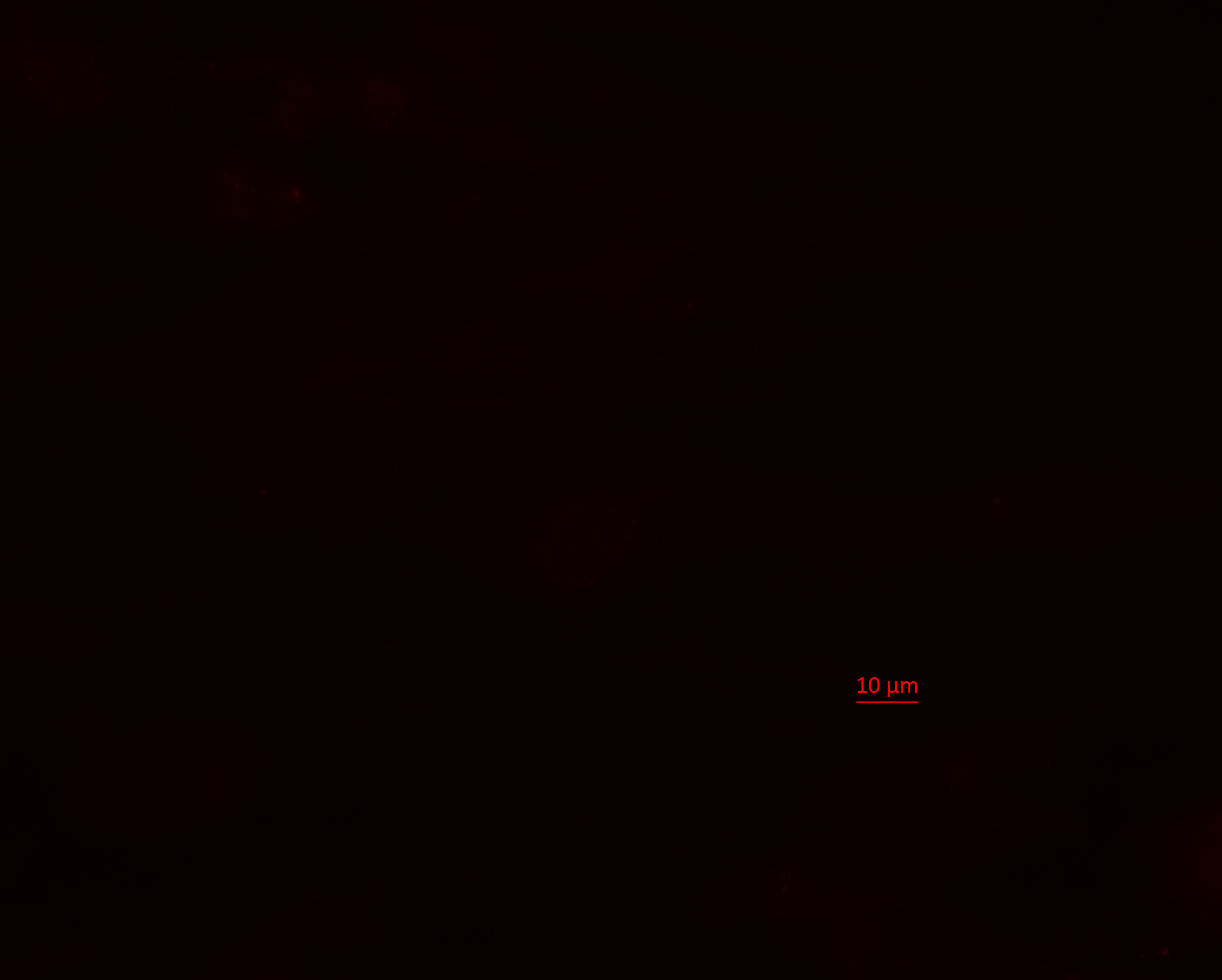

Supplement: Supplementary file 9 — Source data Fig. 3 [file 44321_2025_252_MOESM9_ESM.zip › Figure 3 Source Data/3c/BCLA (green) - DBA (red)/UT/Snap-1750_c4 (DBA).tif]

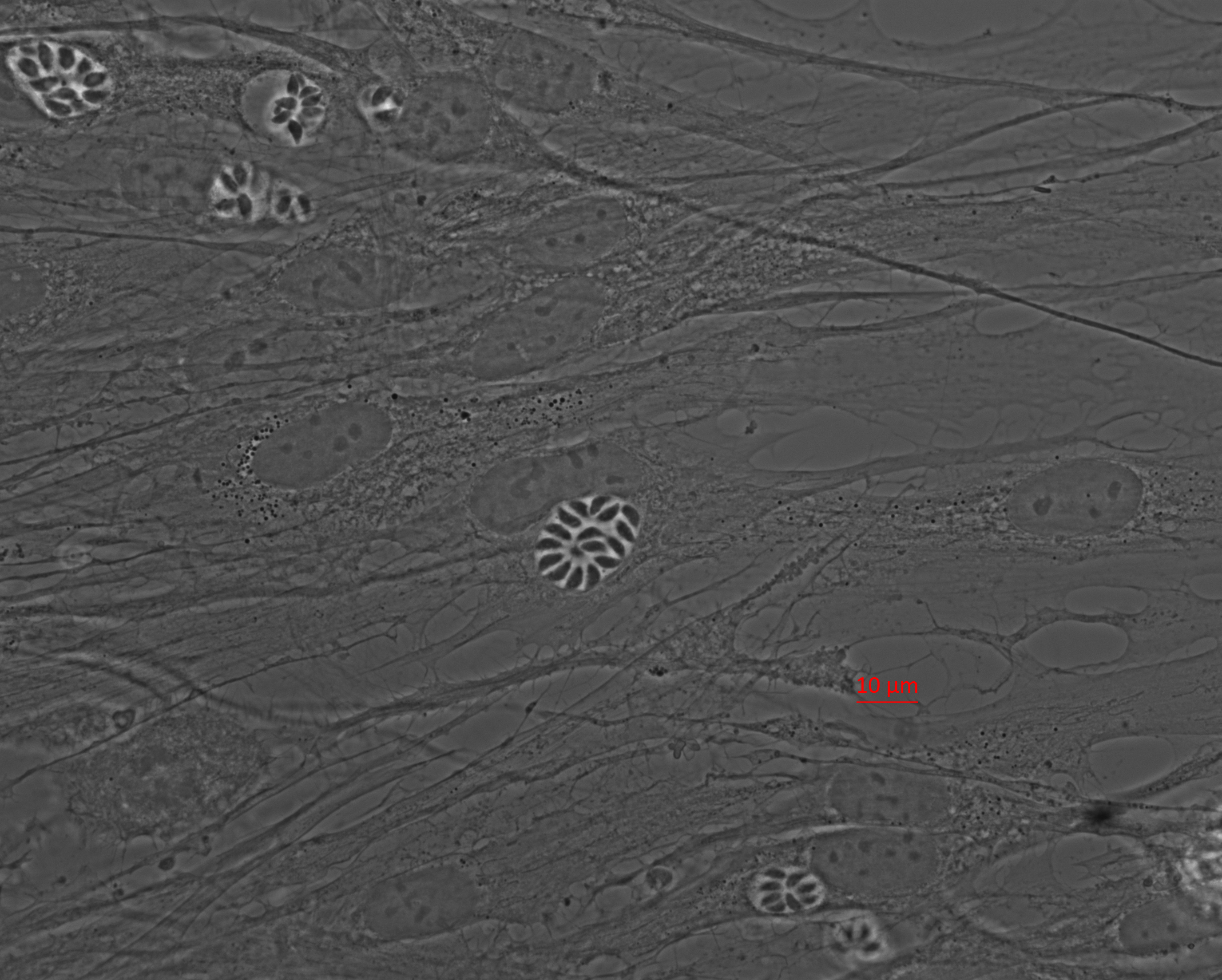

Supplement: Supplementary file 9 — Source data Fig. 3 [file 44321_2025_252_MOESM9_ESM.zip › Figure 3 Source Data/3c/BCLA (green) - DBA (red)/UT/Snap-1750_c1 (Phase).tif]

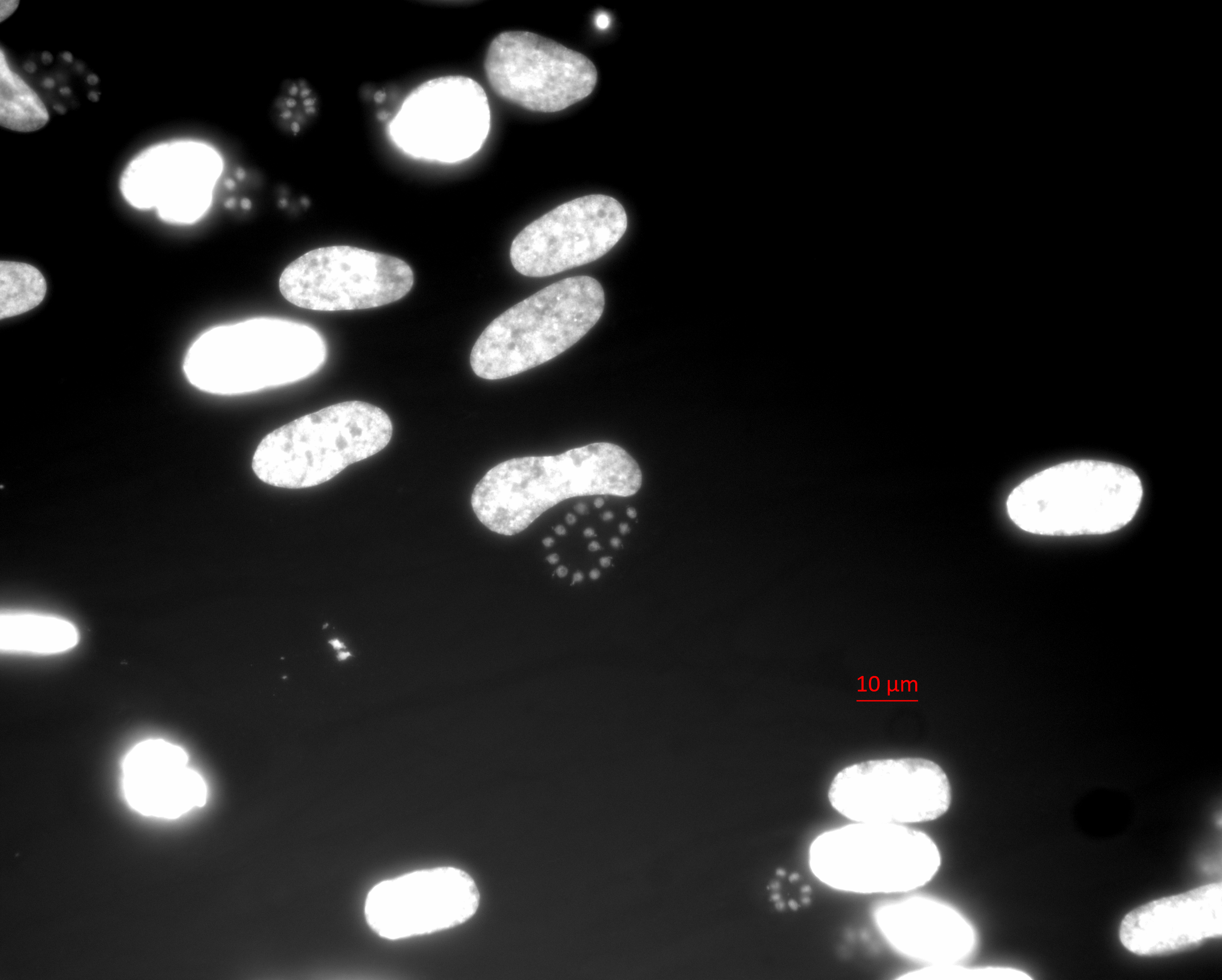

Supplement: Supplementary file 9 — Source data Fig. 3 [file 44321_2025_252_MOESM9_ESM.zip › Figure 3 Source Data/3c/BCLA (green) - DBA (red)/UT/Snap-1750_c2 (DNA).tif]

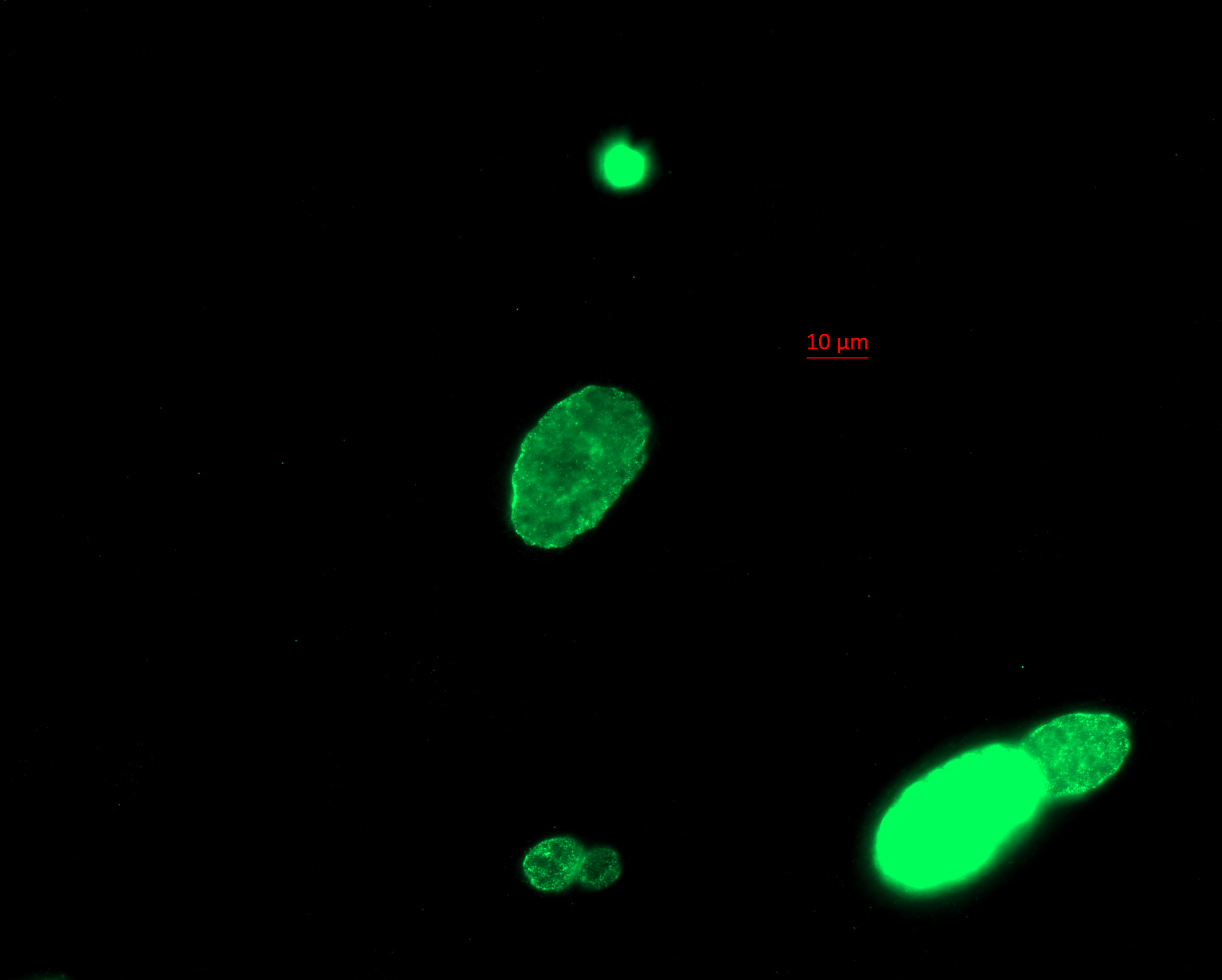

Supplement: Supplementary file 9 — Source data Fig. 3 [file 44321_2025_252_MOESM9_ESM.zip › Figure 3 Source Data/3c/BCLA (green) - DBA (red)/Shield (72h)/Snap-1748_c3 (BCLA).tif]

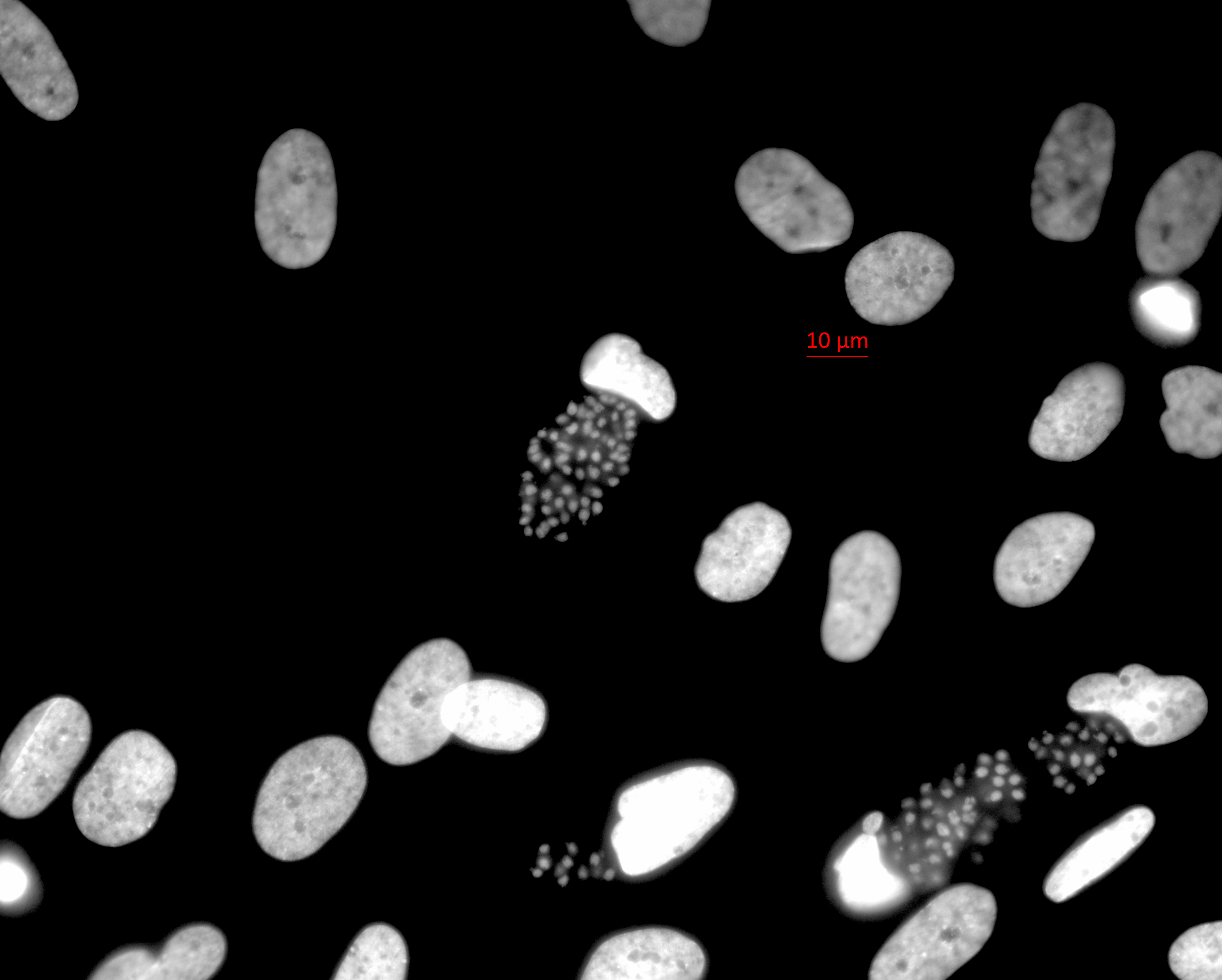

Supplement: Supplementary file 9 — Source data Fig. 3 [file 44321_2025_252_MOESM9_ESM.zip › Figure 3 Source Data/3c/BCLA (green) - DBA (red)/Shield (72h)/Snap-1748_c2 (DNA).tif]

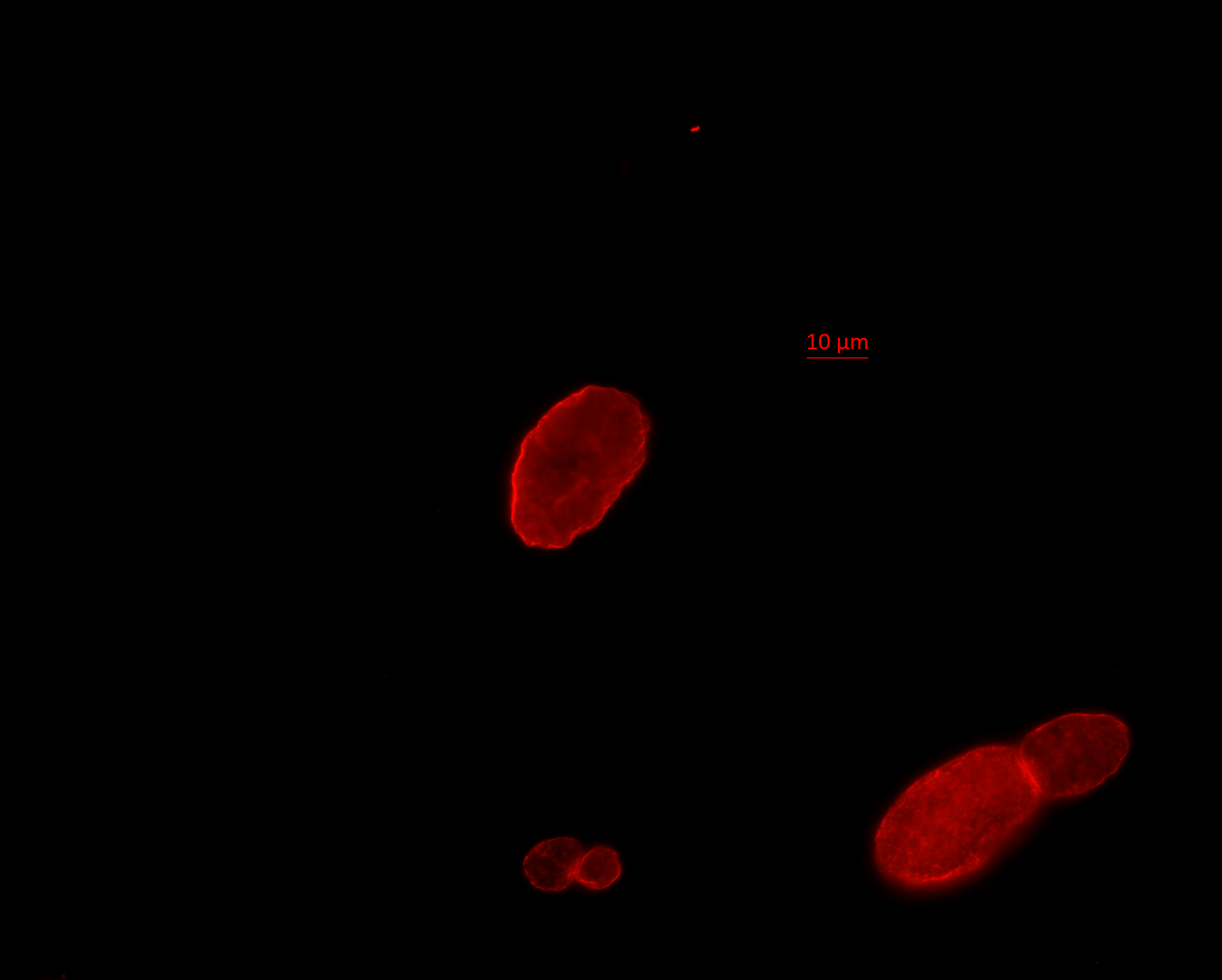

Supplement: Supplementary file 9 — Source data Fig. 3 [file 44321_2025_252_MOESM9_ESM.zip › Figure 3 Source Data/3c/BCLA (green) - DBA (red)/Shield (72h)/Snap-1748_c4 DBA.tif]
