# Supplementary figures and images for: Uncovering biomarkers for chronic toxoplasmosis detection highlights alternative pathways shaping parasite dormancy (part 2 of 2)
Source: EMBO Mol Med. 2025 May 19;17(7):1686–715. doi: 10.1038/s44321-025-00252-0 (PMC12254245; doi:10.1038/s44321-025-00252-0)

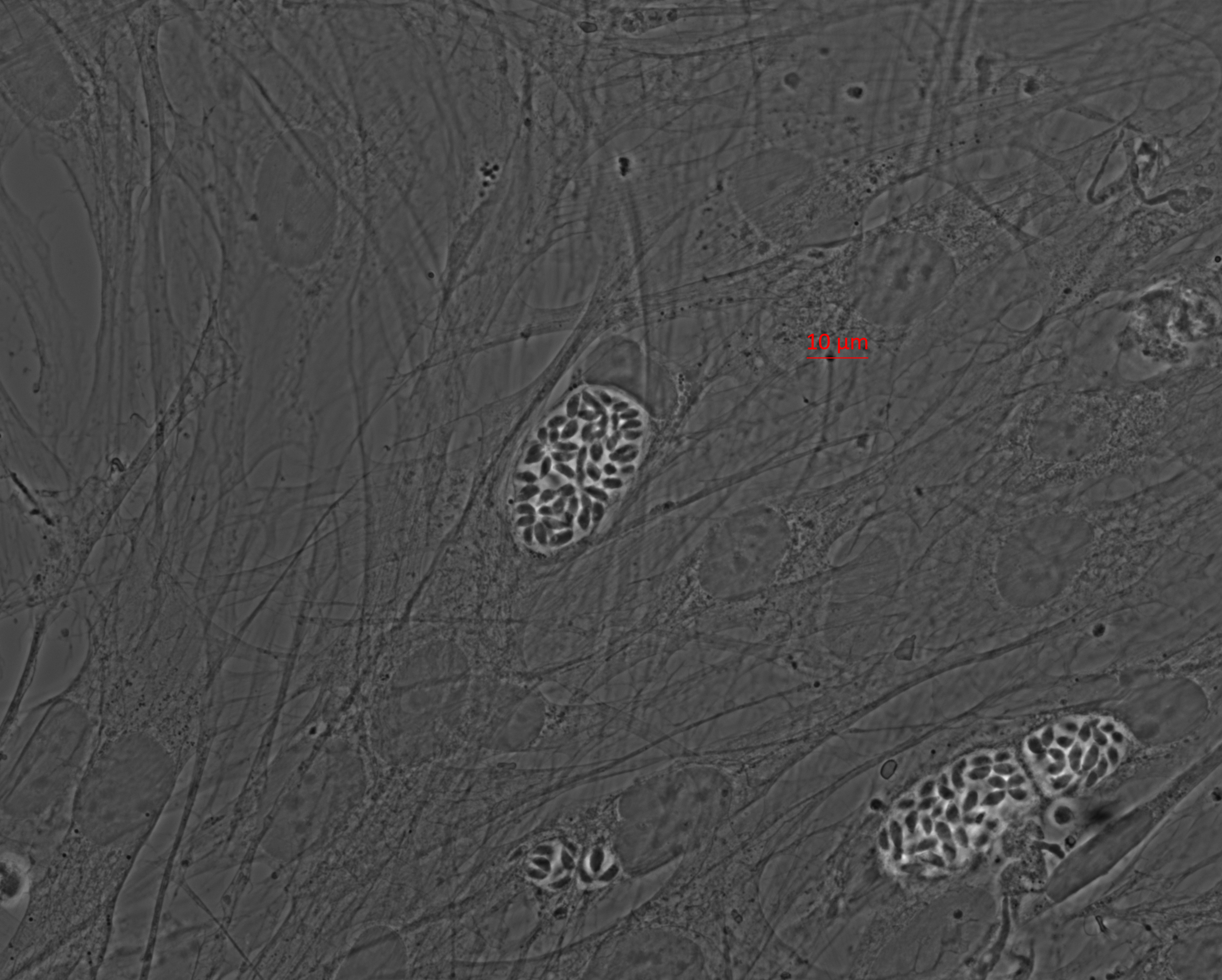

Supplement: Supplementary file 9 — Source data Fig. 3 [file 44321_2025_252_MOESM9_ESM.zip › Figure 3 Source Data/3c/BCLA (green) - DBA (red)/Shield (72h)/Snap-1748_c1 (Phase).tif]

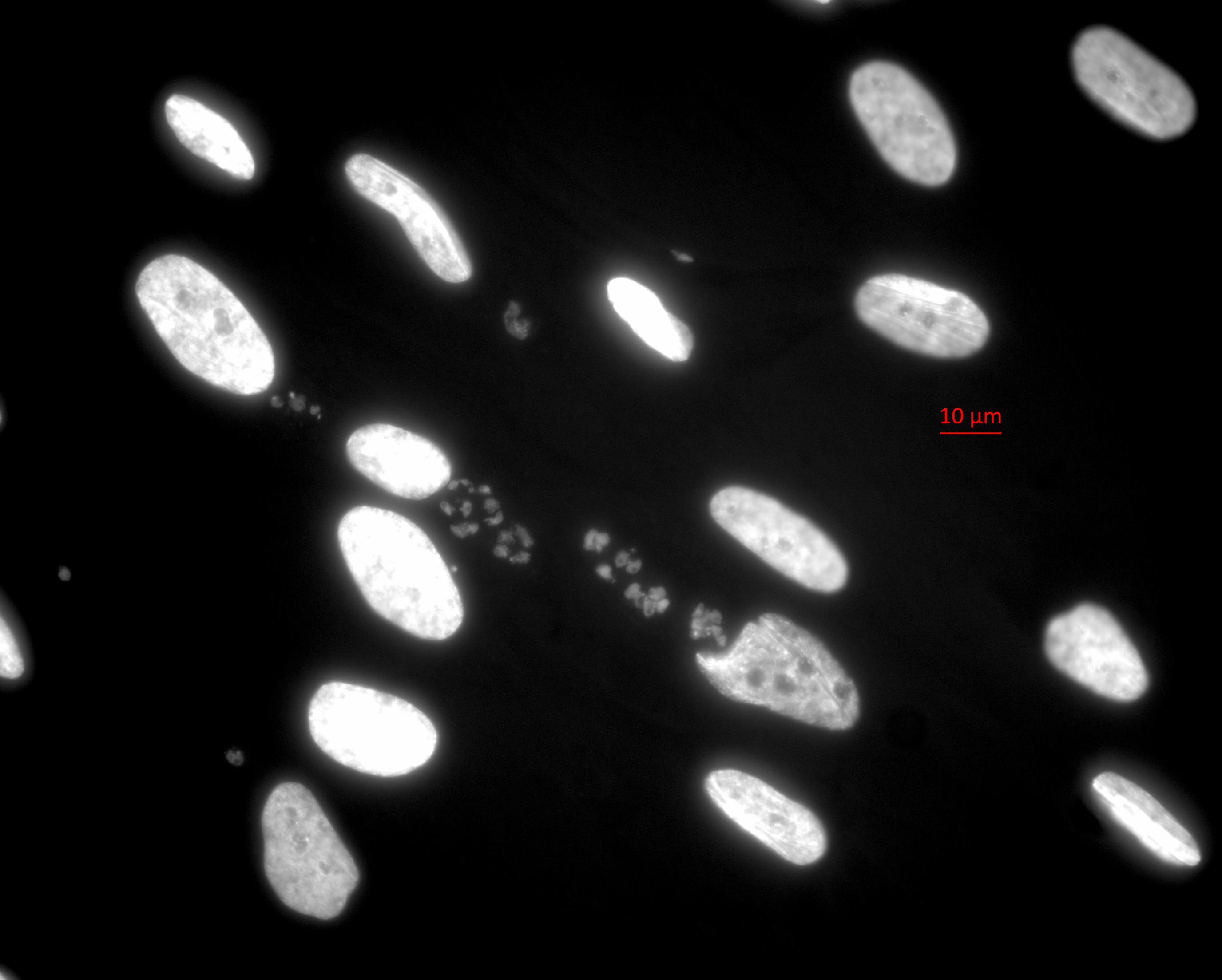

Supplement: Supplementary file 9 — Source data Fig. 3 [file 44321_2025_252_MOESM9_ESM.zip › Figure 3 Source Data/3a/Pru MORC KD/Dolichos red BCLA green/IAA/Snap-4002_c2 (DNA).tif]

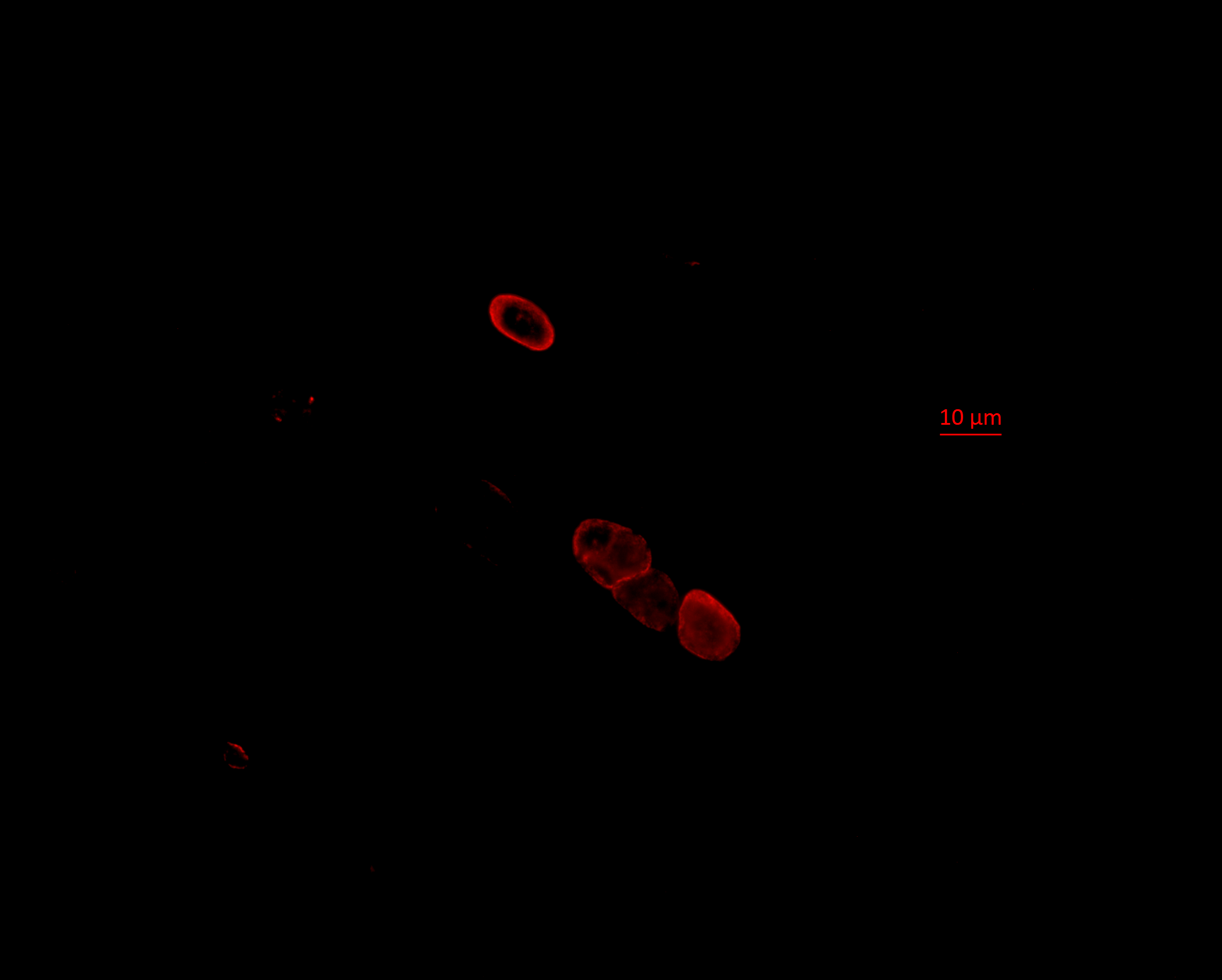

Supplement: Supplementary file 9 — Source data Fig. 3 [file 44321_2025_252_MOESM9_ESM.zip › Figure 3 Source Data/3a/Pru MORC KD/Dolichos red BCLA green/IAA/Snap-4002_c4 (Dolichos).tif]

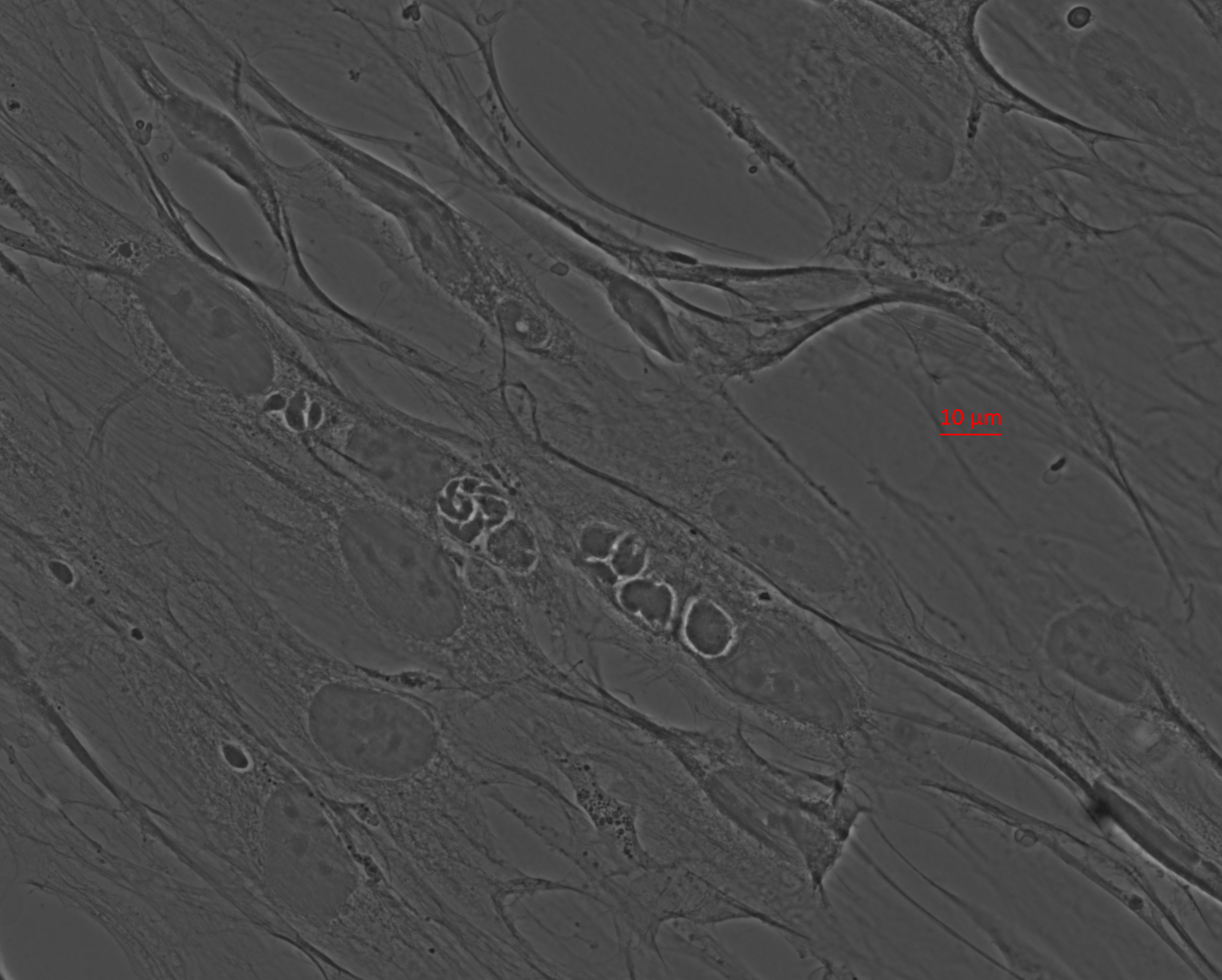

Supplement: Supplementary file 9 — Source data Fig. 3 [file 44321_2025_252_MOESM9_ESM.zip › Figure 3 Source Data/3a/Pru MORC KD/Dolichos red BCLA green/IAA/Snap-4002_c1 (Phase).tif]

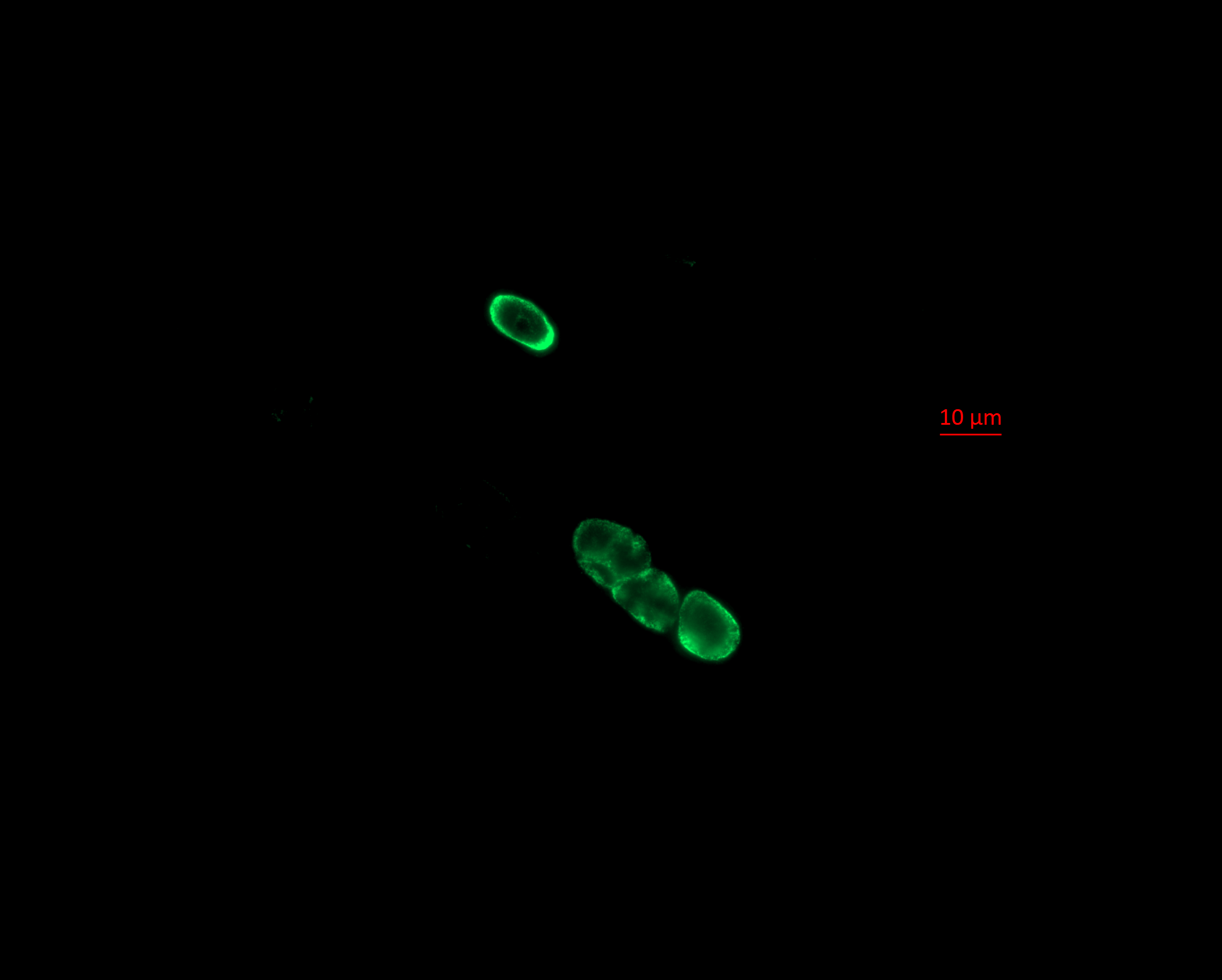

Supplement: Supplementary file 9 — Source data Fig. 3 [file 44321_2025_252_MOESM9_ESM.zip › Figure 3 Source Data/3a/Pru MORC KD/Dolichos red BCLA green/IAA/Snap-4002_c3 (BCLA).tif]

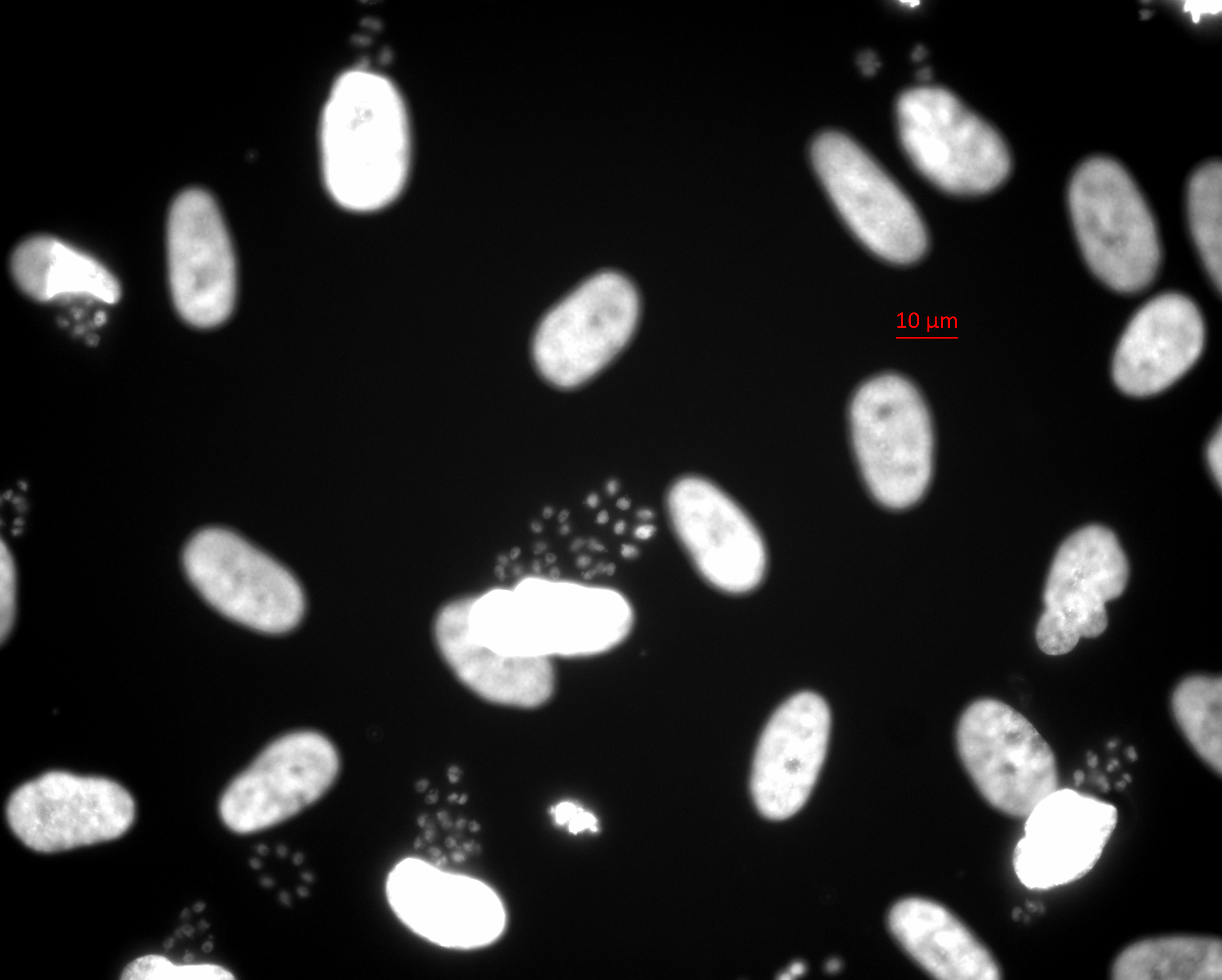

Supplement: Supplementary file 9 — Source data Fig. 3 [file 44321_2025_252_MOESM9_ESM.zip › Figure 3 Source Data/3a/Pru MORC KD/Dolichos red BCLA green/UT/Snap-4005_c2 (DNA).tif]

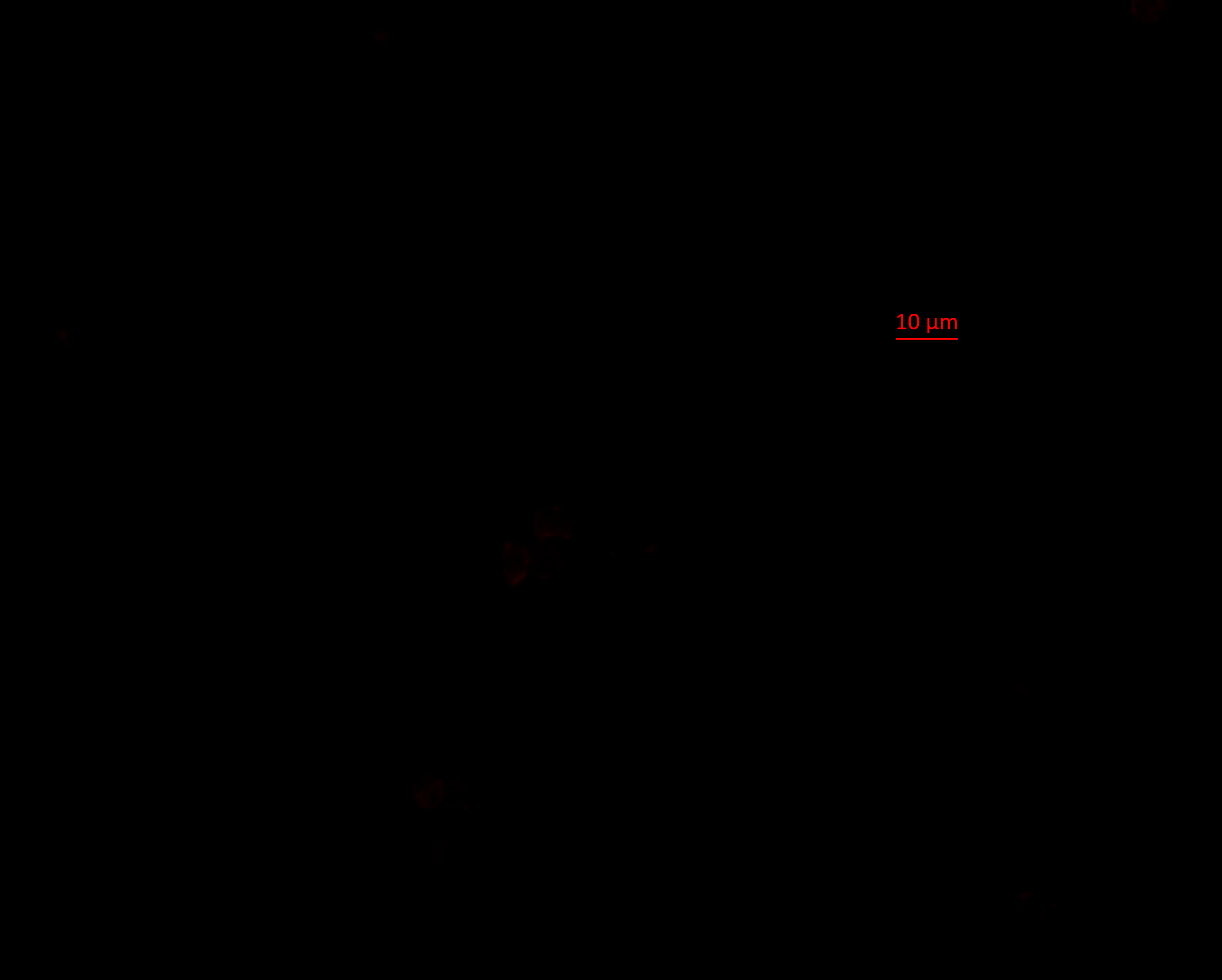

Supplement: Supplementary file 9 — Source data Fig. 3 [file 44321_2025_252_MOESM9_ESM.zip › Figure 3 Source Data/3a/Pru MORC KD/Dolichos red BCLA green/UT/Snap-4005_c4 (Dolichos).tif]

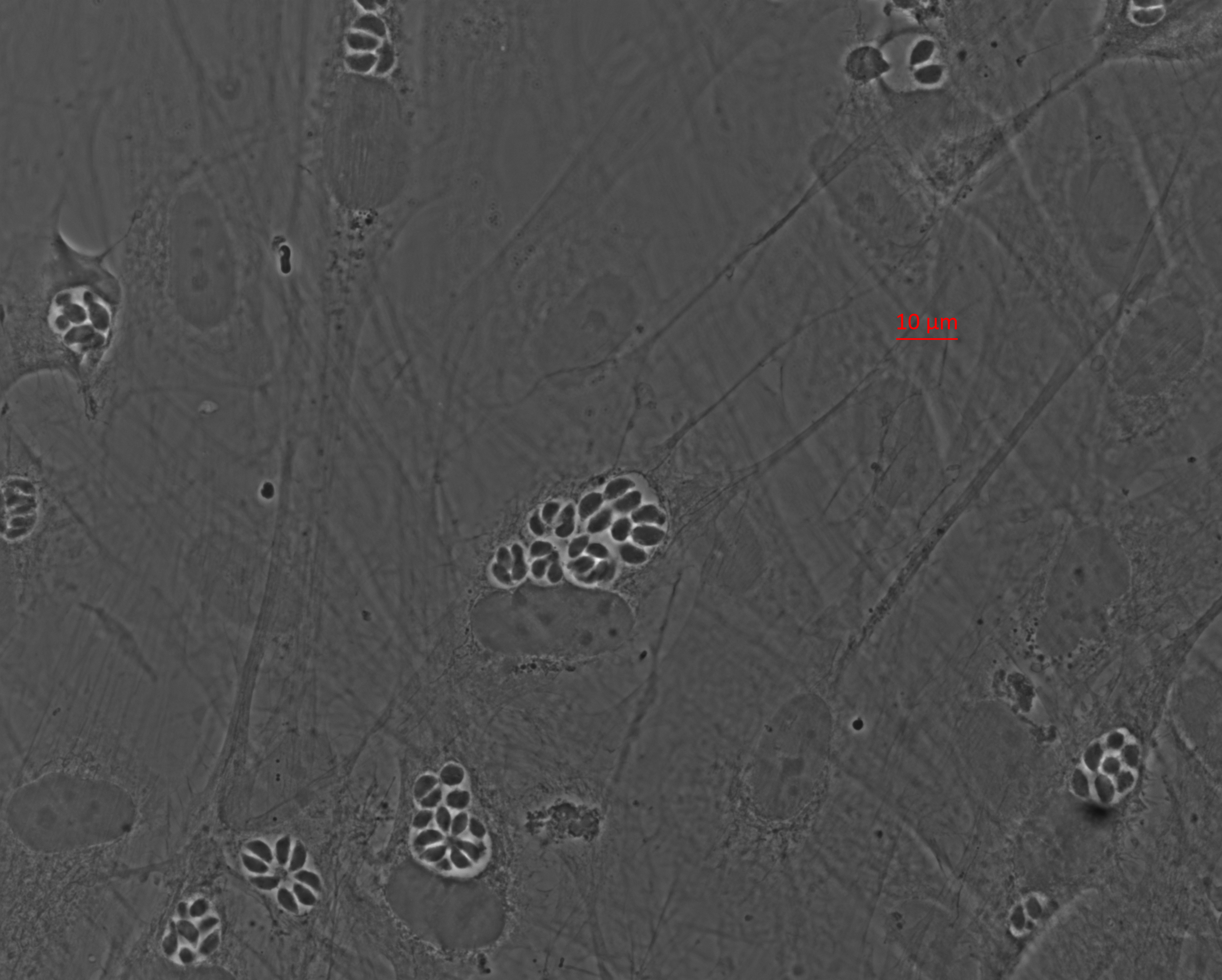

Supplement: Supplementary file 9 — Source data Fig. 3 [file 44321_2025_252_MOESM9_ESM.zip › Figure 3 Source Data/3a/Pru MORC KD/Dolichos red BCLA green/UT/Snap-4005_c1 (Phase).tif]

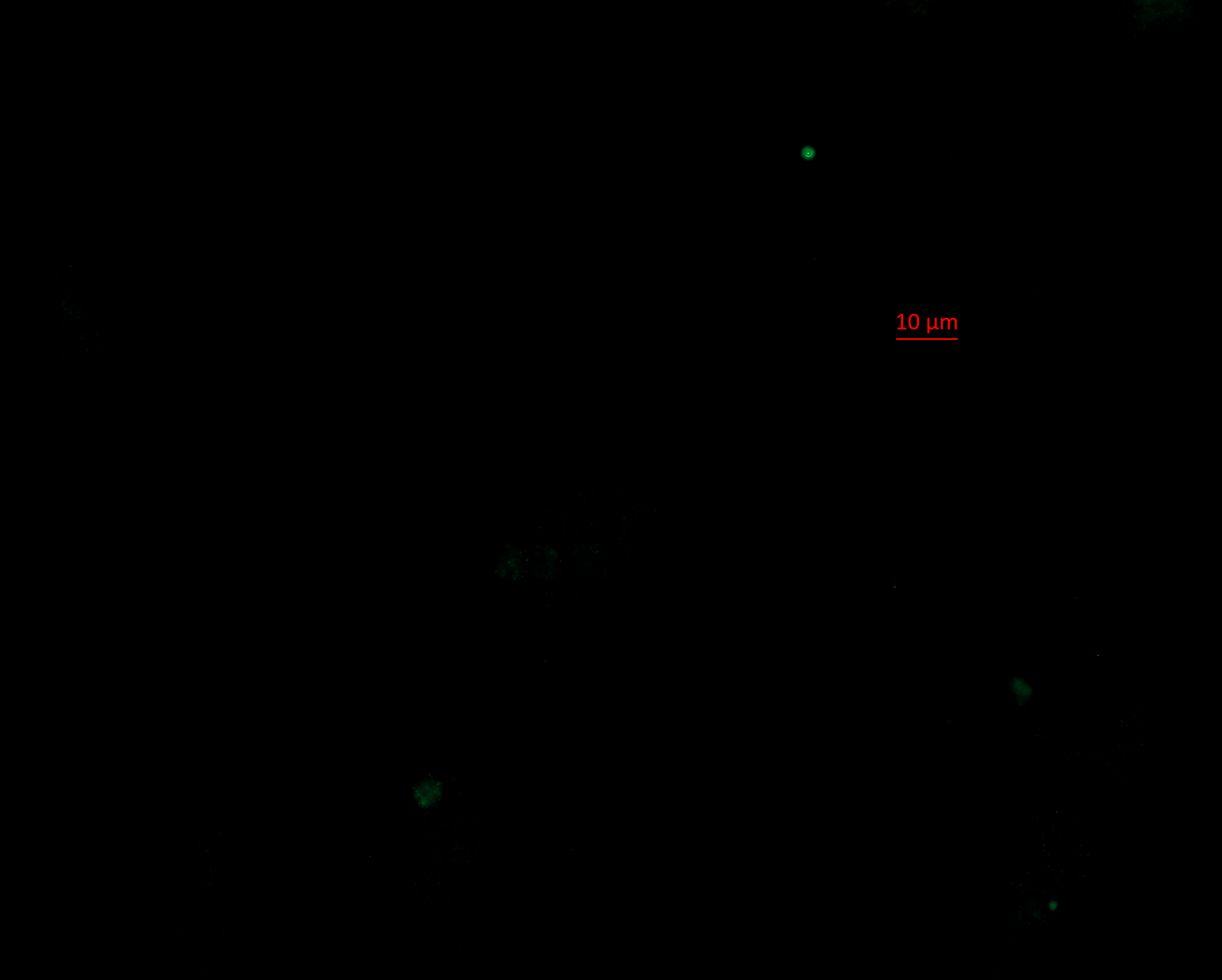

Supplement: Supplementary file 9 — Source data Fig. 3 [file 44321_2025_252_MOESM9_ESM.zip › Figure 3 Source Data/3a/Pru MORC KD/Dolichos red BCLA green/UT/Snap-4005_c3 (BCLA).tif]

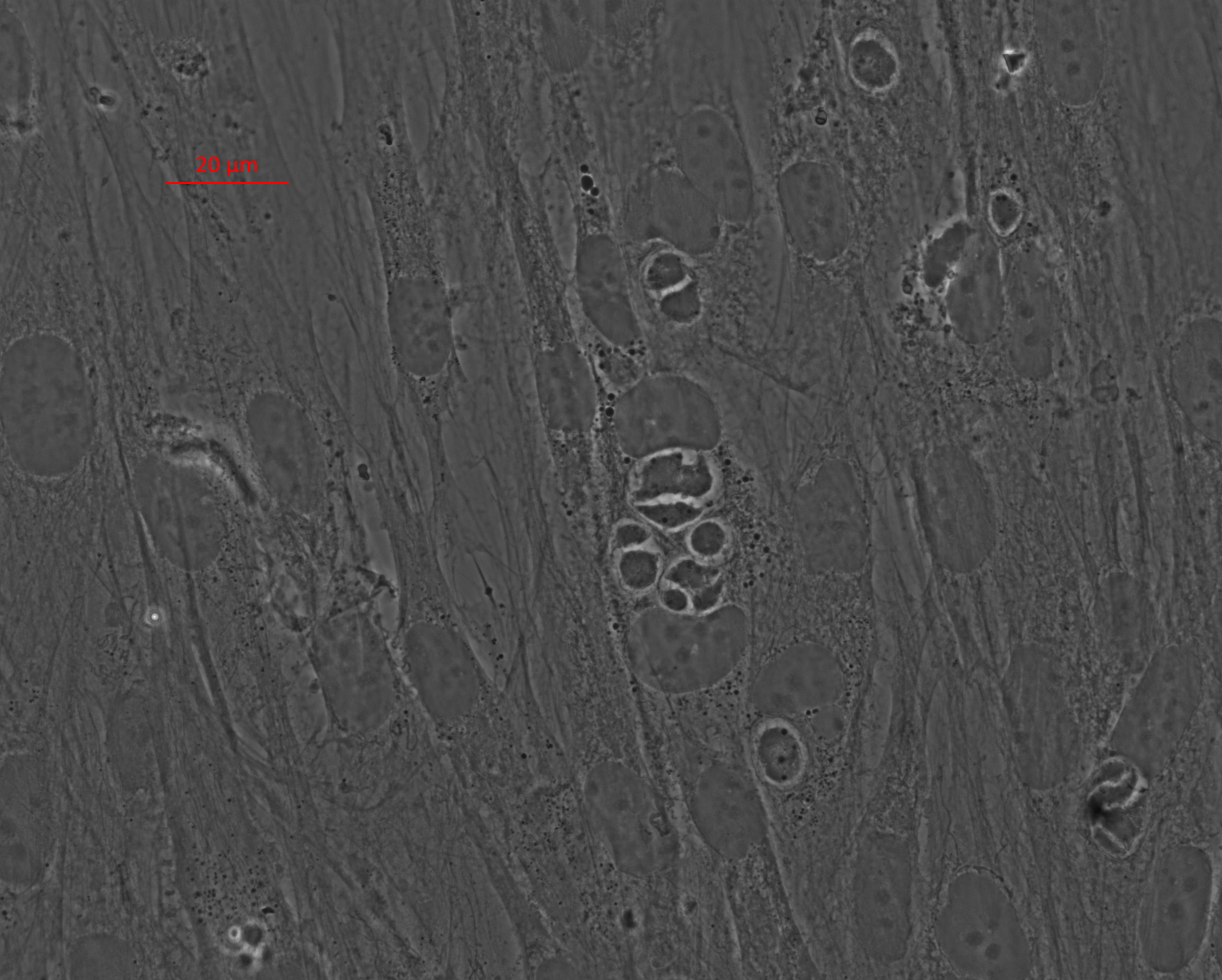

Supplement: Supplementary file 9 — Source data Fig. 3 [file 44321_2025_252_MOESM9_ESM.zip › Figure 3 Source Data/3a/Pru MORC KD/Dolichos red BSM green/IAA/Snap-3962_c1 (Phase).tif]

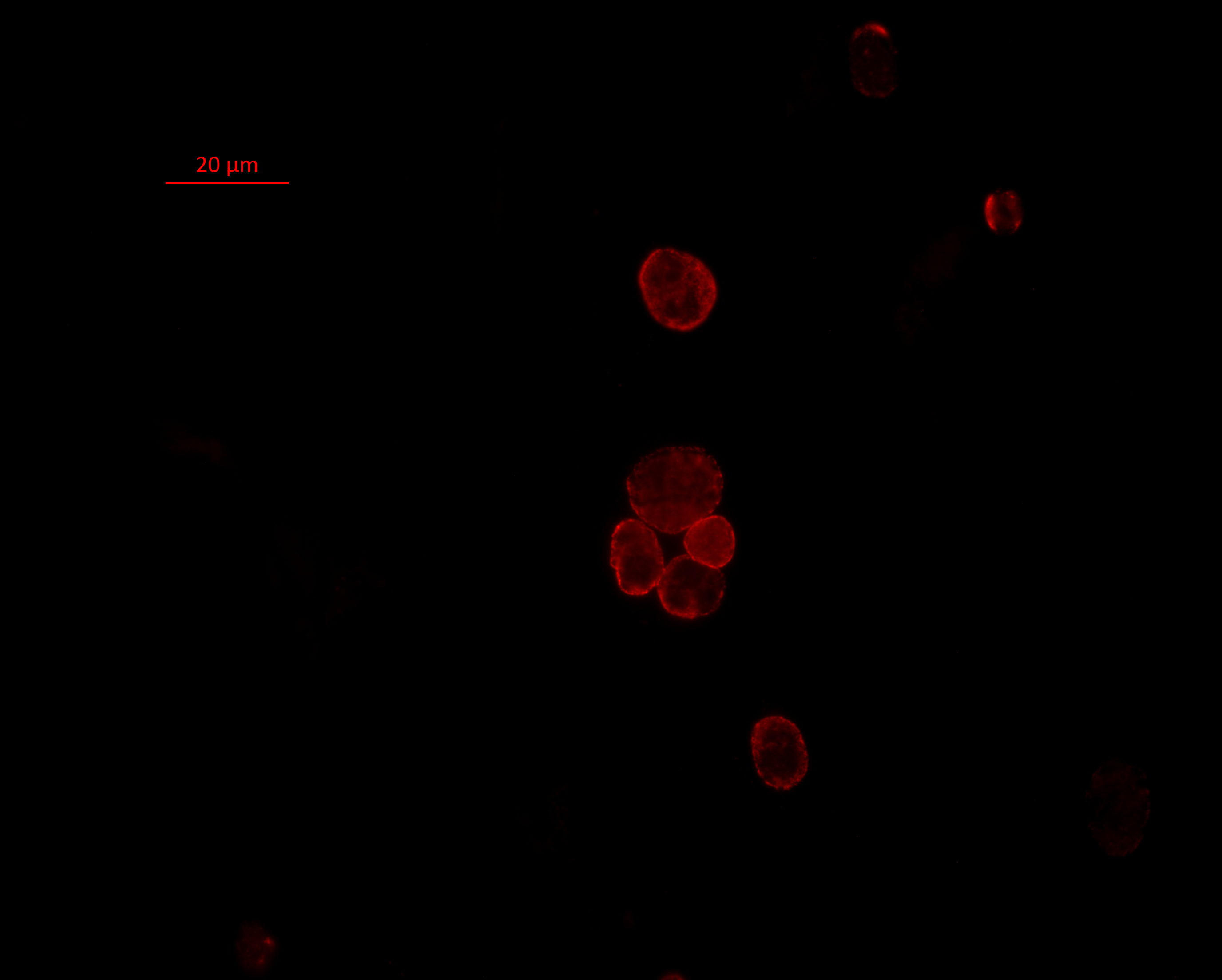

Supplement: Supplementary file 9 — Source data Fig. 3 [file 44321_2025_252_MOESM9_ESM.zip › Figure 3 Source Data/3a/Pru MORC KD/Dolichos red BSM green/IAA/Snap-3962_c4 (Dolichos).tif]

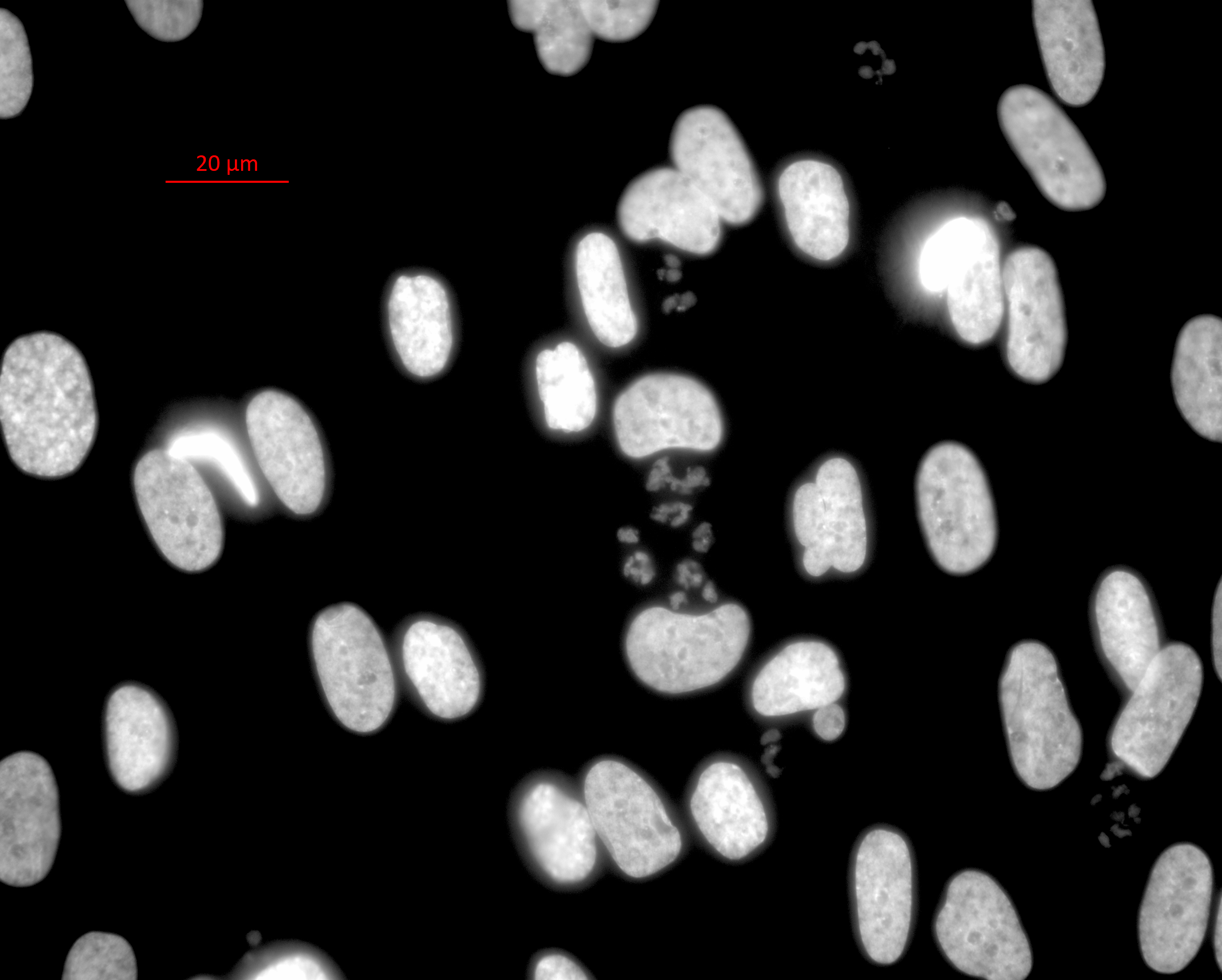

Supplement: Supplementary file 9 — Source data Fig. 3 [file 44321_2025_252_MOESM9_ESM.zip › Figure 3 Source Data/3a/Pru MORC KD/Dolichos red BSM green/IAA/Snap-3962_c2 (DNA).tif]

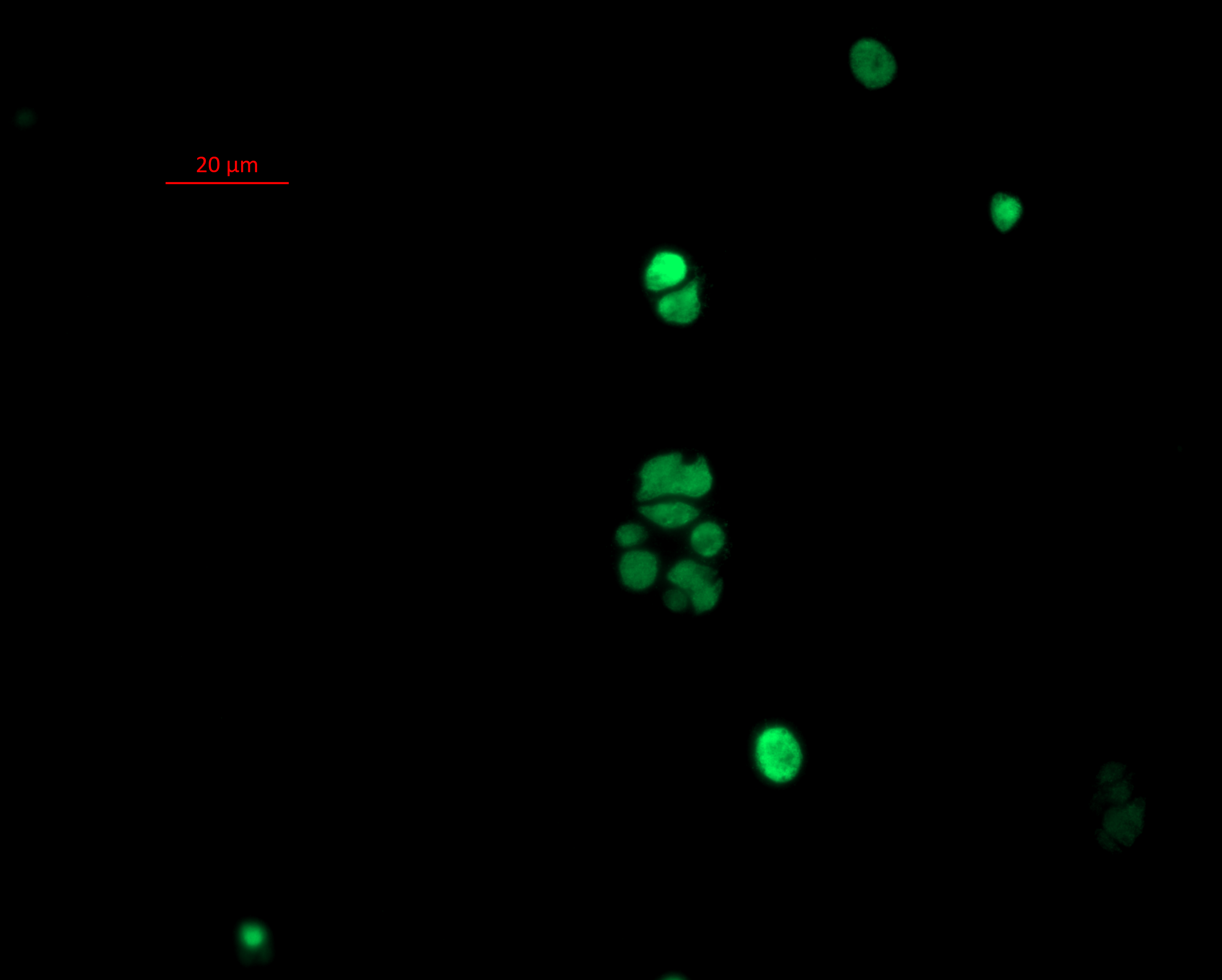

Supplement: Supplementary file 9 — Source data Fig. 3 [file 44321_2025_252_MOESM9_ESM.zip › Figure 3 Source Data/3a/Pru MORC KD/Dolichos red BSM green/IAA/Snap-3962_c3 (BSM).tif]

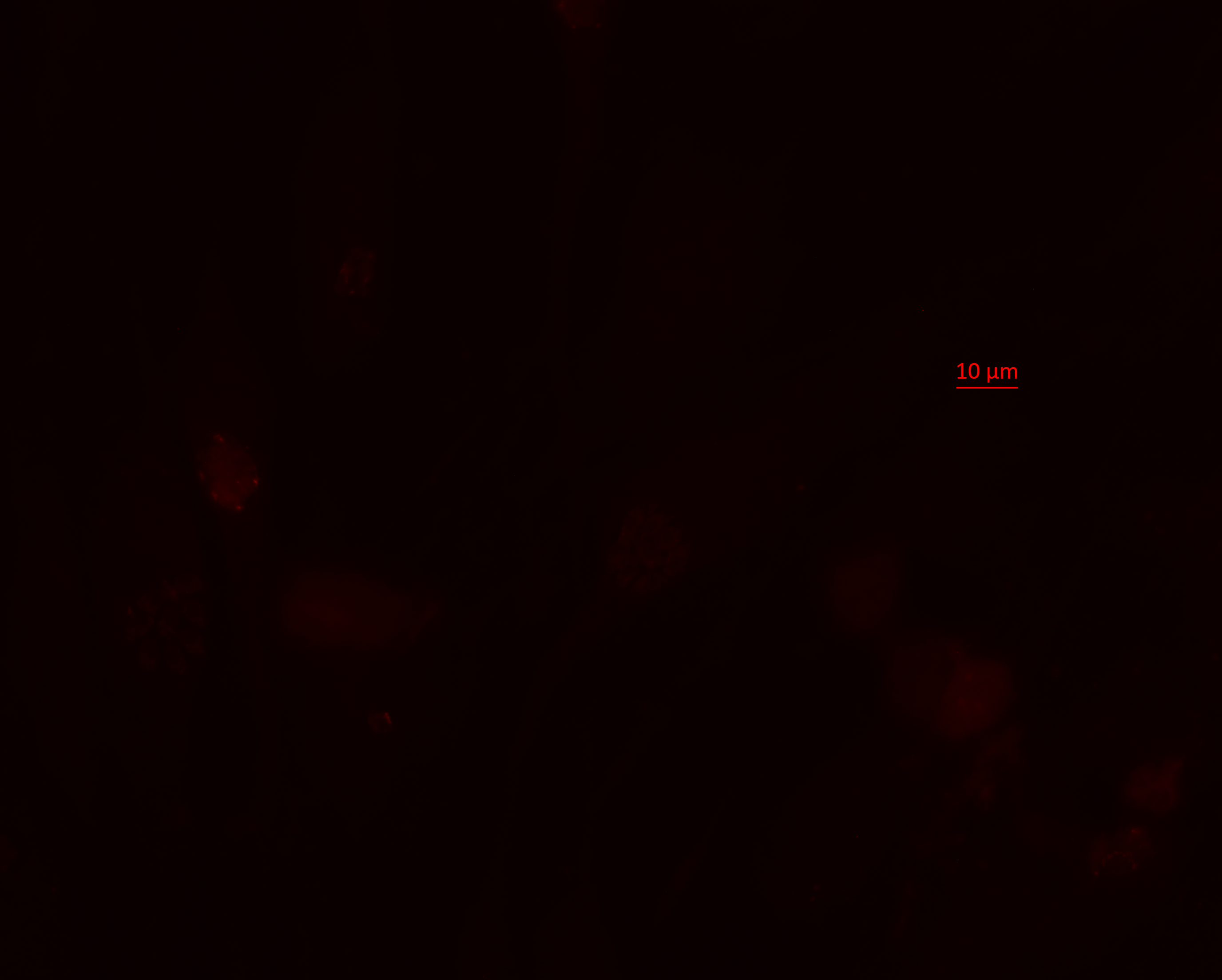

Supplement: Supplementary file 9 — Source data Fig. 3 [file 44321_2025_252_MOESM9_ESM.zip › Figure 3 Source Data/3a/Pru MORC KD/Dolichos red BSM green/UT/Snap-3976_c4 (DOLICHOS).tif]

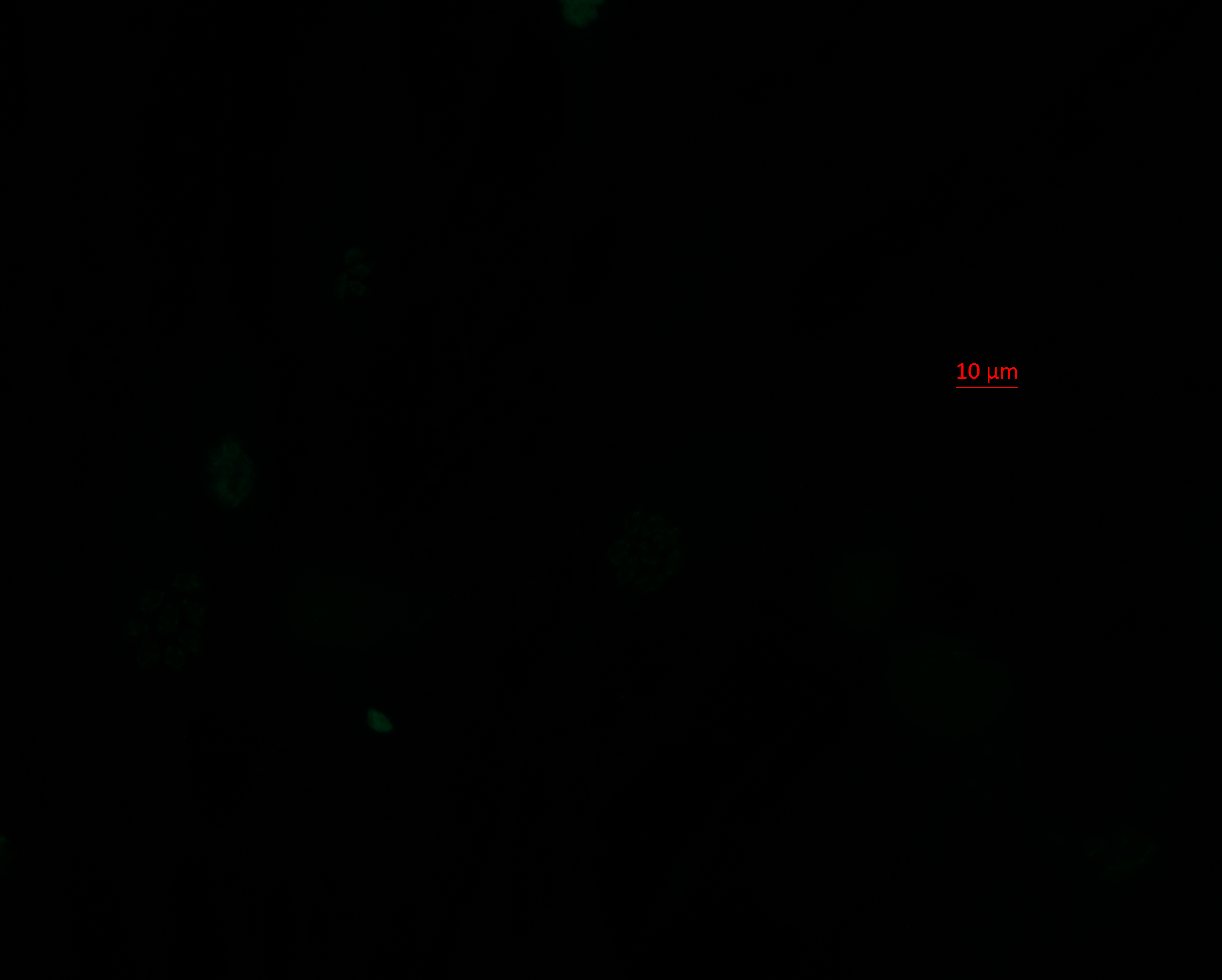

Supplement: Supplementary file 9 — Source data Fig. 3 [file 44321_2025_252_MOESM9_ESM.zip › Figure 3 Source Data/3a/Pru MORC KD/Dolichos red BSM green/UT/Snap-3976_c3 (BSM).tif]

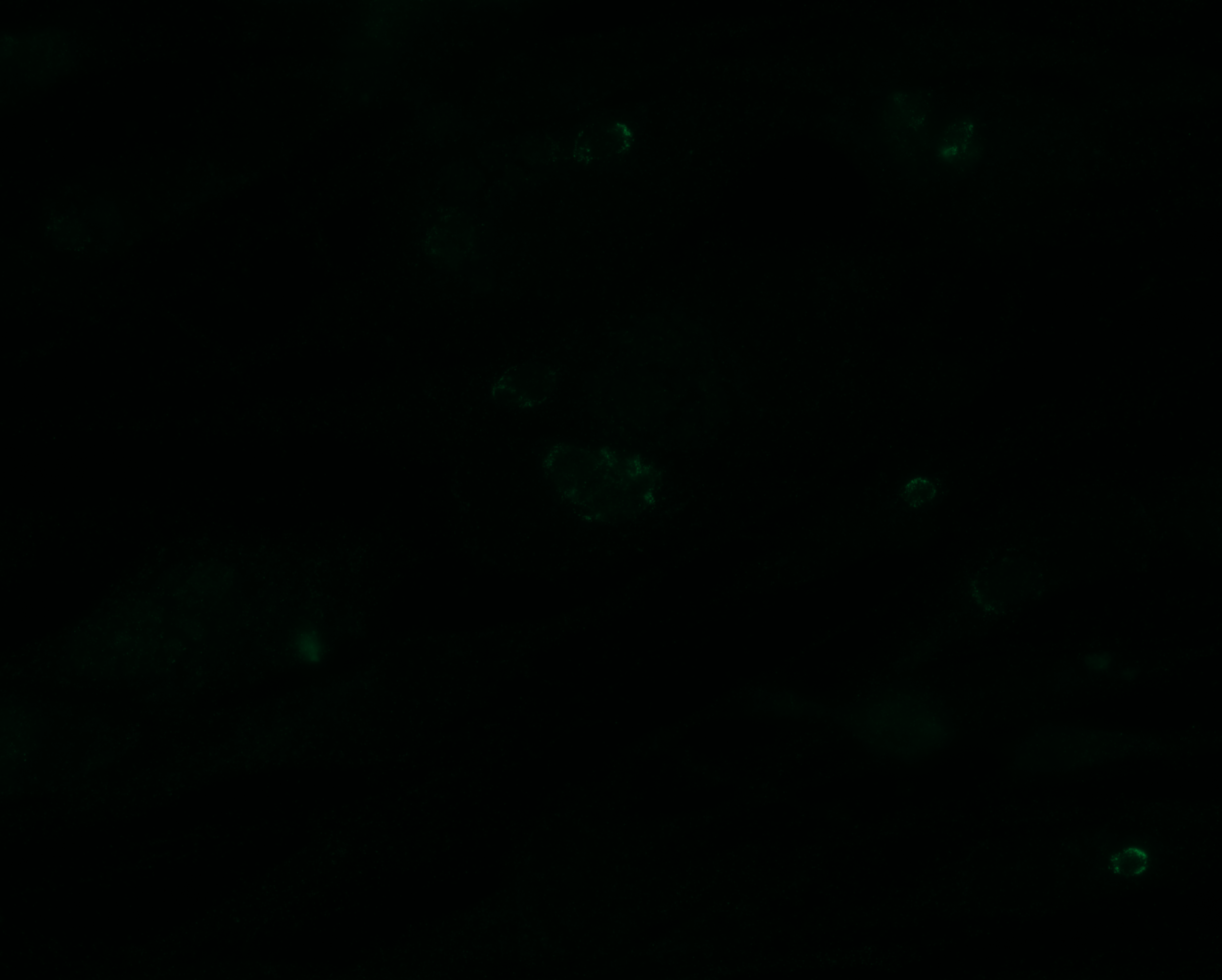

Supplement: Supplementary file 9 — Source data Fig. 3 [file 44321_2025_252_MOESM9_ESM.zip › Figure 3 Source Data/3a/Pru MORC KD BFD1 KO/Dolichos red BCLA green/IAA/Snap-4010_c3 (BCLA).tif]

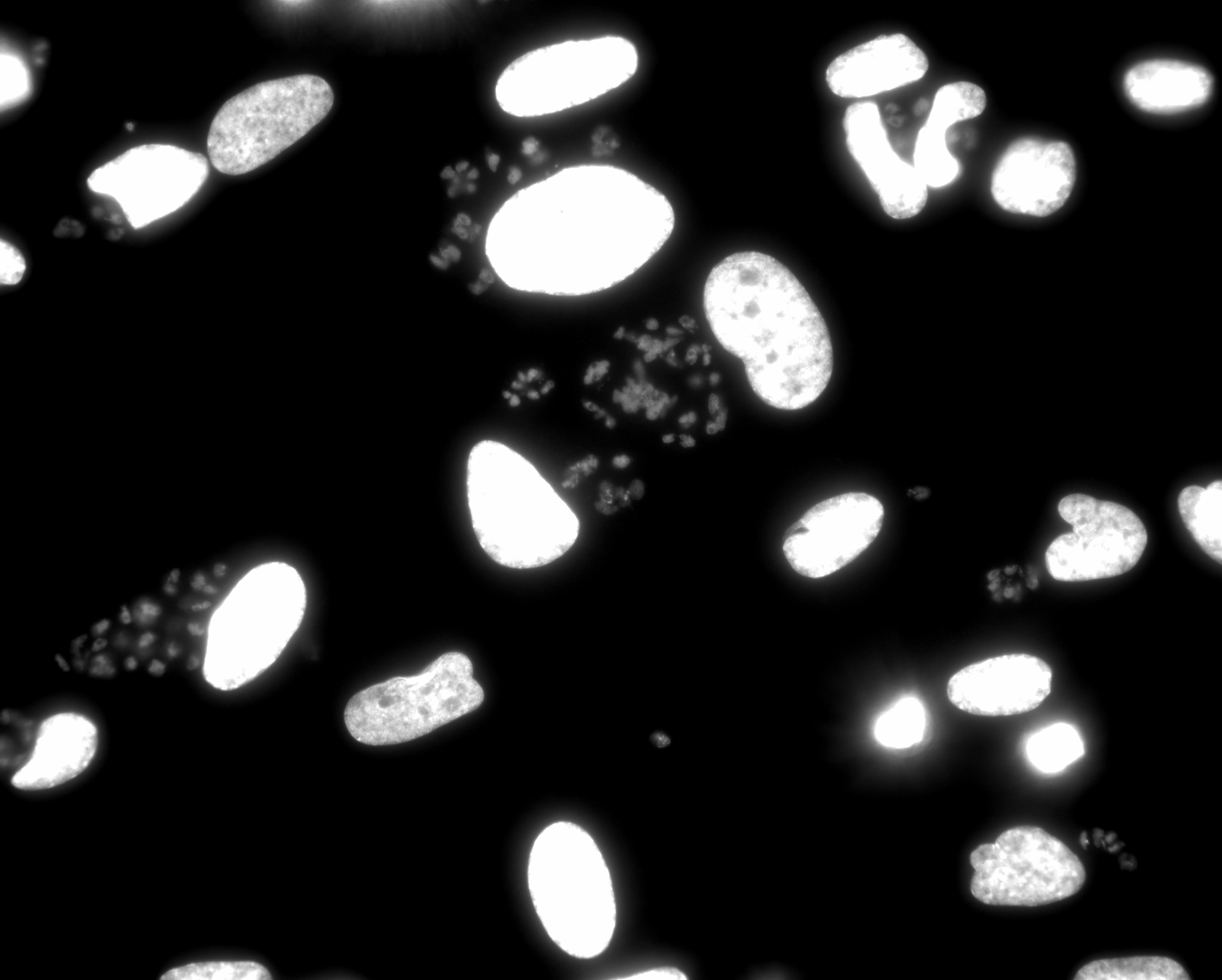

Supplement: Supplementary file 9 — Source data Fig. 3 [file 44321_2025_252_MOESM9_ESM.zip › Figure 3 Source Data/3a/Pru MORC KD BFD1 KO/Dolichos red BCLA green/IAA/Snap-4010_c2 (DNA).tif]

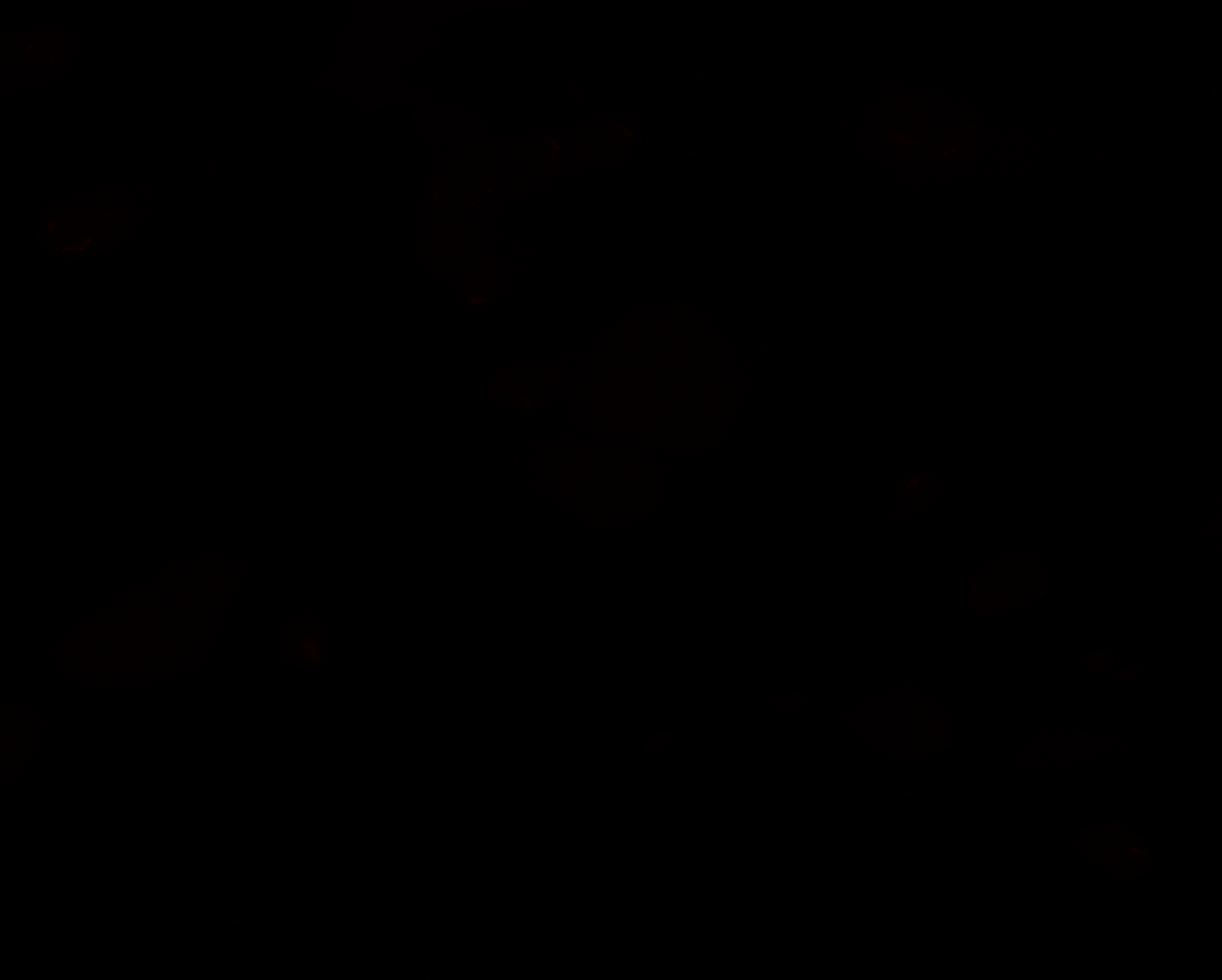

Supplement: Supplementary file 9 — Source data Fig. 3 [file 44321_2025_252_MOESM9_ESM.zip › Figure 3 Source Data/3a/Pru MORC KD BFD1 KO/Dolichos red BCLA green/IAA/Snap-4010_c4 (Dolichos).tif]

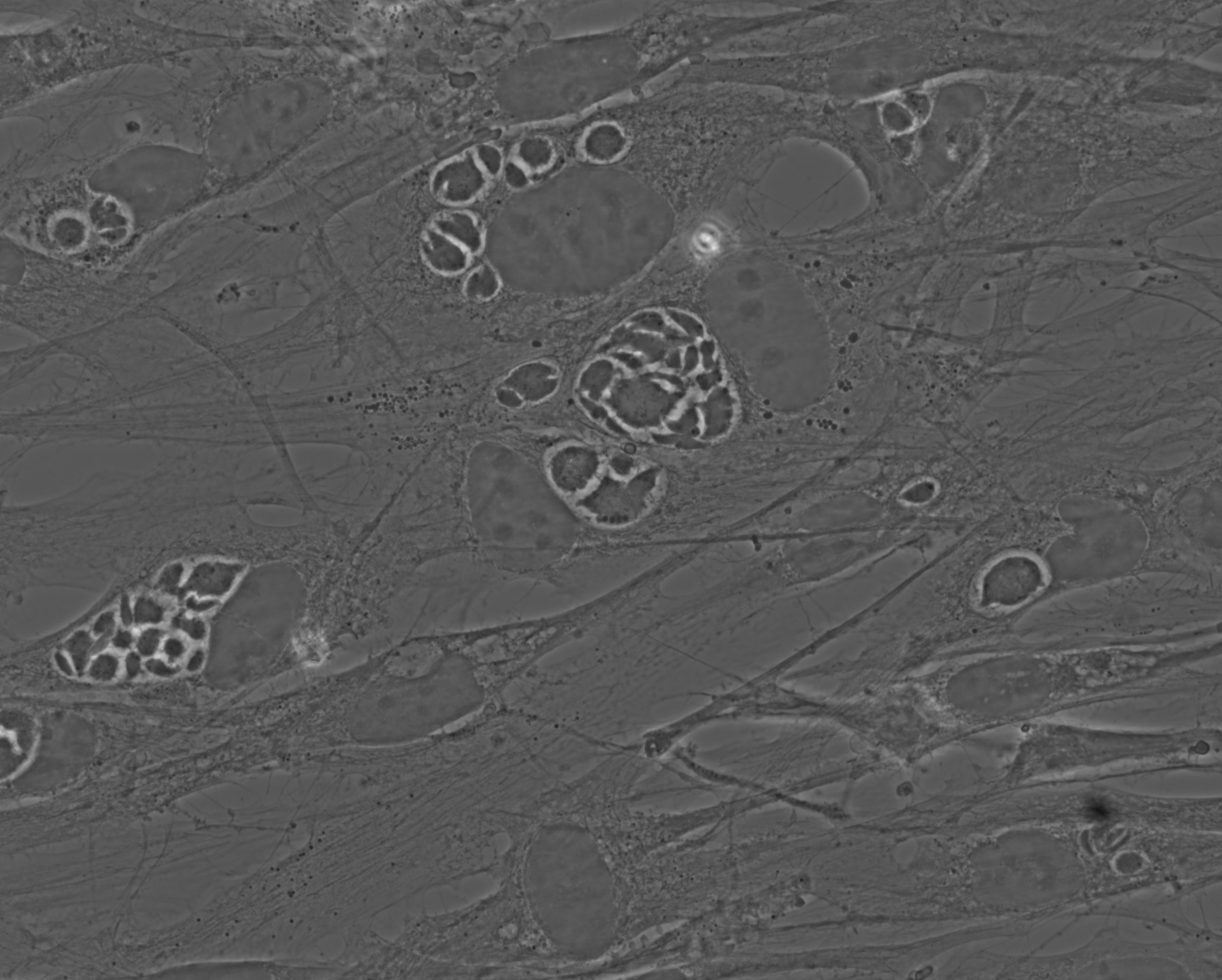

Supplement: Supplementary file 9 — Source data Fig. 3 [file 44321_2025_252_MOESM9_ESM.zip › Figure 3 Source Data/3a/Pru MORC KD BFD1 KO/Dolichos red BCLA green/IAA/Snap-4010_c1 (Phase).tif]

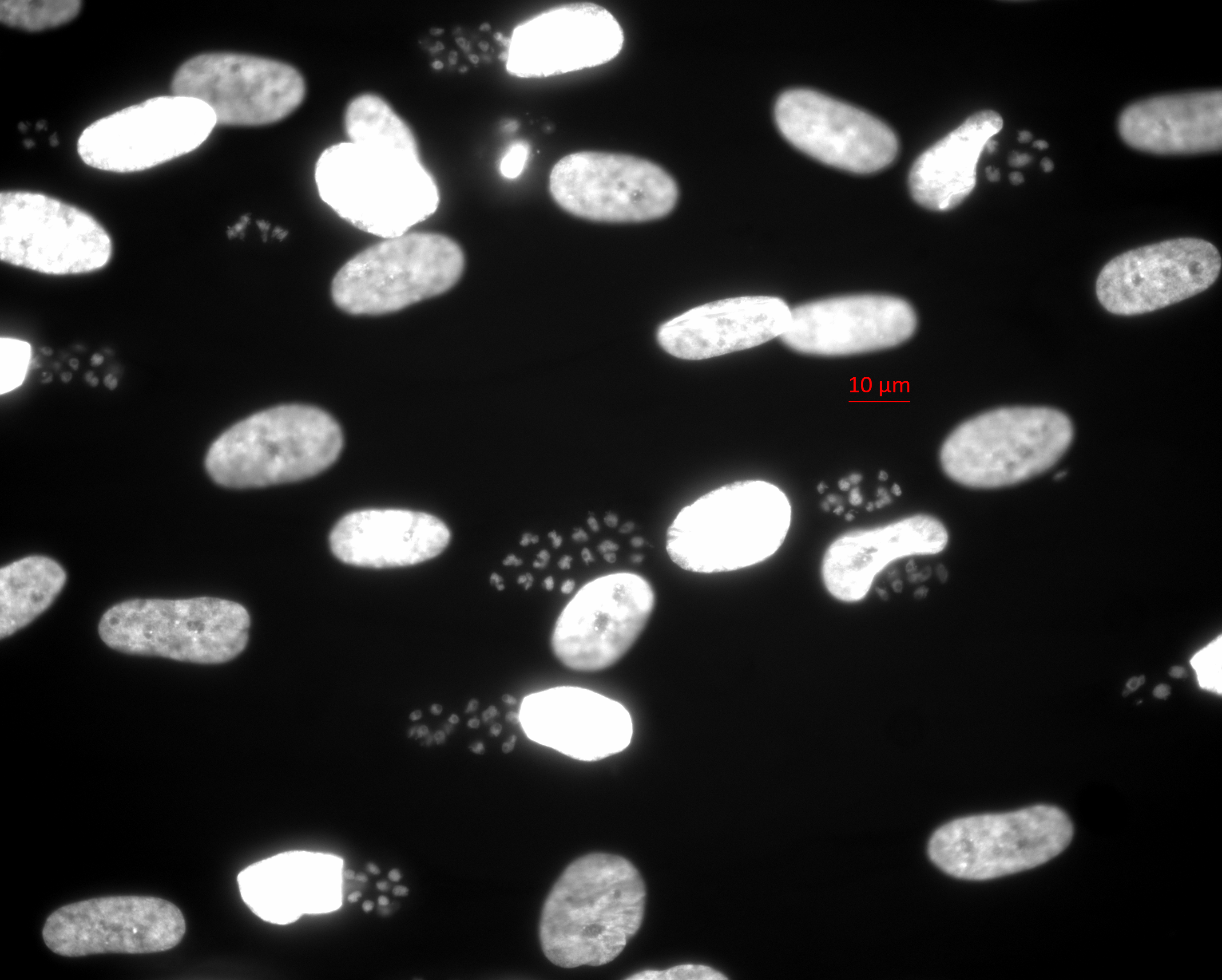

Supplement: Supplementary file 9 — Source data Fig. 3 [file 44321_2025_252_MOESM9_ESM.zip › Figure 3 Source Data/3a/Pru MORC KD BFD1 KO/Dolichos red BCLA green/UT/Snap-4017_c2 (DNA).tif]

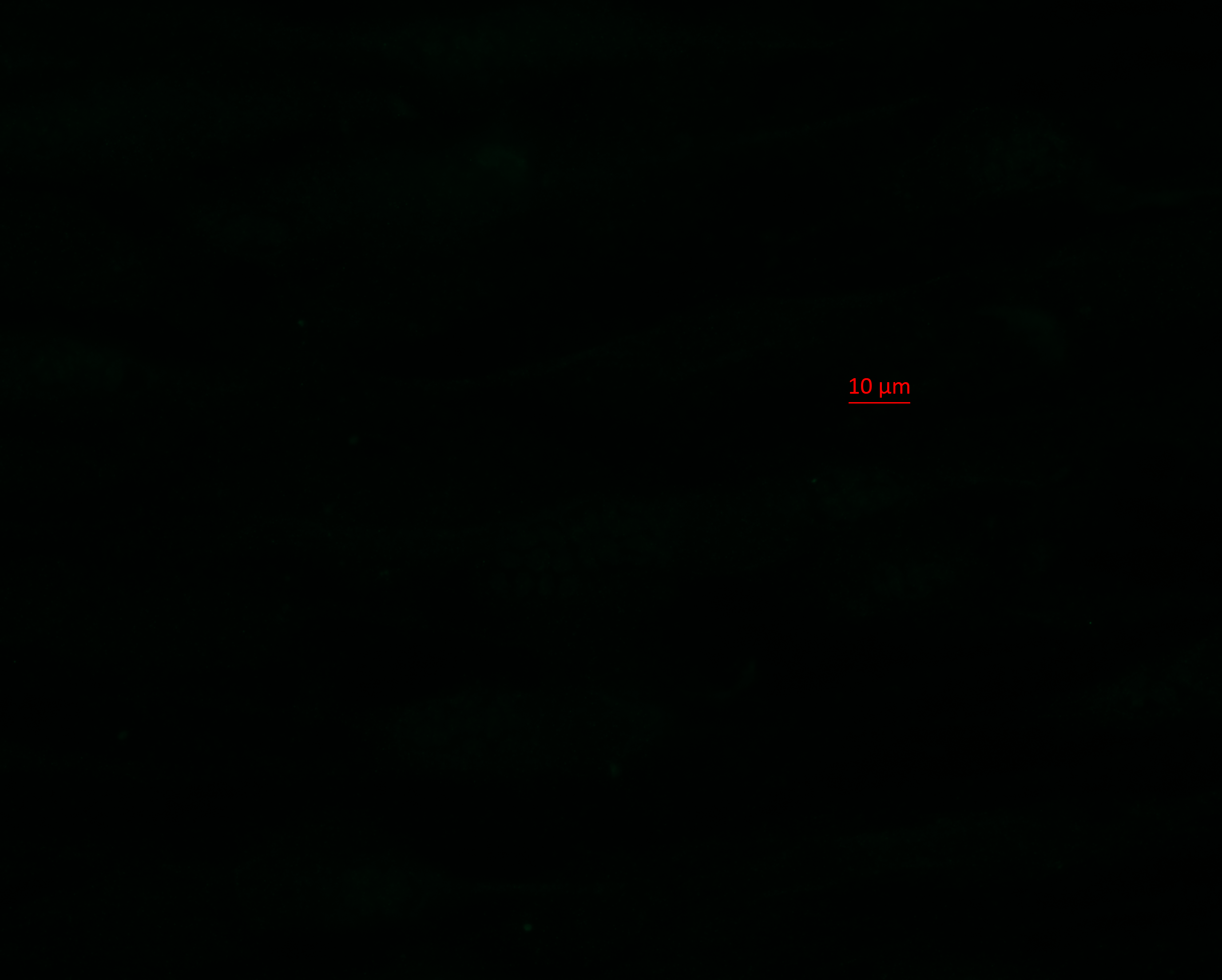

Supplement: Supplementary file 9 — Source data Fig. 3 [file 44321_2025_252_MOESM9_ESM.zip › Figure 3 Source Data/3a/Pru MORC KD BFD1 KO/Dolichos red BCLA green/UT/Snap-4017_c3 (BCLA).tif]

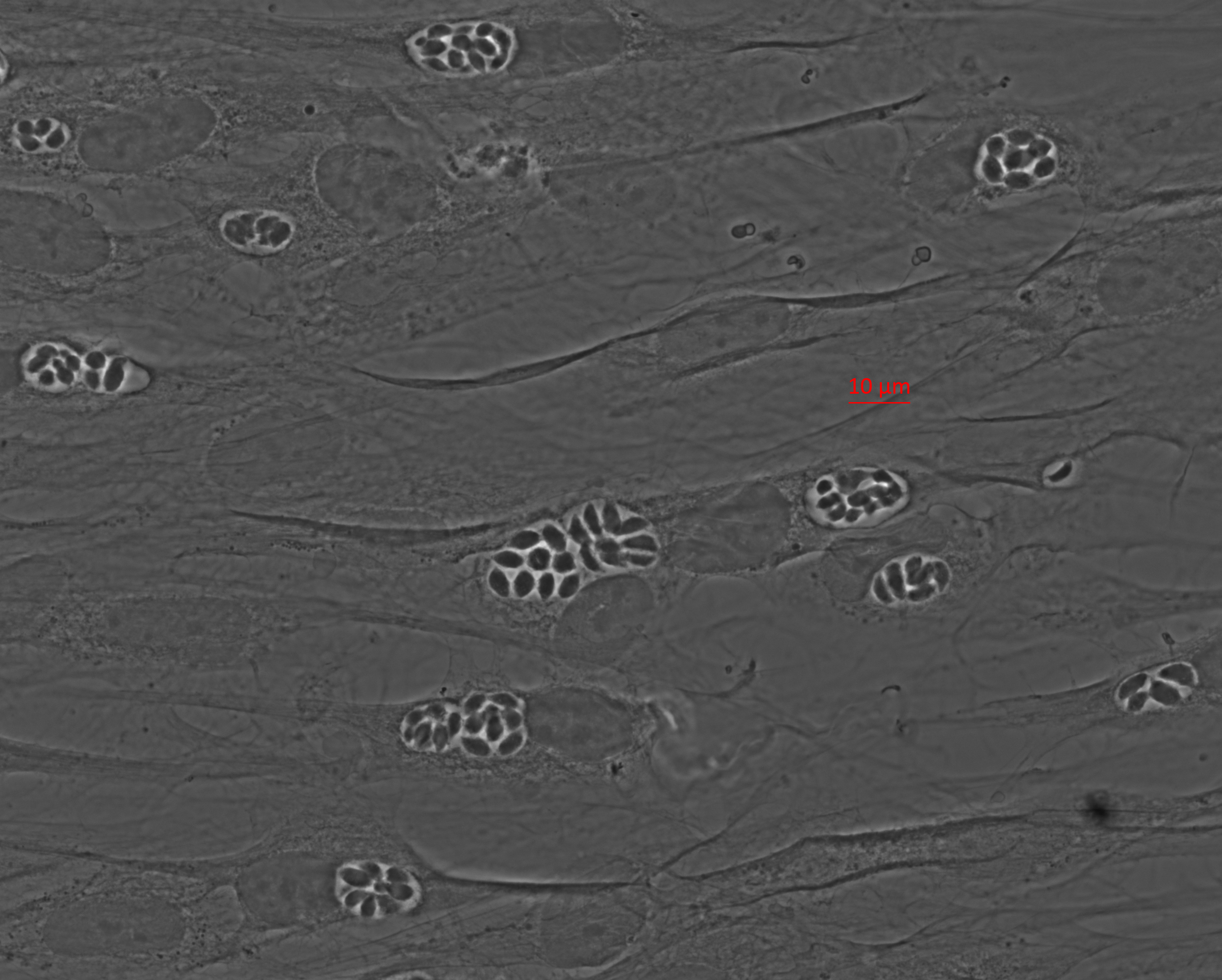

Supplement: Supplementary file 9 — Source data Fig. 3 [file 44321_2025_252_MOESM9_ESM.zip › Figure 3 Source Data/3a/Pru MORC KD BFD1 KO/Dolichos red BCLA green/UT/Snap-4017_c1 (Phase).tif]

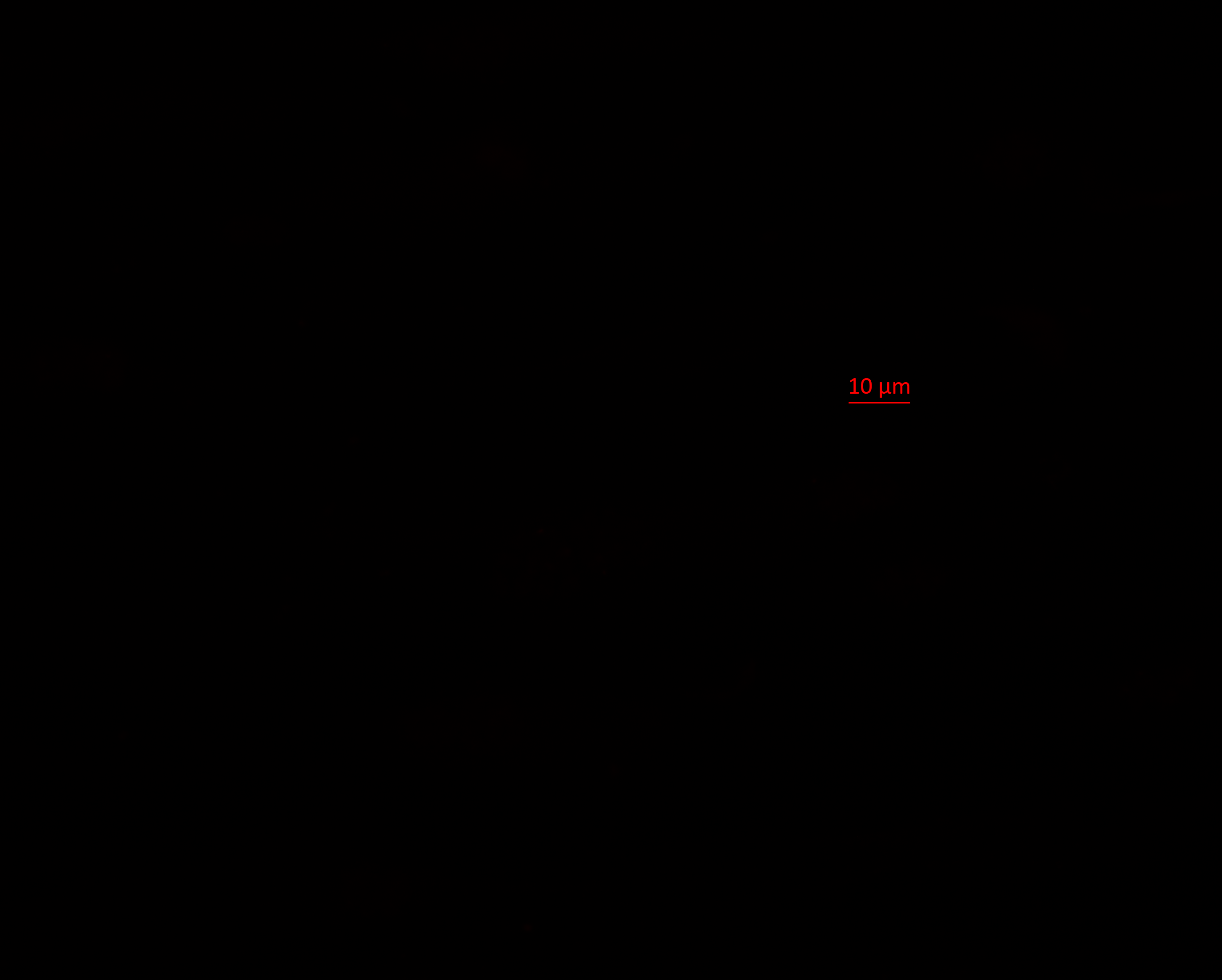

Supplement: Supplementary file 9 — Source data Fig. 3 [file 44321_2025_252_MOESM9_ESM.zip › Figure 3 Source Data/3a/Pru MORC KD BFD1 KO/Dolichos red BCLA green/UT/Snap-4017_c4 (Dolichos).tif]

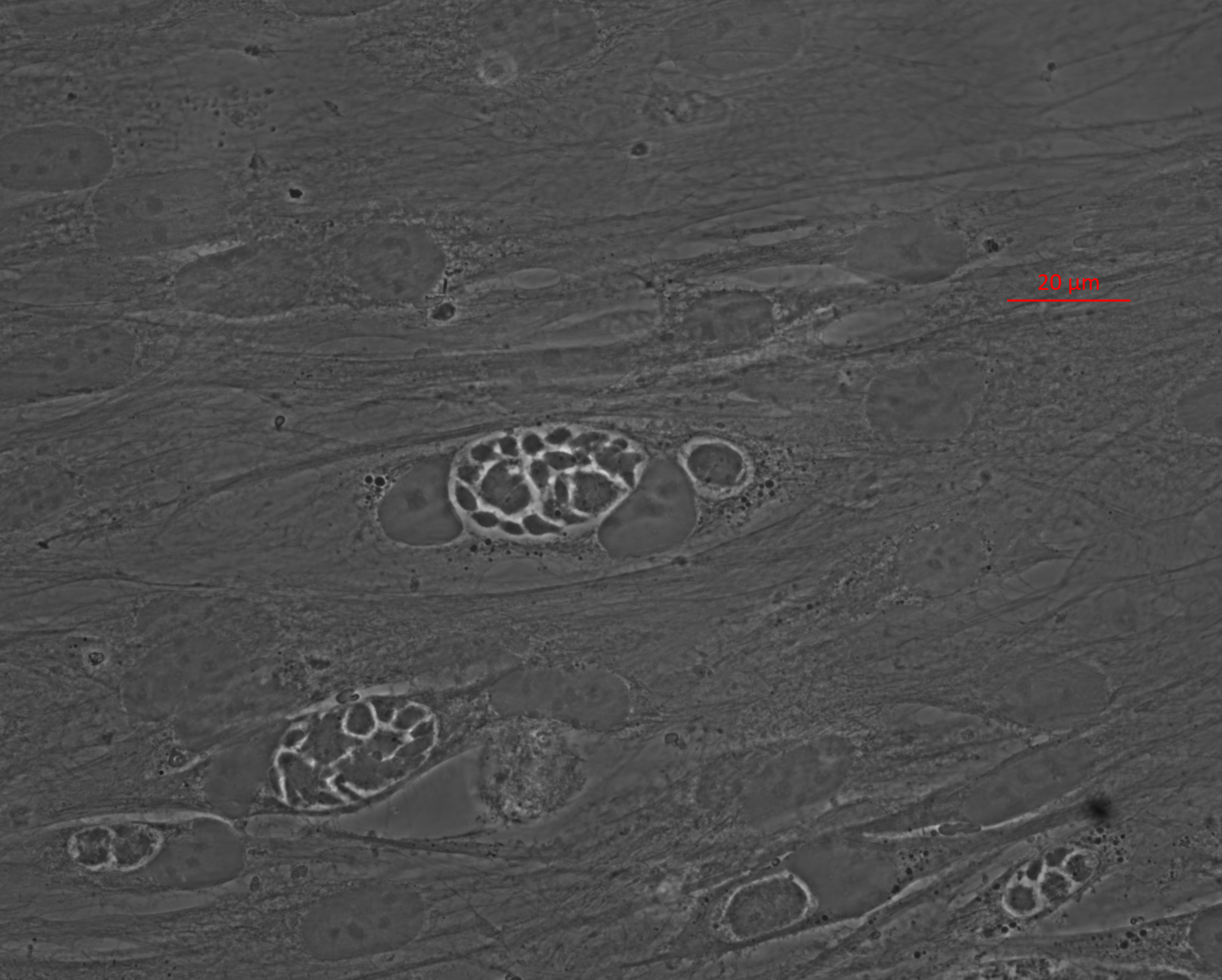

Supplement: Supplementary file 9 — Source data Fig. 3 [file 44321_2025_252_MOESM9_ESM.zip › Figure 3 Source Data/3a/Pru MORC KD BFD1 KO/Dolichos red BSM green/IAA/Snap-3987_c1 (Phase).tif]

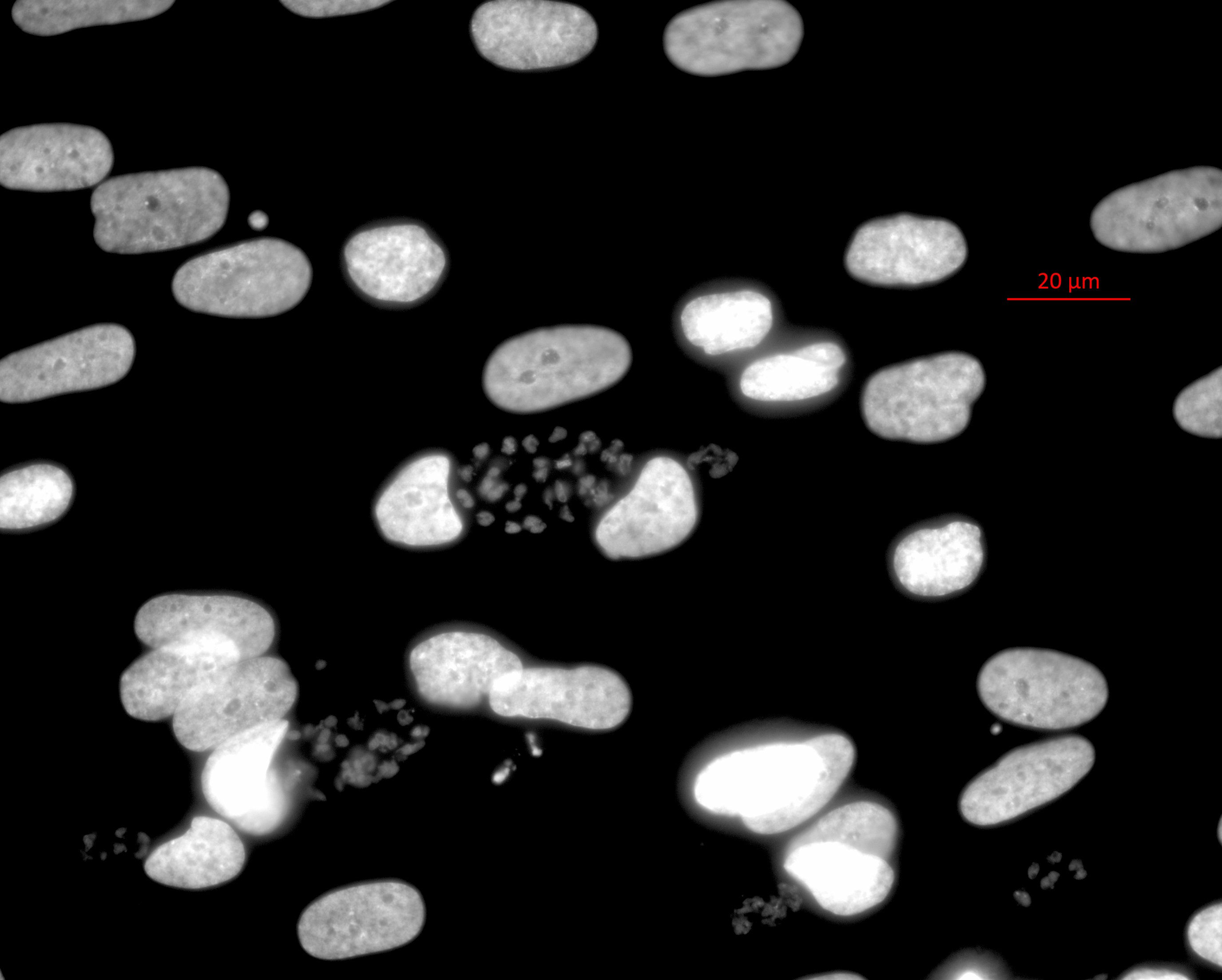

Supplement: Supplementary file 9 — Source data Fig. 3 [file 44321_2025_252_MOESM9_ESM.zip › Figure 3 Source Data/3a/Pru MORC KD BFD1 KO/Dolichos red BSM green/IAA/Snap-3987_c2 (DNA).tif]

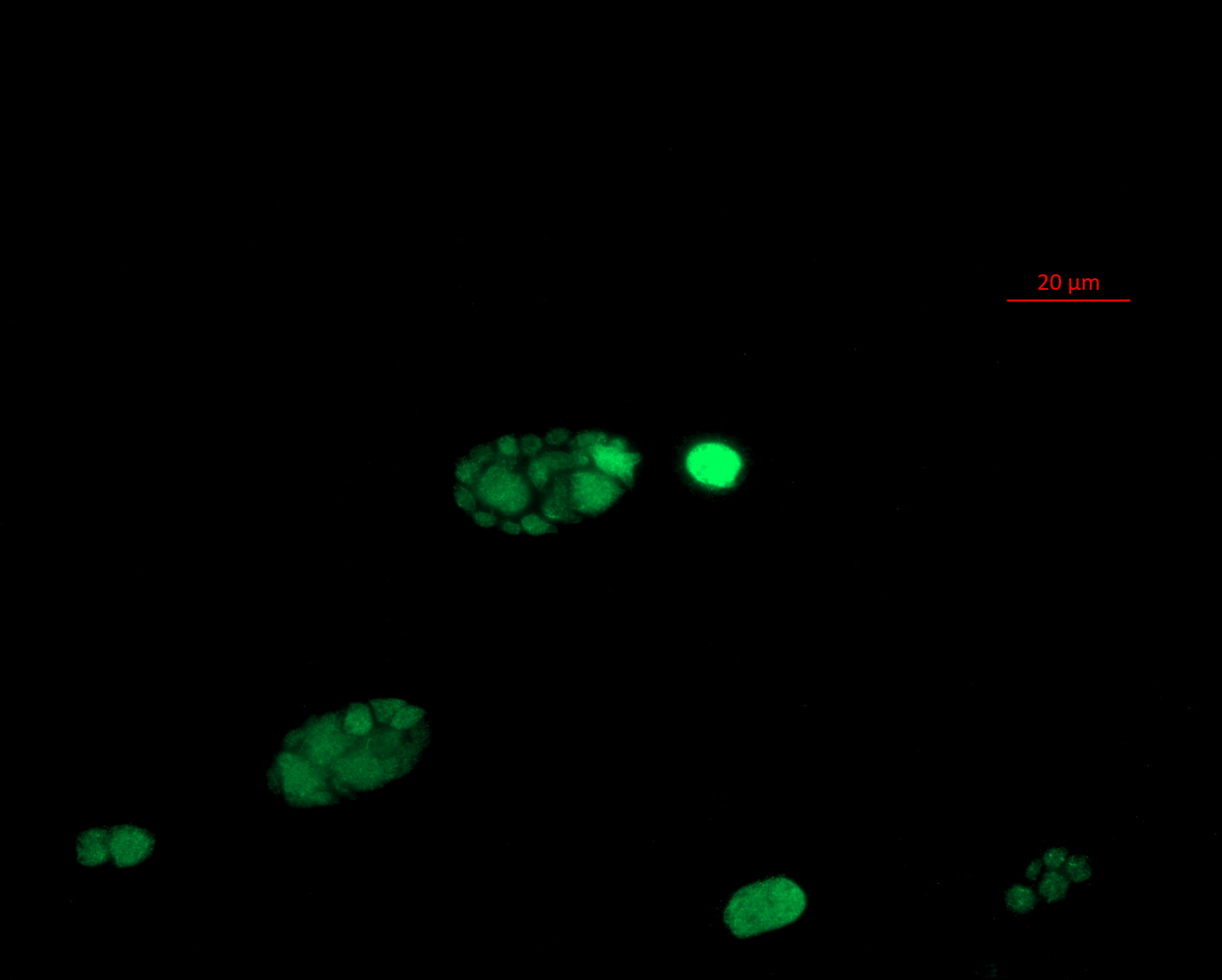

Supplement: Supplementary file 9 — Source data Fig. 3 [file 44321_2025_252_MOESM9_ESM.zip › Figure 3 Source Data/3a/Pru MORC KD BFD1 KO/Dolichos red BSM green/IAA/Snap-3987_c3 (BSM).tif]

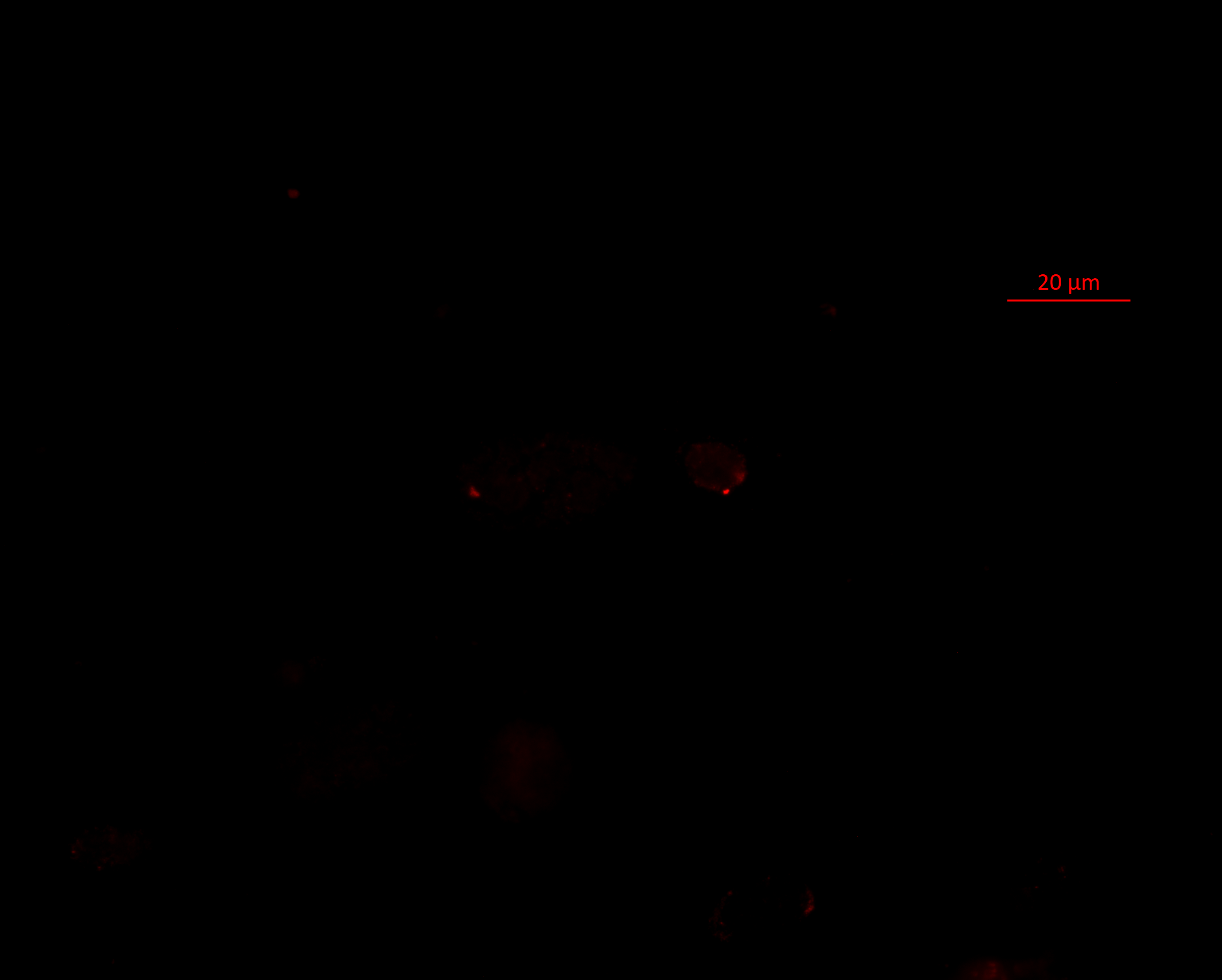

Supplement: Supplementary file 9 — Source data Fig. 3 [file 44321_2025_252_MOESM9_ESM.zip › Figure 3 Source Data/3a/Pru MORC KD BFD1 KO/Dolichos red BSM green/IAA/Snap-3987_c4 (Dolichos).tif]

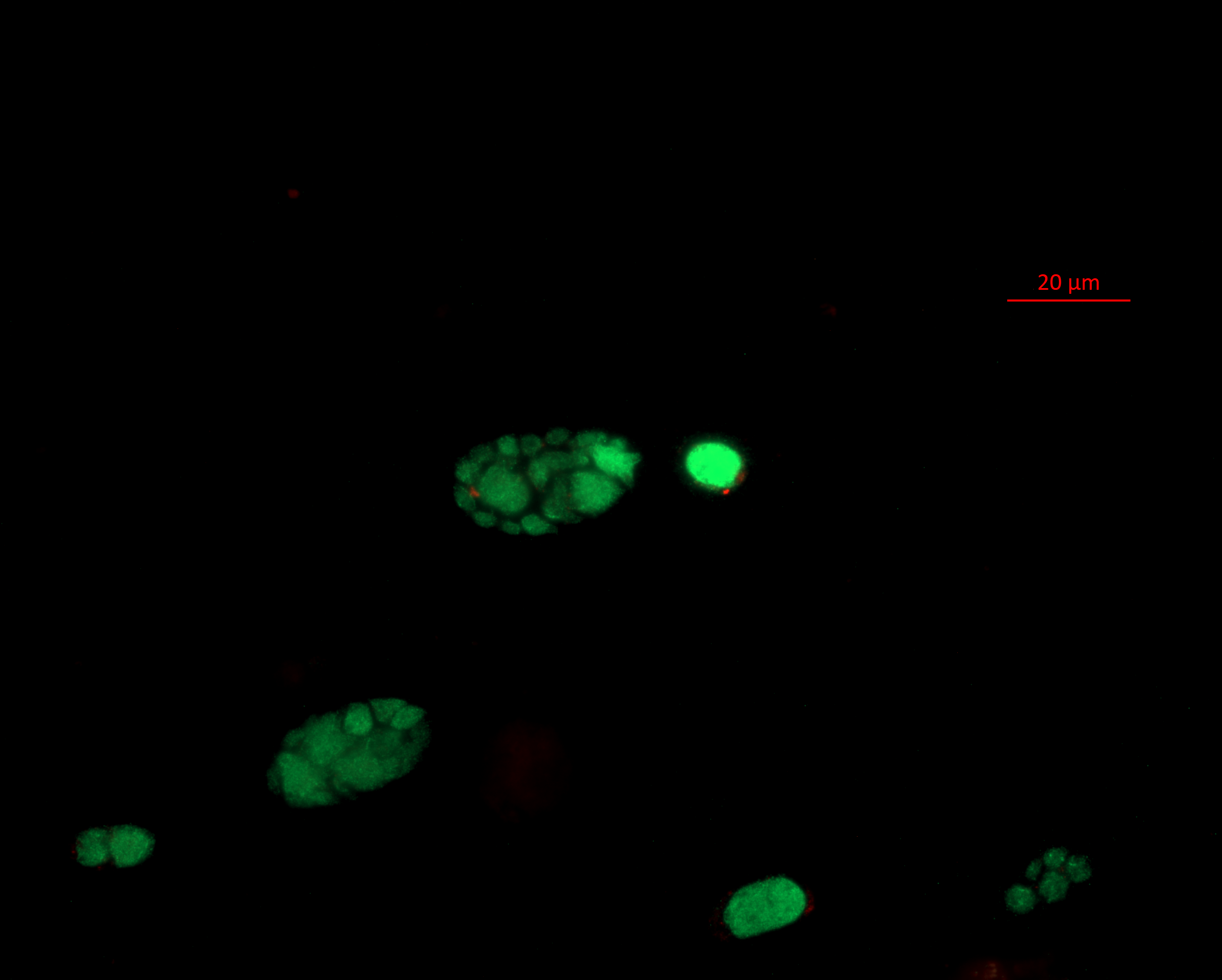

Supplement: Supplementary file 9 — Source data Fig. 3 [file 44321_2025_252_MOESM9_ESM.zip › Figure 3 Source Data/3a/Pru MORC KD BFD1 KO/Dolichos red BSM green/IAA/Snap-3987_c3+4.tif]

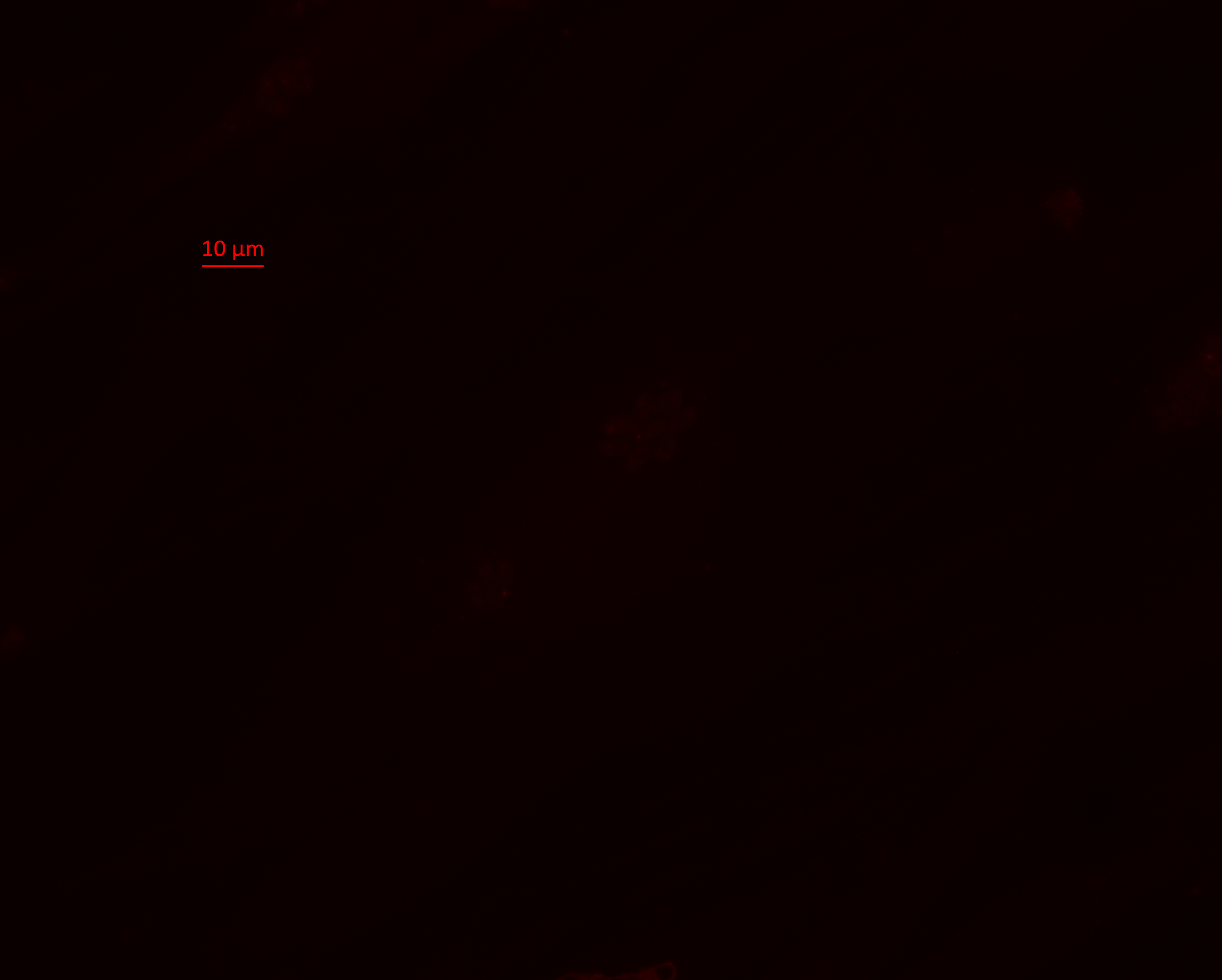

Supplement: Supplementary file 9 — Source data Fig. 3 [file 44321_2025_252_MOESM9_ESM.zip › Figure 3 Source Data/3a/Pru MORC KD BFD1 KO/Dolichos red BSM green/UT/Snap-3990_c4 (Dolichos).tif]

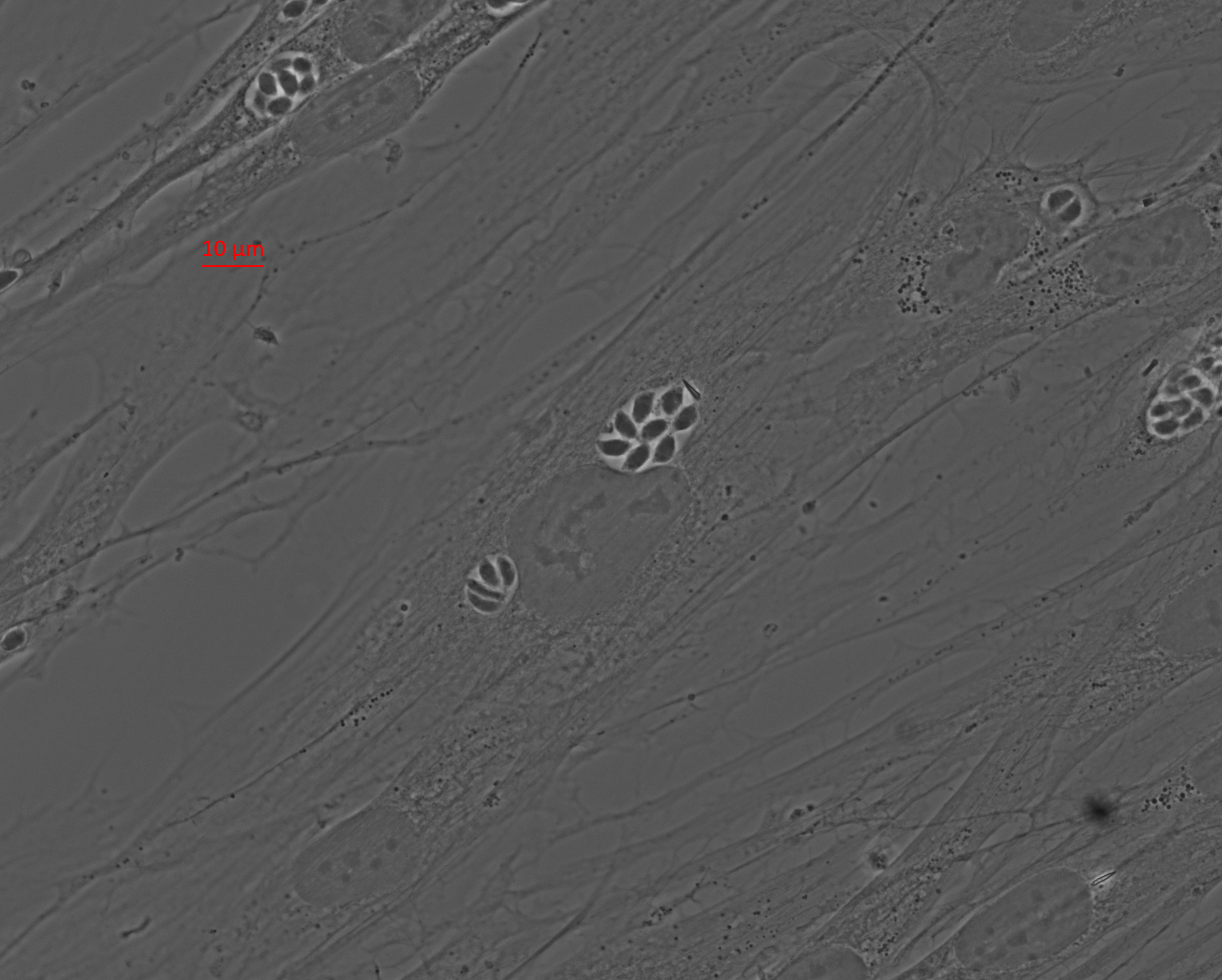

Supplement: Supplementary file 9 — Source data Fig. 3 [file 44321_2025_252_MOESM9_ESM.zip › Figure 3 Source Data/3a/Pru MORC KD BFD1 KO/Dolichos red BSM green/UT/Snap-3990_c1 (Phase).tif]

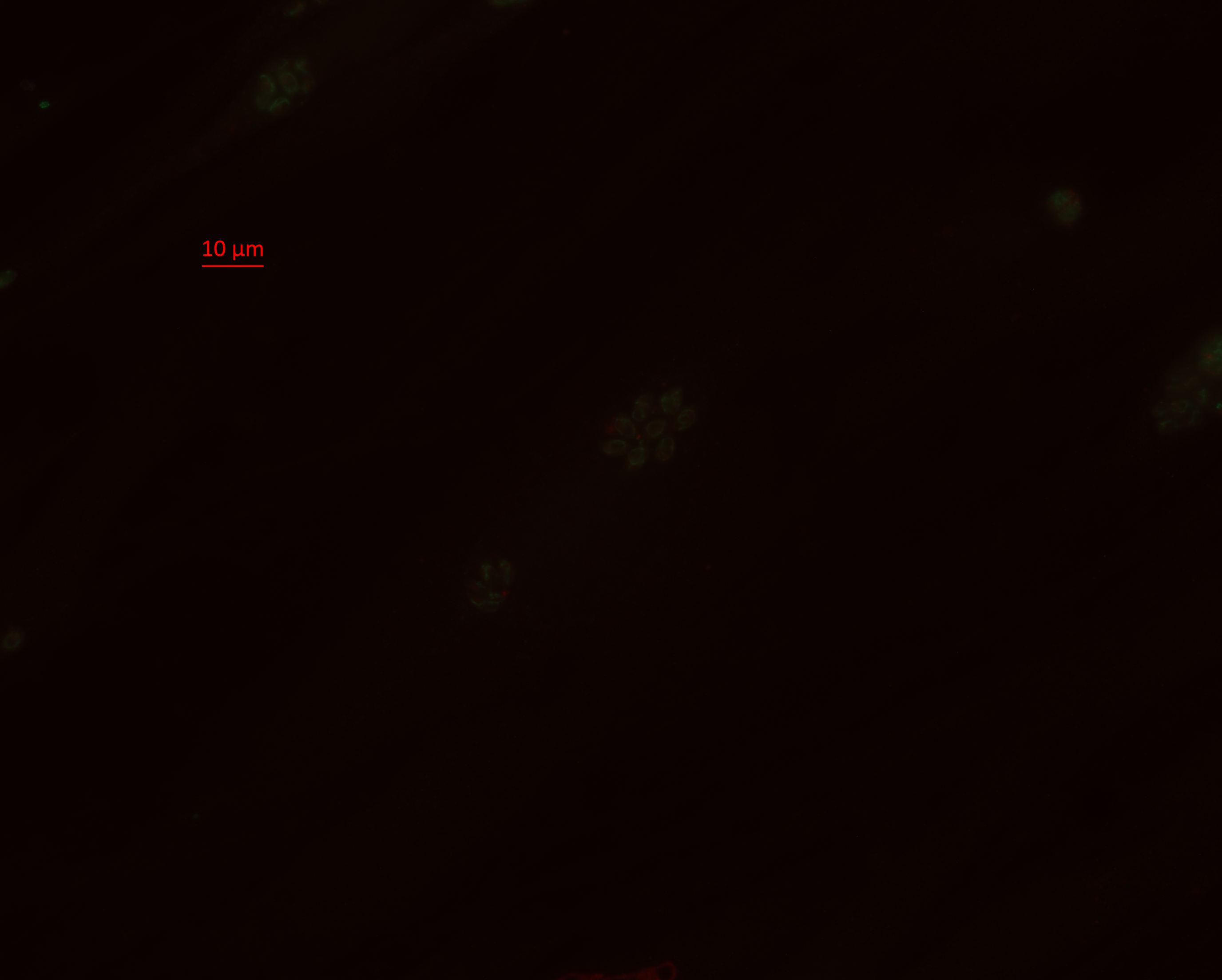

Supplement: Supplementary file 9 — Source data Fig. 3 [file 44321_2025_252_MOESM9_ESM.zip › Figure 3 Source Data/3a/Pru MORC KD BFD1 KO/Dolichos red BSM green/UT/Snap-3990_c3+4.tif]

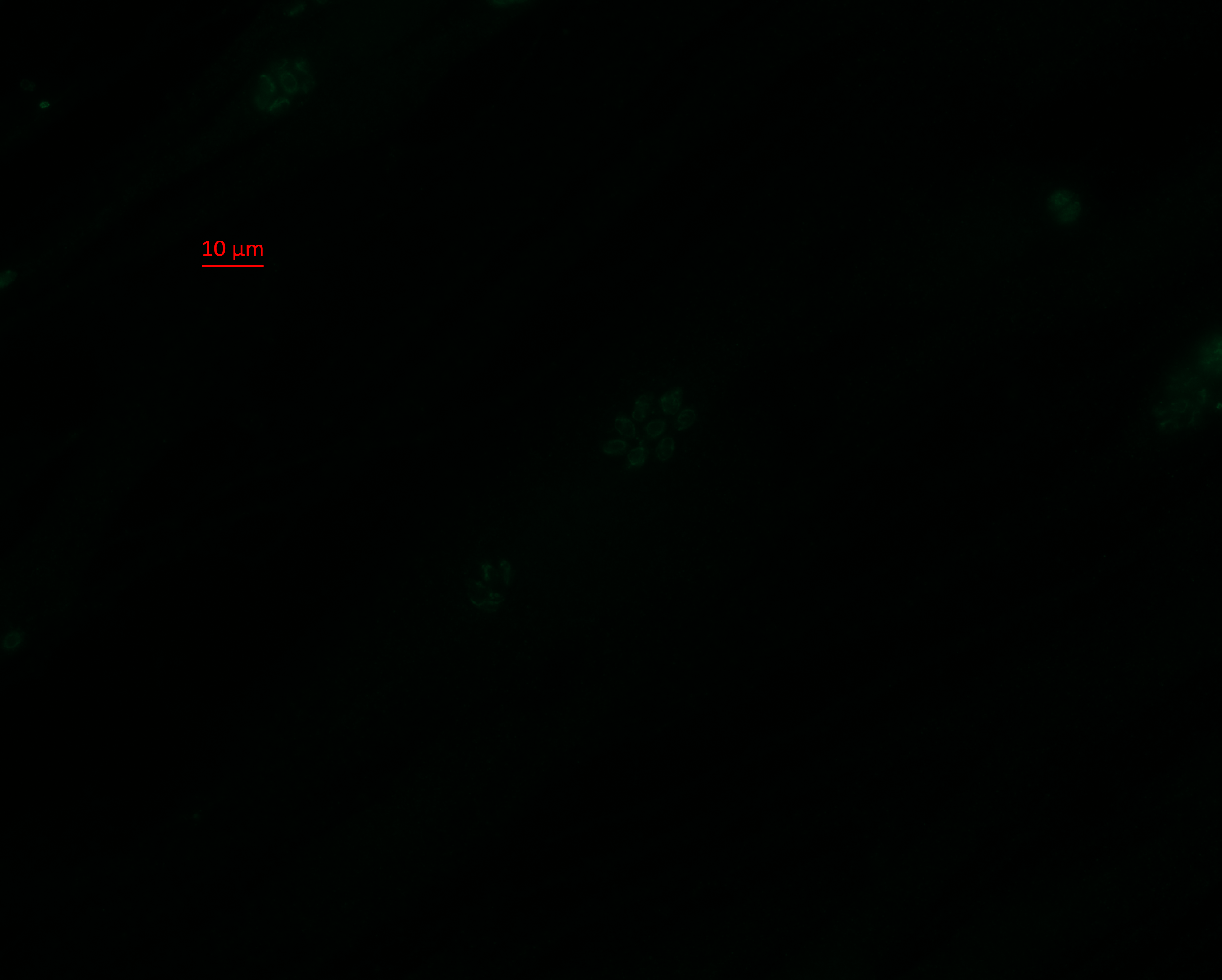

Supplement: Supplementary file 9 — Source data Fig. 3 [file 44321_2025_252_MOESM9_ESM.zip › Figure 3 Source Data/3a/Pru MORC KD BFD1 KO/Dolichos red BSM green/UT/Snap-3990_c3 (BSM).tif]

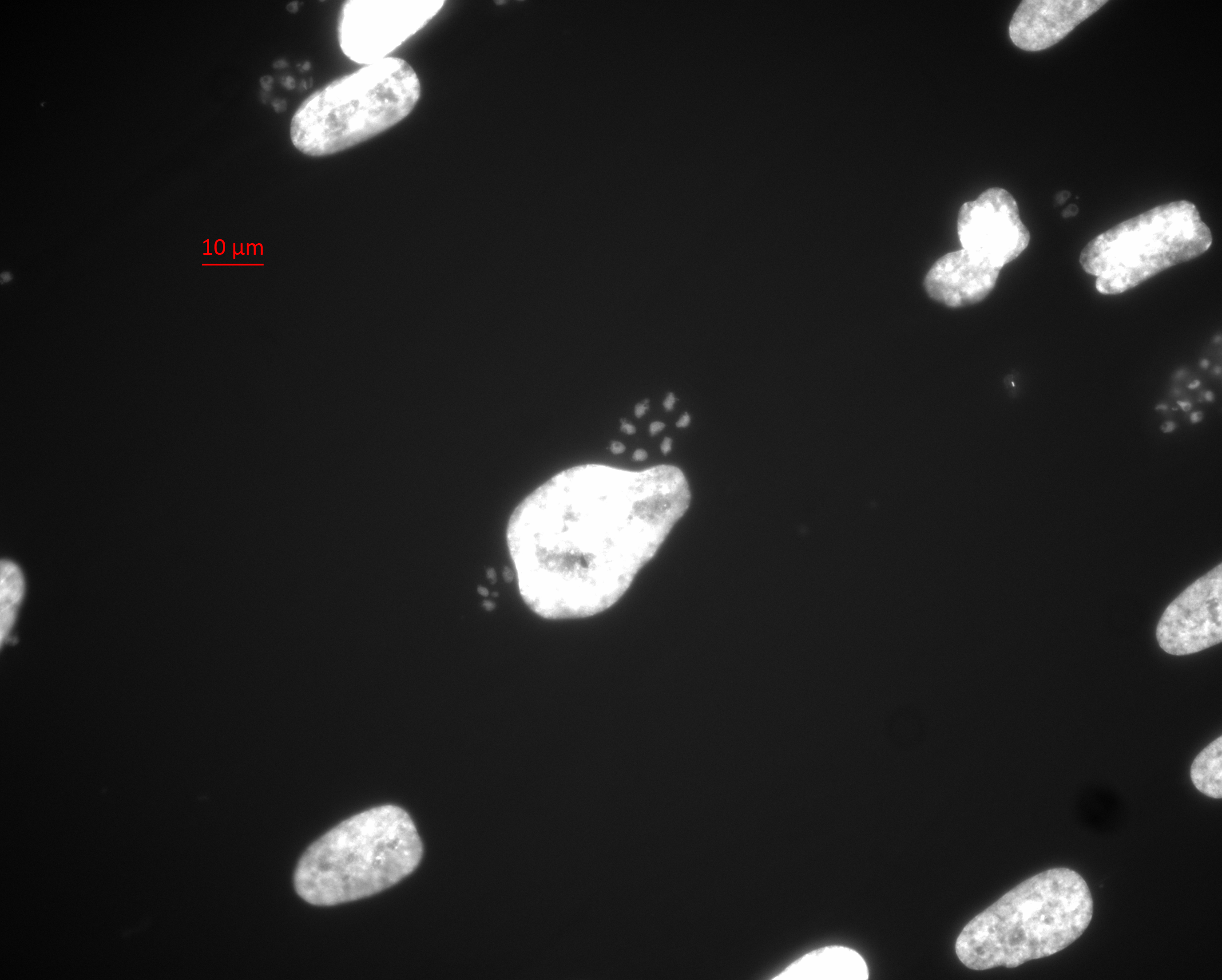

Supplement: Supplementary file 9 — Source data Fig. 3 [file 44321_2025_252_MOESM9_ESM.zip › Figure 3 Source Data/3a/Pru MORC KD BFD1 KO/Dolichos red BSM green/UT/Snap-3990_c2 (DNA).tif]

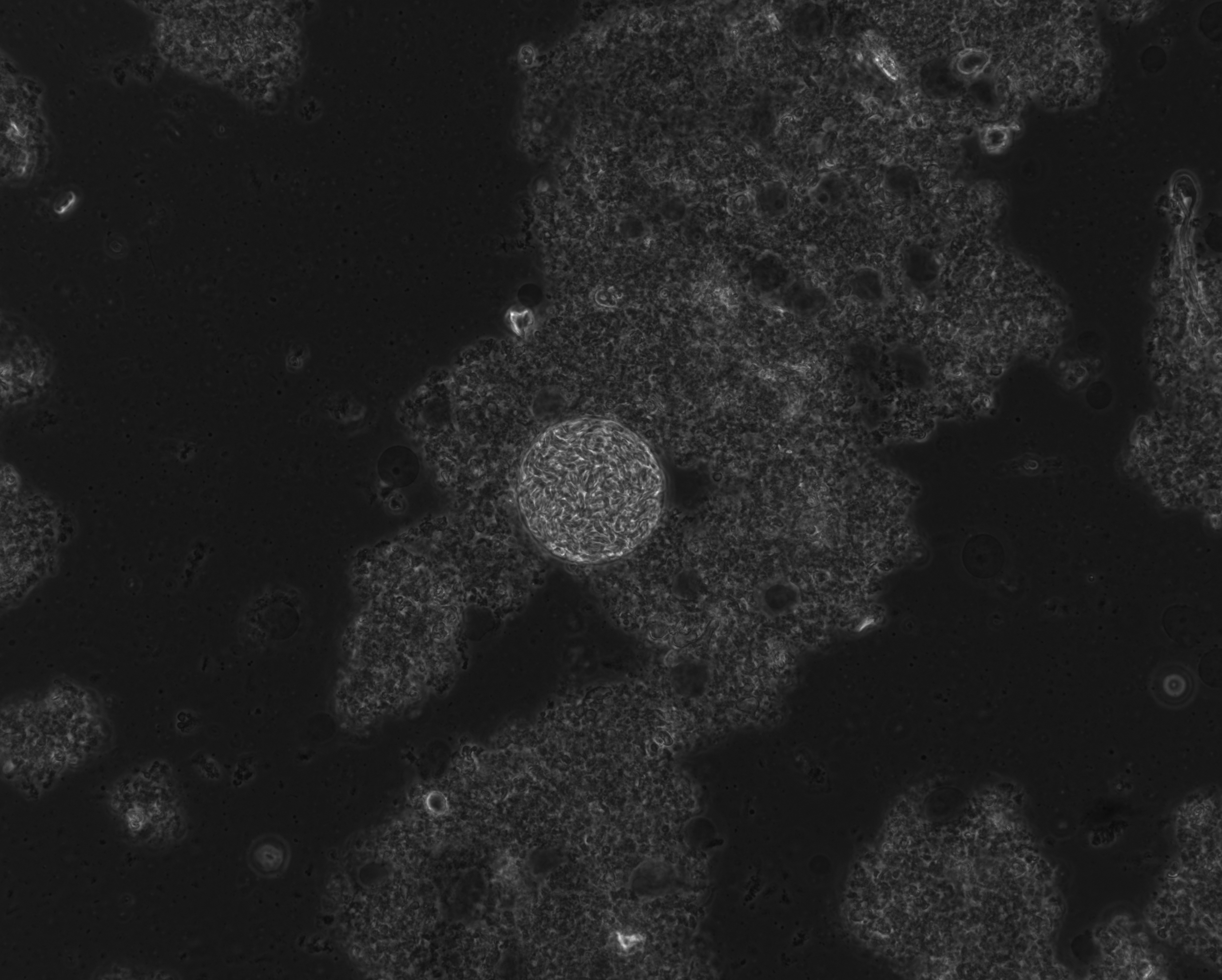

Supplement: Supplementary file 10 — Source data Fig. 5 [file 44321_2025_252_MOESM10_ESM.zip › Figure 5 Source Data/5b/76 BSM KO.tiff]

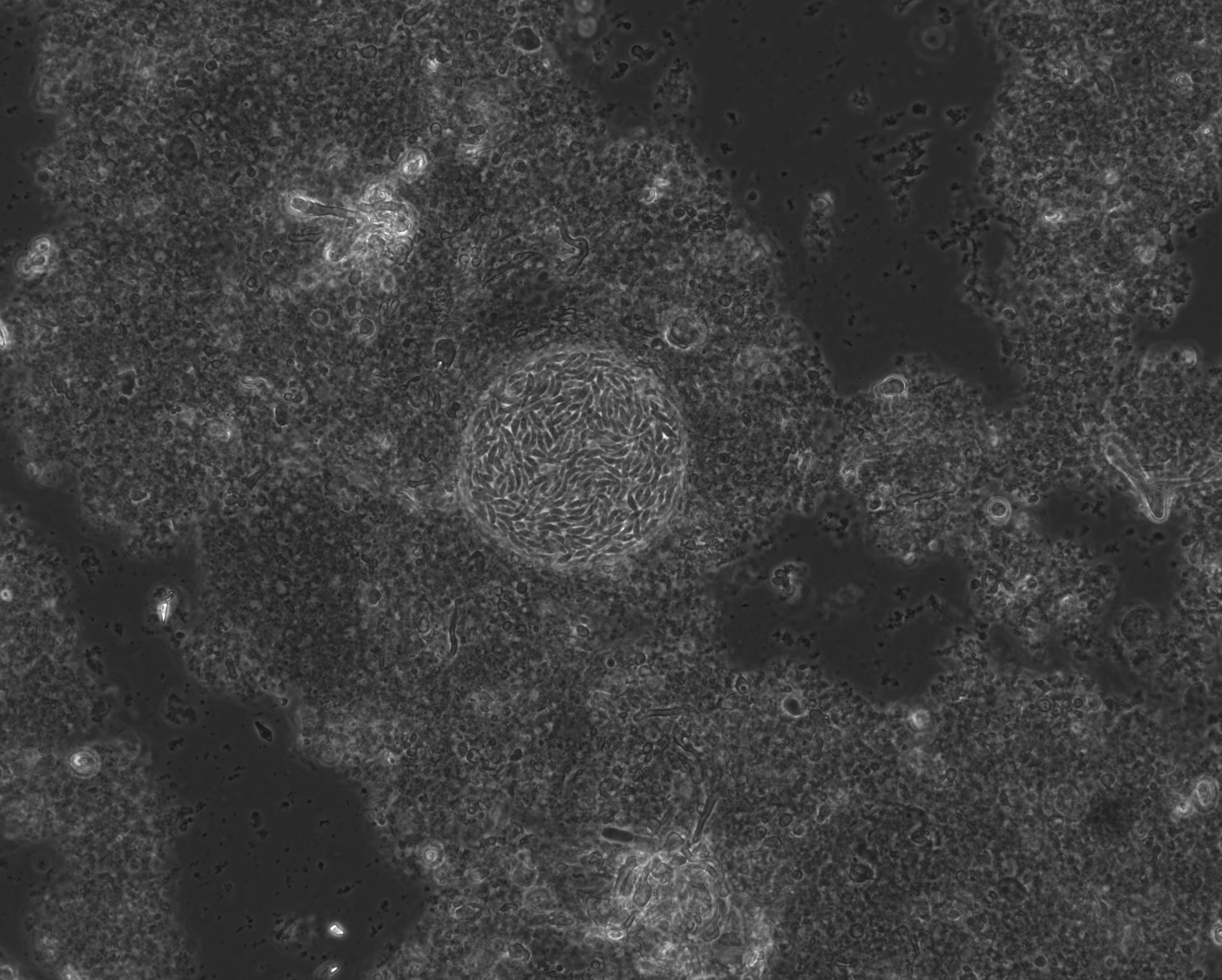

Supplement: Supplementary file 10 — Source data Fig. 5 [file 44321_2025_252_MOESM10_ESM.zip › Figure 5 Source Data/5b/76K WT.tiff]
