# Supplementary material for: Uncovering biomarkers for chronic toxoplasmosis detection highlights alternative pathways shaping parasite dormancy
Source: EMBO Mol Med. 2025 May 19;17(7):1686–715. doi: 10.1038/s44321-025-00252-0 (PMC12254245; doi:10.1038/s44321-025-00252-0)
Supplement: Supplementary file 12 — Expanded View Figures [file 44321_2025_252_MOESM12_ESM.pdf]

Expanded View Figures

Figure EV1. Identification of BSM in the MORC-depleted bradyzoite-enriched proteome.

(**A**) Purification scheme. MORC-depleted extract was fractionated by chromatography as described in the Methods section. (**B**) Western blot analysis of the initial purification steps and S200 gel filtration fractions (F), using serum from an NMRI mouse chronically infected with the 76 K cystogenic strain of *Toxoplasma gondii*. (**C**) Histogram showing the expression levels of BSM (*TGME49_202020*) and *TGME49_216140* following MORC depletion or HDAC3 inhibition with FR235222. Transcript abundance is also displayed across various in vivo stages, including merozoites, EES1–EES5 stages, tachyzoites, sporozoites, and cysts. (**D**) The MORC KD strain was modified to express BSM or the protein encoded by TGME49_216140 with a C-terminal HA-Flag tag. Expression was assessed by IFA in untreated or IAA-treated parasites (24 h). Chimeric proteins were detected using FLAG staining (red). (**E**, **F**) FLAG immunoprecipitation eluates (**E**) of TGME49_216140 (**E**) or BSM (**F**) were probed with cyst-bearing mouse sera and/or FLAG antibodies.

 © The Author(s)

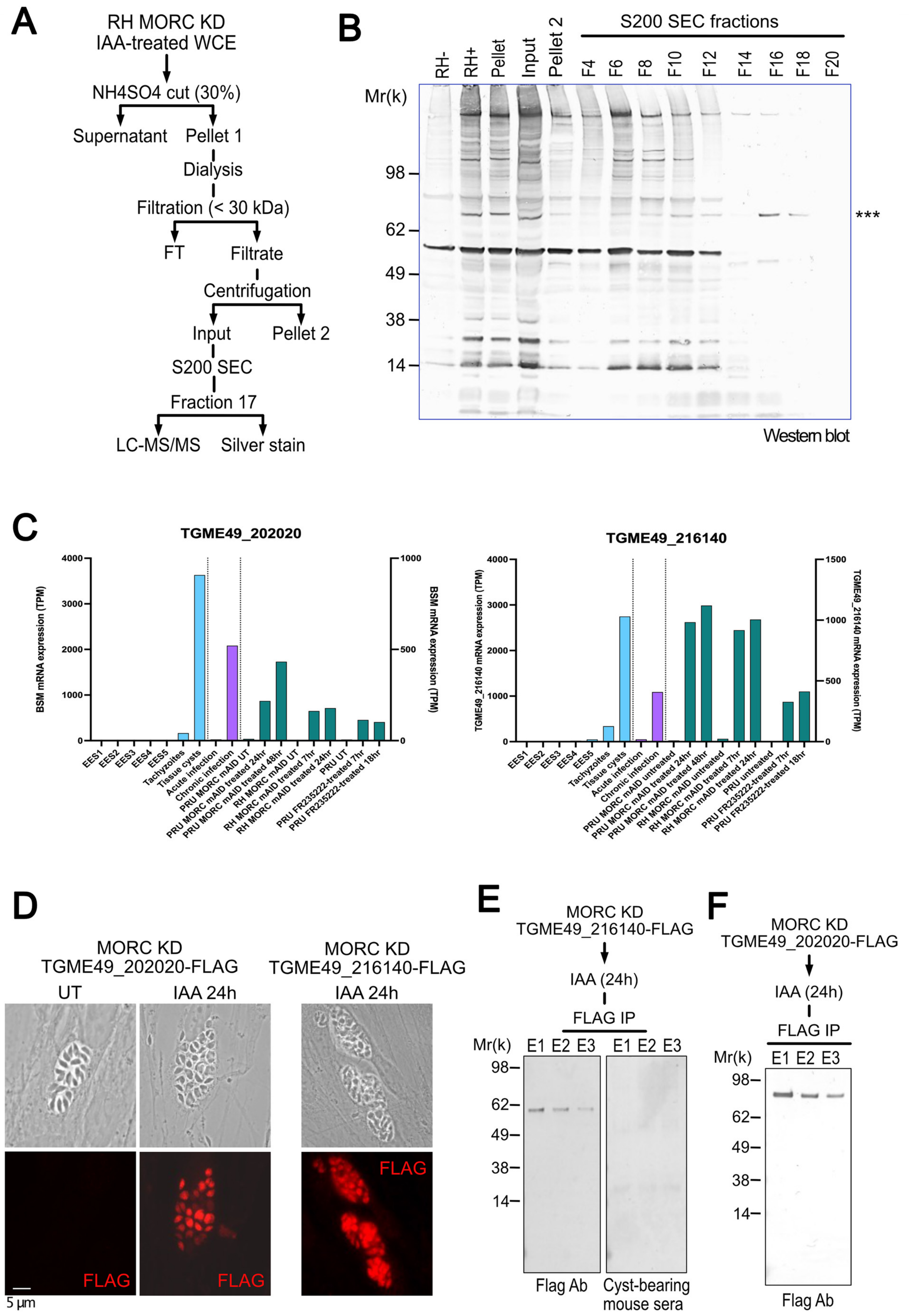
A
RH MORC KD
IAA-treated WCE
NH4SO4 cut (30%)
Supernatant
Pellet 1
Dialysis
Filtration (< 30 kDa)
FT
Filtrate
Centrifugation
Input
Pellet 2
S200 SEC
Fraction 17
LC-MS/MS
Silver stain
B
S200 SEC fractions
RH-
RH+
Pellet
Input
Pellet 2
F4
F6
F8
F10
F12
F14
F16
F18
F20
Mr(k)
98
62
49
38
14
***
Western blot
C
TGME49_202020
BSM mRNA expression (TPM)
TGME49_216140
TGME49_216140 mRNA expression (TPM)
D
MORC KD
TGME49_202020-FLAG
UT
IAA 24h
MORC KD
TGME49_216140-FLAG
IAA 24h
FLAG
5 µm
E
MORC KD
TGME49_216140-FLAG
IAA (24h)
FLAG IP
E1 E2 E3
Flag Ab
Cyst-bearing
mouse sera
F
MORC KD
TGME49_202020-FLAG
IAA (24h)
FLAG IP
E1 E2 E3
Flag Ab

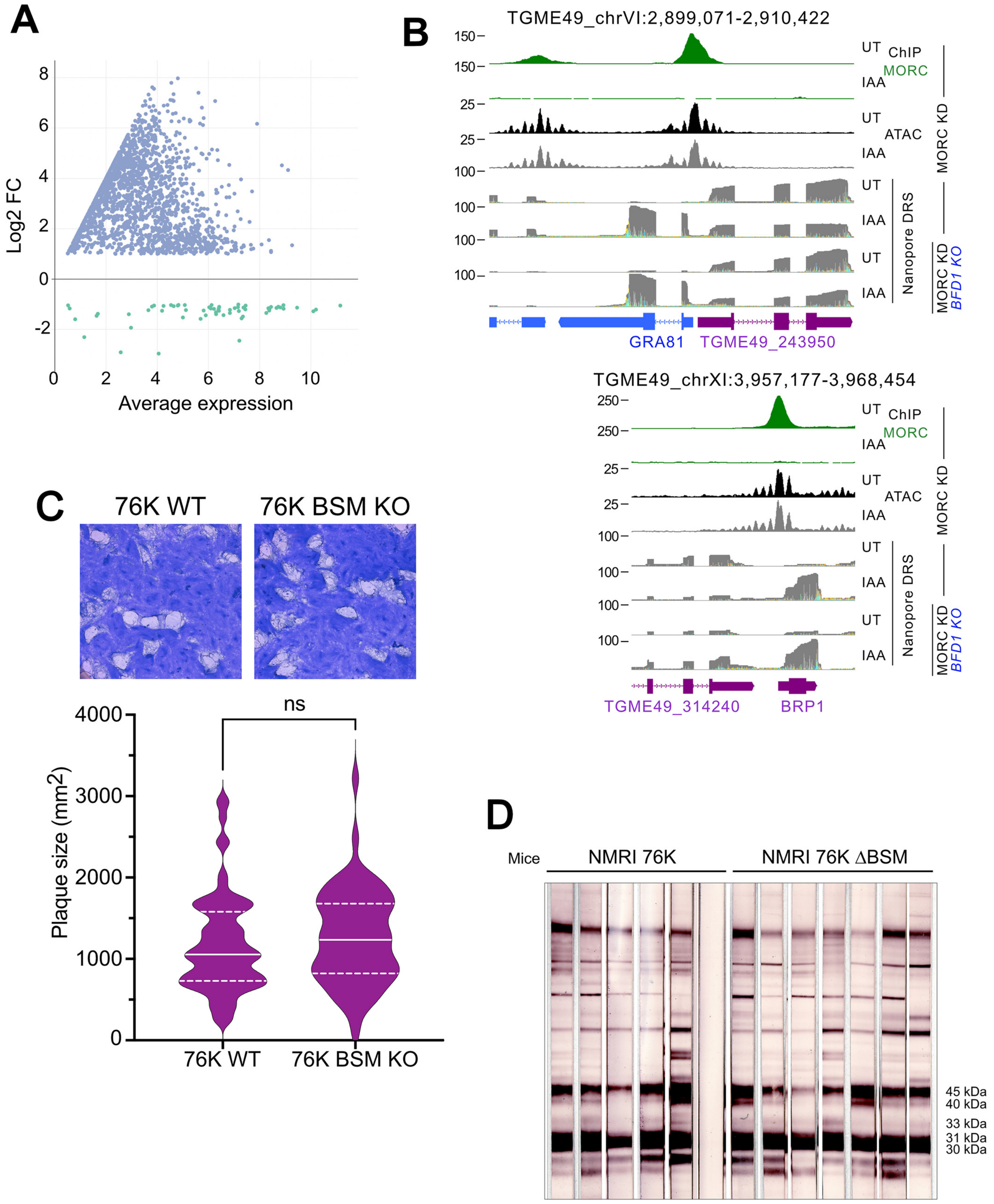
A
Log2 FC
Average expression
B
TGME49_chrVI:2,899,071-2,910,422
UT
IAA
ChIP
MORC
ATAC
Nanopore DRS
MORC KD
MORC KD
BFD1 KO
GRA81
TGME49_243950
TGME49_chrXI:3,957,177-3,968,454
TGME49_314240
BRP1
C
76K WT
76K BSM KO
ns
Plaque size (mm2)
D
Mice
NMRI 76K
NMRI 76K ΔBSM
45 kDa
40 kDa
33 kDa
31 kDa
30 kDa

 © The Author(s)

Figure EV2. MORC regulome and BSM serology.

(**A**) MA plots display Log2(FC) against Log2(mean expression) for genes before and after MORC depletion, using DESeq2. Upregulated genes (log2[FC] > 1, *P* value < 0.05) are blue, downregulated (log2[FC] < -0.58, *P* value < 0.05) are green. (**B**) IGB screenshots of representative genes expressed in merozoite (GRA81) or in merozoite/bradyzoite (BRP1) displaying ChIP-seq signal for MORC (HA antibody, green) in MORC KD strains under untreated and IAA-treated conditions. ATAC-seq profiles for both conditions, showing Tn5 transposase accessibility with read density on the y axis, are included. Nanopore DRS data for MORC KD and MORC KD/*BFD1* KO strains, untreated and IAA-treated, are also shown. (**C**) The effects of deletion of *BSM* on the lytic cycle were determined by plaque assay. After 7 days, the cells were fixed and stained with Coomassie blue to detect the presence of plaques (top panel). Graphs below show the distribution of the size of visible plaques ($n = 50$ per condition). Statistical analyses were performed using Mann–Whitney test. (**D**) LDBIO TOXO II IgG western blot membranes were probed with sera from NMRI mice 8 weeks post-infection with the 76 K wild-type strain ($n = 6$; two sera were insufficient) or ΔBSM ($n = 7$; one mouse died before 8 weeks). A test is considered positive if at least three of the 30, 31, 33, 40, and 45 kDa bands are present, including the 30 kDa band.

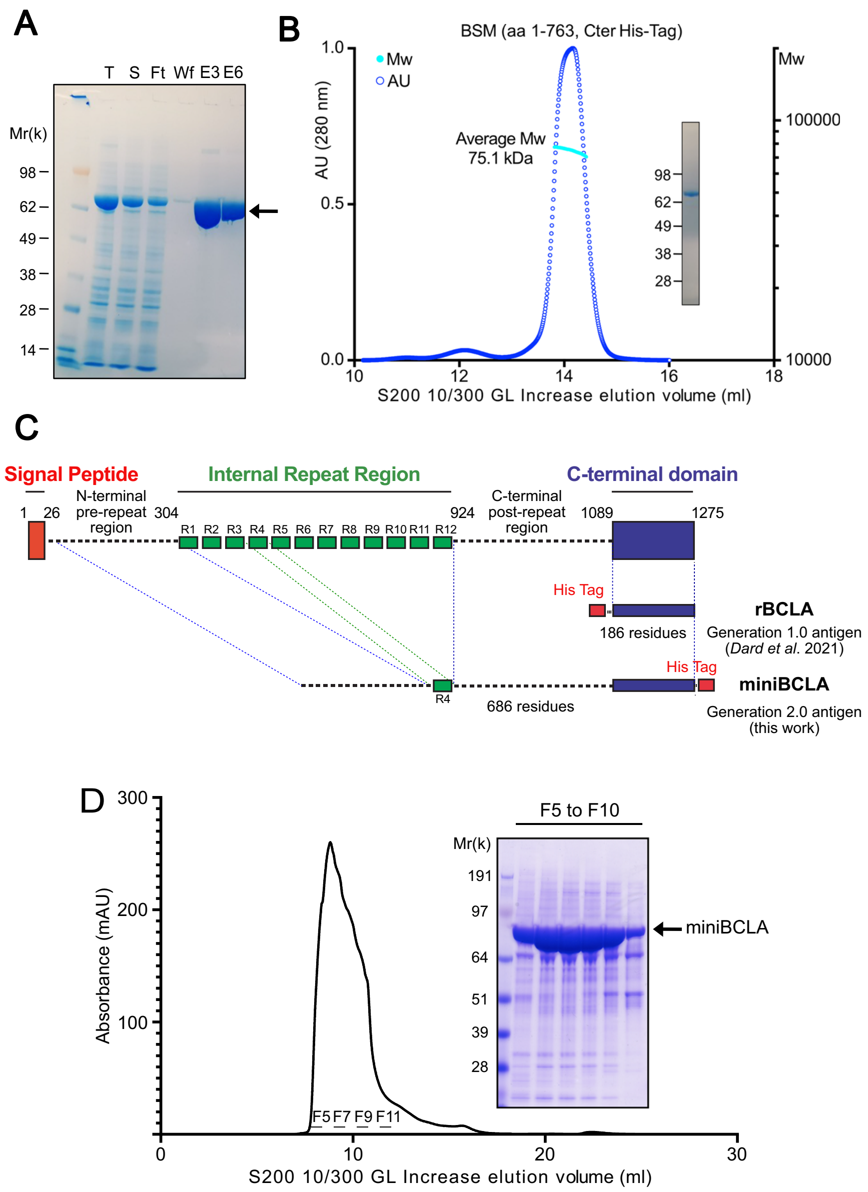

Figure EV3. Recombinant BSM purification steps.

(**A**) Nickel-nitrilotriacetic (Ni-NTA) elution. SDS-PAGE electrophoresis of total (T), soluble (S), flow though (Ft) and elution fractions 3 (E3) and 6 (E6). The black arrow points to the recombinant BSM protein. (**B**) Size Exclusion Chromatography using a S200 (10/300 Gl) combined to a Multi-Angle Laser Light Scattering analysis. Absorbance values are shown in deep blue while the predicted molecular weight plot (Mw) is displayed in light blue. (**C**) Schematic representation of rBCLA (1st generation of the antigen) and miniBCLA (this work) proteins. (**D**) Size exclusion chromatography of the purified miniBCLA antigen, 280 mm absorbance is shown as a function of volume. Peak elution fractions F5 to F10 were analyzed by Coomassie blue stained 4–12% NuPAGE.

© The Author(s)
